# Carbene and photocatalyst-catalyzed decarboxylative radical coupling of carboxylic acids and acyl imidazoles to form ketones

**DOI:** 10.1038/s41467-022-30583-2

**Published:** 2022-05-23

**Authors:** Shi-Chao Ren, Xing Yang, Bivas Mondal, Chengli Mou, Weiyi Tian, Zhichao Jin, Yonggui Robin Chi

**Affiliations:** 1 Laboratory Breeding Base of Green Pesticide and Agricultural Bioengineering, Key Laboratory of Green Pesticide and Agricultural Bioengineering, Ministry of Education, Guizhou University, Huaxi District Guiyang, China; 2 Division of Chemistry & Biological Chemistry, School of Physical & Mathematical Sciences, Nanyang Technological University, Singapore, 637371 Singapore; 3 Guizhou University of Traditional Chinese Medicine, Guiyang, 550025 China

**Keywords:** Synthetic chemistry methodology, Organocatalysis, Photocatalysis

## Abstract

The carbene and photocatalyst co-catalyzed radical coupling of acyl electrophile and a radical precursor is emerging as attractive method for ketone synthesis. However, previous reports mainly limited to prefunctionalized radical precursors and two-component coupling. Herein, an *N*-heterocyclic carbene and photocatalyst catalyzed decarboxylative radical coupling of carboxylic acids and acyl imidazoles is disclosed, in which the carboxylic acids are directly used as radical precursors. The acyl imidazoles could also be generated in situ by reaction of a carboxylic acid with CDI thus furnishing a formally decarboxylative coupling of two carboxylic acids. In addition, the reaction is successfully extended to three-component coupling by using alkene as a third coupling partner via a radical relay process. The mild conditions, operational simplicity, and use of carboxylic acids as the reacting partners make our method a powerful strategy for construction of complex ketones from readily available starting materials, and late-stage modification of natural products and medicines.

## Abstract (web summary)

The combination of carbene- and photocatalysis has enabled unorthodox routes to ketone syntheses, but usually requires engineered or activated substrates. Herein the authors present a carbene- and photocatalytic decarboxylative radical coupling of carboxylic acids and acyl imidazoles, in which the carboxylic acids are directly used as radical precursors.

## Introduction

**Fig. 1:**
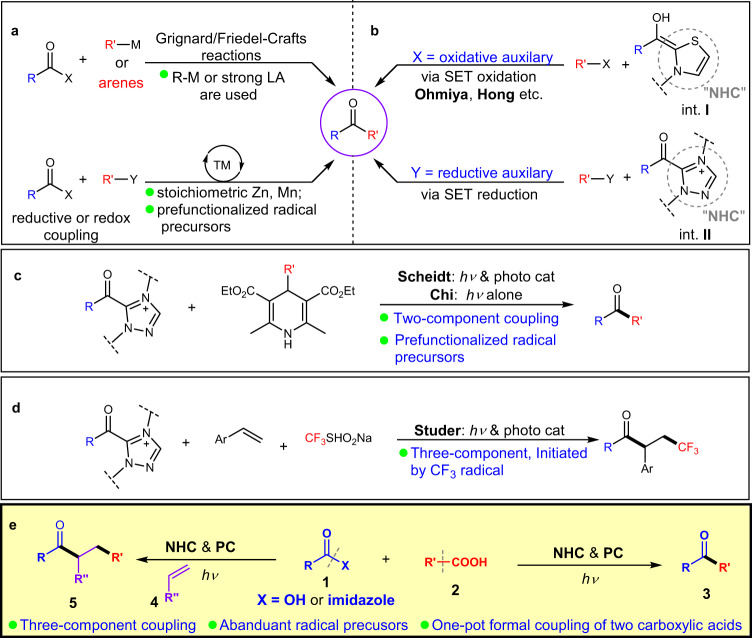
Synthesis of ketones from carboxylic acid derivatives. **a** Conventional and metal-catalyzed methods for ketone synthesis. TM = transition metal. LA = Lewis Acid. **b** Earlier ketone synthesis involving NHC-catalyzed radical process. **c** Two-component coupling using prefunctionalized radical precursors via NHC/photo catalysis. **d** CF_3_ radical initiated three-component coupling. **e** This work: Two/three-component radical coupling for ketone synthesis using carboxylic acids as radical precursors. PC = photo catalyst.

Ketones are basic structural motifs in natural and synthetic molecules with broad applications in nearly all areas involving chemicals, such as medicines, agrochemicals, and materials^1,2^. Searching for new synthetic strategies for efficient access to ketones, in either small or complex molecules, remains as an active topic in modern chemistry. The ketones can be prepared via oxidation of hydrocarbons or alcohols, especially when the molecules are relatively simple and chemo-selectivity issues are minimal. Alternatively, ketones can be prepared from the corresponding carboxylic esters and their derivatives via an overall reductive process^3^. Traditional methods include reactions of acyl electrophiles with organometallic reagents (such as Grignard reagent) or electron-rich arenes (Friedel–Crafts reactions, under catalysis of Brønsted or Lewis acids) (Fig. 1a, top)^3–6^. Metal-catalyzed couplings of acyl electrophiles with nucleophiles^7–11^ or electrophiles^12^ have also been developed (Fig. 1a, bottom). In these traditional or metal-catalyzed methods, the key ketone formation step is either an electron-pair-transfer process or a reductive elimination process of a metal intermediate. In contrast, catalytic formation and direct coupling of two radical intermediates to form ketones are much less studied. The impressive relevant successes are either initiated by a single radical intermediate or mostly involve transition metal-catalyzed processes (with metal-carbon bond formed in the key intermediates). Examples include reactions of carboxylic acids and their derivatives with alkenes via radical processes^13–18^ or nickel-catalyzed coupling reactions^19–22^ to prepare ketones. *N*-heterocyclic carbenes (NHCs) as organic catalysts^23–29^ are proved effective in generating radical intermediates^30–34^ for various reactions, as earlier disclosed by Scheidt^35,36^, Studer^37,38^, Rovis^39,40^, our laboratories^41,42^, Sun^43^, and Ye^44–48^. The NHC-derived radical intermediates are typically persistent radicals, and thus coupling with another transient radical become feasible^49^. As aforementioned^35–48^, this NHC-derived radical intermediates can be generated by single-electron oxidation of Breslow intermediate (int. **I**, Fig. 1b, top). For example, Ohmiya showed that single-electron-oxidation of the Breslow intermediate by redox active esters could lead to two radical intermediates that reacted with each other to eventually form ketones in 2019^50–55^. Hong reported that Breslow intermediate can also be oxidized by Katritzky pyridinium salts followed by deaminative cross-coupling to forge ketones^56^. Very recently, studies from Scheidt^57–59^, Studer^60–63^, and our laboratories^64^ found that NHC-derived azolium ester intermediates (int. **II**) can undergo single-electron-reduction to generate radical intermediates for further couplings to form ketones (Fig. 1b, bottom). Scheidt^57,58^ and our group^64^ used Hantzsch esters as precursors of the other transient radical intermediates to furnish two-component radical coupling reactions (Fig. 1c). Studer reported a trifluoromethyl radical initiated three-component coupling for the synthesis of β-Trifluoromethylated ketones (Fig. 1d)^62^. Merits and clear limitations exist in these previous methods^65–71^. For example, stoichiometric amount of metal reductants is needed in metal-catalyzed reductive cross coupling of acyl reagents with electrophiles such as alkyl halides and redox active esters. In metal-catalyzed redox coupling and recent NHC catalytic approaches, preparation and isolation of the reaction partners (such as redox active esters, Katritzky pyridinium salts, or Hantzsch esters, etc.) bring undesired operations and pose limitations on substrate scopes. In addition, despite the seminal work achieved in NHC and photocatalyst co-catalyzed ketone synthesis^57–64^, three-component radical relay coupling that involves various carbon radicals is still limited^58,72^.

Here we disclose an operationally simple strategy for coupling of acyl imidazoles and carboxylic acids to form ketones (Fig. 1e, right), in which the carboxylic acids were directly used as radical precursors. The acyl imidazoles could also be generated in situ via the reaction of a carboxylic acid with CDI (carbonyldiimidazole), thus provides a formally decarboxylative coupling of two carboxylic acids. Importantly, our NHC and photocatalyst co-catalyzed coupling reaction could be extended to three-component coupling reactions with alkenes as the third radical coupling partner via a radical relay process (Fig. 1e, left), providing a straightforward strategy to construct ketones with high levels of complexity from readily available materials. To demonstrate the utility of this method in synthetic chemistry, late-stage modification of marketed drugs and direct coupling of fragments of two medicinal molecules were performed.

## Results

### Optimization of reaction conditions

To simplify our condition optimizations, we first evaluated the coupling reactions between activated acyl electrophiles (**1a**, **1b**, **1c**) and carboxylic acid (**2a**) (Table 1). Built on our earlier studies on NHC-catalyzed reaction of carboxylic esters^73,74^, we first used nitrophenyl carboxylic ester **1a** as activated ester. A typical reaction mixture contained **1a** (1.5 equivalent), carboxylic acid **2a** (1.0 equivalent), an iridium complex (**PC-1**) as a photocatalyst (1 mol%), an NHC pre-catalyst (**A**, 20 mol%)^75^, and a base (Cs_2_CO_3_) in CH_3_CN as the solvent. The reaction was carried out under visible light (blue light LED, λ_max_ = 427 nm) at 30–40 °C. With **1a** as precursor of the NHC-bound acyl azolium intermediate, the corresponding ketone product (**3a**) could be detected but with very low yield (entry 1). Switching **1a** to acyl chloride (**1b**) under otherwise identical conditions led to slightly improved while still very low reaction yield (<5% yield, entry 2). When acyl imidazole (**1c**) was used, the ketone product (**3a**) could be obtained in an appreciable 22% yield (entry 3). We then moved to search for better NHC catalysts (entries 4–6) and found that with the use of triazolium **B** could give **3a** in 42% yield (entry 4). The *N*-substituents on the triazolium pre-catalyst had a clear influence on the reaction efficiency, as replacing the *N*-mesityl substituent of **B** with a *N*-2,6-methoxyl (**C**) or *N*-2,6-chloro (**D**) substituents led to much-dropped yields (entries 5–6). With **B** as an NHC pre-catalyst, we then evaluated several metal and organic photocatalysts (entries 7-11, for details see Supplementary Table 3). Electron-deficient iridium complex (**PC-3**) turned to perform the best, giving **3a** in 78% isolated yield (entry 8). It’s worth to note that organic photocatalyst 4CzIPN can give comparable result (entry 11). The screen of solvent didn’t give improved yield (for details see Supplementary Table 1). Notably, the light source has clear influence on the reaction outcomes (for details see Supplementary Table 2). For example, when blue light LEDs (λ_max_ = 440 and 467 nm) were used under otherwise identical conditions, target **3a** were obtained in slightly dropped yields (76 and 62%, respectively). At last, control experiments were conducted. No ketone product could be detected when the reaction was performed in dark or in the absence of photocatalyst (entries 12 and 13). Little ketone product was observed when NHC pre-catalyst was not used (entry 14).

**Table 1:** Optimization of the reaction conditions^*a*^.


Entry	Conditions	Yield [%]^*b*^
1	**PC-1**, **A**, **1a**	Trace
2	**PC-1**, **A**, **1b**	<5
3	**PC-1**, **A**, **1c**	22
4	As entry 3, NHC = **B**	42
5	As entry 3, NHC = **C**	14
6	As entry 3, NHC = **D**	<5
7	**PC-2**, **B**, **1c**	74
8	As entry 7, **PC** = **PC-3**	82 (78)^*c*^
9	As entry 7, **PC** = **PC-4**	76
10	As entry 7, **PC** = **PC-5**	8
11	As entry 7, **PC** = **PC-6**	78
12	As entry 8, without light irradiation	0
13	As entry 8, without **PC**	0
14	As entry 8, without NHC	<5

^*a*^Reaction conditions: **1** (0.15 mmol), **2a** (0.10 mmol), **PC** (1 mol %), NHC (20 mol %), Cs_2_CO_3_ (2.0 equiv), MeCN (2.0 mL), blue LED (Kessil PR160 series, λ_max_ = 427 nm), Ar atmosphere, 30–40 °C, 24 h. ^*b*^NMR yields of reaction mixtures using 1,1,2,2-tetrachloroethane as internal standard. ^*c*^Isolated yield is shown in parentheses.

### Development of one-pot operation

**Fig. 2:**
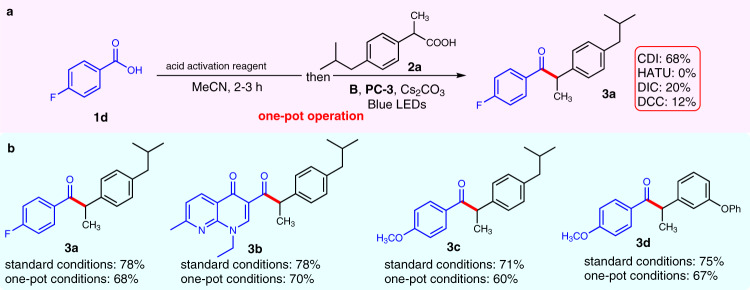
Development of one-pot operation. **a** One-pot procedure for coupling of two carboxylic acids. **b** Comparision between one-pot operation and standard conditions. For synthesis of **3b**, DMSO was used as solvent; HATU: 2-(7-Azabenzotriazol-1-yl)-*N*,*N*,*N’*,*N’*-tetramethyluroniumhexafluorophosphate; DCC: *N*,*N’*-Dicyclohexylcarbodiimide; DIC: *N*,*N’*-Diisopropylcarbodiimide.

We next moved to identify conditions for coupling of two carboxylic acids instead of using pre-prepared acyl imidazole (**1c**) as precursor of the NHC-bound acyl azolium intermediate (Fig. 2a). To our delight, we found that when carboxylic acid **1d** and the carboxylic acid activation reagent CDI were mixed and stirred for 2–3 h followed by the addition of the other reagents (as those used in the optimal condition in Table 1, entry 8), **3a** could be afforded in 68% yield. Several other acid activation reagents (HATU, DIC, and DCC) evaluated here did not give satisfactory results under the standard condition. It turned out the one-pot operation approach worked well for different substrates including marketed drugs, such as nalidixic acid and fenoprofen, giving the corresponding ketone products (**3b**-**3d**) with yields that were only slightly lower than those by using pre-prepared acyl imidazole substrates (Fig. 2b). This formally decarboxylative coupling of two carboxylic acids exhibits attractive applications in late-stage functionalization of marketed drugs and coupling of two drug molecules (Vide infra, Fig. 5).

### Substrate scope

**Fig. 3:**
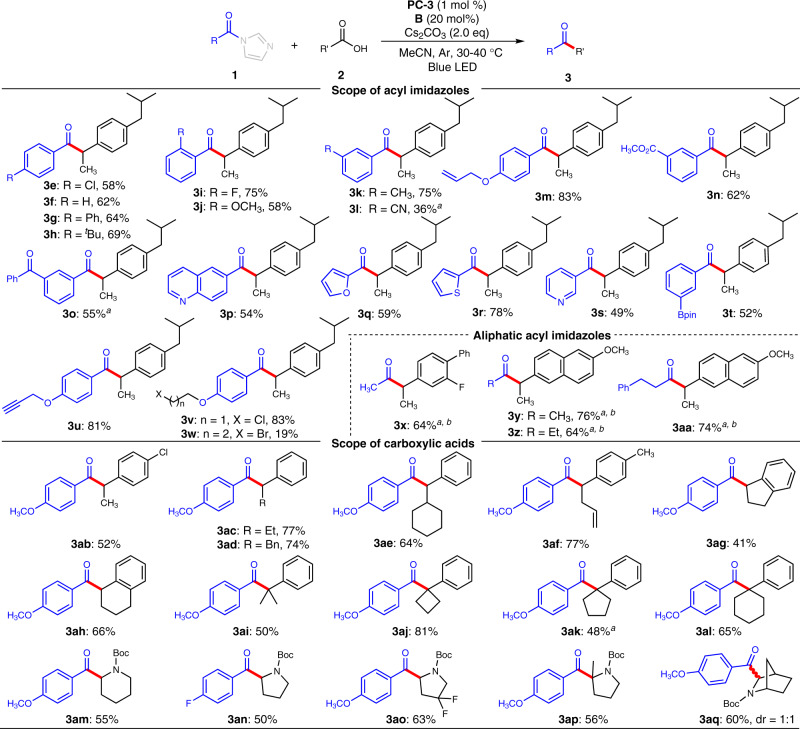
Scope of the reaction. Reaction conditions: **1** (1.5 equiv), **2** (0.1–0.2 mmol), **B** (20 mol %), **PC-3** (1 mol %) and Cs_2_CO_3_ (2.0 equiv) in MeCN (2.0 mL), blue LED (Kessil PR160 series, λ_max_ = 427 nm), Ar atmosphere, 30–40 °C, 24 h. ^*a*^Blue LEDs (Kessil PR160 series, λ_max_ = 440 nm) were used. ^*b*^1.2 equiv Cs_2_CO_3_ was used.

With optimized reaction condition in hands, we then moved to evaluate the scope of our reaction (Fig. 3). To have a precise estimation on the reaction efficiency of this NHC and photocatalyst-catalyzed coupling process, we chose to use pre-prepared acyl imidazoles as precursors of the acyl azolium intermediates. As a technical note, the one-pot operation is recommended for practical synthetic applications, albeit with a slight loss on product yields.

We first evaluated the scope of acyl imidazoles (**1**) by using **2a** as model substrate (Fig. 3). Various substituents on *para*-position of aryl ring were tolerated to give the corresponding coupling ketone products with good to high yields, regardless of their electronic nature (**3e**-**3h**). For example, electron withdrawing groups such as fluorine and chlorine atom substituted acyl imidazoles gave **3a** (Fig. 2b) and **3e** in 78 and 58% yields respectively. Electron donating groups such as methoxyl and tertiary butyl substituted substrates gave desired **3c** (Fig. 2b) and **3h** in 71% and 69% yields. The position of substituents on aryl ring has little effect on the reaction outcomes. *Meta*- and *ortho*-substituted acyl imidazoles were all converted to the target ketone products with good yields (**3i**-**3k**). Cyano group was also tolerated, albeit with lower yield (**3l**). Acyl imidazole containing radical-sensitive C–C double bond was excellent substrate, leading to **3m** in 83% yield. Notably, the ester and ketone moieties, which are typically incompatible in traditional methods for ketone synthesis such as Grignard reactions, can be tolerated in this mild coupling reactions (**3n** and **3o**). Acyl imidazoles bearing heteroaryls such as quinoline (**3p**), furan (**3q**), thiophene (**3r**), and pyridine (**3s**) proceeded smoothly in this reaction to give the target ketone products in moderate to good yields. Potentially reactive functional groups such as boronic ester (**3t**), terminal alkynyl (**3u**), and alkyl chloride (**3v**) were all tolerated. To test the feasibility of our method for the synthesis of dialkyl ketones, a variety of aliphatic acyl imidazoles were investigated. Our method can be used to prepare methyl ketones by using acetic acid-derived acyl imidazole as an acetyl source. For example, flurbiprofen and naproxen underwent decarboxylative acetylation smoothly to afford methyl ketone **3x** and **3y** in 64% and 76% yield, respectively. Other aliphatic acyl imidazoles also proceed smoothly to afford the dialkyl ketones in high yields (**3z**-**3aa**). As a technical note, when aliphatic acyl imidazole substrates were used, a lower loading of Cs_2_CO_3_ and a longer wavelength were optimal in order to avoid side reactions likely caused by base-mediated α-carbon deprotonation (of the acyl imidazole substrates)^73,74^ and high energy irradiation.

We next examined the scope of the carboxylic acids by using *para*-methoxyl substituted acyl imidazole **1e** as a model precursor of NHC-bound acyl azolium intermediate. Chlorine atom on aryl ring was tolerated to give 52% yield of target ketone (**3ab**). The methyl group at α position of carboxylic acids (**2**) could be replaced with ethyl (**3ac**), benzyl (**3ad**), cyclohexyl (**3ae**), and allyl (**3af**) groups without affecting the reaction outcomes dramatically. Cyclic alkyl units such as cyclopentyl (**3ag**) and cyclohexyl (**3ah**) could also be incorporated into the ketone product, albeit with a slight decrease in yield for the former one (**3ag**). Besides, the α-tertiary alkyl substituted carboxylic acids were also competent in this coupling reaction, which thus render this method to be an efficient tool to access sterically hindered ketones with an all-carbon quaternary center at α-position (**3ai**-**3al**, **3ap**). Notably, these congested ketone products are generally challenging to synthesis^76–79^. To our delight, *N*-protected cyclic amino acids were excellent substrates for this coupling reaction, thus offering a straightforward method to access valuable α-amino ketones from readily available and abundant materials. For example, *N*-protected piperidine-2-carboxylic acid, proline, and their derivatives such as 4-fluornated proline, 2-methylproline, and bridged-ring containing piperidine-2-carboxylicacid were all competent substrates to give the corresponding α-amino ketones in moderate yields (**3am**-**3aq**).

### Three-component radical relay reactions

Multi-component radical relay reactions provide a powerful tool for the synthesis of complex skeletons from simple and readily accessible starting materials^80–82^. While significant progress has been achieved in NHC and photocatalyst co-catalyzed ketone synthesis^57–64,83^, three-component radical relay coupling that allows various carbon radicals involved is still limited^72^.

**Fig. 4:**
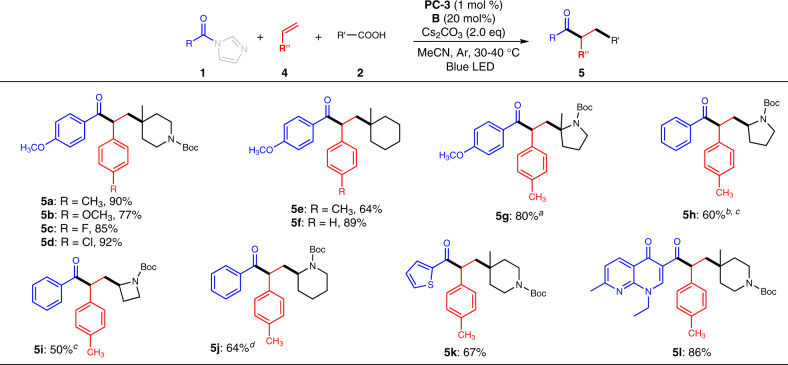
Examples of three-component radical relay reactions. Reaction conditions: **1** (2.0 equiv), **2** (0.1–0.3 mmol, 1.0 equiv), **4** (3.0 equiv), **B** (20 mol%), **PC-3** (1 mol %) and Cs_2_CO_3_ (2.0 equiv) in MeCN, blue LED (Kessil PR160 series, λ_max_ = 427 nm), Ar atmosphere, 30–40 °C, 24 h. ^*a*^dr = 1.6/1. ^*b*^The reaction was set up in 2 mmol scale. ^*c*^dr = 1.4/1. ^*d*^dr = 1.9/1.

To test the feasibility of our coupling reaction in muti-component radical relay reactions, alkenes were incorporated into the system as third coupling partners. We began the investigation with *N*-protected-4-methyl-4-carboxylic acid (**2b**), *para*-methoxyl substituted acyl imidazole **1**, and 4-methyl styrene (**4a**) as model substrates (Fig. 4). To our delight, the three-component coupling ketone product (**5a**) was obtained exclusively in up to 90% yield with complete regioselectivity under our optimal conditions. The exceptional regioselectivity comes from the preference in forming aryl ring stabilized benzyl radical. Further investigation revealed that various styrenes bearing different functional groups such as methoxyl, fluorine, and chlorine atoms are tolerated to give desired ketones in high yields, regardless of their electronic nature (**5b**-**5d**). Other radical precursors (including amino acids) and acyl imidazoles (including hetero cyclic ones) were also competent for this three-component radical relay coupling (**5e**-**5l**). For example, tertiary and secondary radicals generated from cyclic amino acids were incorporated into a series of γ-amino ketones (**5g**-**5j**) in high yields. Marketed drugs such as nalidixic acid was transformed into ketone product (**5l**) with high level of complexity. These three-component radical relay coupling reaction further demonstrated the flexibility and utility of our NHC and photocatalyst co-catalyzed coupling reactions.

### Synthetic applications in late-stage functionalization of marked drugs

**Fig. 5:**
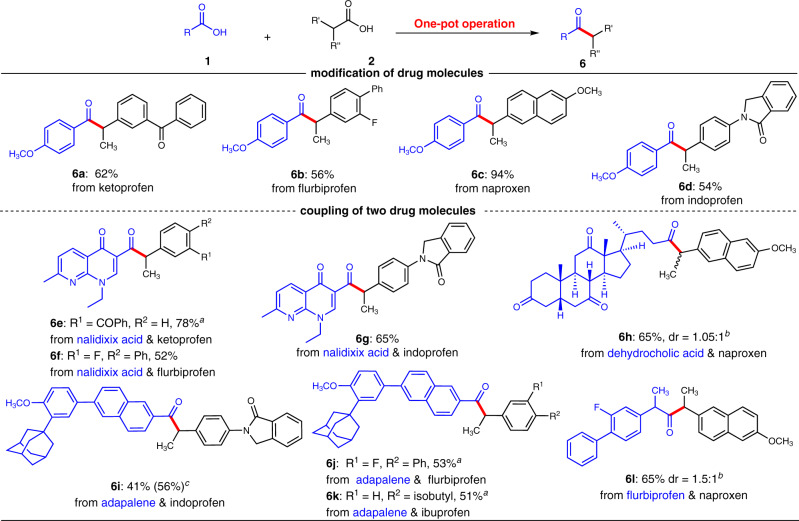
Application of the method in modification of drug molecules. Reaction conditions: **1** (1.5 equiv), **2** (0.05-0.1 mmol, 1.0 equiv), **B** (20 mol%), **PC-3** (1 mol %) and Cs_2_CO_3_ (2.0 equiv) in MeCN, blue LED (Kessil PR160 series, λ_max_ = 427 nm), Ar atmosphere, 30–40 °C, 24 h. ^*a*^ Pre-prepared acyl imidazoles were used. ^*b*^ Blue LEDs (Kessil PR160 series, λ_max_ = 440 nm) were used. ^*c*^ Yield in parentheses is obtained by using pre-prepared acyl imidazole as starting material.

To test the synthetic potential of this mild coupling reaction, modification of marketed drug molecules was explored using the one-pot operation approach (Fig. 5). For example, α-aryl propionic acids (such as ketoprofen, flurbiprofen, naproxen, and indoprofen), which are widely used as nonsteroidal anti-inflammatory drugs, were smoothly transformed into the corresponding ketone products (**6a**-**6d**) in moderate to high yields under our one-pot operation approach. Besides, our method also allows direct decarboxylative coupling of two drug molecules that tethered to carboxylic acid group, affording ketone entities bearing two drug fragments. For example, nalidixic acid, a synthetic quinolone antibiotic, was coupled with flurbiprofen, and indoprofen smoothly to deliver corresponding new ketone entities (**6f**-**6g**) in good yields. In case of the coupling of nalidixic acid and ketoprofen, while the one-pot coupling gave poor yield, the target ketone product (**6e**) was obtained in 78% yield when pre-prepared acyl imidazole was used as starting material. Aliphatic carboxylic acids could also undergo the one-pot coupling to construct dialkyl ketones. For example, dehydrocholic acid and flurbiprofen were coupled with naproxen smoothly under the one-pot condition, giving the dialkyl ketones (**6h, 6l**) in 65% yields. Our method was also applied in the modification of adapalene, a third-generation topical retinoid. Nevertheless, the one-pot coupling of adapalene and indoprofen only gave 41% yield of desired ketone product (**6i**). To our delight, the using of pre-prepared acyl imidazole could give moderate yield (56%). Under the same condition, adapalene coupled with ibuprofen, and flurbiprofen successfully to give the target coupling products (**6j**-**6k**) in moderate yields. These results revealed that our method exhibits remarkable potential application in rapid assembly of complex molecules.

### Mechanistic studies

**Fig. 6:**
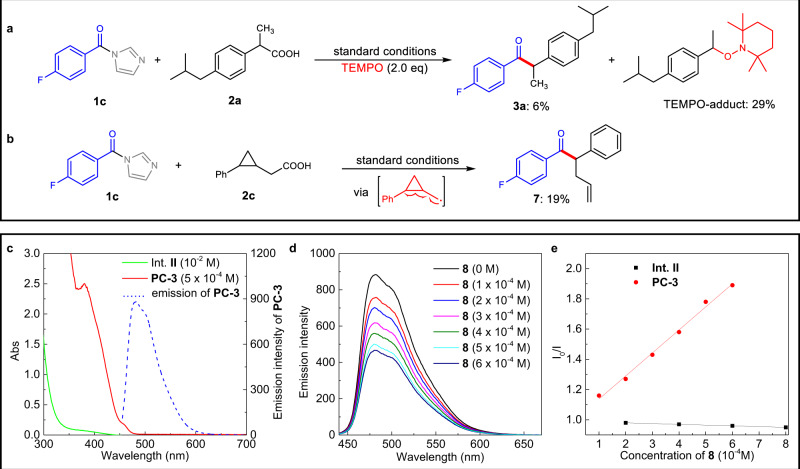
Mechanistic studies. **a** Reaction in the presence of TEMPO (2,2,6,6-Tetramethylpiperidinooxy). **b** Radical clock reaction. **c** UV–Vis absorption spectra of Int. **II** and **PC-3**. **d** Stern–Volmer quenching experiments between **PC-3** and sodium carboxylate **8**. **e** Stern–Volmer quenching experiments between **Int. II** and sodium carboxylate **8** (black line).

To gain insight into the mechanism, mechanistic studies were conducted next (Fig. 6). The model reaction was suppressed with 6% yield of the target product was observed in the presence of TEMPO (2,2,6,6-Tetramethylpiperidinooxy) under otherwise identical conditions. Meanwhile, TEMPO-adduct was isolated in 29% yield, suggesting a radical pathway (Fig. 6a). This was further supported by a radical clock reaction, in which a cyclopropyl group was installed in the carboxylic acid (Fig. 6b). The observation of the cyclopropane ring-opening product (**7**) strongly supports the presence of α-cyclopropyl carbon radical that generated from oxidative decarboxylative of carboxylic acid (**2c**) (Fig. 6b). It is well known that excited state of photocatalyst can promote the oxidative decarboxylation of carboxylic acids^84,85^. Meanwhile, the acyl azolium intermediate **II** (Int. **II**) were proved to be excited under visible light irradiation^64,86^ and further act as strong oxidant^64^, which may also contribute to the decarboxylation process. Thus, photophysical behaviors of pre-prepared Int. **II** and **PC-3** were investigated to identify these two possibilities. We firstly investigated the UV–Vis absorption spectra of Int. **II** and photocatalyst (**PC-3**) under the above reaction concentration (Fig. 6c). While Int. **II** can absorb visible light until around 430 nm (Fig. 6c, green line), **PC-3** shows much more strong absorption (Fig. 6c, red line). Besides, Stern–Volmer quenching experiments of Int. **II** and **PC-3** were conducted by using sodium carboxylate (**8**) as a quenching reagent. The results revealed that sodium carboxylate **8** could not quench the emission of Int. **II** (Fig. 6e, black line), suggesting that the Int. **II** was not responsible for the oxidative decarboxylation of carboxylic acid. In contrast, the emission of **PC-3** (Fig. 6c, blue dashed line) could be effectively quenched by sodium carboxylate **8** (Fig. 6d and the red line in Fig. 6e) with the quenching rate constant (*k*_q_) calculated by using the reported lifetime (2280 ns) of **PC-3** as 6.68 × 10^8^ M^−1^s^−1^ ^87^. This result is consistent with the crucial role of **PC-3** in this decarboxylative coupling reaction (Table 1, entry 13), supporting the photocatalyst-initiated oxidative decarboxylation of carboxylic acid.

**Fig. 7:**
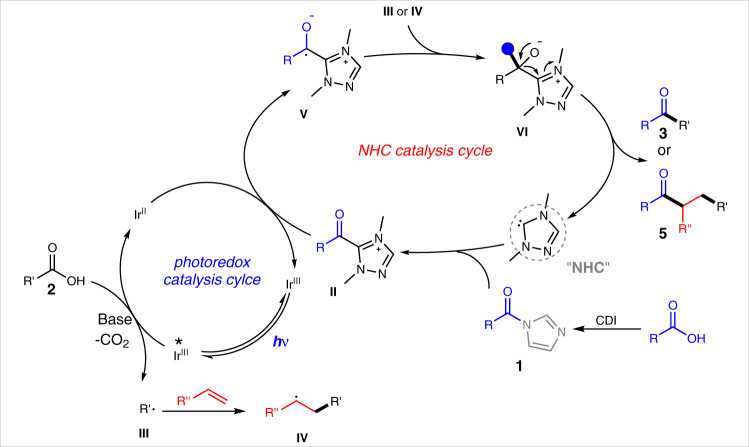
Proposed catalytic cycle. NHC and photocatalyst co-catalyzed mechanism.

Upon the above mechanistic studies and previous reports^57–64,88,89^, a catalytic cycle involving NHC and photo catalysis was proposed (Fig. 7). The acyl imidazoles (**1**) can be pre-prepared or in situ formed via the reaction of a carboxylic acid with CDI (Carbonyldiimidazole) as the condensation reagent. The resulting or pre-prepared acyl imidazole **1** can subsequently react with NHC catalyst to form an acyl azolium intermediate **II**. In the same solution, an iridium complex photocatalyst is excited by visible light to mediate oxidative decarboxylation of another carboxylic acid (**2**) to form a transient radical intermediate **III**^84,85,90^. The reduced iridium species from the above photo process then reduces the acyl azolium intermediate (**II**) to the corresponding persistent NHC-bound radical intermediate **V** with regeneration of the original iridium photocatalyst^57–63^. Coupling of the two radical intermediates (**III** and **V**) eventually affords the ketone product **3** with regeneration of the NHC catalyst. In case of the three-component radical relay coupling, the carbonyl radical (**III**) generated from decarboxylation is trapped by the alkene to form another carbon radical (**IV**) which subsequently couples with **V** to deliver the target ketone **5**.

## Discussion

In conclusion, we have developed a convenient approach for coupling of carboxylic acids and acyl imidazoles by merging NHC and photo catalysis. The acyl imidazoles can be generated in situ via the reaction of another carboxylic acid with CDI, thus providing a straightforward strategy for the synthesis of ketone moieties from two carboxylic acids. The mild condition, operational simplicity, and use of readily available carboxylic acid as both acyl source and radical precursors make our method much more convenient (than previous approaches) for broad applications. Notably, the coupling process can also be intercepted by the addition of an alkene as the third coupling partner, leading to a three-component radical reaction with the formation of sophisticated products from simple starting materials. Natural products and medicinal molecules and their fragments can be directly coupled using our methods. Ongoing studies in our laboratories include the preparation and study of drug conjugates and derivatives, and concise synthesis of complex molecules by applying and further developing our carboxylic acid radical coupling reactions.

## Methods

### General procedure for the decarboxylative coupling of carboxylic acids and acyl imidazoles

To a 10 mL Schlenk tube equipped with a stir bar was added acyl imidazole **1** (0.15 mmol), carboxylic acid **2** (0.10 mmol), NHC pre-catalyst **B** (0.02 mmol), photocatalyst **PC-3** (0.001 mmol) and dry Cs_2_CO_3_ (0.20 mmol). The Schlenk tube was sealed and placed under argon before 2 mL of dry MeCN was added. The reaction was stirred and irradiated with one/two blue LED Kessil lamp (λ_max_ = 427 nm, 3 cm away from the Schlenk tube, with cooling fan to keep the reaction temperature at 30–40 °C.) for 24 h. Then the reaction mixture was filtered through a pad of celite and washed with ethyl acetate. The filtrate was concentrated in vacuum to afford the crude material which was purified by column chromatography (silica gel, EtOAc/hexanes) to give product **3**.

### General procedure for the one-pot decarboxylative coupling of two carboxylic acids

In gloves box, to a 4 mL vial equipped with a stir bar was added 4-flurobenzoic acid **1d** (0.2 mmol) and CDI (0.2 mmol). MeCN (2 mL) was added as solvent. The reaction mixture was stirred for 2.5 h in gloves box at room temperature until the solution become homogenous. The resulting reaction mixture was mixed with carboxylic acid **2a** (0.10 mmol), NHC pre-catalyst **B** (0.02 mmol), photocatalyst **PC-3** (0.001 mmol) and dry Cs_2_CO_3_ (0.20 mmol). The resulting mixture was sealed and take out from the gloves box. Then the reaction was stirred and irradiated with one blue LED Kessil lamp (λ_max_ = 427 nm, 3 cm away from the Schlenk tube, with cooling fan to keep the reaction temperature at 30–40 °C.) for 12–24 h. The reaction mixture was filtered through a pad of celite and washed with ethyl acetate. The filtrate was concentrated in vacuum to afford the crude material which was purified by column chromatography (silica gel, EtOAc/hexanes) to give product **3a**.

## Supplementary information

Supplementary Information

## Data Availability

All data generated in this study are provided in the Supplementary Information/Source Data file, and can be obtained from the authors upon request.
