# Supplementary material for: Carbene and photocatalyst-catalyzed decarboxylative radical coupling of carboxylic acids and acyl imidazoles to form ketones
Source: Nat Commun. 2022 May 23;13:2846. doi: 10.1038/s41467-022-30583-2 (PMC9126905; doi:10.1038/s41467-022-30583-2)
Supplement: Supplementary file 1 — Supplementary Information [file 41467_2022_30583_MOESM1_ESM.pdf]

Supplementary Information

Carbene and Photocatalyst-Catalyzed Decarboxylative Radical Coupling of Carboxylic Acids and Acyl Imidazoles to Form Ketones

Shi-Chao Ren[1,3], Xing Yang[3], Bivas Mondal[3], Chengli Mou[2], Weiyi Tian[2,*], Zhichao Jin[1], Yonggui Robin Chi[1,3,*]

[1]Laboratory Breeding Base of Green Pesticide and Agricultural Bioengineering, Key Laboratory of Green Pesticide and Agricultural Bioengineering, Ministry of Education, Guizhou University, Huaxi District, Guiyang 550025, China.

[2]Guizhou University of Traditional Chinese Medicine, Guiyang 550025, China.

[3]Division of Chemistry & Biological Chemistry, School of Physical & Mathematical Sciences, Nanyang Technological University, Singapore 637371, Singapore.

Email: robinchi@ntu.edu.sg, tianweiyi@gzy.edu.cn

Table of Contents

1 **Supplementary Notes** S3

2 **Supplementary Discussion** S4

2.1 Condition optimizations S4

2.2 Mechanistic studies S6

3 **Supplementary Methods** S8

3.1 General procedure for coupling of carboxylic acids and acyl imidazoles S8

3.2 General procedure for formally coupling of two carboxylic acids S9

3.3 General procedure for three-component radical relay coupling S10

3.4 General procedure for preparation of starting materials S11

3.5 Attempt for enantioselective ketone synthesis S12

3.6 Characterizations of new compounds S14

Appendix: ^{1}H and ^{13}C NMR spectra for new compounds S48

Supplementary references S151

1. Supplementary Notes

All reactions and manipulations involving air-sensitive compounds were carried out using standard Schlenk techniques. Anhydrous toluene, hexane, Et_2O and THF were distilled from sodium benzophenone ketyl. Anhydrous CH_2Cl_2 and $CHCl_3$ were distilled from CaH_2 under an atmosphere of nitrogen. Anhydrous MeCN were purchased from Sigma-Aldrich Co., Inc. All reactions were monitored by TLC. TLC analysis was performed by illumination with a UV lamp (254 nm). All flash chromatography was packed with silica-gel as the stationary phase. ^1H NMR spectra were recorded on a Bruker Avance (500 MHz) or Bruker BBFO (400 MHz) instrument, and chemical shifts were reported in ppm downfield from internal TMS with the solvent resonance as the internal standard ($CDCl_3$, δ = 7.26 ppm). ^{13}C NMR spectra were recorded on a Bruker BBFO (100 MHz) or Bruker Avance (125 MHz) instrument, and chemical shifts were reported in ppm downfield from TMS with the solvent resonance as the internal standard ($CDCl_3$, δ = 77.0 ppm). ^{19}F NMR spectra were recorded on a Bruker BBFO (376 MHz) instrument. High resolution mass spectra (HRMS) (EI^+) were recorded on an a Finnigan MAT 95 XP mass spectrometer. Melting points are uncorrected and were recorded on an MPA 100 OptiMelt Automated Melting Point System. Flash column chromatography was performed using Merck silica gel 60 with distilled solvents. Commercially available reagents were purchased from Energy Chemical, J& K Scientific, Adamas-beta and Sigma-Aldrich Co., Inc.

2 Supplementary Discussion

2.1 Condition optimizations

Supplementary Table 1. Influence of solvent[a]

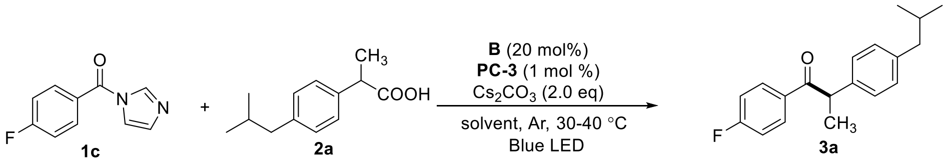

entry	solvent	yield/ %
1	MeCN	82 (78)[b]
2	THF	52
3	DCE	46
4	DMSO	42
5	DCE/H_2O (v:v = 1:1)	15

[a]Reaction conditions: **1c** (0.15 mmol), **2a** (0.1 mmol), **B** (20 mol %), **PC-3** (1 mol %) and Cs_2CO_3 (2.0 equiv) in solvent (2.0 mL), blue LED (Kessil PR160 series, λ_{max}= 427 nm), Ar atmosphere, 30-40 °C, 12 h. [b]Isolated yield.

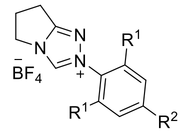

B: $R^1 = R^2 = CH_3$

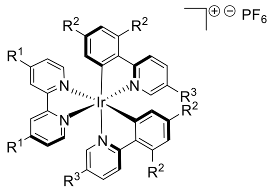

PC-3: R^1 = H, R^2 = F, $R^3 = CF_3$

Supplementary Table 2. Influence of light source[a]

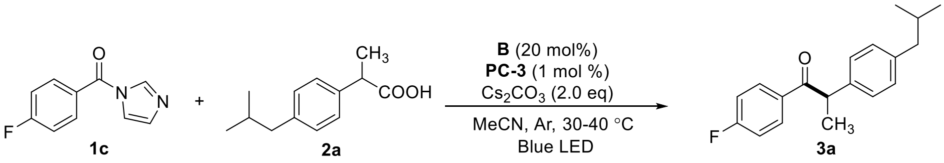

entry	light source	yield/ %
1	Blue LED (λ_{max} = 427 nm)	82 (78)[b]
2	Blue LED (λ_{max} = 440 nm)	76
3	Blue LED (λ_{max} = 467 nm)	62
4	23 W CFL	36

[a]Reaction conditions: **1c** (0.15 mmol), **2** (0.10 mmol), **B** (20 mol %) **PC-3** (1 mol %) and Cs_2CO_3 (2.0 equiv) in MeCN (2.0 mL), light source, Ar atmosphere, 30-40 °C, 12 h. [b]Isolated yield.

Supplementary Table 3. Screening of organic photocatalyst.[a]

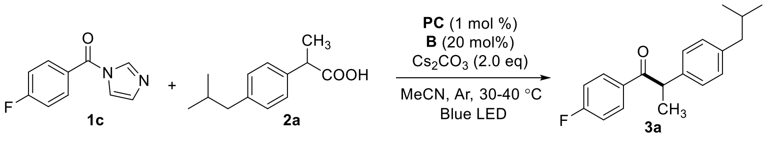

entry	**PC**	light souce	yield/ %
1	**PC-3** (1 mol%)	427 nm	82 (78)[b]
2	4CzIPN (2 mol%)	427 nm	64
3	4CzIPN (3 mol%)	427 nm	72
4	4CzIPN (5 mol%)	427 nm	78
5	Ph-Acr-Me^+ (5 mol%)	427 nm	4
6	eosin Y (3 mol%)	427 nm	0
7	Rose Bengal (3 mol%)	23 W CFL	0
8	10-phenylphenothiazine (3 mol%)	427 nm	0

[a]Reaction conditions: **1c** (0.15 mmol), **2a** (0.1 mmol), **B** (20 mol %), **PC**, and Cs_2CO_3 (2.0 equiv) in solvent (2.0 mL), light souce, Ar atmosphere, 30-40 °C, 12 h. [b]Isolated yield.

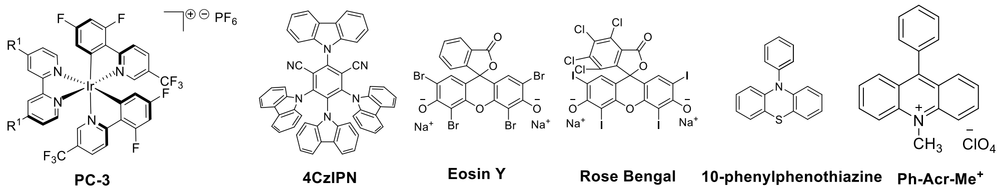

2.2 Mechanistic studies

2.2.1 UV-Vis absorption spectrum and emission spectra

The UV-Vis absorption spectra of pre-formed acyl azolium intermediate **II** (Int. **II**) and **PC-3** were measured on a SHIMADZU UV-3600 UV/Vis spectrometer under reaction concentration in anhydrous DMSO. The emission spectrum of **PC-3** (10^{-5} M in DMSO) upon excitation at 420 nm was recorded (Supplementary Figure 1, blue dashed line).

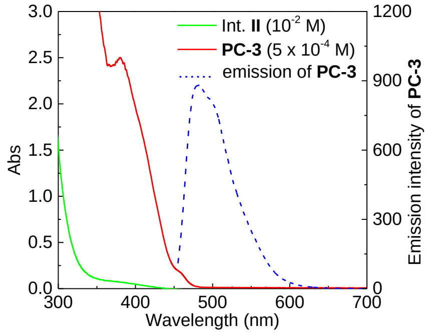

Supplementary Figure 1. UV-Vis absorption spectra and emission spectra. UV-Vis absorption spectra of **Int. II** and **PC-3**. Emission spectrum of **PC-3**.

2.2.2 Stern-Volmer quenching experiments of Int. II

The emission spectrum of acyl azolium intermediate **II** (Int. **II**, 10^{-3} M in DMSO) upon excitation at 420 nm was recorded (Figure S2, black line). Stern-Volmer quenching experiment between Int. **II** and sodium 2-(4-isobutylphenyl)propanoate (**8**) were conducted (Supplementary Figure 2). The results revealed that sodium 2-(4-isobutylphenyl)propanoate (**8**) can not quench the excited Int. **II**. This suggests the excitation of acyl azolium intermediate **II** do not responsible to the oxidative decarboxylation process.

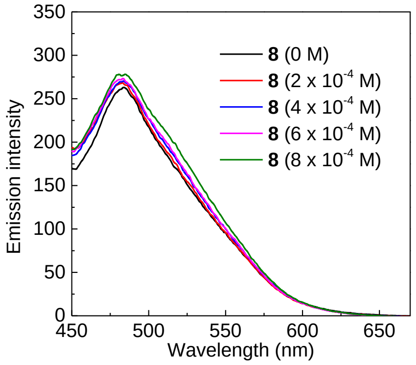

Supplementary Figure 2. **Stern-Volmer quenching experiments**. Quenching of Int. **II** with **8**.

Experimental protocol: The emission spectra of freshly prepared solution of Int. **II** (1 x 10^{-3} M in DMSO, 0.5 mL) was recorded. Then, a solution of sodium 2-(4-isobutylphenyl)propanoate (**8**) in DMSO (2 x 10^{-2} M, 5 μL) was added to the solution, and another emission spectra was recorded. The addition of **8** and the recordation were repeated 4 consecutive times.

2.2.3 Stern-Volmer quenching experiments of PC-3

To support the proposed reductive quenching of photocatalyst (**PC-3**) by carboxylic acid anion, we conducted Stern-Volmer quenching experiments by using sodium 2-(4-isobutylphenyl)propanoate (**8**) as quenching agent (Supplementary Figure 3). It was found that sodium 2-(4-isobutylphenyl)propanoate (**8**) could effectively quench the emission of **PC-3**. The quenching rate constant ($k_q = 6.68 \times 10^8\ M^{-1}s^{-1}$) was calculated by using the reported[1] lifetime of **PC-3** (2280 ns).

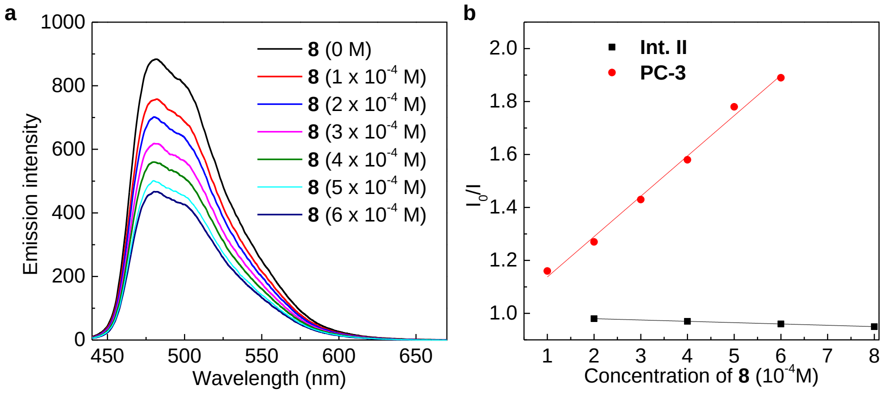

Supplementary Figure 3. Stern-Volmer quenching experiments. **a**. Spectra of Stern-Volmer quenching experiments. **b**. Fit line of I_0/I at 482 nm.

Experimental protocol: The emission spectra of a solution of **PC-3** (10^{-5} M in DMSO, 0.5 mL) was recorded. Then, a solution of sodium 2-(4-isobutylphenyl)propanoate (**8**) in DMSO (1 x 10^{-2} M, 5 μL) was added to the solution, and another emission spectra was recorded. The addition of **8** and the recordation were repeated 5 consecutive times.

3. Supplementary Methods

3.1 General procedure for coupling of carboxylic acids and acyl imidazoles

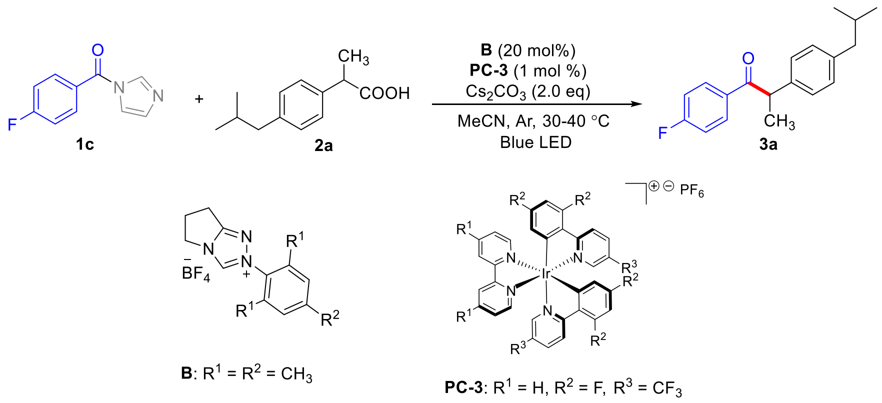

General procedure A: To a 10 mL Schlenk tube equipped with a stir bar was added (4-fluorophenyl)(1H-imidazol-1-yl)methanone **1c** (28.5 mg, 0.15 mmol), 2-(4-isobutylphenyl)propanoic acid **2a** (20.6 mg, 0.10 mmol), NHC pre-catalyst **B** (6.3 mg, 0.02 mmol), photocatalyst **PC-3** (1.0 mg, 0.001 mmol) and dry Cs_2CO_3 (65.0 mg, 0.20 mmol). The Schlenk tube was sealed and placed under argon before 2 mL of dry MeCN was added. The reaction was stirred and irradiated with one/two blue LED Kessil lamp (λ_{max} = 427 nm, intensity = 100%, 3 cm away from the Schlenk tube, with cooling fan to keep the reaction temperature at 30-40 °C. Reaction set-up see Supplementary Picture 1) for 24 hours. Then the reaction mixture was filtered through a pad of celite and washed with ethyl acetate. The filtrate was concentrated in vacuum to afford the crude material which was purified by column chromatography (silica gel, EtOAc/hexanes) to give product **3a** in 78% isolated yield (22.1 mg) as colorless liquid. **Note**: For reactions which used aliphatic carboxylic acyl imidazoles as substrates, Cs_2CO_3 (1.2 equiv) and two 440 nm blue LEDs were used.

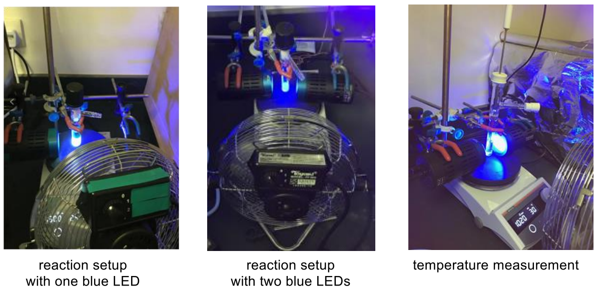

Supplementary Figure 4: **Reaction set-up and temperature measurement**. Reaction set-up with one LED (left) and two LEDs (middle). Temperature measurement (right).

3.2 General procedure for formally coupling of two carboxylic acids

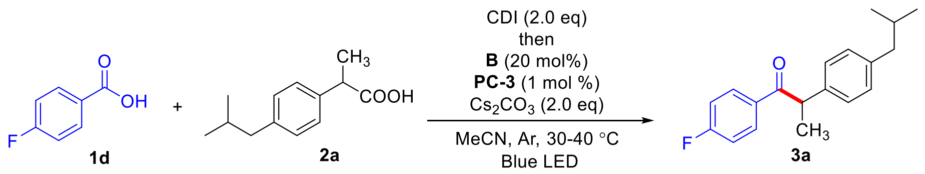

General procedure B: In gloves box, to a 4 mL vial equipped with a stir bar was added 4-flurobenzoic acid **1d** (30.4 mg, 0.2 mmol) and CDI (32.4 mg, 0.2 mmol). MeCN (2 mL) was added as solvent. The reaction mixture was stirred for 2.5 hours in gloves box at room temperature until the solution become homogenous. The resulting reaction mixture was mixed with 2-(4-isobutylphenyl)propanoic acid **2a** (20.6 mg, 0.10 mmol), NHC pre-catalyst **B** (6.3 mg, 0.02 mmol), photocatalyst **PC-3** (1.0 mg, 0.001 mmol) and dry Cs_2CO_3 (65.0 mg, 0.20 mmol). The resulting mixture was sealed and take out from the gloves box. Then the reaction was stirred and irradiated with one blue LED Kessil lamp (λ_{max} = 427 nm, intensity = 100%, 3 cm away from the Schlenk tube, with cooling fan to keep the reaction temperature at 30-40 °C. Reaction set-up see Supplementary Picture 1) for 12-24 hours. The reaction mixture was filtered through a pad of celite and washed with ethyl acetate. The filtrate was concentrated in vacuum to afford the crude material which was purified by column chromatography (silica gel, EtOAc/hexanes) to give product **3a** in 68% isolated yield (19.3 mg) as colorless liquid. **Note**: For nalidixic acid (**3b**, **7g**, **7h**), dehydrocholic acid (**3d**) and adapalene (**7j**) which cannot dissolve in MeCN, DMSO was used as solvent. For synthesis of **3b**, **7g**, **7h**, and **7j**, the corresponding mixtures of acid and CDI were stirred for 10 hours.

3.3 General procedure for three-component radical relay coupling

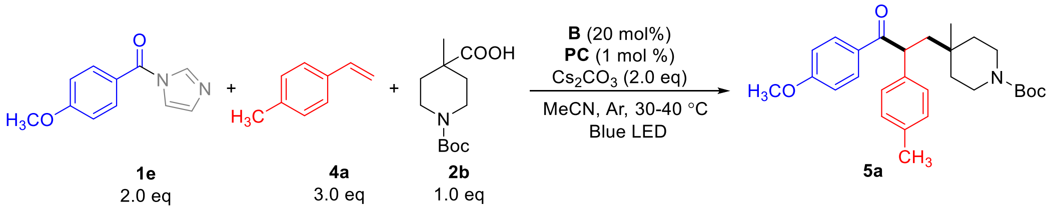

General procedure C: To a 10 mL Schlenk tube equipped with a stir bar was added (1H-imidazol-1-yl)(4-methoxyphenyl)methanone **1e** (40.4 mg, 0.2 mmol),1-methyl-4-vinylbenzene **4a** (35.5 mg, 0.3 mmol) 1-(tert-butoxycarbonyl)-4-methylpiperidine-4-carboxylic acid **2b** (24.3 mg, 0.10 mmol), NHC pre-catalyst **B** (6.3 mg, 0.02 mmol), photocatalyst **PC-3** (1.0 mg, 0.001 mmol) and dryCs_2CO_3 (65.0 mg, 0.20 mmol). The Schlenk tube was sealed and placed under argon before 2-3 mL of dry MeCN was added. The reaction was stirred and irradiated with two blue LED Kessil lamp (λ_{max} = 427 nm, intensity = 75%, 3 cm away from the Schlenk tube, with cooling fan to keep the reaction temperature at 30-40 °C. Reaction set-up see Supplementary Picture 1) for 24 hours. Then the reaction mixture was filtered through a pad of celite and washed with ethyl acetate. The filtrate was concentrated in vacuum to afford the crude material which was purified by column chromatography (silica gel, EtOAc/hexanes) to give product **5a** in 90% yield (40.6 mg) as colorless oil.

3.4 General procedure for preparation of starting materials

All the carboxylic acids are commercially available, and the acyl imidazoles were prepared according to the following procedure.[2]

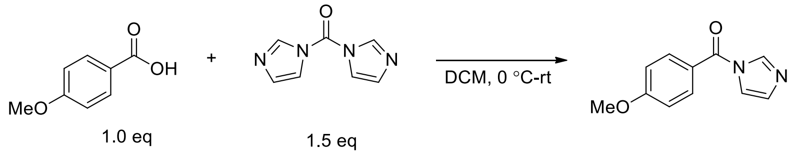

General procedure E: Acyl imidazoles were prepared according to slightly modefied literature procedure[1]. To an oven-dried 100 mL round bottom flask equipped with a stir bar was added 4-methoxybenzoic acid (1.52 g, 10 mmol, 1.0 eq) and dry DCM (30 mL). CDI (2.43g, 15 mmol, 1.5 eq) was added slowly under 0 °C. The reaction mixture was allowed warm to room temperature and stirred over night. Then the reaction mixture was washed vigorously 3 times with water and one time with brine. The organic layer was then dried over $MgSO_4$ and concentration under vacuum. The resulting product (0.98 g) was used directly without further purification.

3.5 Attempt for enantioselective ketone synthesis

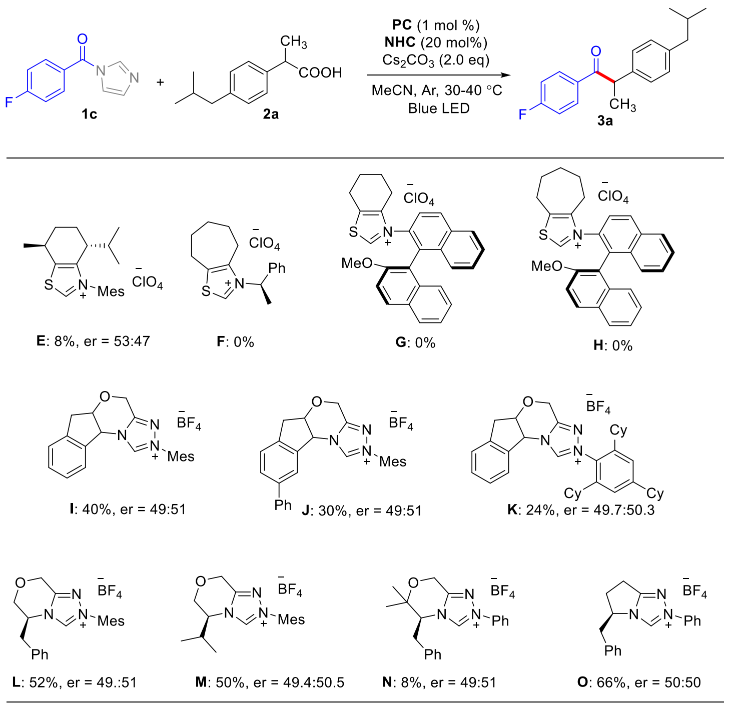

Supplementary Figure 5: **Screening of chiral NHC catalysts**. Reaction conditions: **1c** (0.15 mmol), **2a** (0.1 mmol), **B** (20 mol %), **PC**, and Cs_2CO_3 (2.0 equiv) in MeCN (2.0 mL), light souce, Ar atmosphere, 30-40 °C, 12 h.

When NHC-E was used as catalyst, 53:47 er was determined by HPLC (AD-H, 95:5 hexane/^{i}PrOH, 0.5 mL/min, t_{maj} = 9.07 min, t_{min} = 10.50 min.

<Chromatogram>

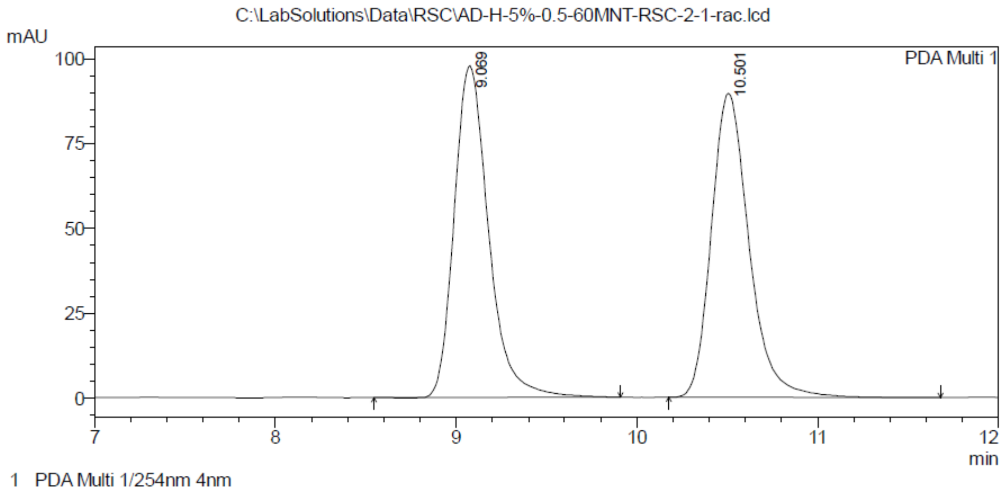

PeakTable

PDA Ch1 254nm 4nm

Peak#	Ret. Time	Area	Height	Area %	Height %
1	9.069	1296754	97894	49.902	52.191
2	10.501	1301825	89676	50.098	47.809
Total		2598580	187570	100.000	100.000

Supplementary Figure 6: **HPLC spectrum of racemic sample**. No ee value was detected.

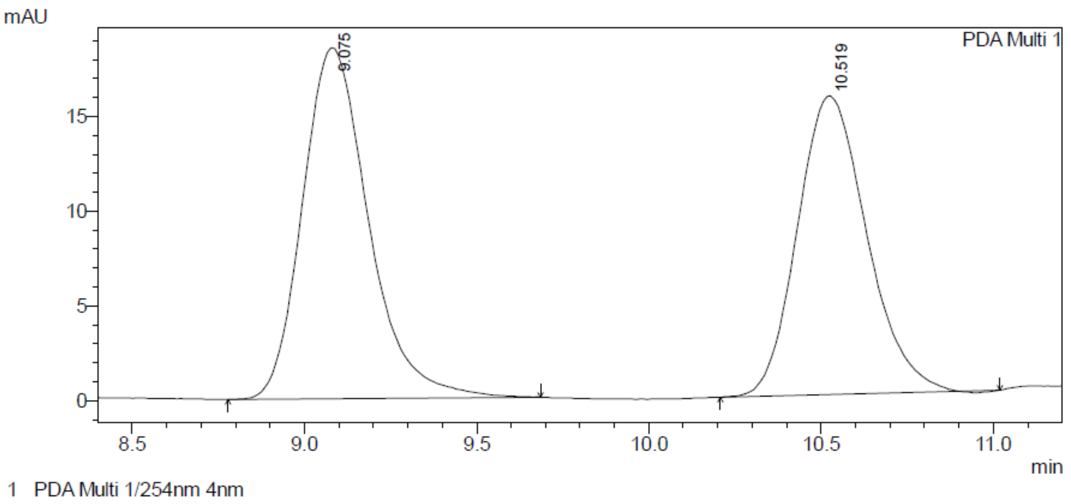

PeakTable

PDA Ch1 254nm 4nm

Peak#	Ret. Time	Area	Height	Area %	Height %
1	9.075	246947	18514	52.877	54.033
2	10.519	220071	15750	47.123	45.967
Total		467019	34264	100.000	100.000

Supplementary Figure 7: **HPLC spectrum of sample from NHC-E**. ee value was detected as 5.7.

3.6 Characterizations of new compounds

1-(4-fluorophenyl)-2-(4-isobutylphenyl)propan-1-one (3a)

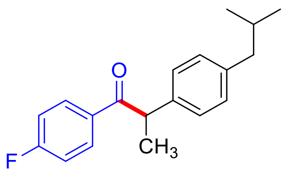

According to the general procedure **A** (0.1 mmol scale), product **3a** was isolated in 78% yield (22.1 mg) as colorless liquid; ^{1}H NMR (400 MHz, $CDCl_3$) δ 8.01-7.94 (1H, m), 7.18-7.13 (2H, m), 7.10-6.99 (4H, m), 4.60 (1H,q, *J* = 6.8 Hz), 2.41 (2H,d, *J* = 7.2 Hz), 1.88-1.74 (1H, m), 1.51 (3H,d, *J* = 6.8 Hz), 0.87 (6H,t, *J* = 6.5 Hz); ^{13}C NMR (100MHz, $CDCl_3$) δ 198.9 (CO), 165.4 (CF, d, *J* = 253.0 Hz), 140.4 (C), 138.5 (C), 132.9 (C, d, *J* = 3.0 Hz), 131.4 (CH, d, *J* = 9.2 Hz), 129.7 (CH), 127.3 (CH), 115.5 (CH, d, *J*= 21.7 Hz), 47.6 (CH), 45.0 (CH_2), 30.1 (CH), 22.35 (CH_3), 22.33 (CH_3), 19.5 (C); ^{19}F NMR (376MHz, $CDCl_3$) δ -105.8 (m); HRMS (ESI): Found: *m/z* 307.1474. Calcd for $C_{19}H_{21}OFNa$ $(M+Na)^+$ 307.1474.

Note: 3a could also be obtained via one-pot operation according to general procedure B in 68% yield.

1-ethyl-3-(2-(4-isobutylphenyl)propanoyl)-7-methyl-1,8-naphthyridin-4(1H)-one (3b)

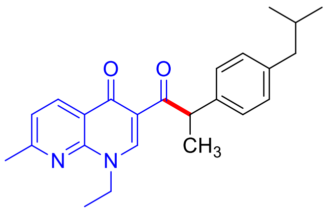

According to the general procedure **A** (0.1 mmol scale), product **3b** was isolated in 78% yield (29.3 mg) as white solid; mp: 140-141 °C; ^{1}H NMR (400 MHz, $CDCl_3$) δ 8.62 (1H, d, *J* = 8.3 Hz), 8.55 (1H, s), 7.30 (2H, d, *J* = 8.0 Hz), 7.22 (1H, d, *J* = 8.1 Hz), 7.07-6.99 (2H, d, *J* = 8.0 Hz), 5.56 (1H, q, *J* =7.2 Hz), 4.51-4.35 (2H, m), 2.63 (3H, s), 2.38 (2H,d, *J* = 7.0 Hz), 1.85-1.74 (1H, m), 1.50 (3H, d, *J* = 6.9 Hz), 1.44 (3H, t, *J* = 7.0 Hz), 0.85 (6H, d, *J* = 6.7 Hz); ^{13}C NMR (100MHz, $CDCl_3$) δ 200.7 (CO), 175.5 (CO), 162.6 (C), 148.6 (C), 148.5 (CH), 139.8 (C), 138.7 (C), 136.8 (CH), 129.0 (CH),

128.2 (CH), 121.9 (C), 121.0 (CH), 118.8 (C), 48.6(CH), 46.6(CH_2), 45.1(CH_2), 30.1 (CH), 25.0(CH_3), 22.4 (CH_3), 18.5 (CH_3), 15.1 (CH_3); HRMS (ESI): Found: *m/z* 399.2039.Calcd for $C_{24}H_{28}N_2O_2Na$ $(M+Na)^+$ 399.2048.

Note: 3b could also be obtained via one-pot operation according to general procedure B in 70% yield.

2-(4-isobutylphenyl)-1-(4-methoxyphenyl)propan-1-one (3c)

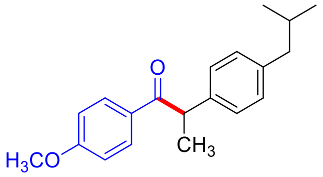

According to the general procedure **A** (0.2 mmol scale), product **3c** was isolated in 71% yield (42.1 mg) as yellow liquid; ^{1}H NMR (400 MHz, $CDCl_3$) δ 7.99-7.92 (2H, m),7.23-7.15 (2H, m), 7.06 (2H, d, *J* = 8.1 Hz), 6.90-6.82 (2H, m), 4.62 (1H, q, *J* = 6.8 Hz), 3.81 (3H, s), 2.41 (2H, d, *J* = 7.3 Hz), 1.88-1.75 (1H, m), 1.51 (3H, d, *J* = 6.9 Hz), 0.87 (6H, d, *J* = 6.7 Hz); ^{13}C NMR (125 MHz, $CDCl_3$) δ 199.1 (CO), 163.1 (C), 140.1 (C), 139.0 (C), 131.0 (CH), 129.6 (C), 129.5 (CH), 127.3 (CH), 113.6 (CH), 55.3 (CH_3), 47.0 (CH), 44.9 (CH_2), 30.1 (CH), 22.35 (CH_3), 22.33 (CH_3), 19.5 (CH_3); HRMS (ESI): Found: *m/z* 297.1853. Calcd for $C_{20}H_{25}O_2$ $(M+H)^+$ 297.1855.

Note: 3c could also be obtained via one-pot operation according to general procedure B in 60% yield.

1-(4-methoxyphenyl)-2-(3-phenoxyphenyl)propan-1-one (3d)

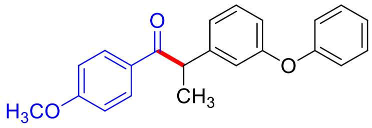

According to the general procedure **B** (0.1 mmol scale), product **3d** was isolated in 75% yield (22.2 mg) as yellow liquid; ^{1}H NMR (400 MHz, $CDCl_3$) δ 7.93 (2H, d, *J* = 8.8 Hz), 7.31 (2H, t, *J* = 7.8 Hz), 7.24 (1H, t, *J* = 8.0 Hz, 1H), 7.09 (1H, t, *J* = 7.3 Hz), 7.04- 6.97 (2H,m), 6.95 (2H, d, *J* = 8.1 Hz), 6.87 (2H, d, *J* = 8.8 Hz), 6.82 (1H, dd, *J* = 8.1, 1.7 Hz), 4.66-4.56 (1H, *J* = 6.9 Hz), 3.83 (3H, s), 1.52 (3H, d, *J* = 6.8 Hz); ^{13}C NMR (100MHz, $CDCl_3$) δ 198.4 (CO), 163.2 (C), 157.5 (C), 157.1 (C), 143.8 (C), 131.0 (CH), 130.1 (CH), 129.7 (CH), 129.3 (C), 123.2 (CH), 122.5 (CH), 118.7 (CH),

118.5 (CH), 117.0 (CH), 113.6 (CH), 55.4 (CH_3), 47.3 (CH), 19.3 (CH_3); HRMS (ESI): Found: *m/z* 355.1318. Calcd for $C_{22}H_{20}O_3Na$ $(M+Na)^+$ 355.1310.

Note: 3d could also be obtained via one-pot operation according to general procedure B in 67% yield.

1-(4-chlorophenyl)-2-(4-isobutylphenyl)propan-1-one (3e)

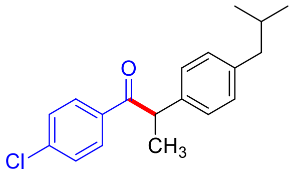

According to the general procedure **A** (0.1 mmol scale), product **3e** was isolated in 58% yield (17.6 mg) as colorless liquid; ^1H NMR (400 MHz, $CDCl_3$) δ 7.91-7.85 (2H,m), 7.38-7.31 (2H,m), 7.18-7.13 (2H, m), 7.09-7.04 (2H, m), 4.58 (1H, q, *J* = 6.8 Hz), 2.41 (2H, d, *J* = 7.2 Hz), 1.91-1.75 (1H, m), 1.51 (3H, d, *J* = 6.8 Hz), 0.87 (6H, d, *J* = 6.6 Hz); ^{13}C NMR (100MHz, $CDCl_3$) δ 199.2 (CO), 140.5 (C), 139.1 (C), 138.3 (C), 134.8 (CH), 130.2 (CH), 129.8 (CH), 128.7 (CH), 127.3 (CH), 47.7 (CH), 44.9 (CH_2), 30.1 (CH), 22.35 (CH_3), 22.34 (CH_3), 19.4 (CH_3); HRMS (ESI): Found: *m/z* 301.1349. Calcd for $C_{19}H_{22}OCl$ $(M+H)^+$ 301.1359.

2-(4-isobutylphenyl)-1-phenylpropan-1-one (3f)

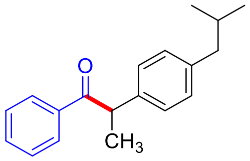

According to the general procedure **A** (0.2 mmol scale), product **3f** was isolated in 62% yield (33.1 mg) as yellow liquid; ^1H NMR (400 MHz, $CDCl_3$) δ 7.99-7.94 (2H,m), 7.50-7.44 (1H,m), 7.41-7.34 (2H, m), 7.22-7.16 (2H, m), 7.10-7.04 (2H, m), 4.67 (1H, q, *J* = 6.7 Hz), 2.41 (2H, d, *J* = 7.3 Hz), 1.89-1.74 (1H, m), 1.53 (3H, d, *J* = 6.8 Hz), 0.87 (6H, d, *J* = 6.6 Hz); ^{13}C NMR (100MHz, $CDCl_3$) δ 200.5 (CO), 140.3 (C), 138.6 (C), 136.6 (C), 132.7 (CH), 129.6 (CH), 128.8 (CH), 128.4 (CH), 127.4 (CH), 47.4 (CH), 44.9 (CH_2), 30.1 (CH), 22.4 (CH_3), 22.3 (CH_3), 19.5 (CH_3); HRMS (ESI): Found: *m/z* 289.1558. Calcd for $C_{19}H_{22}ONa$ $(M+Na)^+$ 3289.1568.

1-([1,1'-biphenyl]-4-yl)-2-(4-isobutylphenyl)propan-1-one (3g)

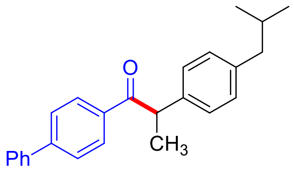

According to the general procedure **A** (0.1 mmol scale), product **3g** was isolated in 64% yield (22.0 mg) as white solid; mp: 88-89 ºC; ^{1}H NMR (500 MHz, $CDCl_3$) δ 8.07-8.01 (2H, m),7.64-7.55 (4H, m), 7.47-7.41 (2H, m), 7.41-7.35 (1H, m), 7.25-7.20 (2H, m), 7.11-7.06 (2H, m), 4.70 (1H, q, *J* = 6.7 Hz), 2.42 (2H, d, *J* = 7.3 Hz), 1.88-1.78 (1H, m), 1.56 (3H, d, *J* = 6.8 Hz), 0.88 (6H, d, *J* = 6.6 Hz); ^{13}C NMR (125 MHz, $CDCl_3$) δ 200.1 (CO), 145.3 (C), 140.3 (C), 139.9 (C), 138.7 (C), 135.2 (C), 129.7 (CH), 129.4 (CH), 128.7 (CH), 128.1 (CH), 127.4 (CH), 127.2 (CH), 127.1 (CH), 47.5 (CH), 44.9 (CH_2), 30.1 (CH), 22.4 (CH_3), 22.35 (CH_3), 19.5 (CH_3); HRMS (ESI): Found: *m/z* 343.2067. Calcd for $C_{25}H_{27}O$ $(M+H)^+$ 343.2062.

1-(4-(tert-butyl)phenyl)-2-(4-isobutylphenyl)propan-1-one (3h)

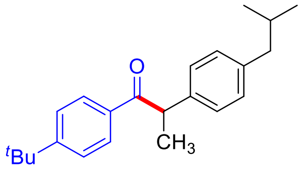

According to the general procedure **A** (0.1 mmol scale), product **3h** was isolated in 69% yield (22.1 mg) as yellow liquid; ^{1}H NMR (500 MHz, $CDCl_3$) δ 7.95-7.89 (2H, m), 7.43-7.38 (2H, m), 7.21(2H, d, *J* = 8.1 Hz), 7.07 (2H, d, *J* = 8.1 Hz), 4.67 (1H, q, *J* = 6.9 Hz), 2.42 (2H, d, *J* = 7.2 Hz), 1.87-1.77 (1H, m), 1.52 (3H, d, *J* = 6.9 Hz), 1.30 (9H, s), 0.88 (6H, d, *J* = 6.7 Hz); ^{13}C NMR (125 MHz, $CDCl_3$) δ 200.1 (CO), 156.3 (C), 140.2 (C), 138.8 (C), 134.0 (C), 129.6 (CH), 128.7 (CH), 127.4 (CH), 125.4 (CH), 47.3 (CH), 45.0 (CH_2), 35.0 (C), 31.0 (CH_3), 30.1(CH), 22.4 (CH_3), 22.35 (CH_3), 19.6 (CH_3); HRMS (ESI): Found: *m/z* 345.2202. Calcd for $C_{23}H_{30}ONa$ $(M+Na)^+$ 345.2194.

1-(2-fluorophenyl)-2-(4-isobutylphenyl)propan-1-one (3i)

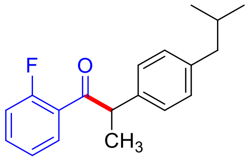

According to the general procedure **A** (0.1 mmol scale), product **3i** was isolated in 75%

yield (21.4 mg) as yellow liquid; ^{1}H NMR (400 MHz, $CDCl_3$) δ 7.70 (1H,td, *J* = 7.6, 1.8 Hz), 7.44-7.36 (1H,m), 7.16-7.10 (3H, m), 7.06-6.98 (3H, m), 4.58 (1H, q, *J* = 6.9 Hz), 2.39 (2H, d, *J* = 7.3 Hz), 1.86-1.74 (1H, m), 1.53 (3H, d, *J* = 6.9 Hz), 0.86 (6H, d, *J* = 6.6 Hz); ^{13}C NMR (100 MHz, $CDCl_3$) δ 200.0 (CO, d, *J* = 4.3 Hz), 160.9 (C, d, *J* = 252.1 Hz), 140.3 (C), 137.6 (C), 133.9 (CH, d, *J* = 8.9 Hz), 131.0 (CH, d, *J* = 2.9 Hz), 129.4 (CH), 127.8 (CH), 126.3 (C, d, *J* = 13.1 Hz), 124.3 (CH, d, *J* = 3.3 Hz), 116.4 (CH, d, *J* = 23.6 Hz), 51.5 (CH, d, *J* = 6.3 Hz), 45.0 (CH_2), 30.1 (CH), 22.3(CH_3), 18.8 (CH_3); ^{19}F NMR (376MHz, $CDCl_3$) δ -110.1 (m); HRMS (ESI): Found: *m/z* 285.1650. Calcd for $C_{19}H_{22}OF$ $(M+H)^+$ 285.1655.

2-(4-isobutylphenyl)-1-(2-methoxyphenyl)propan-1-one (3j)

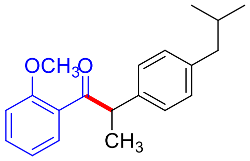

According to the general procedure **A** (0.1 mmol scale), product **3j** was isolated in 58% yield (17.1 mg) as colorless liquid; ^{1}H NMR (400 MHz, $CDCl_3$) δ 7.42 (1H, dd, *J* = 7.6, 1.8 Hz), 7.38-7.31 (1H, m), 7.14-7.09 (2H, m), 7.04-6.98 (2H, m), 6.93-6.83 (2H, m), 4.67 (1H, q, *J* =7.0 Hz), 3.83 (3H, s), 2.40 (2H, d, *J* = 7.2 Hz), 1.86-1.74 (1H, m), 1.50 (3H, d, *J* =7.0 Hz), 0.86 (6H, d, *J* = 6.7 Hz); ^{13}C NMR (100 MHz, $CDCl_3$) δ 204.4 (CO), 157.5 (C), 139.9 (C), 138.4 (C), 132.5 (CH), 130.3 (CH), 129.1 (CH), 129.0 (CH), 127.8 (CH), 120.5 (CH), 111.3 (CH), 55.4 (CH_3), 51.4 (CH), 45.9 (CH_2), 30.1 (CH), 22.34 (CH_3), 22.32 (CH_3), 18.6 (CH_3); HRMS (ESI): Found: *m/z* 319.1669.Calcd for $C_{20}H_{24}O_2Na$ $(M+Na)^+$ 319.1674.

2-(4-isobutylphenyl)-1-(m-tolyl)propan-1-one (3k)

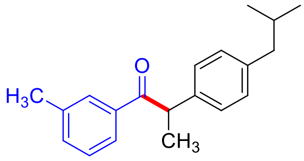

According to the general procedure **A** (0.1 mmol scale), product **3k** was isolated in 75% yield (20.9 mg) as colorless liquid; ^{1}H NMR (400 MHz, $CDCl_3$) δ 7.83-7.79 (1H,m), 7.79-7.74 (1H, m), 7.34-7.27 (2H, m), 7.23-7.18 (2H, m), 7.11-7.06 (2H, m), 4.68 (1H, q, *J* =6.9 Hz), 2.43 (3H, d, *J* = 7.1 Hz), 2.38 (3H,s), 1.89-1.77 (1H, m), 1.54 (3H, d, *J*

=6.9 Hz), 0.89 (6H, d, J = 6.6 Hz); ^{13}C NMR (100 MHz, $CDCl_3$) δ 200.8 (CO), 140.2 (C), 138.7 (C), 138.2 (C), 136.6 (C), 133.5 (CH_3), 129.6 (CH), 129.3 (CH), 128.3 (CH), 127.4 (CH), 126.0 (CH), 47.4 (CH), 45.0 (CH_2), 30.1 (CH), 22.36 (CH_3), 22.33 (CH_3), 21.3 (CH_3), 19.5 (CH_3); HRMS (ESI): Found: m/z 303.1717. Calcd for $C_{20}H_{24}ONa$ $(M+Na)^+$ 303.1725.

3-(2-(4-isobutylphenyl)propanoyl)benzonitrile (3l)

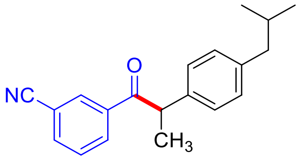

According to the general procedure **A** (0.1 mmol scale, 440 nm blue LED was used as light source), product **3l** was isolated in 36% yield (10.6 mg) as yellow oil; ^{1}H NMR (400 MHz, $CDCl_3$) δ 8.19 (1H, s), 8.14 (1H, d, J =8.0 Hz), 7.73 (1H, d, d, J = 8.0 Hz), 7.50 (1H, t, J = 8.0 Hz), 7.14 (2H, d, J = 8.0 Hz), 7.10-7.05 (2H, d, J = 8.0 Hz), 4.57 (1H, q, J =6.8 Hz), 2.43 (3H, d, J = 7.3 Hz), 1.87-1.75 (1H, m), 1.52 (3H, d, J =6.8 Hz), 0.86 (6H, d, J = 6.6 Hz); ^{13}C NMR (100 MHz, $CDCl_3$) δ 198.3 (CO), 140.9 (C), 137.6 (C), 137.3 (C), 135.5 (CH), 132.7 (CH), 132.4 (CH), 130.0 (CH), 129.4 (CH), 127.4 (CH), 118.0 (C), 112.9 (C), 48.0 (CH), 44.9 (CH_2), 30.1(CH), 22.33 (CH_3), 22.32 (CH_3), 19.3 (CH_3); HRMS (ESI): Found: m/z 292.1693. Calcd for $C_{20}H_{22}NO$ $(M+H)^+$ 292.1701.

1-(4-(allyloxy)phenyl)-2-(4-isobutylphenyl)propan-1-one (3m)

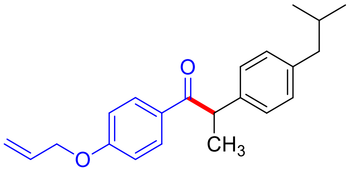

According to the general procedure **A** (0.1 mmol scale), product **3m** was isolated in 83% yield (26.7 mg) as yellow oil; ^{1}H NMR (400 MHz, $CDCl_3$) δ 7.98-7.91 (2H, m), 7.18 (2H, d, J = 8.1 Hz), 7.06 (2H, d, J = 8.0 Hz), 6.90-6.83 (2H, m), 6.08-5.94 (1H, m), 5.39 (1H, dd, J = 17.3, 1.2 Hz), 5.29 (1H, dd, J = 10.5, 1.2 Hz), 4.62 (1H, q, J =6.8 Hz), 4.57-4.51 (2H, m), 2.40 (2H, d, J = 7.3 Hz), 1.87-1.75 (1H, m), 1.50 (3H, d, J =6.9 Hz), 0.87 (6H, d, J = 6.6 Hz); ^{13}C NMR (100 MHz, $CDCl_3$) δ 199.1 (CO), 162.1 (C), 140.1 (C), 139.0 (C), 132.5 (CH), 131.0 (CH), 129.61 (C), 129.59 (CH), 127.3 (CH),

118.1 (CH_2), 114.3 (CH), 68.8 (CH_2O), 47.1 (CH), 45.0 (CH_2), 30.1 (CH), 22.36 (CH_3), 22.34 (CH_3), 19.5 (CH_3); HRMS (ESI): Found: *m/z* 323.2010. Calcd for $C_{22}H_{27}O_2$ $(M+H)^+$ 323.2011.

methyl 3-(2-(4-isobutylphenyl)propanoyl)benzoate (3n)

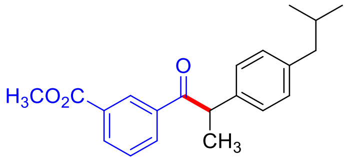

According to the general procedure **A** (0.1 mmol scale), product **3n** was isolated in 62% yield (20.2 mg) as colorless liquid; ^{1}H NMR (400 MHz, $CDCl_3$) δ 8.62 (1H, s), 8.12 (2H, t, *J* =7.0 Hz), 7.46 (1H, t, *J* = 7.7 Hz), 7.18 (2H, d, *J* = 8.0 Hz), 7.06 (2H, d, *J* = 8.0 Hz), 4.68 (1H, q, *J* =6.7 Hz), 3.92 (3H, s), 2.39 (2H, d, *J* = 7.1 Hz), 1.87-1.74 (1H, m), 1.54 (3H, d, *J* =6.9 Hz), 0.85 (6H, d, *J* = 6.6 Hz); ^{13}C NMR (100 MHz, $CDCl_3$) δ 199.7 (CO), 166.3 (CO), 140.5 (C), 138.1 (C), 136.8 (C), 132.4 (CH), 132.9 (CH), 130.5 (C), 129.9 (CH), 129.7 (CH), 128.7 (CH), 127.5 (CH), 52.3 (CH_3), 47.6 (CH), 45.0 (CH_2), 30.1 (CH), 22.33 (CH_3), 22.31 (CH_3), 19.3 (CH_3); HRMS (ESI): Found: *m/z* 325.1806.Calcd for $C_{21}H_{25}O_3$ $(M+H)^+$ 325.1804.

1-(3-benzoylphenyl)-2-(4-isobutylphenyl)propan-1-one (3o)

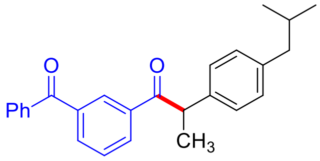

According to the general procedure **A** (0.1 mmol scale, two 440nm LEDs were used as light source), product **3o** was isolated in 55% yield (20.4 mg) as yellow oil; ^{1}H NMR (400 MHz, $CDCl_3$) δ 8.34 (1H, s), 8.18 (1H, d, *J* = 7.8 Hz), 7.94 (1H, d, *J* = 7.4 Hz), 7.75-7.67 (2H, m), 7.62 (1H, t, *J* = 7.4 Hz), 7.56-7.44 (3H, m), 7.13 (2H, d, *J* = 8.1 Hz), 7.06 (2H, d, *J* = 8.1 Hz), 4.64 (1H, q, *J* =6.6 Hz), 2.41 (2H, d, *J* = 7.2 Hz), 1.88-1.74 (1H, m), 1.52 (3H, d, *J* =6.8 Hz), 0.87 (6H, d, *J* = 6.6 Hz); ^{13}C NMR (100 MHz, $CDCl_3$) δ 199.6 (CO), 195.7 (CO), 140.5 (C), 138.1 (C), 137.7 (C), 137.0 (C), 136.5 (C), 133.8 (CH), 132.7 (CH), 132.4 (CH), 130.3 (CH), 130.0 (CH), 129.8 (CH), 128.8 (CH), 128.4 (CH), 127.5 (CH), 47.8 (CH), 45.0 (CH_2), 30.1 (CH), 22.35 (CH_3), 19.3 (CH_3); HRMS (ESI): Found: *m/z* 371.2020. Calcd for $C_{26}H_{27}O_2$ $(M+H)^+$ 371.2011.

2-(4-isobutylphenyl)-1-(quinolin-6-yl)propan-1-one (3p)

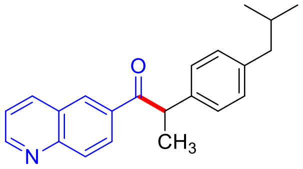

According to the general procedure **A** (0.1 mmol scale, two 427 nm blue LEDs were used), product **3p** was isolated in 54% yield (17.4 mg) as yellow oil; ^{1}H NMR (400 MHz, $CDCl_3$) δ 8.96 (1H, dd, *J* =4.2, 1.7 Hz), 8.45 (1H, d, *J* =1.8 Hz), 8.24 (1H, dd, *J* =8.9, 1.9 Hz), 8.21 (1H, d, *J* = 8.4 Hz), 8.07 (1H, d, *J* = 8.9 Hz), 7.43 (1H, dd, *J* = 8.4, 4.3 Hz), 7.25-7.20 (2H, m), 7.10-7.04 (2H, m), 4.79 (1H, q, *J* =6.9 Hz), 2.39 (2H, d, *J* = 7.2 Hz), 1.85-1.74 (1H, m), 1.58 (3H, d, *J* =6.8 Hz), 0.85 (6H, d, *J* = 6.6 Hz); ^{13}C NMR (100 MHz, $CDCl_3$) δ 200.0 (CO), 152.4 (CH), 149.8 (C), 140.5 (C), 138.4 (C), 137.6 (CH), 134.4 (C), 130.1 (CH), 129.82 (CH), 129.80 (CH), 128.4 (CH), 127.4 (CH), 121.8 (CH), 47.8 (CH), 45.0 (CH_2), 30.1 (CH), 22.34 (CH_3), 22.32 (CH_3), 19.5 (CH_3); HRMS (ESI): Found: *m/z* 318.1862. Calcd for $C_{22}H_{24}NO$ $(M+H)^+$ 318.1858.

1-(furan-2-yl)-2-(4-isobutylphenyl)propan-1-one (3q)

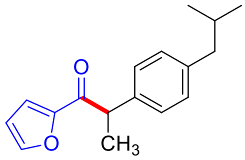

According to the general procedure **A** (0.2 mmol scale, two 427 nm blue LEDs were used), product **3q** was isolated in 59% yield (30.0 mg) as colorless liquid; ^{1}H NMR (400 MHz, $CDCl_3$) δ 7.55-7.50 (1H, m), 7.25-7.20 (2H, m), 7.15-7.11 (1H, m), 7.10-7.04 (2H, m), 6.45 (1H, d, *J* = 3.5, 1.7 Hz), 4.46 (1H, q, *J* =7.0 Hz), 2.41 (2H, d, *J* = 7.2 Hz), 1.88-1.75 (1H, m), 1.51 (3H, d, *J* =7.0 Hz), 0.88 (6H, d, *J* = 6.6 Hz); ^{13}C NMR (100 MHz, $CDCl_3$) δ 189.6 (CO), 152.2 (C), 146.2 (CH), 140.4 (C), 137.9 (C), 129.4 (CH), 127.5 (CH), 117.8 (CH), 112.1 (CH), 47.5 (CH), 45.0 (CH_2), 30.1 (CH), 22.3 (CH_3), 18.3 (CH_3); HRMS (ESI): Found: *m/z* 279.1354. Calcd for $C_{17}H_{20}O_2Na$ $(M+H)^+$ 279.1361.

2-(4-isobutylphenyl)-1-(thiophen-2-yl)propan-1-one (3r)

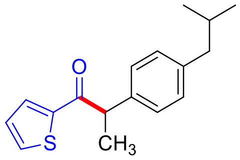

According to the general procedure **A** (0.2 mmol scale, two 427 nm blue LEDs were used), product **3r** was isolated in 78% yield (42.6 mg) as yellow liquid; ^{1}H NMR (400 MHz, $CDCl_3$) δ 7.69 (1H, dd, *J* = 1.0, 3.8 Hz), 7.55 (1H, dd, *J* =5.0, 1.0 Hz), 7.26-7.20 (2H, m), 7.10-7.06 (2H, m), 7.04 (1H,dd, *J* =4.9, 3.9 Hz), 4.48 (1H, q, *J* =6.9 Hz), 2.42 (2H, d, *J* = 7.1 Hz), 1.88-1.76 (1H, m), 1.54 (3H, d, *J* =6.9 Hz), 0.88 (6H, d, *J* = 6.7 Hz); ^{13}C NMR (100 MHz, $CDCl_3$) δ 193.5 (CO), 143.8 (C), 140.5 (C), 138.4 (C), 133.4 (CH), 132.3 (CH), 129.6 (CH), 128.0 (CH), 127.4 (CH), 48.9 (CH), 45.0 (CH_2), 30.1 (CH), 22.35 (CH_3), 22.34 (CH_3), 19.1 (CH_3); HRMS (ESI): Found: *m/z* 295.1134. Calcd for $C_{17}H_{20}ONaS$ $(M+Na)^+$ 295.1133.

2-(4-isobutylphenyl)-1-(pyridin-3-yl)propan-1-one (3s)

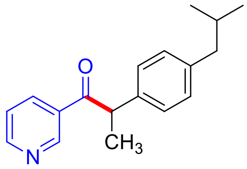

According to the general procedure **A** (0.1 mmol scale, two 427 nm blue LEDs were used), product **3s** was isolated in 49% yield (13.2 mg) as yellow oil; ^{1}H NMR (400 MHz, $CDCl_3$) δ 9.14 (1H, d, *J* = 1.8 Hz),8.67 (1H, dd, *J* =4.8, 1.7 Hz), 8.20 (1H, dt, *J* = 8.1, 2.0 Hz), 7.32 (1H, ddd, *J* = 8.1, 5.0, 0.7 Hz), 7.18-7.13 (2H, m), 7.09-7.04 (2H, m), 4.59 (1H, q, *J* =6.8Hz), 2.40 (2H, d, *J* = 7.18 Hz), 1.86-1.75 (1H, m), 1.53 (3H, d, *J* =6.8 Hz), 0.86 (6H, d, *J* = 6.6 Hz); ^{13}C NMR (100 MHz, $CDCl_3$) δ 199.2 (CO), 153.0 (CH), 150.2 (CH), 140.7 (C), 137.7 (C), 136.1 (CH), 131.8 (C), 129.9 (CH), 127.5 (CH), 123.5 (CH), 48.2 (CH), 45.0 (CH_2), 30.1 (CH), 22.3 (CH_3), 19.1 (CH_3); HRMS (ESI): Found: *m/z* 268.1700. Calcd for $C_{18}H_{22}NO$ $(M+H)^+$ 268.1701.

2-(4-isobutylphenyl)-1-(3-(4,4,5,5-tetramethyl-1,3,2-dioxaborolan-2-yl)phenyl)propan-1-one (3t)

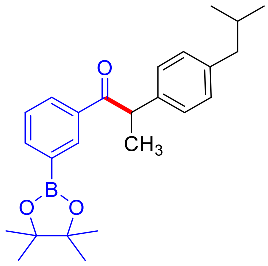

According to the general procedure **A** (0.1 mmol scale), product **3t** was isolated in 52%

yield (20.4 mg) as colorless oil; ^{1}H NMR (400 MHz, $CDCl_3$) δ 8.41 (s, 1H), 8.01 (d, *J* = 7.9 Hz, 1H), 7.90 (d, *J* = 7.3 Hz, 1H), 7.37 (t, *J* = 7.6 Hz, 1H), 7.19 (d, *J* = 8.0 Hz, 2H), 7.05 (d, *J* = 8.0 Hz, 2H), 4.73 (q, *J* = 6.8 Hz, 1H), 2.39 (d, *J* = 7.2 Hz, 2H), 1.86 – 1.74 (m, 1H), 1.53 (d, *J* = 6.9 Hz, 3H), 1.34 (d, *J* = 2.5 Hz, 12H), 0.86 (dd, *J* = 6.6, 1.2 Hz, 6H); ^{13}C NMR (100MHz, $CDCl_3$) δ 200.8 (CO), 140.2 (C), 138.9 (CH), 138.5 (C), 136.0 (C), 135.1 (CH), 131.4 (CH), 129.6 (CH), 127.9 (CH), 127.5 (CH), 84. (C-O), 47.2 (CH), 45.0 (CH_2), 30.1 (CH), 24.9 (CH_3), 24.8 (CH_3), 22.44 (CH_3), 22.3 (CH_3), 19.3 (CH_3).; ^{11}B NMR (128.4MHz, $CDCl_3$) δ 31.2 (s, br); HRMS (ESI): Found: *m/z* 415.2432. Calcd for $C_{25}H_{33}O_3BNa$ $(M+Na)^+$ 425.2420.

2-(4-isobutylphenyl)-1-(4-(prop-2-yn-1-yloxy)phenyl)propan-1-one (3u)

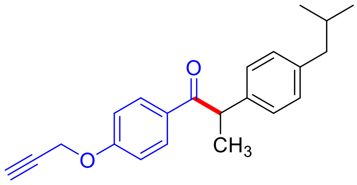

According to the general procedure **A** (0.1 mmol scale), product **3u** was isolated in 81% yield (25.8 mg) as colorless liquid; ^{1}H NMR (400 MHz, $CDCl_3$) δ 7.96 (d, *J* = 8.9 Hz, 2H), 7.18 (d, *J* = 8.0 Hz, 2H), 7.06 (d, *J* = 8.0 Hz, 2H), 6.94 (d, *J* = 8.9 Hz, 2H), 4.70 (d, *J* = 2.4 Hz, 2H), 4.61 (q, *J* = 6.8 Hz, 1H), 2.52 (t, *J* = 2.3 Hz, 1H), 2.40 (d, *J* = 7.2 Hz, 2H), 1.88-1.74 (m, 1H), 1.50 (d, *J* = 6.8 Hz, 3H), 0.87 (d, *J* = 6.6 Hz, 6H); ^{13}C NMR (100MHz, $CDCl_3$) δ 199.0 (CO), 160.9 (C), 140.20 (C), 138.9 (C), 131.0 (CH), 130.3 (C), 129.6 (CH), 127.4 (CH), 114.4 (CH), 77.8 (C), 76.0 (CH), 55.7 (CH_2), 47.2 (CH), 45.0 (ArCH2), 30.1 (CH), 22.4 (CH_3), 22.3 (CH_3), 19.5 (CH_3); HRMS (ESI): Found: *m/z* 343.1674. Calcd for $C_{22}H_{24}O_2Na$ $(M+Na)^+$ 343.1674.

1-(4-(2-chloroethoxy)phenyl)-2-(4-isobutylphenyl)propan-1-one (3v)

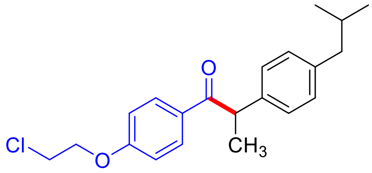

According to the general procedure **A** (0.1 mmol scale), product **3v** was isolated in 83% yield (28.8 mg) as colorless liquid; ^{1}H NMR (400 MHz, $CDCl_3$) δ 7.95 (d, *J* = 8.9 Hz, 2H), 7.18 (d, *J* = 8.0 Hz, 2H), 7.06 (d, *J* = 8.0 Hz, 2H), 6.87 (d, *J* = 8.9 Hz, 2H), 4.61

(q, J = 6.8 Hz, 1H), 4.23 (t, J = 5.8 Hz, 2H), 3.79 (t, J = 5.8 Hz, 2H), 2.40 (d, J = 7.2 Hz, 2H), 1.89-1.73 (m, 1H), 1.50 (d, J = 6.8 Hz, 3H), 0.87 (d, J = 6.6 Hz, 6H); ^{13}C NMR (100MHz, $CDCl_3$) δ 199.0 (CO), 161.6 (C), 140.2 (C), 138.92 (C), 131.1 (CH), 130.1 (C), 129.6 (CH), 127.3 (CH), 114.2 (CH), 67.9 (CH_2), 47.1 (CH), 45.0 (CH_2Cl), 41.5 (CH_2), 30.1 (CH), 22.4 (CH_3), 22.34 (CH_3), 19.5 (CH_3); HRMS (ESI): Found: *m/z* 367.1451. Calcd for $C_{21}H_{25}O_2NaCl$ $(M+Na)^+$ 367.1441.

1-(4-(3-bromopropoxy)phenyl)-2-(4-isobutylphenyl)propan-1-one (3w)

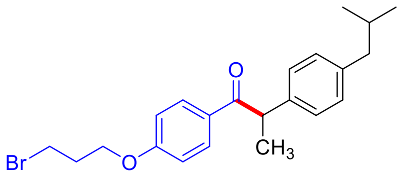

According to the general procedure **A** (0.1 mmol scale), product **3w** was isolated in 19% yield (7.5 mg) as colorless liquid; ^{1}H NMR (400 MHz, $CDCl_3$) δ 7.97-7.91 (m, 2H), 7.20-7.15 (m, 2H), 7.05 (d, J = 8.1 Hz, 1H), 6.88-6.83 (m, 2H), 4.61 (q, J = 6.9 Hz, 1H), 4.12 (t, J = 5.8 Hz, 2H), 3.57 (t, J = 6.4 Hz, 2H), 2.40 (d, J = 7.2 Hz, 2H), 2.31 (p, J = 6.1 Hz, 2H), 1.86-1.74 (m, 1H), 1.50 (d, J = 6.9 Hz, 3H), 0.86 (d, J = 6.6 Hz, 6H); ^{13}C NMR (100MHz, $CDCl_3$) δ 199.1 (CO), 162.2 (C), 140.2 (C), 139.0 (C), 131.1 (CH), 129.8 (C), 129.6 (CH), 127.4 (CH), 114.1 (CH), 65.4 (CH_2), 47.1 (CH), 45.0 (CH_2), 32.1 (CH_2), 30.1 (CH), 29.6 (CH_2), 22.38 (CH_3), 22.36 (CH_3), 19.5 (CH_3); HRMS (ESI): Found: *m/z* 403.1278. Calcd for $C_{22}H_{28}O_2Br$ $(M+H)^+$ 403.1273.

3-(2-fluoro-[1,1'-biphenyl]-4-yl)butan-2-one (3x)

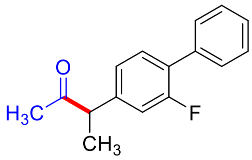

According to the general procedure **A** (0.1 mmol scale), product **3x** was isolated in 64% yield (30.8 mg) as colorless liquid; ^{1}H NMR (400 MHz, $CDCl_3$) δ 7.57-7.51 (m, 2H), 7.48-7.34 (m, 4H), 7.06 (m, 2H), 3.79 (q, J = 7.0 Hz, 1H), 2.12 (s, 3H), 1.44 (d, J = 7.0 Hz, 3H); ^{13}C NMR (100MHz, $CDCl_3$) δ 208.0 (CO), 159.8 (CF, d, J = 249.0 Hz), 141. (C, d, J = 7.5 Hz), 135.3 (C), 131.1 (CH, d, J = 4.0 Hz), 128.9 (CH, d, J = 2.9 Hz), 128.4 (CH), 127.9 (C, d, J = 13.5 Hz), 127.7 (CH), 123.8 (CH, d, J = 3.4 Hz), 115.5 (CH, d, J = 23.4 Hz), 53.1 (CH), 28.5 (CH_3), 17.1 (CH_3); ^{19}F NMR (376 MHz, $CDCl_3$)

δ -117.2 (m); HRMS (ESI): Found: *m/z* 243.1184. Calcd for $C_{16}H_{16}OF$ $(M+H)^+$ 243.1185.

3-(6-methoxynaphthalen-2-yl)butan-2-one (3y)

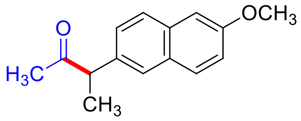

According to the general procedure **A** (0.2 mmol scale, 1.2 equiv Cs_2CO_3 was used; two 440 nm blue LEDs was used as light source), product **3y** was isolated in 76% yield (34.6 mg) as white solid; mp: 69-70 °C; ^{1}H NMR (500 MHz, $CDCl_3$) δ 7.73 (1H, d, *J* =5.6 Hz), 7.71 (1H, d, *J* = 5.9 Hz), 7.61 (1H, s), 7.29 (1H, dd, *J* = 8.5, 1.6 Hz), 7.17 (1H, dd, *J* = 8.9, 2.5 Hz), 7.13 (1H, d, *J* = 2.3 Hz), 3.92 (3H, s), 3.88 (1H, q, *J* =7.2Hz), 2.07 (3H, s), 1.47 (3H, d, *J* = 7.0 Hz); ^{13}C NMR (125MHz, $CDCl_3$) δ 209.0 (CO), 157.7 (C), 135.7 (C), 133.7 (C), 129.1 (CH), 129.08 (C), 127.5 (CH), 126.4 (CH), 126.3 (CH), 119.1 (CH), 105.6 (CH), 55.3 (CH_3O), 53.6 (CH), 28.4 (CH_3), 17.2 (CH_3); HRMS (ESI): Found: *m/z* 229.1217. Calcd for $C_{15}H_{17}O_2$ $(M+H)^+$ 229.1229.

2-(6-methoxynaphthalen-2-yl)pentan-3-one (3z)

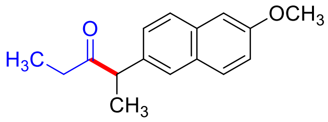

According to the general procedure **A** (0.2 mmol scale, 1.2 equiv Cs_2CO_3 was used; two 440 nm blue LEDs was used as light source), product **3z** was isolated in 64% yield (31.1 mg) as pink liquid; ^{1}H NMR (400 MHz, $CDCl_3$) δ 7.71 (1H, s), 7.69 (1H, s), 7.60 (1H, s), 7.29 (1H, dd, *J* = 8.6, 1.8 Hz), 7.16 (1H, dd, *J* = 8.9, 2.5 Hz), 7.12 (1H, d, *J* = 2.3 Hz), 3.92 (3H, s), 3.89 (1H, q, *J* =7.1Hz), 2.49-2.31 (2H, m), 1.47 (3H, d, *J* = 7.0 Hz), 0.97 (3H, t, *J* =7.3 Hz); ^{13}C NMR (100MHz, $CDCl_3$) δ 211.7 (CO), 157.7 (C), 136.0 (C), 133.6 (C), 129.1 (CH), 129.06 (C), 127.4 (CH), 126.4 (CH), 126.3 (CH), 119.1 (CH), 105.6 (CH), 55.3 (CH_3), 52.6 (CH), 34.2 (CH_2), 17.5 (CH_3), 8.0 (CH_3); HRMS (ESI): Found: *m/z* 243.1379. Calcd for $C_{16}H_{19}O_2$ $(M+H)^+$ 243.1385.

4-(6-methoxynaphthalen-2-yl)-1-phenylpentan-3-one (3aa)

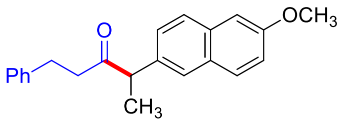

According to the general procedure **A** (0.1 mmol scale, 1.2 equiv Cs_2CO_3 was used;

two 440 nm blue LEDs was used as light source), product **3aa** was isolated in 74% yield (23.4 mg) as colorless oil; ^{1}H NMR (400 MHz, $CDCl_3$) δ 7.70 (1H, d, *J* = 6.4 Hz), 7.68 (1H, d, *J* = 6.8 Hz), 7.55 (1H, s), 7.26-7.10 (6H, m), 7.09-7.01 (2H, m), 3.93 (3H, s), 3.85 (1H, q, *J* =7.1 Hz), 2.91-2.64 (4H, m), 1.46 (3H, d, *J* =6.9 Hz); ^{13}C NMR (100MHz, $CDCl_3$) δ 210.0 (CO), 157.7 (C), 141.0 (C), 135.5 (C), 133.6 (C), 129.2 (CH), 129.0 (C), 128.3 (CH), 128.2 (CH), 127.5 (CH), 126.4 (CH), 126.3 (CH), 125.9 (CH), 119.1 (CH), 105.6 (CH), 55.3 (CH_3), 53.0 (CH), 42.5 (CH_2), 29.9 (CH_2), 17.3 (CH_3); HRMS (ESI): Found: *m/z* 319.1702. Calcd for $C_{22}H_{23}O_2$ $(M+H)^+$ 319.1968.

2-(4-chlorophenyl)-1-(4-methoxyphenyl)propan-1-one (3ab)

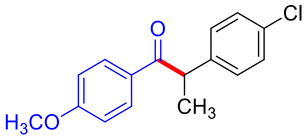

According to the general procedure **A** (0.2 mmol scale), product **3ab** was isolated in 52% yield (28.6 mg) as yellow liquid; ^{1}H NMR (400 MHz, $CDCl_3$) δ 7.96-7.87 (2H, m), 7.26-7.18 (4H, m), 6.90-6.83 (2H, m), 4.63 (1H, q, *J* = 6.8 Hz), 3.82 (3H, s), 1.50 (3H, d, *J* = 6.9 Hz); ^{13}C NMR (100MHz, $CDCl_3$) δ 198.4 (CO), 163.4 (C), 140.3 (C), 132.6 (C), 131.0 (CH), 129.2 (C), 129.1 (CH), 129.0 (CH), 113.7 (CH), 55.4 (CH_3), 46.7 (CH), 19.4 (CH_3); HRMS (ESI): Found: *m/z* 297.0647. Calcd for $C_{16}H_{15}ONaCl$ $(M+Na)^+$ 297.0658.

1-(4-methoxyphenyl)-2-phenylbutan-1-one (3ac)

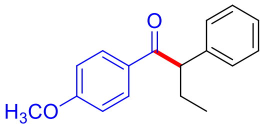

According to the general procedure **A** (0.2 mmol scale), product **3ac** was isolated in 77% yield (39.2 mg) as white solid; mp: 45-46 ºC; ^{1}H NMR (400 MHz, $CDCl_3$) δ 7.99-7.92 (2H, m), 7.33-7.24 (4H, m), 7.21-7.15 (1H, m), 6.89-6.81 (2H, m), 4.40 (1H, t, *J* = 7.2 Hz), 3.80 (3H, s), 2.25-2.12 (1H, m), 1.91-1.77 (1H, m), 0.89 (3H, t, *J* = 7.4 Hz); ^{13}C NMR (100MHz, $CDCl_3$) δ 198.6 (CO), 163.2 (C), 140.1 (C), 130.9 (CH), 130.0 (C), 128.7 (CH), 128.1 (CH), 126.8 (CH), 113.6 (CH), 55.3 (CH_3), 55.0 (CH), 27.1 (CH_2),

12.3 (CH_3); HRMS (ESI): Found: *m/z* 277.1209. Calcd for $C_{17}H_{18}ONa$ $(M+Na)^+$ 277.1204.

1-(4-methoxyphenyl)-2,3-diphenylpropan-1-one (3ad)

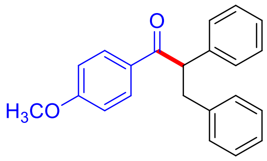

According to the general procedure **A** (0.1 mmol scale), product **3ad** was isolated in 74% yield (23.4 mg) as white solid; mp: 100-101 °C; ^{1}H NMR (400 MHz, $CDCl_3$) δ 7.96-7.87 (2H, m), 7.29-7.23 (4H, m), 7.22-7.11 (4H, m), 7.11-7.06 (2H, m), 6.86-6.79 (2H, m), 4.78 (1H, t, J = 7.3 Hz), 3.80 (3H, s), 3.58 (1H, dd, J = 13.8, 7.5 Hz), 3.07 (1H, dd, J = 13.7, 7.0 Hz); ^{13}C NMR (100 MHz, $CDCl_3$) δ 197.7 (CO), 163.2 (C), 139.9 (C), 139.5 (C), 131.0 (CH), 129.7 (C), 129.1 (CH), 128.8 (CH), 128.19 (CH), 128.16 (CH), 127.0 (CH), 126.0 (CH), 113.6 (CH), 55.5 (CH_3), 55.3 (CH), 40.1 (CH_2); HRMS (ESI): Found: *m/z* 317.1548.Calcd for $C_{22}H_{21}O_2$ $(M+H)^+$ 317.1542.

2-cyclohexyl-1-(4-methoxyphenyl)-2-phenylethanone (3ae)

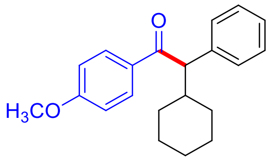

According to the general procedure **A** (0.1 mmol scale), product **3ae** was isolated in 64% yield (19.8 mg) as white solid; mp: 96-97 °C; ^{1}H NMR (400 MHz, $CDCl_3$) δ 8.02-7.94 (2H, m), 7.37-7.31 (2H, m), 7.27-7.24 (2H, m), 7.21-7.15 (1H, m), 6.92-6.84 (2H, m), 4.26 (1H, d, J =10.3 Hz), 3.82 (3H, s), 2.36-2.21(1H, m), 1.88-1.77 (1H, m), 1.70-1.54(4H, m), 1.38-1.24 (2H, m), 1.23-1.07 (2H, m), 1.02-0.9 (1H, m), 0.9-0.76 (1H, m); ^{13}C NMR (100 MHz, $CDCl_3$) δ 199.2 (CO), 163.3 (C), 138.4 (C), 130.8 (C), 130.78 (C), 130.75 (CH), 128.8 (CH), 128.6 (CH), 126.8 (CH), 113.7 (CH), 59.6(CH), 55.4(CH_3), 41.1 (CH), 32.6(CH_2), 30.8(CH_2), 26.5(CH_2), 26.2(CH_2), 26.1(CH_2); HRMS (ESI): Found: *m/z* 309.1856. Calcd for $C_{21}H_{25}O_2$ $(M+H)^+$ 309.1855.

1-(4-methoxyphenyl)-2-(p-tolyl)pent-4-en-1-one (3af)

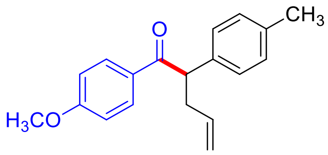

According to the general procedure **A** (0.2 mmol scale), product **3af** was isolated in 77% yield (44.3 mg) as yellow liquid; ^{1}H NMR (400 MHz, $CDCl_3$) δ 8.01-7.92 (2H, m), 7.20 (2H, d, *J* = 7.9 Hz), 7.10 (2H, d, *J* = 7.9 Hz), 6.89-6.81 (2H, m), 5.84-5.67 (1H, m), 5.04 (1H, d, *J* = 17.3 Hz), 4.97 (1H, d, *J* = 10.3 Hz), 4.56 (1H, t, *J* = 7.3 Hz), 3.81 (3H, s), 2.99-2.87 (1H, m), 2.60-2.49 (1H, m), 2.28 (3H, s); ^{13}C NMR (100 MHz, $CDCl_3$) δ 197.8 (CO), 163.2 (C), 136.5 (C), 136.4 (C), 136.2 (CH), 130.9 (CH), 129.6 (C), 129.5 (CH), 127.9 (CH), 116.3 (CH), 113.6 (CH), 55.3 (CH_3), 52.7 (CH), 38.1 (CH_2), 20.9 (CH_3); HRMS (ESI): Found: *m/z* 289.1210. Calcd for $C_{18}H_{18}O_2Na$ $(M+Na)^+$ 289.1204.

(2,3-dihydro-1H-inden-1-yl)(4-methoxyphenyl)methanone (3ag)

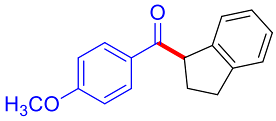

According to the general procedure **A** (0.2 mmol scale, two 427 nm blue LEDs were used), product **3ag** was isolated in 41% yield (20.9 mg) as white solid, mp: 65-66 °C; ^{1}H NMR (400 MHz, $CDCl_3$) δ 8.10-8.03 (2H, m), 7.30-7.26 (1H, m), 7.19 (1H, t, *J* = 6.9 Hz), 7.10 (1H, t, *J* = 6.9 Hz), 7.06 (1H, d, *J* = 7.4 Hz), 7.03-6.97 (2H, m), 5.01 (1H, t, *J* = 7.7 Hz), 3.90 (3H, s), 3.22-3.11 (1H, m), 3.06-2.95 (1H, m), 2.56-2.36 (2H, m); ^{13}C NMR (100MHz, $CDCl_3$) δ 199.0 (CO), 163.6 (C), 144.6 (C), 141.9 (C), 131.2 (CH), 130.0 (C), 127.2 (CH), 126.2 (CH), 124.9 (CH), 124.8 (CH), 113.9 (CH), 55.5 (CH_3), 52.1 (CH), 32.0 (CH_2), 29.6 (CH_2); HRMS (ESI): Found: *m/z* 253.1228. Calcd for $C_{17}H_{17}O_2$ $(M+H)^+$ 253.1229.

(4-methoxyphenyl)(1,2,3,4-tetrahydronaphthalen-1-yl)methanone (3ah)

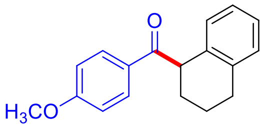

According to the general procedure **A** (0.2 mmol scale), product **3ah** was isolated in 66% yield (26.6 mg) as colorless oil; ^{1}H NMR (400MHz, $CDCl_3$) δ 8.05-7.96 (2H, m), 7.19-7.12 (2H, m), 7.11-7.04 (1H, m), 7.00-6.94 (2H, m), 6.93-6.86 (1H, m), 4.79 (1H, t, *J* = 6.9 Hz), 3.89 (3H, s), 2.97-2.87 (1H, m), 2.87-2.76 (1H, m), 2.23-2.13 (1H, m), 2.12-2.02 (1H, m), 2.02-1.91 (1H, m), 1.84-1.73 (1H, m); ^{13}C NMR (100 MHz, $CDCl_3$) δ 201.2 (CO), 163.4 (C), 137.6 (C), 135.1 (C), 131.1 (CH), 129.5 (C), 129.4 (CH), 129.3

(CH), 126.5 (CH), 125.8 (CH), 113.8 (CH), 55.5 (CH_3), 47.1 (CH), 29.3 (CH_2), 27.8 (CH_2), 20.8 (CH_2); HRMS (ESI): Found: *m/z* 289.1201. Calcd for $C_{18}H_{18}O_2Na$ $(M+Na)^+$ 289.1204.

1-(4-methoxyphenyl)-2-methyl-2-phenylpropan-1-one (3ai)

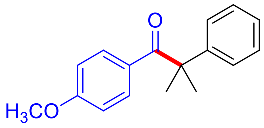

According to the general procedure **A** (0.2 mmol scale, two 427 nm blue LEDs were used), product **3ai** was isolated in 50% yield (25.4 mg) as colorless oil; ^{1}H NMR (400 MHz, $CDCl_3$) δ 7.57-7.50 (2H, d, J = 9.0 Hz), 7.37-7.20 (5H, m), 6.70 (2H, d, J = 8.9 Hz), 3.75 (3H, s), 1.59 (6H, s); ^{13}C NMR (100 MHz, $CDCl_3$) δ 202.0 (CO), 162.2 (C), 146.0 (C), 132.3 (CH), 128.9 (CH), 128.5 (C), 126.6.0 (CH), 125.6 (CH), 113.1 (CH), 55.3 (CH_3), 51.2 (C), 28.1 (CH_3); HRMS (ESI): Found: *m/z* 277.1204. Calcd for $C_{17}H_{18}O_2Na$ $(M+Na)^+$ 277.1204.

(4-methoxyphenyl)(1-phenylcyclobutyl)methanone (3aj)

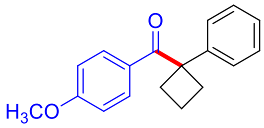

According to the general procedure **A** (0.2 mmol scale, two 427 nm blue LEDs were used), product **3aj** was isolated in 81% yield (43.1 mg) as white solid; ^{1}H NMR (400MHz, $CDCl_3$) δ 7.77-7.68 (2H, m), 7.45-7.38 (2H, m), 7.37-7.30 (2H, m), 7.20 (1H, t, J = 7.3 Hz), 6.80-6.73 (2H, m), 3.77 (3H, s), 2.99-2.87 (2H, m), 2.60-2.50 (2H, m), 2.14-2.00 (1H, m), 1.97-1.84 (1H, m); ^{13}C NMR (100 MHz, $CDCl_3$) δ 199.8 (CO), 162.6 (C), 143.6 (C), 132.0 (CH), 128.9 (CH), 127.0 (C), 126.4 (CH), 125.5 (CH), 113.3 (CH), 56.9 (C), 55.3 (CH_3), 32.3 (CH_2), 16.0 (CH_2); HRMS (ESI): Found: *m/z* 289.1195. Calcd for $C_{18}H_{18}O_2Na$ $(M+Na)^+$ 289.1204.

(4-methoxyphenyl)(1-phenylcyclopentyl)methanone (3ak)

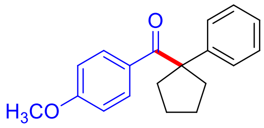

According to the general procedure **A** (0.2 mmol scale, two 440 nm blue LEDs were

used), product **3ak** was isolated in 48% yield (23.0 mg) as white solid, mp: 85-86 °C; ^{1}H NMR (400MHz, $CDCl_3$) δ 7.69-7.63 (2H, m), 7.33-7.25 (4H, m), 7.23-7.17 (1H, m), 6.76-6.69 (2H, m), 3.77 (3H, s), 2.57-2.45 (2H, m), 2.14-2.03 (2H, m), 1.80-1.63 (4H, m); ^{13}C NMR (100MHz, $CDCl_3$) δ 200.4 (C), 162.3 (C), 145.2 (C), 132.3 (CH), 128.8 (CH), 128.6 (C), 126.3 (CH), 125.9 (CH), 113.1 (CH), 63.1 (C), 55.3 (CH_3), 37.7 (CH_3), 24.8 (CH_3); HRMS (ESI): Found: *m/z* 303.1364. Calcd for $C_{19}H_{20}O_2Na$ $(M+Na)^+$ 303.1361.

(4-methoxyphenyl)(1-phenylcyclohexyl)methanone (3al)

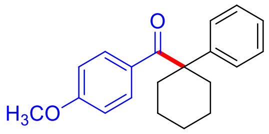

According to the general procedure **A** (0.1 mmol scale, two 427 nm blue LEDs were used), product **3al** was isolated in 65% yield (19.1 mg) as white solid, mp: 88-89 °C; ^{1}H NMR (400MHz, $CDCl_3$) δ 7.44-7.33 (6H, m), 7.28-7.23 (1H, m), 6.76-6.66 (2H, m), 3.76 (3H, s), 2.52 (2H, d, J = 13.3 Hz), 1.85-1.73 (2H, m), 1.69-1.58 (3H, m), 1.53-1.39 (2H, m), 1.34-1.26 (1H, m); ^{13}C NMR (100MHz, $CDCl_3$) δ 203.0 (CO), 161.8 (C), 144.9 (C), 131.2 (CH), 130.5 (C), 129.0 (CH), 126.7 (CH), 126.1 (CH), 113.0 (CH), 55.3 (C), 55.2 (CH_3), 36.4 (CH_2), 25.9 (CH_2), 23.3 (CH_2); HRMS (ESI): Found: *m/z* 295.1696. Calcd for $C_{20}H_{23}O_2$ $(M+H)^+$ 295.1698.

tert-butyl 2-(4-methoxybenzoyl)piperidine-1-carboxylate (3am)

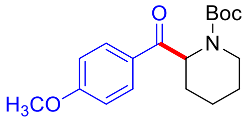

According to the general procedure **A** (0.1 mmol scale), product **3am** was isolated in 55% yield (17.4 mg) as white solid, mp: 85-86 °C; The spectra data of **3am** were consistent with the previous reported literature[3].

tert-butyl 2-(4-fluorobenzoyl)pyrrolidine-1-carboxylate (3an)

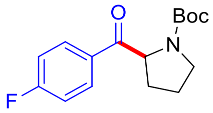

According to the general procedure **A** (0.1 mmol scale), product **3an** was isolated in 50% yield (14.6 mg) as yellow liquid; Mixture of rotamers (ratio: 45/55); ^{1}H NMR(500

MHz, $CDCl_3$) δ 8.05-7.95 (2H, m), 7.18-7.08 (2H, m), 5.31-5.25 (0.43H, m), 5.17-5.10 (0.55H, m),3.71-3.64 (0.55H, m), 3.64-3.59 (0.43H, m), 3.58-3.51 (0.55H, m), 3.51-3.43 (0.43H, m), 2.38-2.21 (1H, m), 2.02-1.82 (3H, m), 1.46 (3.87H, s), 1.25 (4.95H, s); ^{13}C NMR (125 MHz, $CDCl_3$) δ 197.4 (CO), 197.0 (CO), 165.8 (CF, d, J = 255.9 Hz), 154.5 (CO), 153.7 (CO), 131.6 (C, d, J = 3.0 Hz), 131.5 (C, d, J = 3.0 Hz), 131.2 (CH, d, J = 9.2 Hz), 130.8 (CH, d, J = 9.2 Hz), 115.9 (CH, d, J = 21.5 Hz), 115.7 (CH, d, J = 21.5 Hz), 79.8 (C), 79.7 (C), 61.2 (CH), 60.9 (CH), 46.8 (CH_2), 46.6 (CH_2), 30.8 (CH_2), 29.8 (CH_2), 28.4 (CH_3), 28.1 (CH_3), 24.2 (CH_2), 23.6 (CH_2); ^{19}F NMR (376MHz, $CDCl_3$) δ -104.7 (m), -104.9 (m); HRMS (ESI): Found: *m/z* 316.1237. Calcd for $C_{16}H_{20}NO_3NaF$ $(M+Na)^+$ 316.1325.

tert-butyl 4,4-difluoro-2-(4-methoxybenzoyl)pyrrolidine-1-carboxylate (3ao)

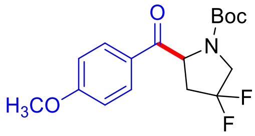

According to the general procedure **A** (0.1 mmol scale, two 427 nm blue LEDs were used), product **3ao** was isolated in 63% yield (21.5 mg) as yellow oil; Mixture of rotamers (ratio: 48/52); ^{1}H NMR (400MHz, $CDCl_3$) δ 7.93 (2H, dd, J = 8.4, 6.4 Hz), 6.95 (2H, dd, J = 10.7, 9.0 Hz), 5.46 (0.48H, dd, J = 9.4, 5.7 Hz), 5.35 (0.52H, dd, J = 8.8, 7.0 Hz), 4.06-3.76 (5H, m), 2.88-2.69 (1H, m), 2.46-2.26 (1H, m), 1.46 (4.32H, s), 1.26 (4.68H, s); ^{13}C NMR (100MHz, $CDCl_3$) δ 194.9 (CO), 194.4 (C), 164.1 (C), 164.0 (C), 153.8 (CO), 153.2 (CO), 130.9 (CH), 130.6 (CH), 127.4 (C), 127.3 (C), 126.8 (CF_2, t, J = 249.6 Hz), 125.9 (CF_2, t, J = 249.5 Hz), 114.1 (CH), 114.0 (CH), 81.0 (C), 58.5 (CH), 58.4 (CH), 55.5 (CH_3), 53.9 (CH_2, t, J = 32.5 Hz), 53.3 (CH_2, t, J = 32.9 Hz), 39.1 (CH_2, t, J = 26.1 Hz), 53.3 (CH_2, t, J = 25.5 Hz), 28.3 (CH_3), 28.0 (CH_3); ^{19}F NMR (376MHz, $CDCl_3$) δ -95.5 (m), -96.2 (m), -96.3 (m), -99.2 (m), -99.8 (m), -101.6 (m), -102.3 (m); HRMS (ESI): Found: *m/z* 342.1515. Calcd for $C_{17}H_{22}NO_4F_2$ $(M+H)^+$ 342.1517.

tert-butyl 2-(4-methoxybenzoyl)-2-methylpyrrolidine-1-carboxylate (3ap)

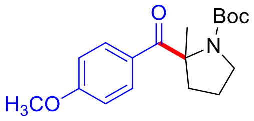

According to the general procedure **A** (0.1 mmol scale, two 427 nm blue LEDs were used), product **3ap** was isolated in 56% yield (17.8 mg) as yellow solid, mp: 87-88 ºC; Mixture of rotamers (ratio: 1/5); ^{1}H NMR (400MHz, $CDCl_3$) δ 7.91-7.78 (2H, m), 6.95-6.80 (2H, m), 3.84 (3H, s), 3.79-3.60 (2H, m), 2.50-2.35 (1H, m), 2.13-2.07 (1H, m), 1.95-1.85 (1H, m), 1.78-1.64 (1H, m), 1.54 (2.5H, s), 1.43 (0.5H, s), 1.26 (1.5H, s), 1.07 (7.5H, s); ^{13}C NMR (100 MHz, $CDCl_3$) δ 198.9 (CO), 162.6 (C), 153.1(CO), 130.7 (CH), 130.4 (C), 127.5 (C), 113.5 (CH), 113.1, 80.3 (C), 79.5 (C), 69.5 (C), 55.4 (CH_3), 47.1 (CH_2), 46.9 (CH_2), 38.6 (CH_2), 37.7 (CH_2), 28.4 (CH_3), 28.3 (CH_3), 27.7 (CH_3), 22.8 (CH_3), 22.6 (CH_2); HRMS (ESI): Found: *m/z* 320.1864. Calcd for $C_{18}H_{26}NO_4$ $(M+H)^+$ 320.1862.

(1R,4S)-tert-butyl3-(4-methoxybenzoyl)-2-azabicyclo[2.2.1]heptane-2-carboxylate (3aq)

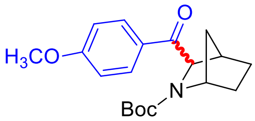

According to the general procedure **A** (0.1 mmol scale, two 427 nm blue LEDs were used), product **3aq** was isolated in 60% yield (19.7 mg) as yellow solid, mp: 172-173 ºC; dr = 1/1; ^{1}H NMR (400MHz, $CDCl_3$) δ 7.99-7.95 (2H, m), 7.95-7.92 (2H, m), 6.97-6.93 (2H, m), 6.93-6.89 (2H, m), 4.73 (1H, s), 4.64 (1H, s), 4.43 (1H, s), 4.29 (1H, s), 3.90-3.80 (7H, m), 2.67-2.58 (2H, m), 1.95-1.88 (2H, m), 1.84-1.75 (2H, m), 1.72-1.63 (3H, m), 1.46 (9H, s), 1.27 (9H, s), 1.23-1.16 (2H, m); ^{13}C NMR (125 MHz, $CDCl_3$) δ 194.4 (CO), 194.0 (CO), 163.47 (C), 163.45 (C), 154.2 (CO), 153.0 (CO), 130.7 (CH), 130.3 (CH), 128.5 (C), 128.2 (C), 113.8(CH), 113.7 (CH), 79.5 (C), 79.5 (C), 66.6 (CH), 66.4 (CH), 57.2 (CH), 56.0 (CH), 55.4 (C), 55.4 (C), 42.9 (CH), 42.3 (CH), 34.8 (CH_2), 34.1 (CH_2), 30.6 (CH_2), 30.6 (CH_2), 28.5 (CH_3), 28.3 (CH_2), 28.2 (CH_3), 28.1 (CH_2); HRMS (ESI): Found: *m/z* 332.1872. Calcd for $C_{19}H_{26}NO_4$ $(M+H)^+$ 332.1862.

t-butyl4-(3-(4-methoxyphenyl)-3-oxo-2-(p-tolyl)propyl)-4-methylpiperidine-1-carboxylate (5a)

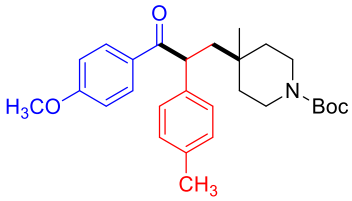

According to the general procedure **C** (0.1 mmol scale), product **5a** was isolated in 90% yield (40.6 mg) as colorless oil; ^{1}H NMR (400 MHz, $CDCl_3$) δ 7.99 (2H, d, J = 8.8 Hz), 7.18 (2H, d, J = 7.9 Hz), 7.07 (2H, d, J = 7.8 Hz), 6.88 (2H, d, J = 8.7 Hz), 4.70-4.60 (1H, m), 3.82 (3H, s), 3.62-3.46 (2H, m), 3.21-3.05 (2H, m), 2.65 (1H, dd, J = 14.1, 8.7 Hz), 2.26 (3H, s), 1.63-1.54 (1H, m), 1.42 (9H, s), 1.36-1.13 (4H, m), 0.92 (3H, s); ^{13}C NMR (100MHz, $CDCl_3$) δ 198.1(CO), 163.3 (C), 154.9 (CO), 138.2 (C), 136.4 (C), 130.9 (CH), 129.6 (CH), 127.8 (CH), 113.8 (CH), 79.2 (C), 55.4 (CH_3), 47.6 (CH), 45.5 (CH_2), 37.0 (CH_2), 32.2 (C), 28.4 (CH_3), 23.7 (CH_3), 20.9 (CH_3); HRMS (ESI): Found: *m/z* 452.2796. Calcd for $C_{28}H_{38}NO_4$ $(M+H)^+$ 452.2801.

t-butyl4-(2,3-bis(4-methoxyphenyl)-3-oxopropyl)-4-methylpiperidine-1-carboxylate (5b)

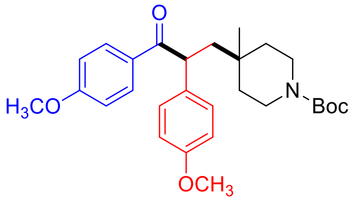

According to the general procedure **C** (0.1 mmol scale), product **5b** was isolated in 77% yield (36.1 mg) as colorless oil; ^{1}H NMR (400 MHz, $CDCl_3$) δ 7.97 (2H, d, J = 8.7 Hz), 7.21 (2H, d, J = 8.4 Hz), 6.88 (2H, d, J = 8.6 Hz), 6.80 (2H, d, J = 8.3 Hz), 4.69-4.60 (1H, m), 3.82 (3H, s), 3.73 (3H, s), 3.61-3.45 (2H, m), 3.20-3.06 (2H, m), 2.61 (1H, dd, J = 14.0, 8.5 Hz), 1.61 (1H, dd, J = 14.2, 2.9 Hz), 1.42 (9H,s), 1.36-1.14 (4H, m), 0.92 (3H, s); ^{13}C NMR (100MHz, $CDCl_3$) δ 198.2 (CO), 163.3 (C), 158.4 (C), 154.9 (CO), 133.2 (C), 130.8 (CH), 129.6 (C), 129.0 (CH), 114.3 (CH), 113.8 (CH), 79.2 (C), 55.4 (CH_3), 55.2 (CH_3), 47.1 (CH), 45.5 (CH_2), 37.0 (CH_2), 32.1 (C), 28.4 (CH_3), 23.7 (CH_3); HRMS (ESI): Found: *m/z* 468.2744. Calcd for $C_{28}H_{38}NO_5$ $(M+H)^+$ 468.2750.

t-butyl4-(2-(4-fluorophenyl)-3-(4-methoxyphenyl)-3-oxopropyl)-4-methylpiperidine-1-carboxylate (5c)

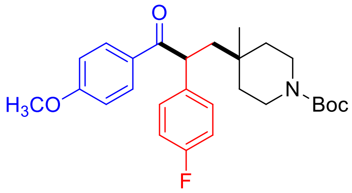

According to the general procedure **C** (0.1 mmol scale), product **5c** was isolated in 85% yield (38.8 mg) as colorless oil; ^{1}H NMR (400 MHz, $CDCl_3$) δ 7.98-7.93 (2H, m), 7.29-7.23 (2H, m), 6.98-6.92 (2H, m), 6.91-6.88 (2H, m), 4.69 (1H, dd, J = 8.5, 3.3 Hz), 3.82 (3H, s), 3.63-3.46 (2H, m), 3.17-3.04 (2H, m), 2.62 (1H, dd, J = 14.1, 8.6 Hz), 1.59 (1H, dd, J = 14.0, 3.2 Hz), 1.42 (9H, s), 1.37-1.13 (4H, m), 0.92 (3H, s); ^{13}C NMR (100MHz, $CDCl_3$) δ 198.0 (CO), 163.5 (C), 161.7 (CF, d, J = 245.7 Hz), 154.9 (CO), 136.9 (C, d, J = 3.2 Hz), 130.8 (CH), 129.5 (CH, d, J = 7.9 Hz), 129.4 (C), 115.8 (CH, d, J = 21.3 Hz), 113.9 (CH), 79.2 (C), 55.4 (CH_3), 47.0 (CH), 45.7 (CH_2), 37.1 (CH_2), 32.2 (C), 28.4 (CH_3), 23.6 (CH_3); ^{19}F NMR (376MHz, $CDCl_3$) δ -115.8 (m); HRMS (ESI): Found: m/z 456.2541. Calcd for $C_{27}H_{35}NO_4F$ $(M+H)^+$ 456.2550.

t-butyl4-(2-(4-chlorophenyl)-3-(4-methoxyphenyl)-3-oxopropyl)-4-methylpiperidine-1-carboxylate (5d)

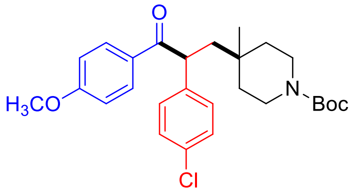

According to the general procedure **C** (0.1 mmol scale), product **5d** was isolated in 92% yield (43.2 mg) as colorless oil; ^{1}H NMR (400 MHz, $CDCl_3$) δ 7.97-7.92 (2H,m), 7.23 (4H,s), 6.92-6.86 (2H,m), 4.67 (1H, dd, J = 8.5, 3.4 Hz), 3.83 (3H, s), 3.63-3.46 (2H,m), 3.17-3.04 (2H,m), 2.63 (1H, dd, J = 14.1, 8.6 Hz), 1.58 (1H, dd, J = 14.2, 3.2 Hz), 1.42 (9H, s), 1.36-1.12 (4H, m), 0.92 (3H, s); ^{13}C NMR (100MHz, $CDCl_3$) δ 197.7 (CO), 163.5 (C), 154.9 (CO), 139.7 (C), 132.7 (C), 130.8 (CH), 129.3 (CH), 129.3 (C), 129.1 (CH), 113.9 (CH), 79.2 (C), 55.4 (CH_3), 47.2 (CH), 45.6 (CH_2), 37.0 (CH_2), 32.2 (CH), 28.4 (CH_3), 23.6 (CH_3); HRMS (ESI): Found: m/z 472.2259. Calcd for $C_{27}H_{35}NO_4Cl$ $(M+H)^+$ 472.2255.

1-(4-methoxyphenyl)-3-(1-methylcyclohexyl)-2-(p-tolyl)propan-1-one (5e)

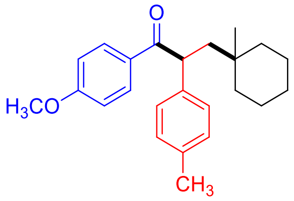

According to the general procedure **C** (0.1 mmol scale), product **5e** was isolated in 64% yield (21.8 mg) as yellow solid, 82-83 °C; ^{1}H NMR (400 MHz, $CDCl_3$) δ 8.04-7.96 (2H, m), 7.23-7.18 (2H, m), 7.09-7.03 (2H, m), 6.92-6.85 (2H, m), 4.68 (1H, dd, *J* = 8.8, 3.0 Hz), 3.82 (3H,s), 2.62 (1H, dd, *J* = 14.1, 8.8 Hz), 2.27 (3H,s), 1.58 (1H, dd, *J* = 14.1, 3.0 Hz), 1.46-1.32 (5H, m), 1.29-1.15 (5H, m), 0.85 (3H,s); ^{13}C NMR (125MHz, $CDCl_3$) δ 198.6 (CO), 163.2 (C), 138.8 (C), 136.1 (C), 130.8 (CH), 129.9 (C), 129.5 (CH), 127.9 (CH), 113.7 (CH), 55.4 (CH_3), 47.7 (CH), 46.4 (CH_2), 38.09 (CH_2), 38.06 (CH_2), 33.6 (C), 26.4 (CH_2), 24.9 (CH_3), 21.95 (CH_2), 21.94 (CH_2), 20.9 (CH_3); HRMS (ESI): Found: *m/z* 373.2130. Calcd for $C_{24}H_{30}O_2Na$ $(M+Na)^+$ 373.2143.

1-(4-methoxyphenyl)-3-(1-methylcyclohexyl)-2-phenylpropan-1-one (5f)

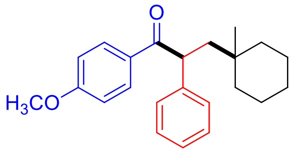

According to the general procedure **C** (0.1 mmol scale), product **5f** was isolated in 89% yield (29.9 mg) as colorless liquid; ^{1}H NMR (400 MHz, $CDCl_3$) δ 8.04-7.96 (2H, m), 7.35-7.29 (2H, m), 7.28-7.22 (2H, m), 7.15 (1H, t, *J* = 7.2 Hz), 6.92-6.84 (2H, m), 4.71 (1H, dd, *J* = 8.7, 2.9 Hz), 3.82 (3H, s), 2.62 (1H, dd, *J* = 14.1, 8.8 Hz), 1.60 (1H, dd, *J* = 14.1, 2.9 Hz), 1.52-1.08 (11H, m), 0.85 (3H, s); ^{13}C NMR (100MHz, $CDCl_3$) δ 198.5 (CO), 163.2 (C), 141.8 (C), 130.8 (CH), 129.9 (C), 128.8 (CH), 128.0 (CH), 126.5 (CH), 113.7 (CH), 55.4 (CH_3), 48.1(CH), 46.4 (CH_2), 38.1 (CH_2), 38.0 (CH_2), 33.6 (C), 26.3 (CH_2), 24.9 (CH_3), 21.94 (CH_2), 21.93 (CH_2); HRMS (ESI): Found: *m/z* 359.1996. Calcd for $C_{23}H_{28}O_2Na$ $(M+Na)^+$ 359.1987.

tert-butyl2-(3-(4-methoxyphenyl)-3-oxo-2-(p-tolyl)propyl)-2-methylpyrrolidine-1-carboxylate (5g)

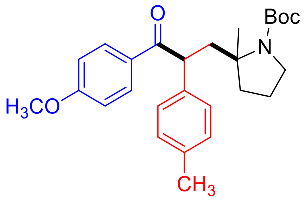

According to the general procedure **C** (0.1 mmol scale), product **5g** was isolated in 80% yield (34.9 mg) as yellow oil; dr = 1.6/1. ^{1}H NMR (500 MHz, $CDCl_3$)δ 8.02-7.91 (2H, m), 7.25-7.18 (1H, m), 7.17-7.11 (1H, m), 7.09-6.99 (2H, m), 6.90-6.80 (2H, m), 4.82-4.46 (1H, m), 3.80 (3H, s), 3.64-3.13 (2H, m), 3.07-2.72 (1H, m), 2.25 (3H, s), 1.84-1.72 (1H, m), 1.70-1.59 (2H, m), 1.56-1.31 (12H, m), 1.30-1.22 (2H, m); ^{13}C NMR (125 MHz, $CDCl_3$) δ 198.7 (CO), 198.5 (CO), 198.4 (CO), 163.4 (C), 163.3 (C), 163.2 (C), 163.1 (C), 154.6 (CO), 154.4 (CO), 153.7 (CO), 153.5 (CO), 138.2 (C), 137.8 (C), 137.7 (C), 136.5 (C), 136.3 (C), 136.1 (C), 131.0 (CH), 130.9 (CH), 129.9 (CH), 129.6 (CH), 129.6 (CH), 129.5 (CH), 128.5 (CH), 128.3 (CH), 128.0 (CH), 113.8 (CH), 113.7 (CH), 113.6 (CH), 79.6 (C), 79.6 (C), 78.5 (C), 78.5 (C), 62.9 (C), 62.6 (C), 55.4 (CH_3), 49.0 (CH), 48.7 (CH_2), 48.6 (CH_2), 48.5 (CH_2), 48.4 (CH_2), 48.3 (CH_2), 42.4 (CH_2), 42.1 (CH_2), 39.5 (CH_2), 39.4 (CH_2), 39.2 (CH_2), 28.6 (CH_3), 28.5 (CH_3), 28.5 (CH_3), 26.7 (CH_3), 26.6 (CH_3), 25.5 (CH_3), 25.0 (CH_3), 21.8 (CH_2), 21.7 (CH_2), 21.6 (CH_2), 21.4 (CH_2), 20.99 (CH_3), 20.9 (CH_3); HRMS (ESI): Found: *m/z* 438.2645. Calcd for $C_{27}H_{36}NO_4$ $(M+H)^+$ 438.2644.

tert-butyl 2-(3-oxo-3-phenyl-2-(p-tolyl)propyl)pyrrolidine-1-carboxylate (5h)

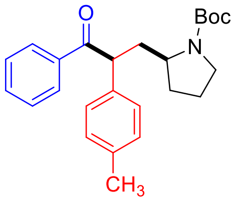

According to the general procedure **C** (2 mmol scale), product **5h** was isolated in 73% yield (537.7 mg), dr = 1.4/1.

Diasteromer 1: Colorless oil; Mixture of rotamers (ratio; 40/60); ^{1}H NMR (400 MHz, $CDCl_3$) δ 8.03-7.89 (2H, m), 7.49-7.29 (3H, m), 7.21 (2H,s,br), 7.12-7.02 (2H, m), 4.95 (0.6H,s,br), 4.60 (0.4 H, s, br), 4.07 (0.6H, s,br), 3.79 (0.4 H, s, br), 3.46-3.11 (2H, m),

2.83-2.55 (1H, m), 2.26 (3H, s), 2.04-1.66 (4H, m), 1.49 (4H, s, br), 1.27 (6H, s); ^{13}C NMR (100MHz, $CDCl_3$) δ 199.2 (CO), 155.0 (CH_2), 137.2 (C), 137.1 (C), 136.6 (C), 136.4 (C), 132.5 (CH), 129.6 (CH), 128.9 (CH), 128.3 (CH), 128.1 (CH), 79.5 (C), 78.8 (C), 56.1 (CH), 50.5 (CH), 46.0 (CH_2), 39.6 (C), 39.2 (C), 31.2 (CH_2), 28.3 (CH_3), 23.6 (CH_2), 23.0 (CH_2), 21.0 (CH_3); HRMS (ESI): Found: *m/z* 394.2386. Calcd for $C_{25}H_{32}NO_3$ $(M+H)^+$ 394.2382.

Diasteromer 2: White solid, mp: 93-94 °C; Mixture of rotamers (ratio; 40/60); ^{1}H NMR (400 MHz, $CDCl_3$) δ 8.03-7.89 (2H, m), 7.50-7.30 (3H, m), 7.20-7.12 (2H, m), 7.12-7.02 (2H, m), 4.70 (0.4H,s,br), 4.51 (0.6H,s,br), 3.92 (0.4H, s, br), 3.68 (0.6H, s, br), 3.54-3.17 (2H, m), 2.26 (3H, s), 2.13-1.62 (6H, m), 1.49-1.16 (9H, m); ^{13}C NMR (100MHz, $CDCl_3$) δ 199.4 (CO), 199.0 (CO), 154.8 (CO), 137.0 (C), 136.7 (C), 136.5 (C), 136.0 (C), 132.8 (CH), 132.4 (CH), 129.7 (CH), 128.8 (CH), 128.4 (CH), 128.1 (CH), 79.4 (C), 78.8 (C), 55.6 (CH), 50.5 (CH), 46.3 (CH_2), 45.7 (CH_2), 39.1 (C), 37.8 (C), 30.7 (CH_2), 28.4 (CH_2), 23.7 (CH_2), 22.8 (CH_2), 21.0 (CH_2); HRMS (ESI): Found: *m/z* 394.2377. Calcd for $C_{25}H_{32}NO_3$ $(M+H)^+$ 394.2382.

tert-butyl 2-(3-oxo-3-phenyl-2-(p-tolyl)propyl)azetidine-1-carboxylate (5i)

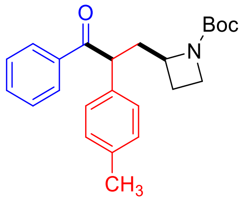

According to the general procedure **C** (0.3 mmol scale), product **5i** was isolated in 50% yield (56.9 mg), dr = 1.4/1;

Diasteromer 1: Colorless oil; ^{1}H NMR (400 MHz, $CDCl_3$) δ 8.01-7.92 (2H, m), 7.45 (1H, t, J = 7.3 Hz), 7.41-7.33 (2H, m), 7.31-7.22 (2H, m), 7.12-7.05 (2H, m), 4.89 (1H, s, br), 4.29-4.18 (1H, m), 3.79-3.68 (2H, m), 2.77 (1H, s, br), 2.27 (3H, s), 2.19-1.97 (2H, m), 1.69-1.58 (1H, m), 1.44 (9H, s); ^{13}C NMR (100MHz, $CDCl_3$) δ 199.6 (CO), 156.8 (CO), 136.6 (C), 136.5 (C), 136.4 (C), 132.7 (CH), 129.6 (CH), 128.8 (CH), 128.4 (CH), 128.3 (CH), 79.2 (C), 60.4 (CH), 49.5 (CH), 46.6 (CH_2), 40.2 (CH_2), 28.4 (CH_3), 22.5 (CH_2), 21.0 (CH_3); HRMS (ESI): Found: *m/z* 380.2231. Calcd for $C_{24}H_{30}NO_3$ $(M+H)^+$ 380.2226.

Diasteromer2: Colorless oil; ^{1}H NMR (500 MHz, $CDCl_3$)δ 7.99-7.92 (2H, m), 7.45 (1H, t, J = 7.3 Hz), 7.39-7.33 (2H, m), 7.19-7.14 (2H, m), 7.11-7.05 (2H, m), 4.71 (1H, s, br), 4.10 (1H, s, br), 3.78 (2H, t, J = 7.6 Hz), 2.55-2.32 (2H, m), 2.31-2.21 (4H, m), 1.90-1.80 (1H, m), 1.38 (9H, s); ^{13}C NMR (125MHz, $CDCl_3$) δ 199.3 (CO), 156.8 (CO), 136.7 (C), 136.4 (C), 132.7 (CH), 129.7 (CH), 128.8 (CH), 128.4 (CH), 128.0 (CH), 79.2 (C), 60.6 (CH), 49.5 (CH), 46.4 (CH_2), 39.9 (CH_2), 28.3 (CH_3), 22.4 (CH_2), 21.0 (CH_3); HRMS (ESI): Found: *m/z* 380.2231. Calcd for $C_{24}H_{30}NO_3$ $(M+H)^+$ 380.2226.

tert-butyl 2-(3-oxo-3-phenyl-2-(p-tolyl)propyl)piperidine-1-carboxylate (5j)

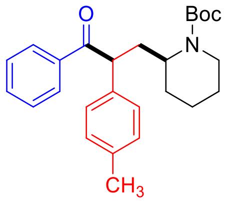

According to the general procedure **C** (0.3 mmol scale), product **5j** was isolated in 64% yield (77.8 mg), dr = 1.9/1;

Diasteromer 1: Colorless oil; ^{1}H NMR (400 MHz, $CDCl_3$) δ 7.98-7.89 (2H, m), 7.44 (1H, t, J = 7.3 Hz), 7.39-7.32 (2H, m), 7.25-7.19 (2H, m), 7.11-7.04 (2H, m), 4.62(1H, s, br), 4.37 (1H, s, br), 3.83 (1H, s, br), 3.16-2.64 (2H, m), 2.26 (3H, s), 1.72-1.51 (5H, m), 1.47-1.15 (11H, m); ^{13}C NMR (100MHz, $CDCl_3$) δ 199.2 (CO), 155.2 (CO), 136.7 (C), 136.6 (C), 132.6 (CH), 129.6 (CH), 128.8 (CH), 128.4 (CH), 128.1 (CH), 79.1 (C), 50.6 (CH), 49.8 (CH), 39.2 (CH_2), 34.8 (CH_2), 29.7 (CH_2), 28.2 (CH_3), 25.6 (CH_2), 21.0 (CH_3), 19.4 (CH_2); HRMS (ESI): Found: *m/z* 408.2544. Calcd for $C_{26}H_{34}NO_3$ $(M+H)^+$ 408.2539.

Diasteromer 2: Colorless oil; ^{1}H NMR (400 MHz, $CDCl_3$) δ 8.02-7.90 (2H, m), 7.49-7.39 (1H, m), 7.38-7.29 (2H, m), 7.18-7.03 (4H, m), 4.46 (1H, dd, J = 10.3, 2.9 Hz), 4.28 (1H, s, br), 4.13 (1H, s, br), 2.78 (1H, t, J = 12.7 Hz), 2.44-2.30 (1H, m), 2.26 (3H, s), 2.14-2.02 (1H, m), 1.66-1.50 (5H, m), 1.44-0.95 (10H, m); ^{13}C NMR (100MHz, $CDCl_3$) δ 198.5 (CO), 155.1 (CO), 137.2 (C), 136.6 (C), 132.7 (C), 129.8 (CH), 128.9 (CH), 128.3 (CH), 127.8 (CH), 79.3 (C), 49.5 (CH), 38.1 (CH_2), 34.0 (CH_2), 29.4 (CH_2), 28.0 (CH_3), 25.6 (CH_2), 20.9 (CH_3), 19.0 (CH_2); HRMS (ESI): Found: *m/z* 408.2538. Calcd for $C_{26}H_{34}NO_3$ $(M+H)^+$ 408.2539.

tert-butyl4-methyl-4-(3-oxo-3-(thiophen-2-yl)-2-(p-tolyl)propyl)piperidine-1-carboxylate (5k)

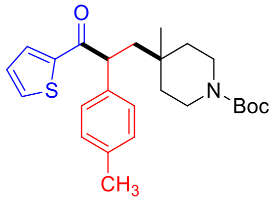

According to the general procedure **C** (0.1 mmol scale), product **5k** was isolated in 67% yield (28.5 mg) as white solid, mp:158-159 °C; ^{1}H NMR (400 MHz, $CDCl_3$) δ 7.77 (1H, dd, J = 3.8, 0.9 Hz), 7.57 (1H, dd, J = 4.9, 1.0 Hz), 7.24-7.19 (2H, m), 7.11-7.06 (3H, m), 4.49 (1H, dd, J = 8.5, 3.5 Hz), 3.63-3.46 (2H,m), 3.18-3.07 (2H,m), 2.61 (1H, dd, J = 14.2, 8.6 Hz), 2.28 (3H,s), 1.63 (1H, dd, J = 14.2, 3.4 Hz), 1.43 (9H, s), 1.36-1.29 (2H, m), 1.28-1.17 (2H,m), 0.94 (3H, s); ^{13}C NMR (100MHz, $CDCl_3$) δ 192.5 (CO), 154.9 (CO), 143.8 (C), 137.8 (C), 136.7 (C), 133.7 (CH), 132.0 (CH), 129.6 (CH), 128.2 (CH), 127.9 (CH), 79.2 (C), 49.9 (CH), 45.2 (CH_2), 37.0 (CH_2), 32.2 (C), 28.4 (CH_2), 23.6 (CH_2), 21.0 (CH_2); HRMS (ESI): Found: *m/z* 428.2264. Calcd for $C_{25}H_{34}NO_3S$ $(M+H)^+$ 428.2259.

tert-butyl4-(3-(1-ethyl-7-methyl-4-oxo-1,4-dihydro-1,8-naphthyridin-3-yl)-3-oxo-2-(p-tolyl)propyl)-4-methylpiperidine-1-carboxylate (5l)

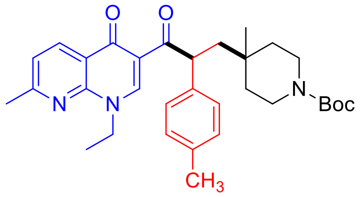

According to the general procedure **C** (0.1 mmol scale), product **5l** was isolated in 86% yield (45.8 mg) as yellowoil; ^{1}H NMR (400 MHz, $CDCl_3$) δ 8.65 (1H, d, J = 8.1 Hz), 8.52 (1H, s), 7.29 (2H, d, J = 7.9 Hz), 7.23 (1H, d, J = 8.1 Hz), 7.03 (2H, d, J = 7.9 Hz), 5.92 (1H, dd, J = 8.9, 2.9 Hz), 4.49-4.32 (2H, m), 3.61-3.46 (2H, m), 3.21-3.08 (2H, m), 2.73-2.60 (4H, m), 2.24 (3H, s), 1.59 (1H, dd, J = 13.9, 2.9 Hz), 1.47-1.38 (14H, m), 1.32-1.21 (2H, m), 0.99 (3H, s); ^{13}C NMR (100MHz, $CDCl_3$) δ 199.8 (CO), 175.7 (CO), 162.7 (CH_3), 154.9 (CO), 149.0 (CH), 148.6 (C), 138.6 (C), 136.8 (CH), 136.2 (C), 129.2 (CH), 128.5 (CH), 122.0 (C), 121.1 (CH), 118.2 (C), 79.0 (C), 49.2 (CH), 46.7 (CH), 45.1 (CH_2), 37.1 (CH_2), 37.1 (CH_2), 32.3 (C), 28.4 (CH_3), 25.0 (CH_3), 23.7 (CH_3), 21.0 (CH_3), 15.1 (CH_3); HRMS (ESI): Found: *m/z* 532.3171. Calcd for

$C_{32}H_{42}N_3O_4$ $(M+H)^+$ 532.3175.

2-(3-benzoylphenyl)-1-(4-methoxyphenyl)propan-1-one (6a)

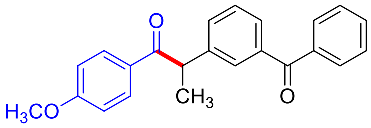

According to the general procedure **B** (0.1 mmol scale), product **6a** was isolated in 62% yield (21.3 mg) as colorless oil; ^1H NMR (400 MHz, $CDCl_3$) δ 7.98-7.91 (2H,m), 7.78-7.69 (3H, m), 7.64-7.55 (2H, m), 7.54-7.50 (1H, m), 7.45 (2H, t, *J* = 7.7 Hz), 7.40 (1H, t, *J* = 7.7 Hz), 6.92-6.85 (2H, m), 4.74 (1H, q, *J* = 6.9 Hz), 3.83 (3H, s), 1.55 (3H, d, *J* = 6.9 Hz); ^{13}C NMR (100MHz, $CDCl_3$) δ 198.4 (CO), 196.5 (CO), 163.4 (C), 142.1 (C), 138.1 (C), 137.4 (C), 132.5 (CH), 131.6 (CH), 131.05 (CH), 130.0 (CH), 129.5 (CH), 129.1 (C), 128.8 (CH), 128.7 (CH), 128.3 (CH), 113.8 (CH), 55.4 (CH_3), 47.1 (CH), 19.4 (CH_3); HRMS (ESI): Found: *m/z* 367.1320. Calcd for $C_{23}H_{20}O_3Na$ $(M+Na)^+$ 367.1310.

2-(2-fluoro-[1,1'-biphenyl]-4-yl)-1-(4-methoxyphenyl)propan-1-one (6b)

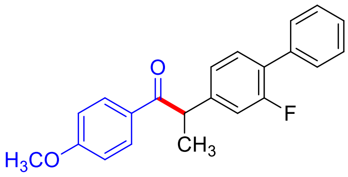

According to the general procedure **B** (0.1 mmol scale), product **6b** was isolated in 56% yield (18.7 mg) as colorless oil; ^1H NMR (400 MHz, $CDCl_3$) δ8.02-7.94 (2H,m), 7.54-7.47 (2H,m), 7.44-7.39 (2H,m), 7.38-7.32 (2H,m), 7.17-7.08 (2H,m), 6.94-6.87 (2H,m), 4.70 (1H, q, *J* = 6.9 Hz), 3.84 (3H,s), 1.56 (3H, d, *J* = 6.9 Hz); ^{13}C NMR (100MHz, $CDCl_3$) δ 198.2 (CO), 163.4 (C), 159.9 (CF, d, *J* =247.1 Hz), 143.2 (C, d, *J* = 7.5 Hz), 135.4 (C, d, *J* = 1.1 Hz), 131.1 (CH), 131.0 (CH), 129.2 (C), 128.9 (CH, d, *J* = 2.8 Hz), 128.4 (CH), 127.6 (CH), 127.5 (C, d, *J* = 13.5 Hz), 123.7 (CH, d, *J* = 3.3 Hz), 115.4 (CH, d, *J* = 23.3 Hz), 113.8 (CH), 55.4 (CH), 46.7 (CH_3), 19.4 (CH_3); ^{19}F NMR (376MHz, $CDCl_3$) δ -117.2 (m); HRMS (ESI): Found: *m/z* 357.1263. Calcd for $C_{22}H_{19}O_2FNa$ $(M+Na)^+$ 357.1267.

2-(6-methoxynaphthalen-2-yl)-1-(4-methoxyphenyl)propan-1-one (6c)

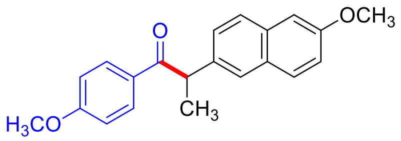

According to the general procedure **B** (0.1 mmol scale), product **6c** was isolated in 94% yield (30.2 mg) as colorless oil; ^{1}H NMR (400MHz, $CDCl_3$) δ 8.03-7.95 (2H,m), 7.72-7.61 (3H,m), 7.43-7.36 (1H,m), 7.12 (1H, dd, *J* = 9.0, 2.4 Hz), 7.08 (1H, d, *J* = 2.1 Hz), 6.88-6.80 (2H, m), 4.77 (1H, q, *J* = 6.8 Hz), 3.88 (3H, s), 3.78 (3H, s), 1.59 (3H, d, *J* = 6.8 Hz); ^{13}C NMR (100MHz, $CDCl_3$) δ 198.9 (CO), 163.1 (C), 157.5 (C), 137.1 (C), 133.4 (C), 131.0 (CH), 129.5 (C), 129.2 (CH), 127.5 (CH), 126.4 (CH), 126.1 (CH), 118.9 (CH), 113.6 (CH), 105.5 (CH), 55.3 (CH_3), 55.3 (CH_3), 47.5 (CH), 19.5 (CH_3); HRMS (ESI): Found: *m/z* 321.1501.Calcd for $C_{21}H_{21}O_3$ $(M+H)^+$ 321.1491.

2-(4-(1-(4-methoxyphenyl)-1-oxopropan-2-yl)phenyl)isoindolin-1-one (6d)

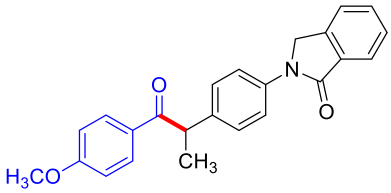

According to the general procedure **B** (0.1 mmol scale), product **6d** was isolated in 54% yield (19.9 mg) as yellow solid, mp: 164-165 °C; ^{1}H NMR (400MHz, $CDCl_3$) δ 7.99-7.93 (2H,m), 7.91-7.87 (1H,m), 7.82-7.75 (2H,m), 7.61-7.54 (1H,m), 7.52-7.44 (2H,m), 7.37-7.30 (2H,m), 6.89-6.82 (2H, m), 4.79 (2H, s), 4.66 (1H, q, *J* = 6.8 Hz), 3.81 (3H, s), 1.52 (3H, d, *J* = 6.8 Hz); ^{13}C NMR (125 MHz, $CDCl_3$) δ 198.7 (CO), 167.4 (CON), 163.2 (C), 140.0 (C), 138.1 (C), 137.9 (C), 133.1 (C), 132.0 (CH), 131.0 (CH), 129.3 (C), 128.4 (CH), 128.3 (CH), 124.1 (CH), 122.6 (CH), 119.9 (CH), 113.7 (CH), 55.4 (CH_3), 50.6 (CH_2), 46.8 (CH), 19.4 (CH_3); HRMS (ESI): Found: *m/z* 394.1410. Calcd for $C_{24}H_{21}NO_3Na$ $(M+Na)^+$ 394.1419.

3-(2-(3-benzoylphenyl)propanoyl)-1-ethyl-7-methyl-1,8-naphthyridin-4(1H)-one (6e)

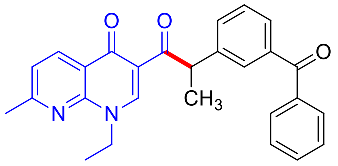

According to the general procedure **A** (0.1 mmol scale), product **6e** was isolated in 78% yield (33.2 mg) as white solid; mp: 133-134 °C; ^{1}H NMR (400 MHz, $CDCl_3$) δ 8.63-8.56 (2H, m), 7.81 (1H, s), 7.79-7.73 (2H, m), 7.65 (1H, d, *J* = 7.7 Hz), 7.61 (1H, d, *J* = 7.7 Hz), 7.55 (1H, t, *J* = 7.4 Hz), 7.43 (2H, t, *J* = 7.6 Hz), 7.38 (1H, t, *J* = 7.7 Hz), 7.23 (1H, d, *J* = 8.1 Hz), 5.61 (1H, q, *J* = 7.0 Hz), 4.54-4.34 (2H, m), 2.64 (3H, s), 1.53 (3H, d, *J* = 7.0 Hz), 1.45 (3H, t, *J* = 7.2 Hz); ^{13}C NMR (100MHz, $CDCl_3$) δ 199.8 (CO), 196.7 (CO), 175.4 (CO), 162.8 (C), 148.7 (CH), 148.6 (C), 141.8 (C), 137.6 (C), 137.5 (C), 136.7 (CH), 132.9 (CH), 132.3 (CH), 130.2 (CH), 130.1 (CH), 128.3 (CH), 128.2 (CH), 128.1 (CH), 121.9 (C), 121.2 (CH), 118.4 (C), 49.3, 46.7, 25.0, 18.5 (CH_3), 15.1 (CH_3); HRMS (ESI): Found: *m/z* 447.1691.Calcd for $C_{27}H_{24}N_2O_3Na$ $(M+Na)^+$ 447.1685.

1-ethyl-3-(2-(2-fluoro-[1,1'-biphenyl]-4-yl)propanoyl)-7-methyl-1,8-naphthyridin-4(1H)-one (6f)

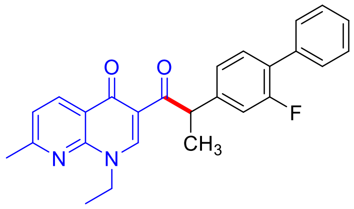

According to the general procedure **B** (0.1 mmol scale), product **6f** was isolated in 52% yield (21.5 mg) as yellow solid; mp: 127-128 °C; ^{1}H NMR (400 MHz, $CDCl_3$) δ8.67-8.59 (2H, m), 7.53-7.45 (2H, m), 7.42-7.36 (2H, m), 7.36-7.30 (2H, m), 7.30-7.19 (3H, m), 5.65 (1H, q, *J* = 7.0 Hz), 4.56-4.37 (2H, m), 2.65 (3H, s), 1.55 (3H, d, *J* = 7.0 Hz), 1.47 (3H, t, *J* = 7.2 Hz); ^{13}C NMR (100MHz, $CDCl_3$) δ 199.8 (CO), 175.6 (CO), 162.8 (C), 159.6 (CF, d, *J* = 247.6 Hz), 148.8 (CH), 148.6 (C), 143.1 (C, d, *J* = 7.7 Hz), 136.8 (CH), 135.8 (C), 130.4 (CH, d, *J* = 3.9 Hz), 128.9 (CH, d, *J* = 3.0 Hz), 128.3 (CH), 127.3 (CH), 127.0 (CH, d, *J* = 13.5 Hz), 124.6 (CH, d, *J* = 3.2 Hz), 121.9 (C), 121.2 (CH), 118.4 (C), 116.0 (CH, d, *J* = 23.3 Hz), 48.8, 46.8, 25.0, 18.4 (CH_3), 15.1 (CH_3); ^{19}F NMR (376MHz, $CDCl_3$) δ -118.3 (m); HRMS (ESI): Found: *m/z* 415.1808. Calcd for $C_{26}H_{24}N_2O_2F$ $(M+H)^+$ 415.1822.

1-ethyl-7-methyl-3-(2-(4-(1-oxoisoindolin-2-yl)phenyl)propanoyl)-1,8-naphthyridin-4(1H)-one (6g)

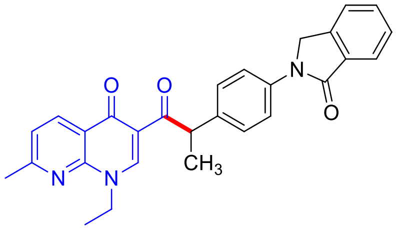

According to the general procedure **B** (0.1 mmol scale), product **6g** was isolated in 65% yield (29.6 mg) as white solid; mp: 178-179 ºC; ^{1}H NMR (400 MHz, $CDCl_3$) δ 8.60 (1H, d, J = 8.1 Hz), 8.56 (1H, s), 7.90-7.84 (1H, m), 7.77-7.70 (2H, m), 7.58-7.52 (1H, m), 7.50-7.42 (4H, m), 7.21 (1H, d, J = 8.1 Hz), 5.59 (1H, q, J = 6.9 Hz), 4.78 (2H, s), 4.51-4.36 (2H, m), 2.62 (3H, s), 1.52 (3H,d, J = 7.0 Hz), 1.45 (3H,t, J = 7.2 Hz); ^{13}C NMR (125MHz, $CDCl_3$) δ 200.2 (CO), 175.5 (CO), 167.3 (CON), 162.7 (C), 148.6 (C and CH overlaped), 140.1 (C), 137.9 (C), 137.8 (C), 136.7 (CH), 133.2 (C), 131.9 (CH), 129.3 (CH), 128.3 (CH), 124.0 (CH), 122.5 (CH), 121.9 (C), 121.1 (CH), 119.6 (CH), 118.7 (C), 50.7, 48.8, 46.7, 25.0, 18.4, 15.1; HRMS (ESI): Found: m/z 452.1979. Calcd for $C_{28}H_{26}N_3O_3$ $(M+H)^+$ 452.1974.

(5S,8R,9S,10S,13R,14S,17R)-17-((R)-6-(6-methoxynaphthalen-2-yl)-5-oxoheptan-2-yl)-10,13-dimethyldecahydro-1H-cyclopenta[a]phenanthrene-3,7,12(2H,4H,8H)-trione (6h)

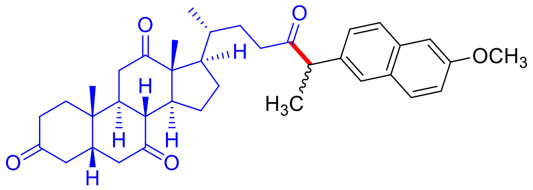

According to the general procedure **B** (0.1 mmol scale, DMSO was used as solvent; 1.2 equiv Cs_2CO_3 was used; two 440 nm blue LEDs was used as light source), product **6h** was isolated in 65% yield (41.1 mg) as yellow solid, dr = 1.2:1, mp:188-189 °C; ^{1}H NMR (500 MHz, $CDCl_3$) δ 7.72-7.65 (2H, m), 7.59 (1H, d, J = 5.6 Hz), 7.30-7.26 (1H, m), 7.16-7.07 (2H, m), 3.93-3.83 (4H, m), 2.94-2.71 (3H, m), 2.50-2.16 (7H, m), 2.14-2.03 (2H, m), 2.00 (1H, d, J = 13.2 Hz), 1.95-1.88 (2H, m), 1.88-1.65 (4H, m), 1.59 (1H, td, J = 14.2, 5.1 Hz), 1.45 (1.46 H, d, J = 2.8 Hz), 1.44 (1.46 H, d, J = 2.8 Hz),1.36 (3H, s), 1.28-1.11 (4H, m), 0.97 (1.46 H, s), 0.93 (1.54 H, s), 0.69 (1.46 H, d, J = 6.3 Hz), 0.56 (1.56 H, d, J = 6.6 Hz); ^{13}C NMR (125 MHz, $CDCl_3$) δ 211.9 (CO), 211.9

(CO), 211.5 (CO), 211.3 (CO), 209.0 (CO), 208.7 (CO), 208.6 (CO), 157.6 (C), 135.8 (C), 135.7 (C), 133.62 (C), 133.61 (C), 129.14 (C), 129.12 (CH), 129.1 (CH), 127.44 (CH), 127.42 (CH), 126.43 (CH), 126.41 (CH), 126.3 (CH), 119.0 (CH), 105.6 (CH), 105.6 (CH), 56.8 (C), 56.7 (C), 55.3 (CH_3), 53.1 (CH), 52.9 (CH), 51.7 (CH), 51.6 (CH), 48.9 (CH), 48.89 (CH_3), 46.8 (CH), 45.6 (CH), 45.5 (CH), 45.4 (CH), 45.3 (CH), 44.9 (CH_2), 42.7 (CH_2), 38.5 (CH_2), 38.2 (CH_2), 37.8 (CH_2), 36.4 (CH_2), 35.9 (C), 35.2 (CH_2), 35.2 (CH), 35.1 (CH), 29.4 (CH_2), 29.2 (CH_2), 27.5 (CH_2), 27.4 (CH_2), 25.0 (CH_2), 24.99 (CH_2), 21.8 (CH_3), 18.7 (CH_3), 18.5 (CH_3), 17.44 (CH_3), 17.42 (CH_3), 11.7 (CH_3), 11.69 (CH_3); HRMS (ESI): Found: *m/z* 571.3431. Calcd for $C_{37}H_{47}O_5$ $(M+H)^+$ 571.3423.

2-(4-(1-(6-(3-((3r,5r,7r)-adamantan-1-yl)-4-methoxyphenyl)naphthalen-2-yl)-1-oxopropan-2-yl)phenyl)isoindolin-1-one (6i)

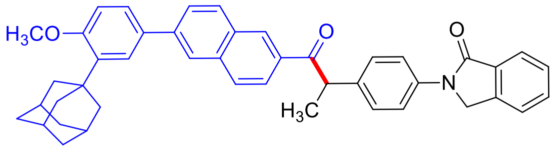

According to the general procedure **B** (0.05 mmol scale), product **6i** was isolated in 41% yield (12.9 mg) as yellow sold, mp: 226-227 °C; ^{1}H NMR (400 MHz, $CDCl_3$) δ 8.50 (1H, s), 8.04 (1H, dd, *J* = 8.6, 1.3 Hz), 7.98-7.93 (2H,m), 7.92-7.84 (2H,m), 7.84-7.79 (2H,m), 7.76 (1H, dd, *J* = 8.6, 1.4 Hz), 7.59-7.54 (2H,m), 7.53-7.45 (3H,m), 7.45-7.38 (2H,m), 6.98 (1H, d, *J* = 8.5 Hz), 4.89 (1H, q, *J* = 6.8 Hz), 4.79 (2H,s), 3.89 (3H,s), 2.17 (6H, s, br), 2.10 (3H, s, br), 1.80 (6H, s, br), 1.62 (3H, d, *J* = 6.8 Hz); ^{13}C NMR (100MHz, $CDCl_3$) δ 200.1 (CO), 167.4 (C), 158.9 (C), 141.6 (C), 140.0 (C), 139.0 (C), 138.3 (C), 137.7 (C), 135.8 (C), 133.3 (C), 133.1 (C), 132.4 (C), 132.0 (CH), 131.2 (C), 130.2 (CH), 130.0 (CH), 128.5 (CH), 128.4 (CH), 128.4 (CH), 126.5 (CH), 125.9 (CH), 125.7 (CH), 124.9 (CH), 124.6 (CH), 124.1 (CH), 122.6 (CH), 120.0 (CH), 112.1 (CH), 55.1 (CH_3), 50.6 (CH_2), 47.2 (CH), 40.6 (CH), 37.2 (C), 37.1 (CH), 29.1 (CH), 19.5 (CH_3); HRMS (ESI): Found: *m/z* 632.3138. Calcd for $C_{44}H_{42}NO_3$ $(M+H)^+$ 632.3165.

6i could also be obtained according to general procedure **A** in 56% yield when acyl imidazole was used as starting material.

1-(6-(3-((3r,5r,7r)-adamantan-1-yl)-4-methoxyphenyl)naphthalen-2-yl)-2-(2-fluoro-[1,1'-biphenyl]-4-yl)propan-1-one (6j)

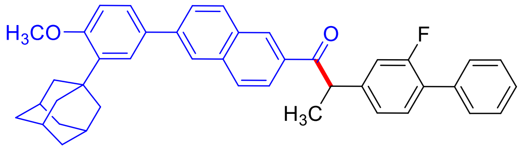

According to the general procedure **A** (0.05 mmol scale), product **6j** was isolated in 53% yield (15.7 mg) as white solid, mp:192-193 °C; ^{1}H NMR (400 MHz, $CDCl_3$) δ 8.52 (1H, s), 8.06 (1H, d, *J* = 8.6 Hz), 8.02-7.94 (2H,m), 7.90 (1H, d, *J* = 8.7 Hz), 7.79 (1H, d, *J* = 9.6 Hz), 7.59 (1H, d, *J* = 2.0 Hz), 7.57-7.47 (3H, m), 7.45-7.30 (4H, m), 7.24-7.15 (2H, m), 6.99 (1H, d, *J* = 8.4 Hz), 4.92 (1H, q, *J* = 6.8 Hz), 3.90 (3H, s), 2.18 (6H, s, br), 2.11 (3H, s, br), 1.81 (6H, s, br), 1.65 (3H, d, *J* = 6.8 Hz); ^{13}C NMR (100MHz, $CDCl_3$) δ 199.6 (CO), 159.9 (CF, d, *J* = 250.0 Hz), 159.0 (C), 142.9 (C, d, *J* = 8.0 Hz), 141.7 (C), 139.0 (C), 136.0 (C), 135.4 (C), 133.2 (C), 132.4 (C), 131.17 (CH), 131.13 (C), 130.2 (CH), 130.0 (CH), 128.9 (CH, d, *J* = 2.8 Hz), 128.6 (CH), 128.4 (CH), 127.6 (C, d, *J* = 13.5 Hz), 127.59 (CH), 126.6 (CH), 125.9 (CH), 125.7 (CH), 124.8 (CH), 124.6 (CH), 123.8 (CH, d, *J* = 3.2 Hz), 115.5 (CH, d, *J* = 23.3 Hz), 112.1 (CH), 55.2 (CH_3), 47.1 (CH), 40.6 (CH), 37.2 (C), 37.1 (CH), 29.1 (CH), 19.4 (CH_3); ^{19}F NMR (376MHz, $CDCl_3$) δ -117.1 (m); HRMS (ESI): Found: *m/z* 595.3004. Calcd for $C_{42}H_{40}O_2F$ $(M+H)^+$ 595.3012.

1-(6-(3-((3r,5r,7r)-adamantan-1-yl)-4-methoxyphenyl)naphthalen-2-yl)-2-(4-isobutylphenyl)propan-1-one (6k)

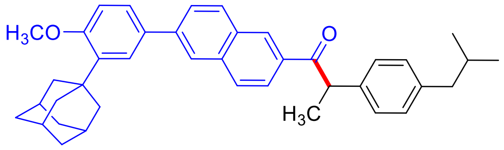

According to the general procedure **A** (0.05 mmol scale), product **6k** was isolated in 51% yield (14.1 mg) as yellow solid, mp: 137-138 °C; ^{1}H NMR (500 MHz, $CDCl_3$) δ 8.50 (1H, s), 8.04 (1H, dd, *J* = 8.7, 1.6 Hz), 7.97-7.92 (2H,m), 7.86 (1H, d, *J* = 8.7 Hz), 7.77 (1H, dd, *J* = 8.6, 1.6 Hz), 7.58 (1H, d, *J* = 2.2 Hz), 7.53 (1H, dd, *J* = 8.4, 2.2 Hz), 7.26-7.24 (2H, m), 7.08 (2H, d, *J* = 8.2 Hz), 6.99 (1H, d, *J* = 8.5 Hz), 4.85 (1H, q, *J* =6.8 Hz), 3.90 (3H, s), 2.41 (2H, d, *J* = 7.1 Hz), 2.21-2.16 (6H, m), 2.13-2.08 (3H, m),

1.84-1.77 (7H, m), 1.60 (3H, d, J = 6.9 Hz), 0.86 (6H, d, J = 6.6 Hz); ^{13}C NMR (125 MHz, $CDCl_3$) δ 200.4 (CO), 158.9 (C), 141.5 (C), 140.3 (C), 139.0 (C), 138.8 (C), 135.8 (C), 133.5 (C), 132.5 (C), 131.2 (C), 130.2 (CH), 130.0 (CH), 129.7 (CH), 128.3 (CH), 127.5 (CH), 126.4 (CH), 125.9 (CH), 125.7 (CH), 125.0 (CH), 124.6 (CH), 112.1 (CH), 55.1 (CH_3), 47.4 (CH), 45.0 (CH_2), 40.6 (CH_2), 37.2 (CH), 37.1 (CH_2), 30.1 (CH), 29.1 (CH_2), 22.37 (CH_3), 22.35 (CH_3), 19.6 (CH_3); HRMS (ESI): Found: *m/z* 579.3235. Calcd for $C_{40}H_{44}O_2Na$ $(M+Na)^+$ 579.3239.

2-(2-fluoro-[1,1'-biphenyl]-4-yl)-4-(6-methoxynaphthalen-2-yl)pentan-3-one (6l)

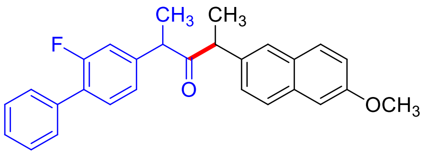

According to the general procedure **B** (0.1 mmol scale), product **6l** was isolated in 65% yield (24.9 mg) as colorless oil, dr =1.5/1; ^{1}H NMR (400 MHz, $CDCl_3$) δ 7.61-7.54 (2H, m), 7.44-7.30 (6H, m), 7.14-7.03 (4H, m), 6.76 (1H, dd, J = 11.8, 1.4 Hz), 6.70 (1H, dd, J = 7.9,1.4 Hz), 4.04 (1H, q, J = 7.0 Hz), 3.93 (1H, q, J =7.0 Hz),3.91 (3H, s), 1.47 (3H, d, J = 7.0 Hz), 1.42 (3H, d, J = 7.1 Hz); ^{13}C NMR (100MHz, $CDCl_3$) δ 210.8 (CO), 159.4 (CF, d, J = 248.3 Hz), 157.6 (C), 141.7 (C, d, J = 7.7 Hz), 135.5 (C), 135.0 (C), 133.6 (C), 130.3 (CH, d, J = 4.0 Hz), 129.1 (CH), 128.88 (C and CH overlaped), 128.86 (CH), 128.3 (CH), 127.5 (CH), 127.3 (C, d, J = 13.5 Hz), 127.2 (CH), 126.9 (CH), 126.5 (CH), 123.9 (CH, d, J = 3.3 Hz), 119.0 (CH), 115.6 (CH, d, J = 23.5 Hz), 105.6 (CH), 55.3 (CH_3), 52.2 (CH), 50.9 (CH), 18.4 (CH_3), 18.2 (CH_3). ^{19}F NMR (376MHz, $CDCl_3$) δ -118.1 (m); HRMS (ESI): Found: *m/z* 413.1911. Calcd for $C_{28}H_{26}O_2F$ $(M+H)^+$ 413.1917.

1-(4-fluorophenyl)-2-phenylpent-4-en-1-one (7)

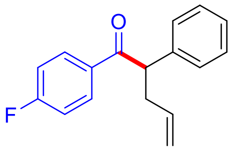

According to the general procedure **A** (0.1 mmol scale), product **7** was isolated in 19% yield (4.8 mg); ^{1}H NMR (400 MHz, $CDCl_3$) δ 8.02-7.93 (2H, m), 7.33-7.27 (4H, m), 7.24-7.17 (1H, m), 7.09-6.99 (2H, m), 5.74 (1H, ddt, J = 17.1, 10.2, 6.9 Hz), 5.03 (1H,

d, J = 17.1 Hz), 4.97 (1H, d, J = 10.2 Hz), 4.56 (1H, t, J = 7.3 Hz), 2.99-2.87 (1H, m), 2.61-2.50 (1H, m); ^{13}C NMR (100 MHz, CDCl$_3$) δ 197.6 (CO), 165.5 (CF, d, J = 254.9 Hz), 138.9 (C), 135.9 (CH), 133.1 (C, d, J = 3.1 Hz), 131.3 (CH, d, J = 9.3 Hz), 129.0 (CH), 128.1 (CH), 127.2 (CH), 116.7 (CH$_2$), 115.6 (CH, d, J = 21.8 Hz), 53.7 (CH), 38.1 (CH$_2$); ^{19}F NMR (376MHz, CDCl$_3$) δ -105.4. HRMS (ESI): Found: *m/z* 255.1186. Calcd for $C_{17}H_{16}OF$ $(M+H)^+$ 255.1185.

1-(1-(4-isobutylphenyl)ethoxy)-2,2,6,6-tetramethylpiperidine

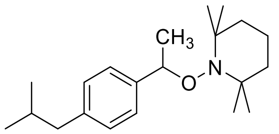

According to the general procedure **A** (0.2 mmol scale), TEMPO-adduct was isolated in 29% yield (18.6 mg); ^{1}H NMR (400 MHz, CDCl$_3$) δ 7.22 (d, J = 8.0 Hz, 2H), 7.08 (d, J = 8.0 Hz, 2H), 4.75 (q, J = 6.7 Hz, 1H), 2.46 (d, J = 7.2 Hz, 2H), 1.92-1.79 (m, 1H), 1.60-1.34 (m, 8H), 1.29 (s, 3H), 1.17 (s, 3H), 1.03 (s, 3H), 0.89 (d, J = 6.6 Hz, 6H), 0.63 (s, 3H); ^{13}C NMR (100 MHz, CDCl$_3$) δ 142.9 (C), 140.1 (C), 128.6 (CH), 126.5 (CH), 82.8 (CO), 59.7, 59.5, 45.1 (CH$_2$), 40.3, 34.4, 34.0, 30.2 (CH), 23.2, 22.3, 20.3, 17.2; HRMS (ESI): Found: *m/z* 340.2625. Calcd for $C_{21}H_{35}NONa$ $(M+H)^+$ 255.1185.

Appendix: ^{1}H, ^{13}C, and ^{19}F NMR spectra for new compounds

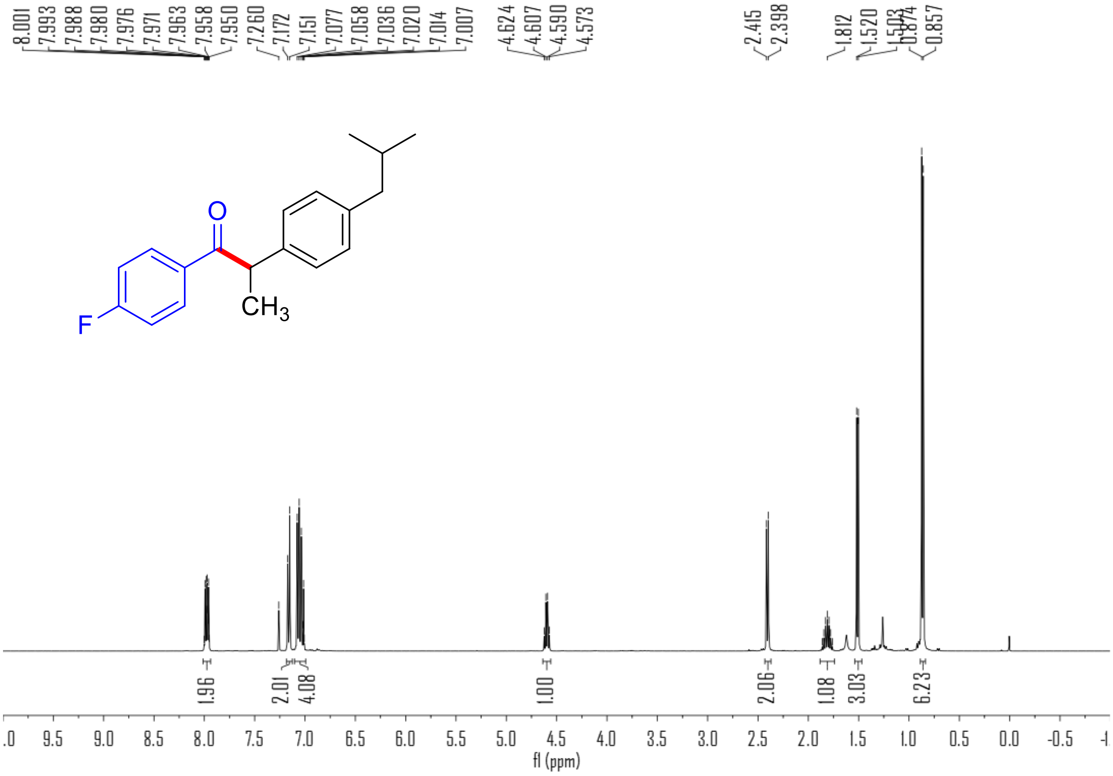

Supplementary Figure 8. ^{1}**H NMR** spectrum of **3a**

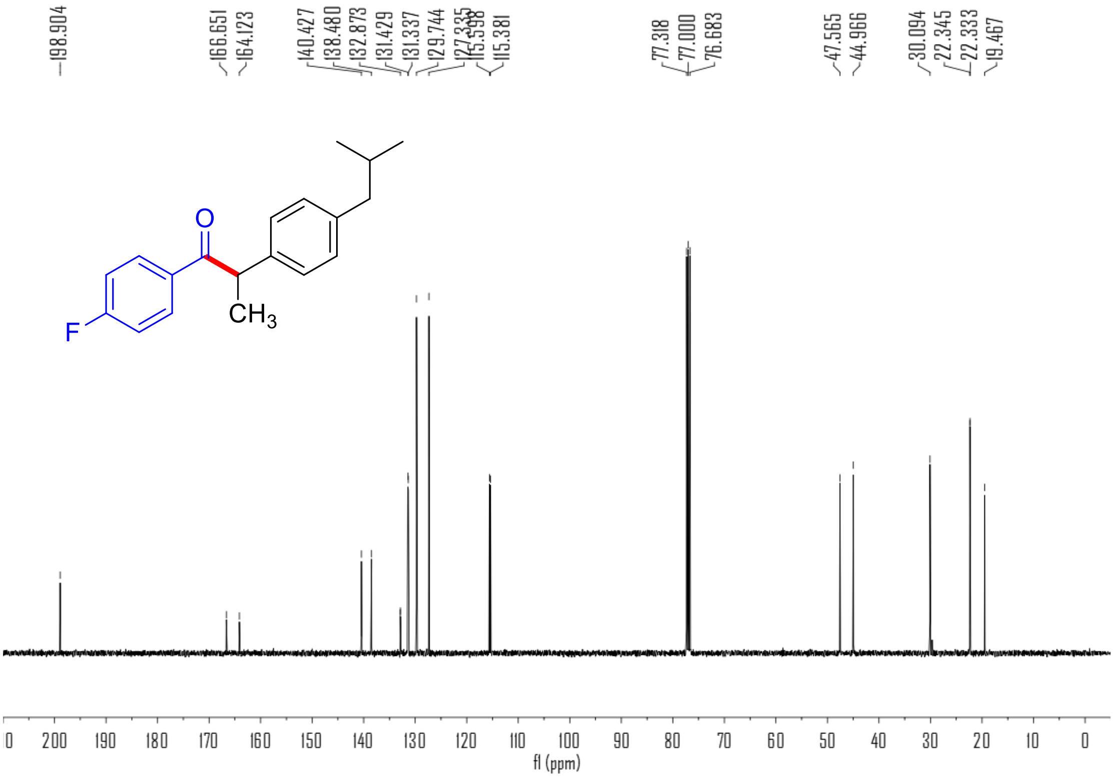

Supplementary Figure 9. ^{13}**C NMR** spectrum of **3a**

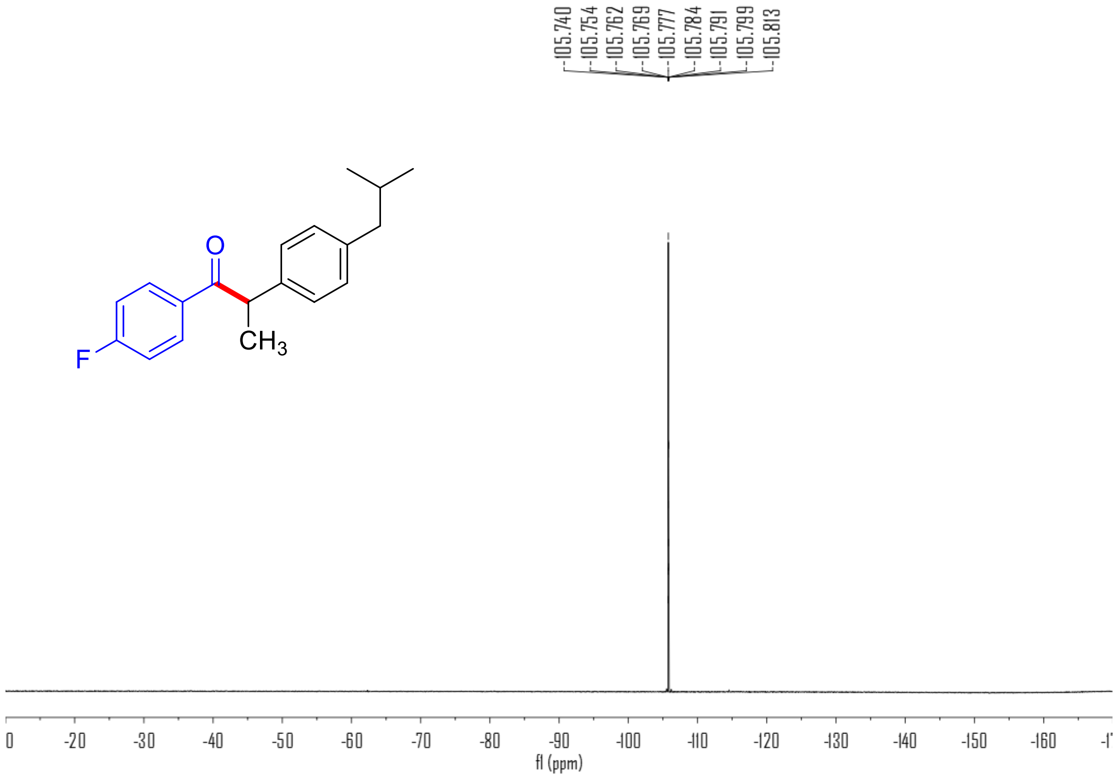

Supplementary Figure 10. ^{19}**F NMR** spectrum of **3a**

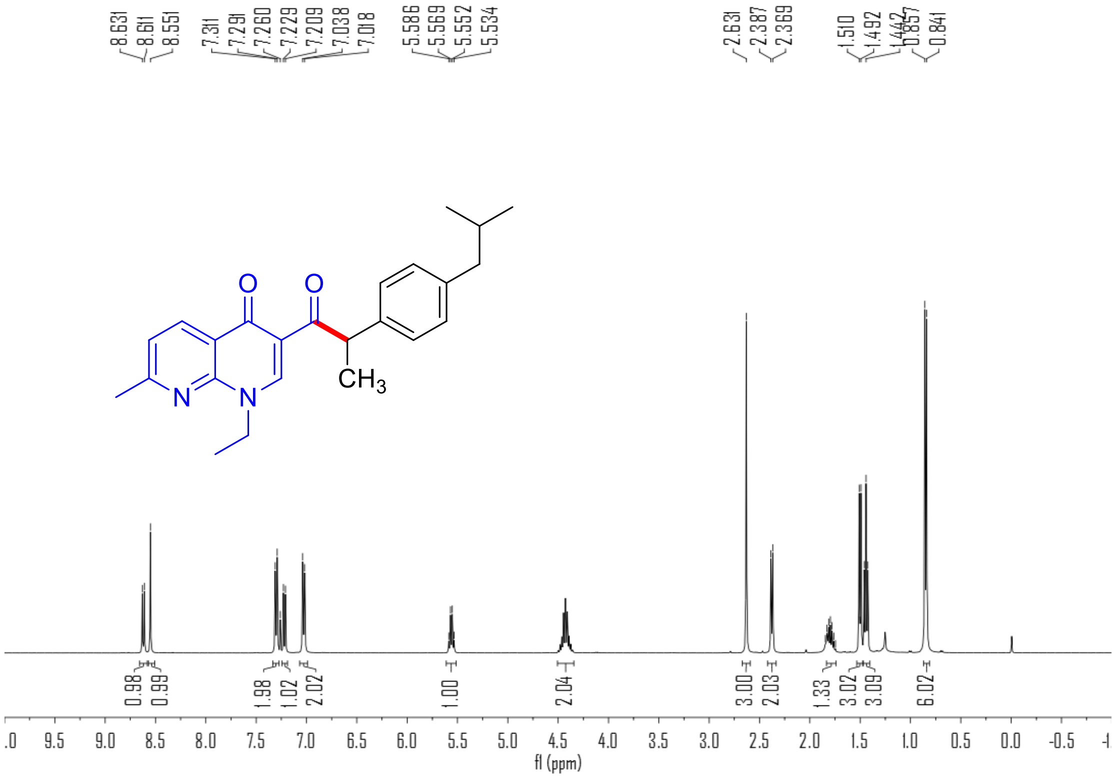

Supplementary Figure 11. ^{1}**H NMR** spectrum of **3b**

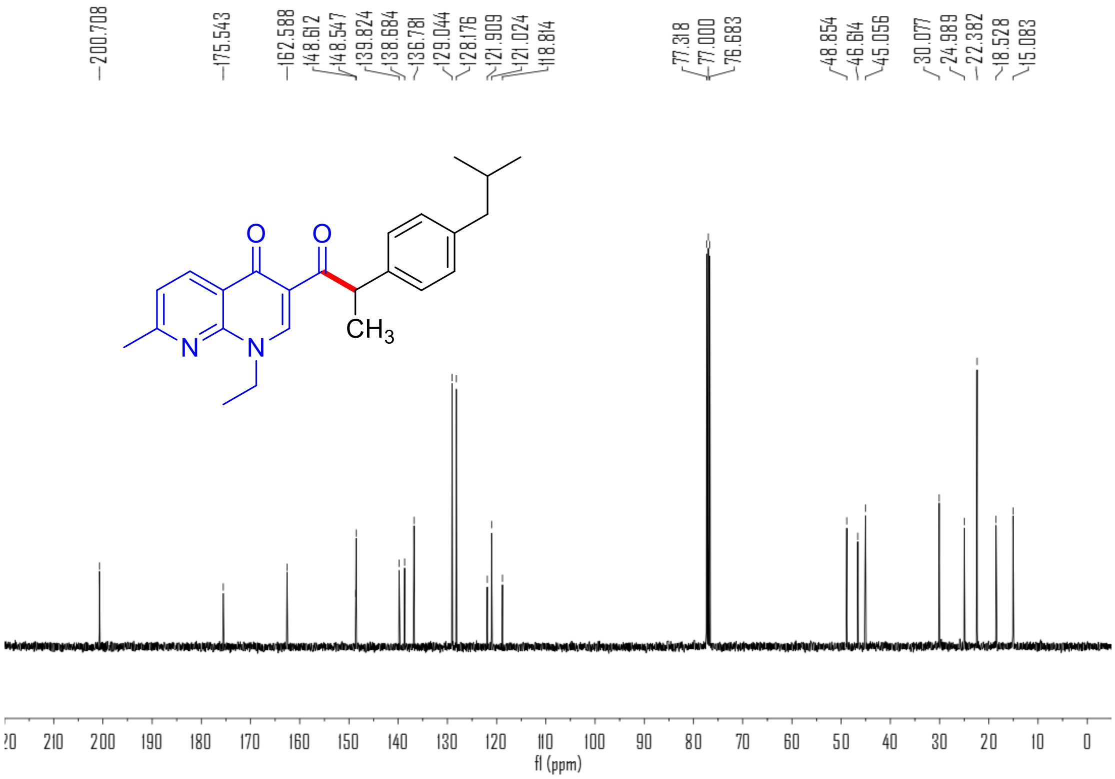

Supplementary Figure 12. ^{13}**C NMR** spectrum of **3b**

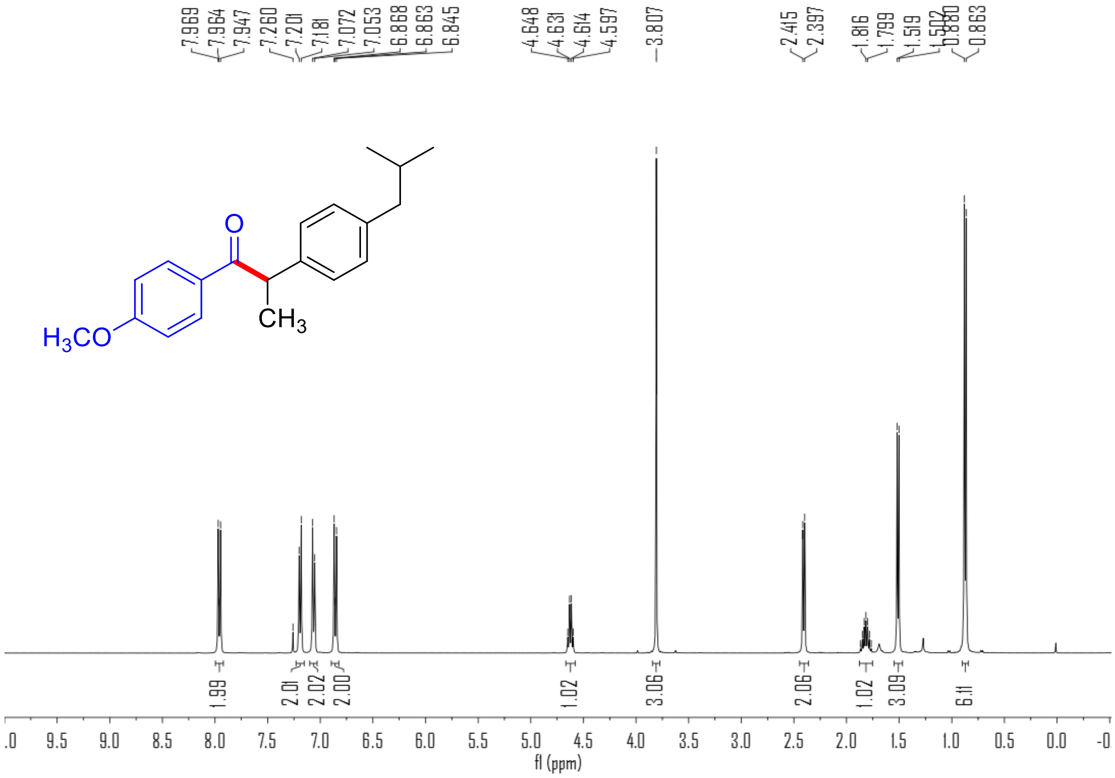

Supplementary Figure 13. ^{1}**H NMR** spectrum of **3c**

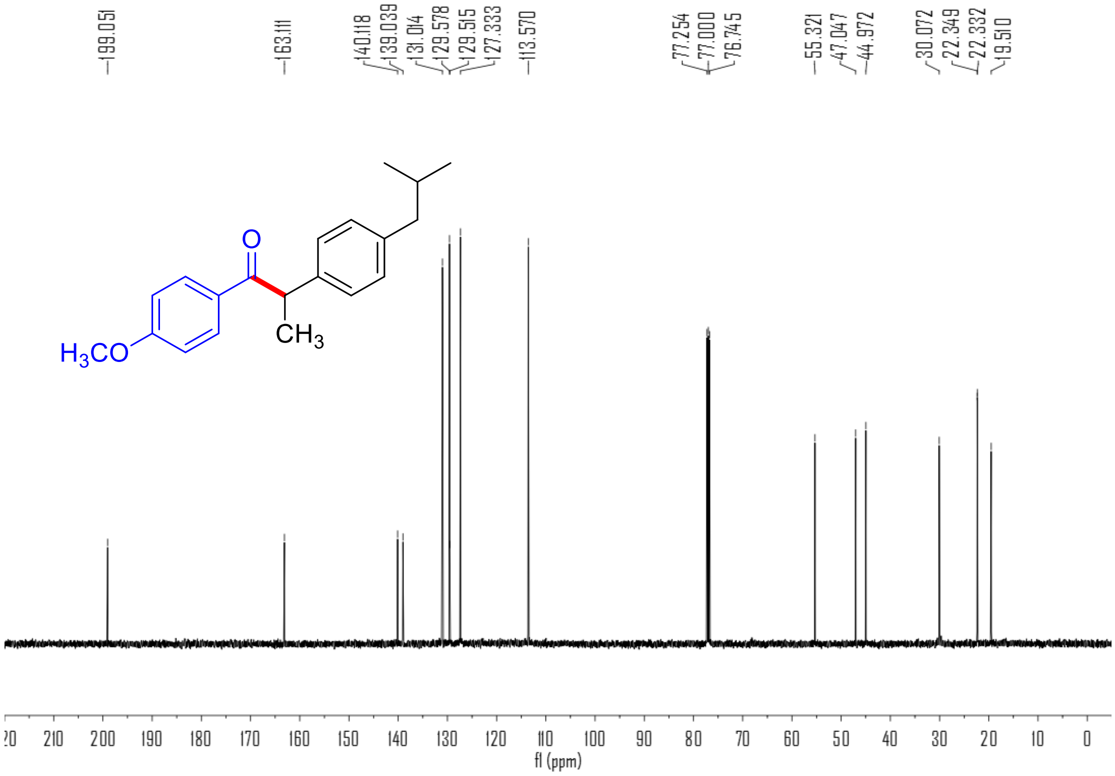

Supplementary Figure 14. ^{13}**C NMR** spectrum of **3c**

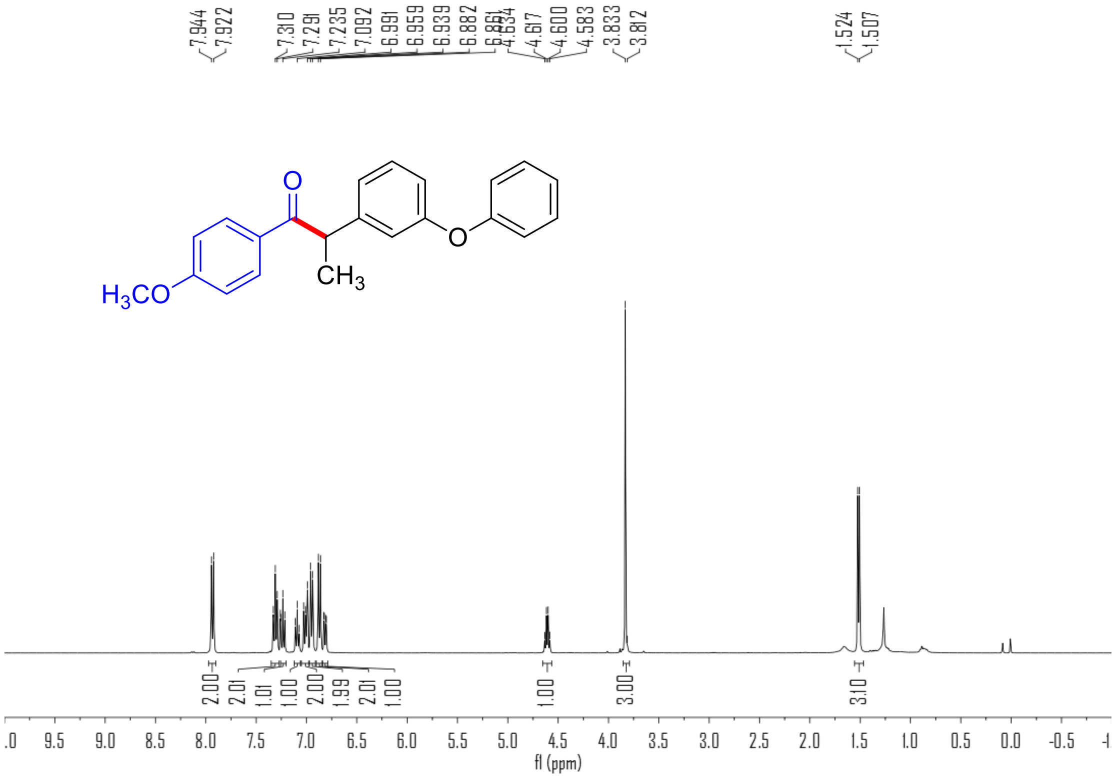

Supplementary Figure 15. ^{1}**H NMR** spectrum of **3d**

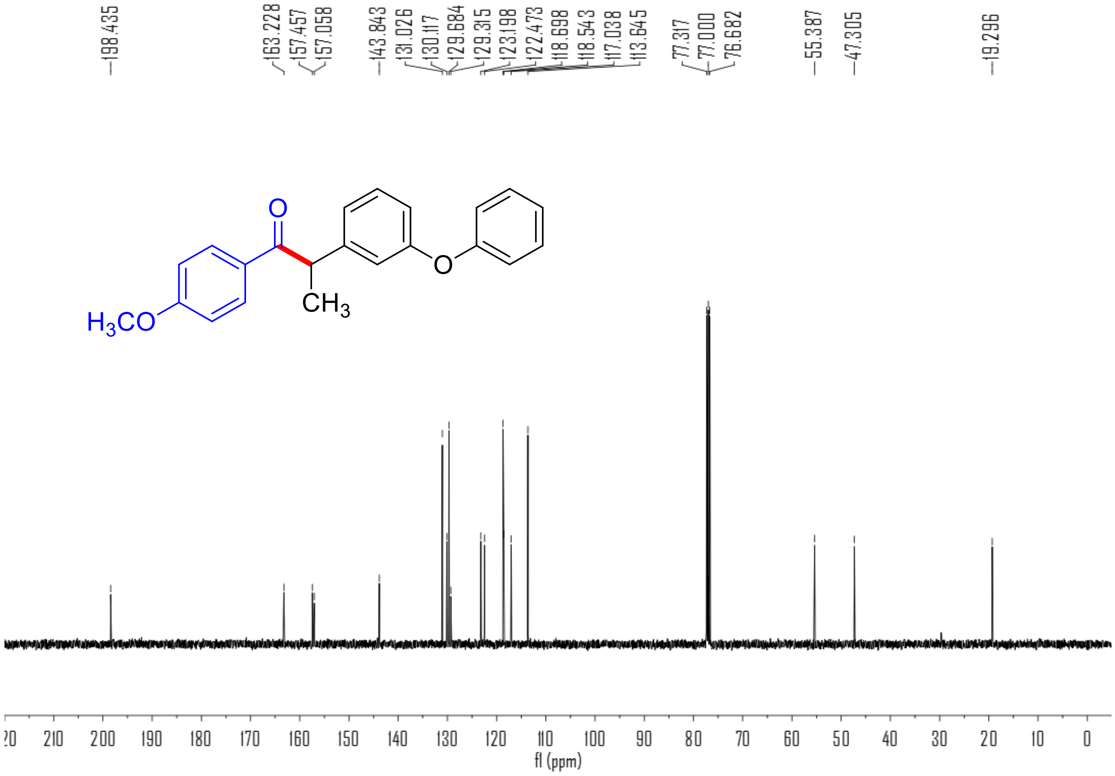

Supplementary Figure 16. ^{13}**C NMR** spectrum of **3d**

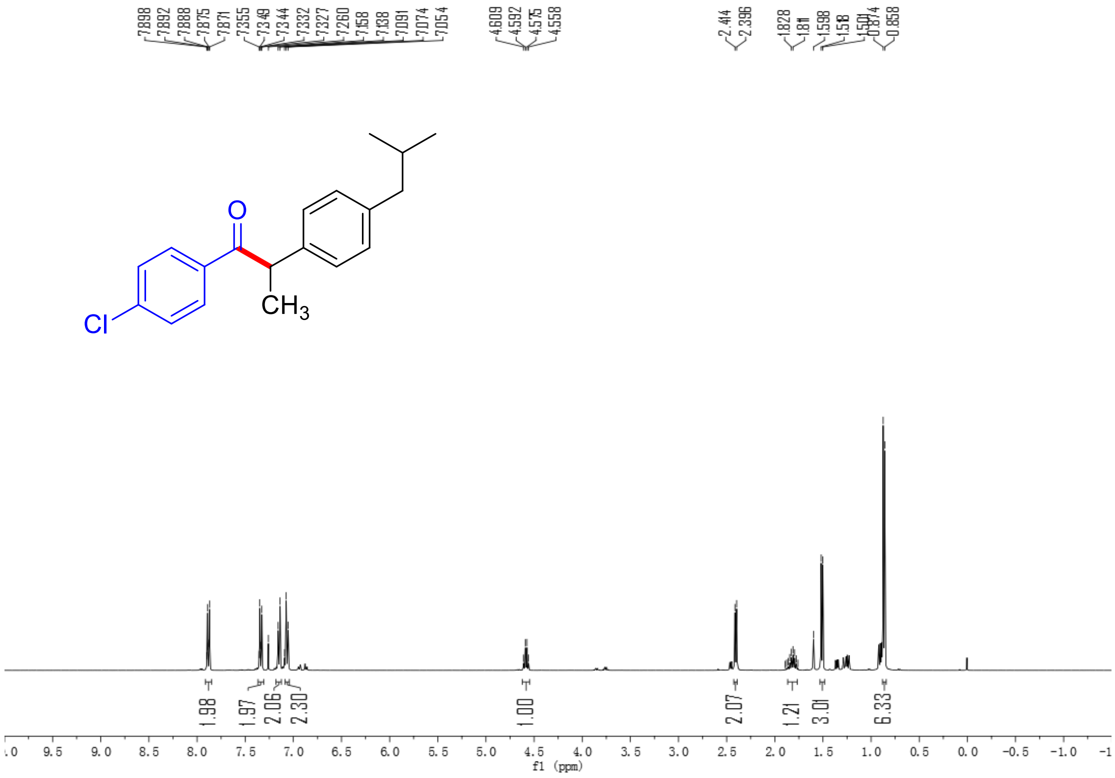

Supplementary Figure 17. ^{1}**H NMR** spectrum of **3e**

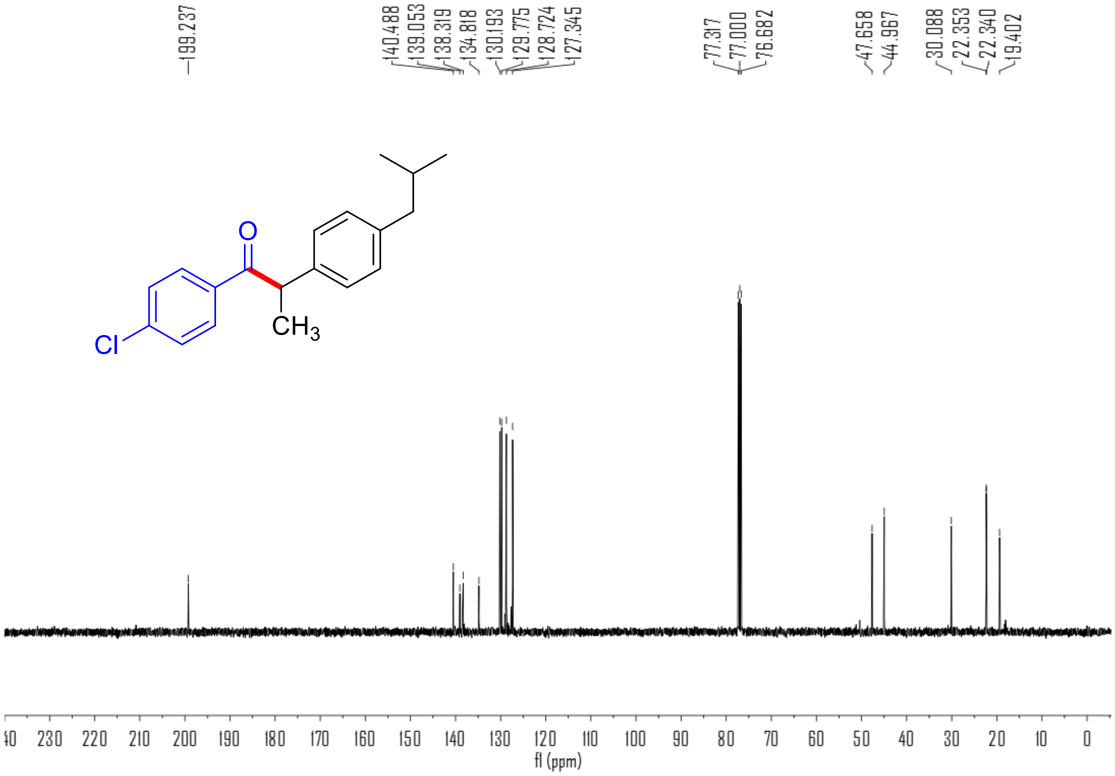

Supplementary Figure 18. ^{13}**C NMR** spectrum of **3e**

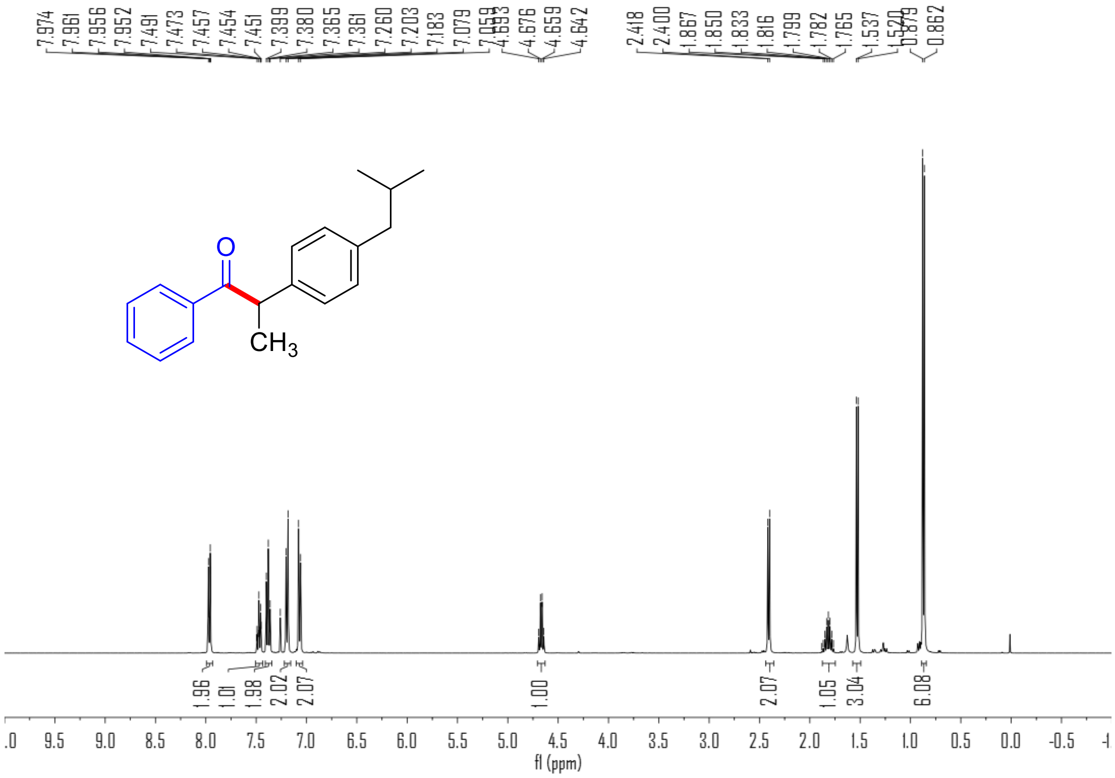

Supplementary Figure 19. ^{1}**H NMR** spectrum of **3f**

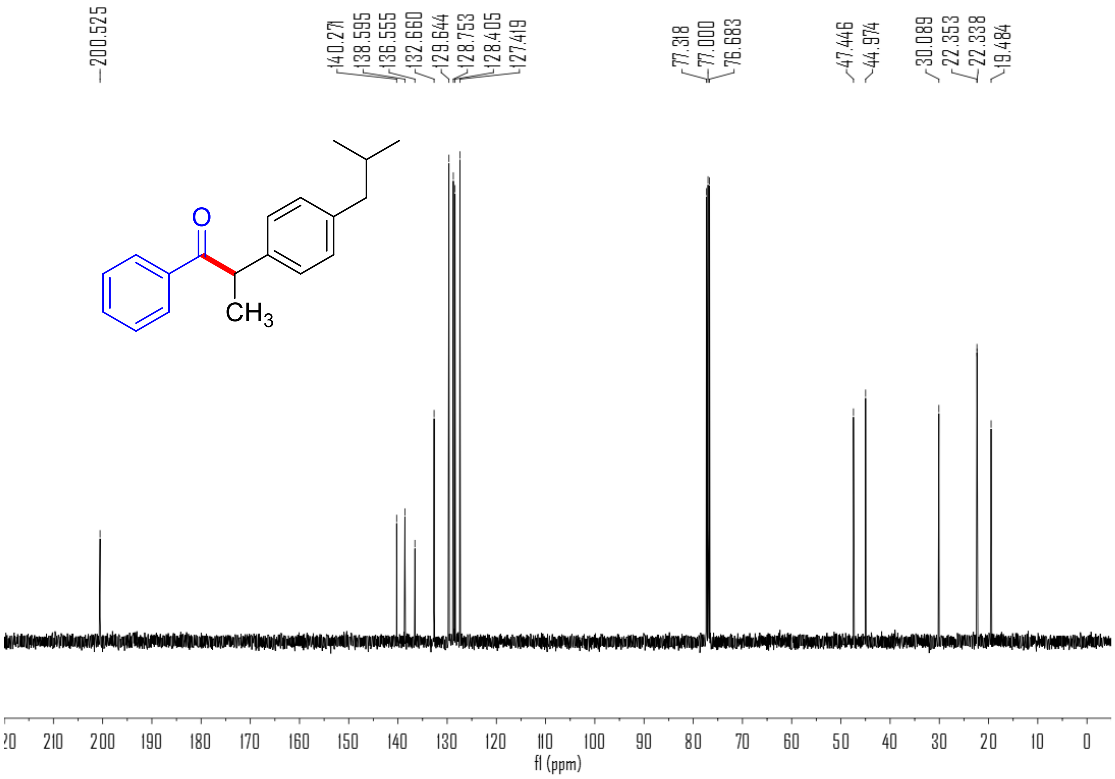

Supplementary Figure 20. ^{13}**C NMR** spectrum of **3f**

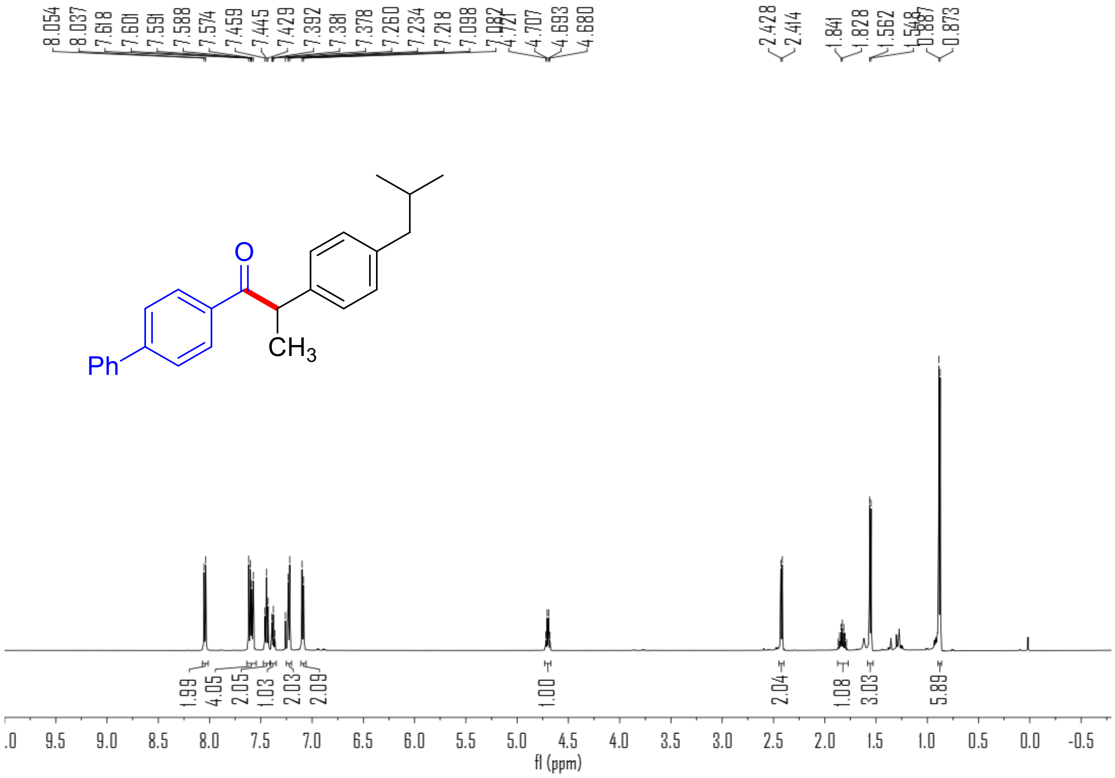

Supplementary Figure 21. ^{1}H **NMR** spectrum of **3g**

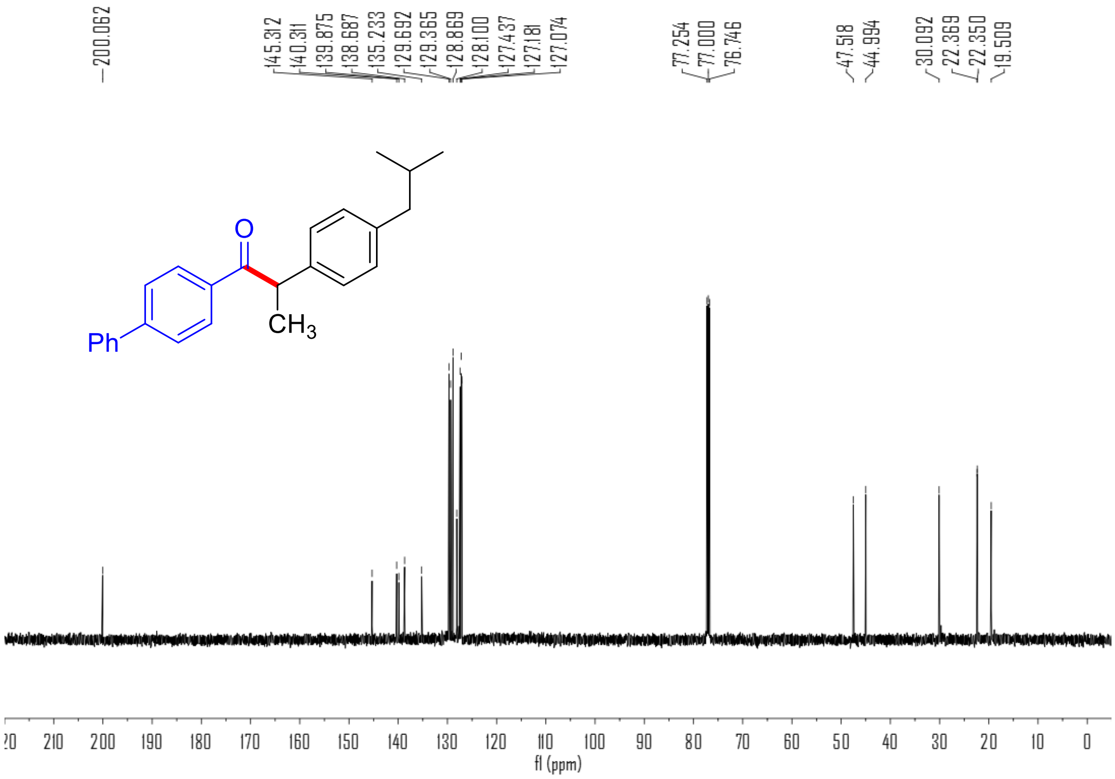

Supplementary Figure 22. ^{13}C **NMR** spectrum of **3g**

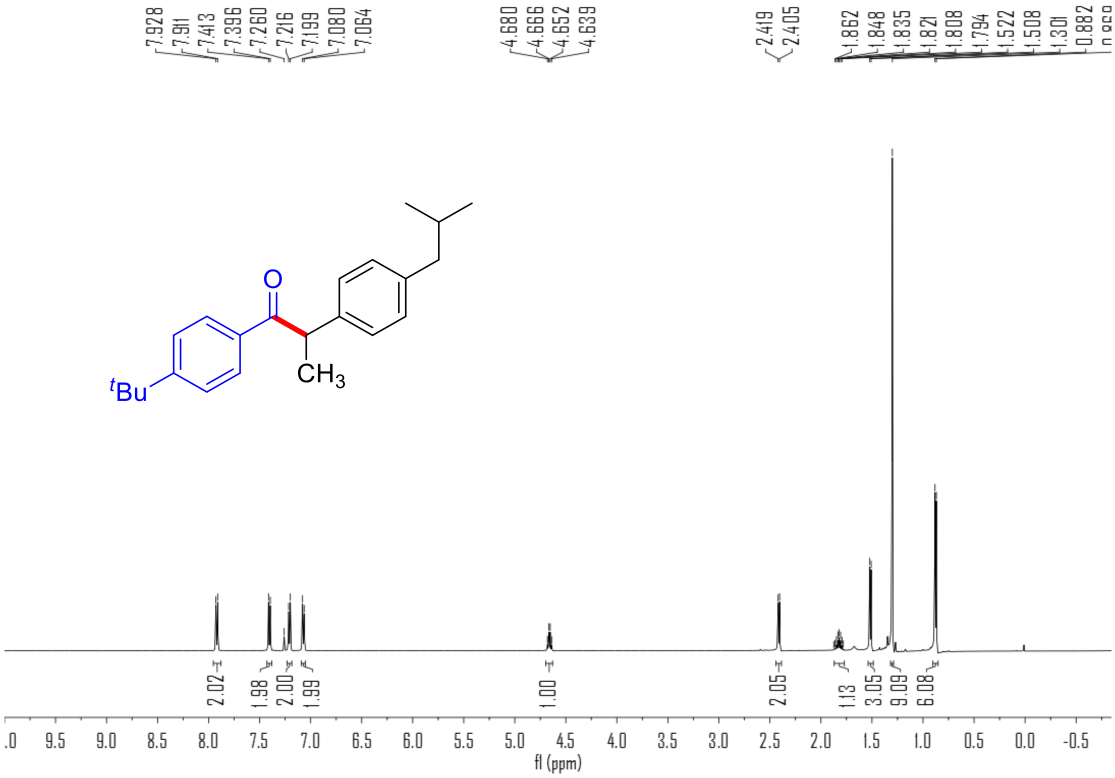

Supplementary Figure 23. ^{1}**H NMR** spectrum of **3h**

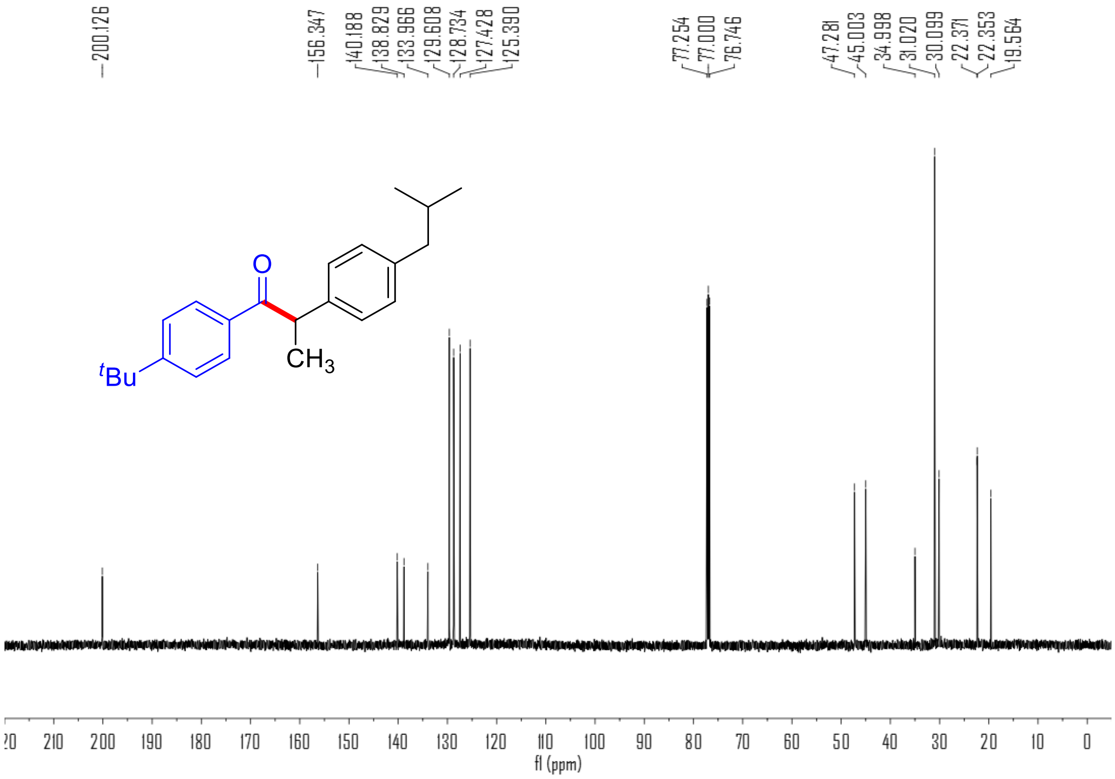

Supplementary Figure 24. ^{13}**C NMR** spectrum of **3h**

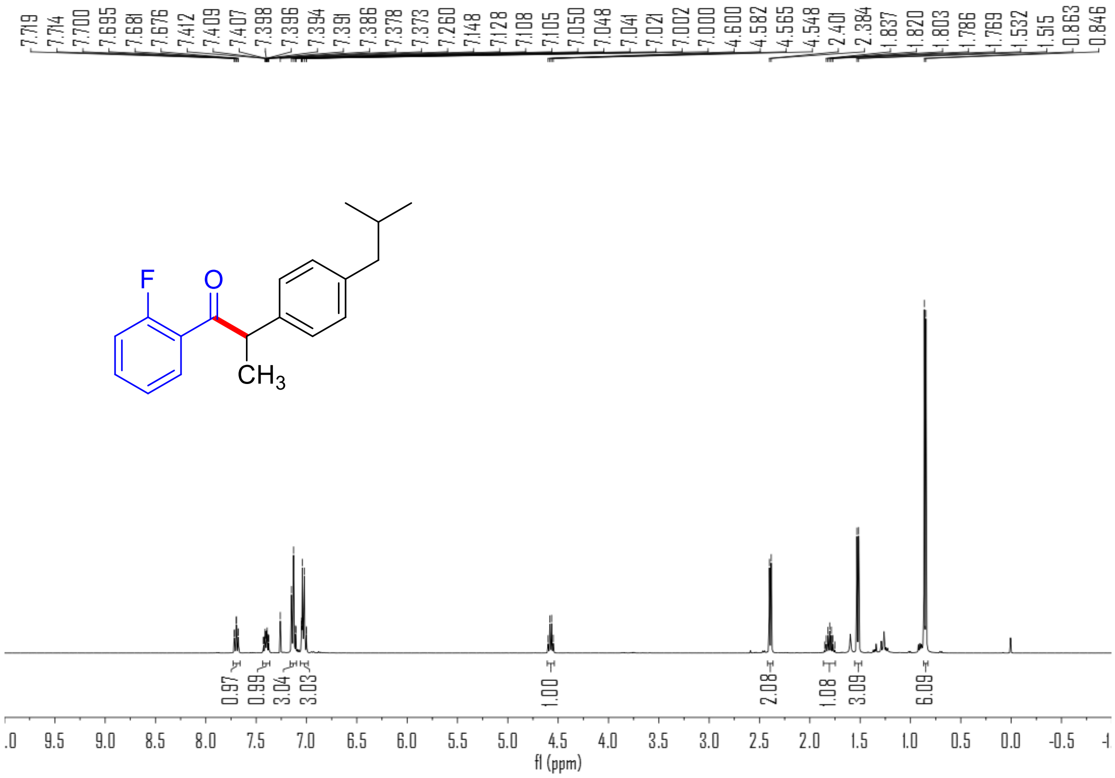

Supplementary Figure 25. ^{1}**H NMR** spectrum of **3i**

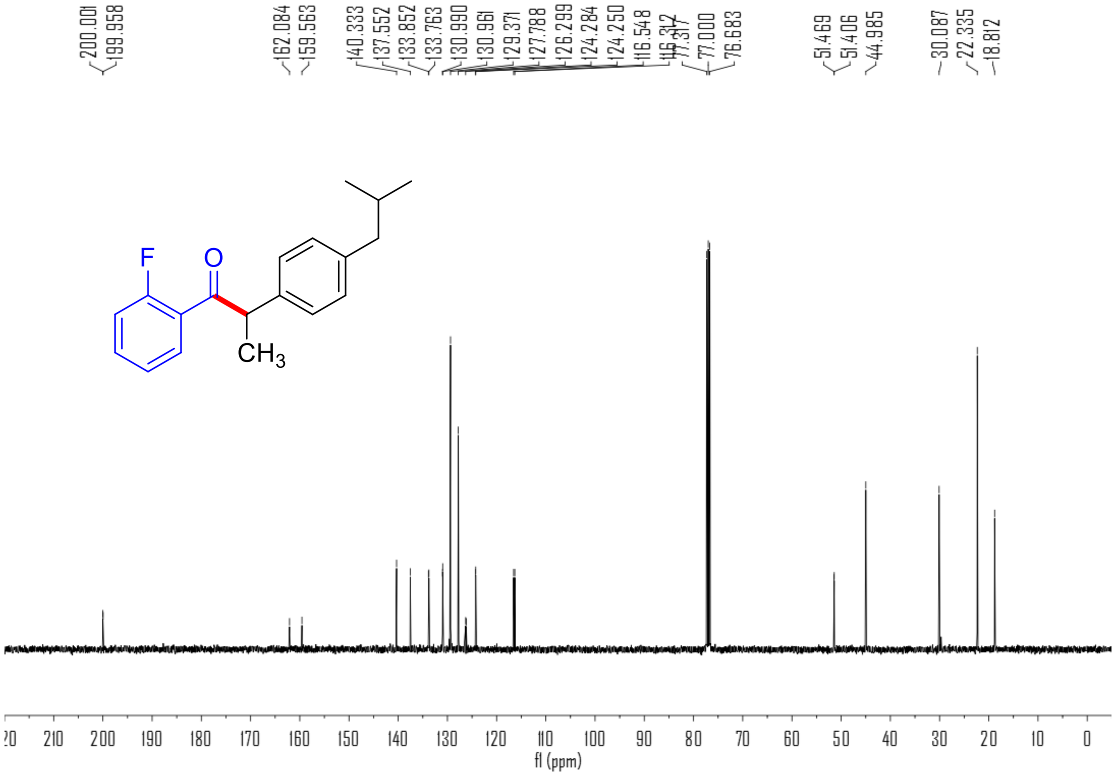

Supplementary Figure 26. ^{13}**C NMR** spectrum of **3i**

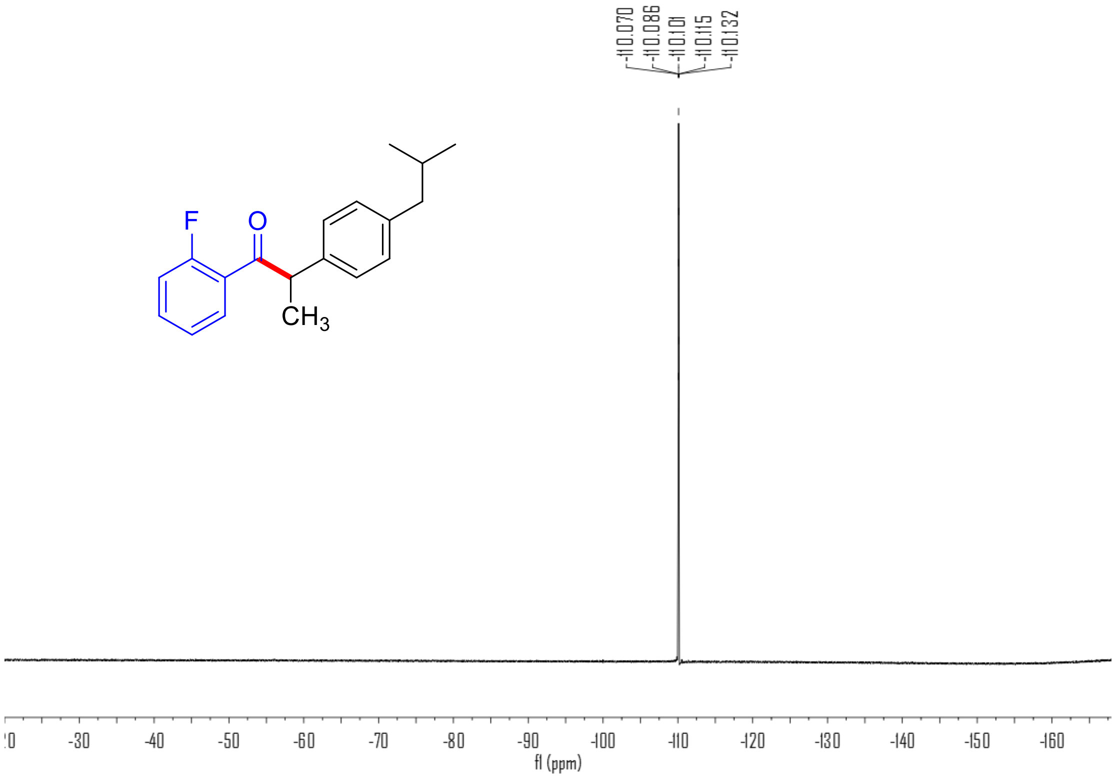

Supplementary Figure 27. ^{19}**F NMR** spectrum of **3i**

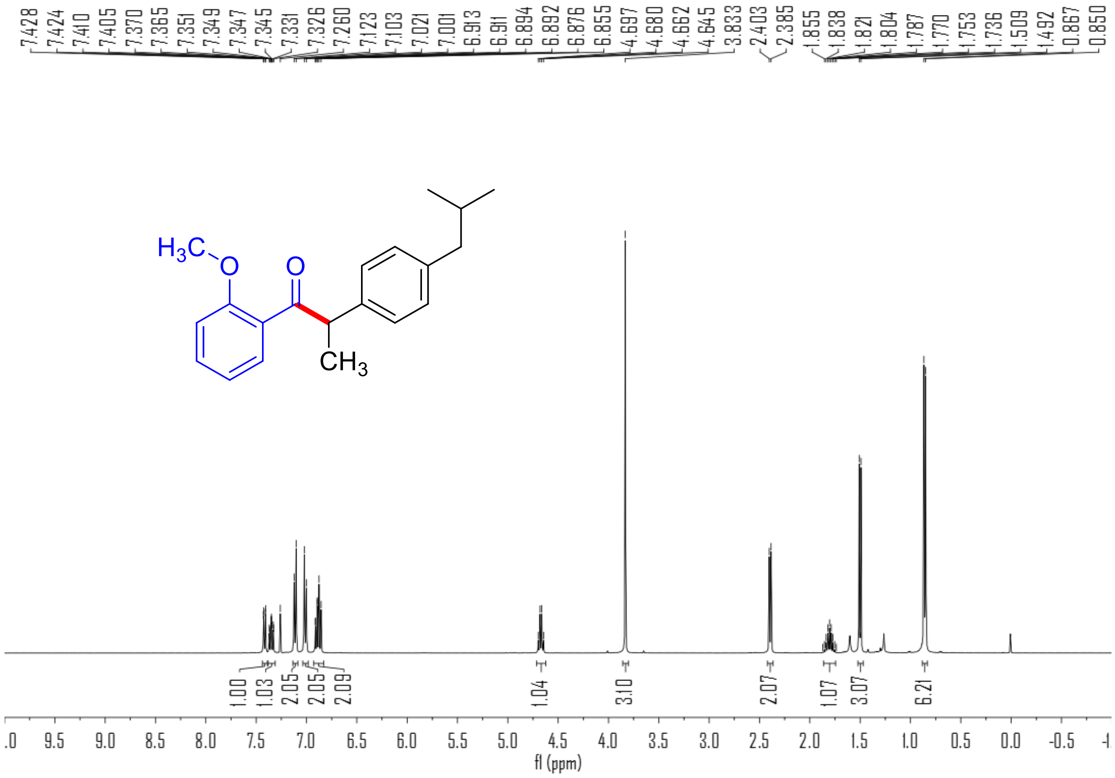

Supplementary Figure 28. ^{1}**H NMR** spectrum of **3j**

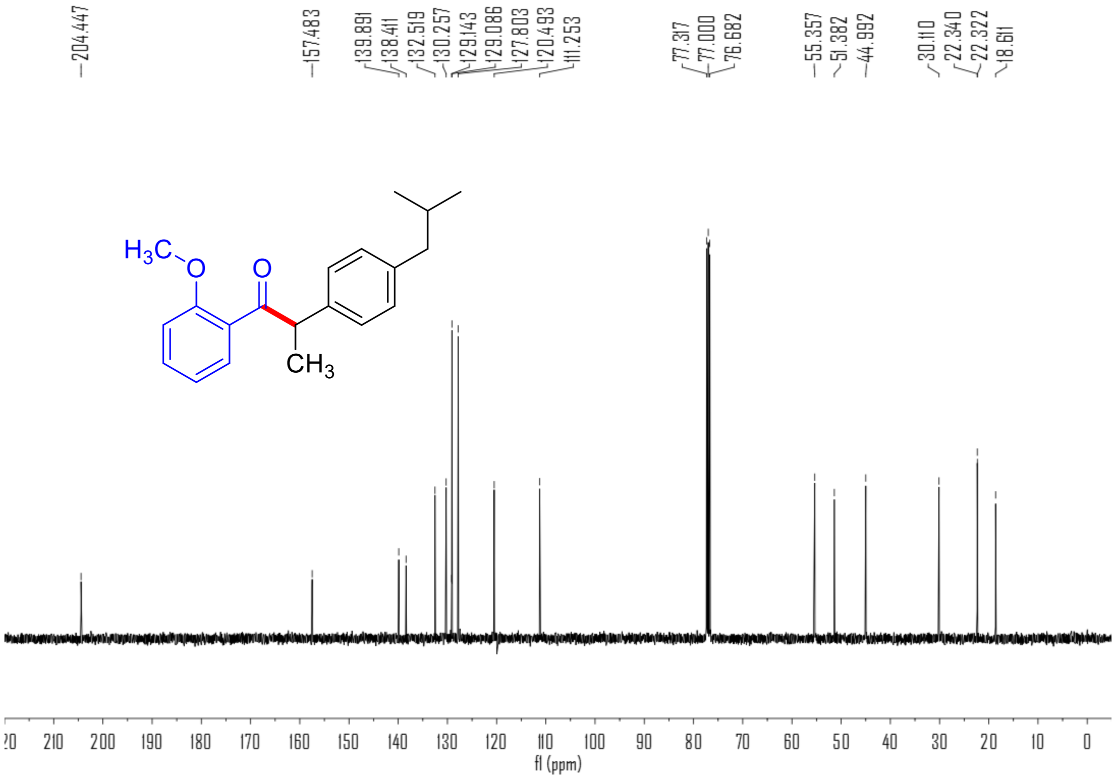

Supplementary Figure 29. ^{13}**C NMR** spectrum of **3j**

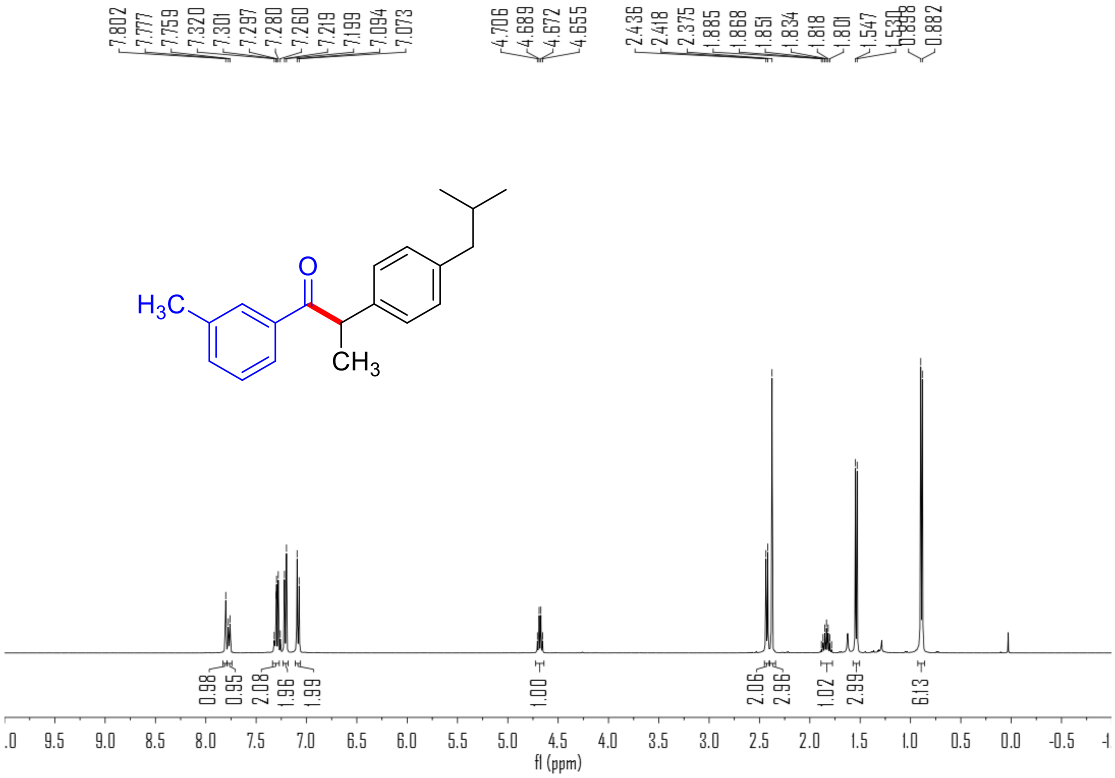

Supplementary Figure 30. ^{1}**H NMR** spectrum of **3k**

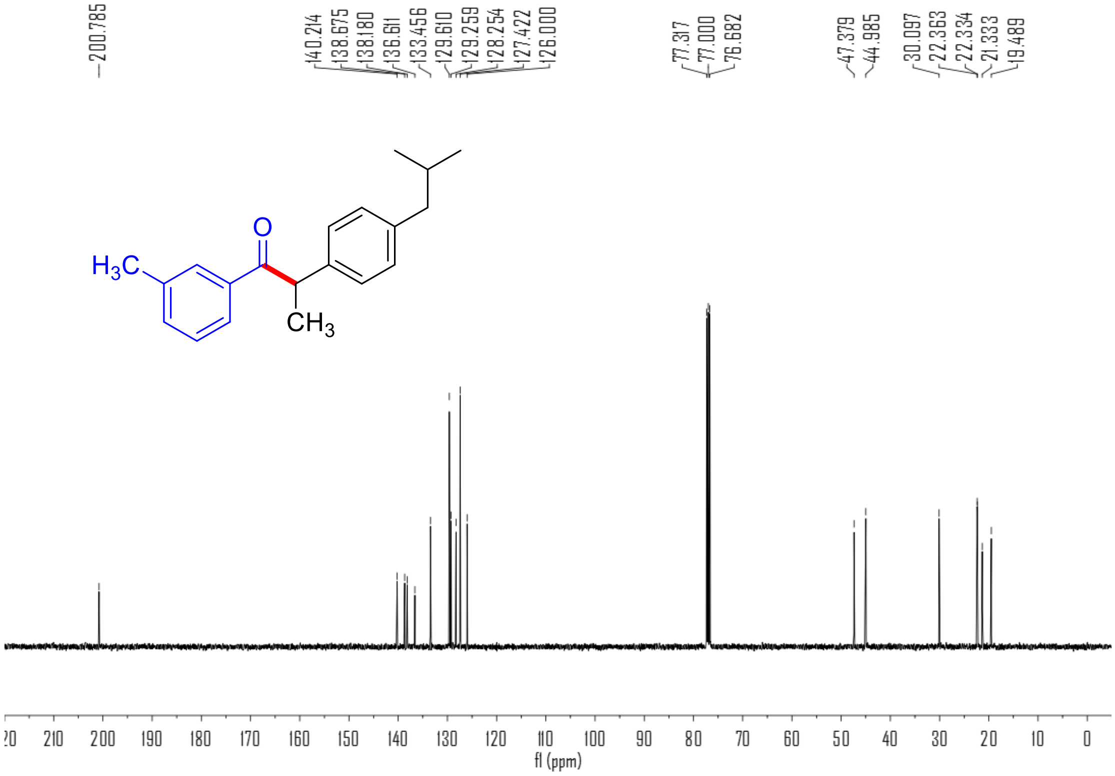

Supplementary Figure 31. ^{13}**C NMR** spectrum of **3k**

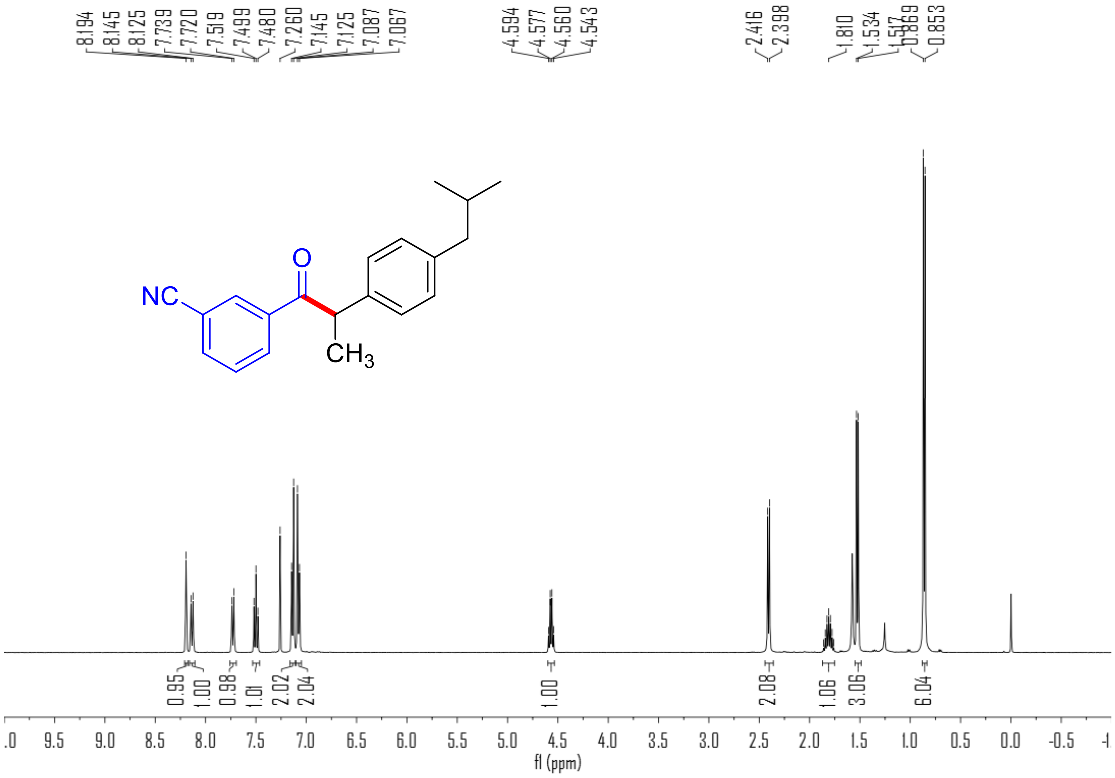

Supplementary Figure 32. ^{1}**H NMR** spectrum of **3l**

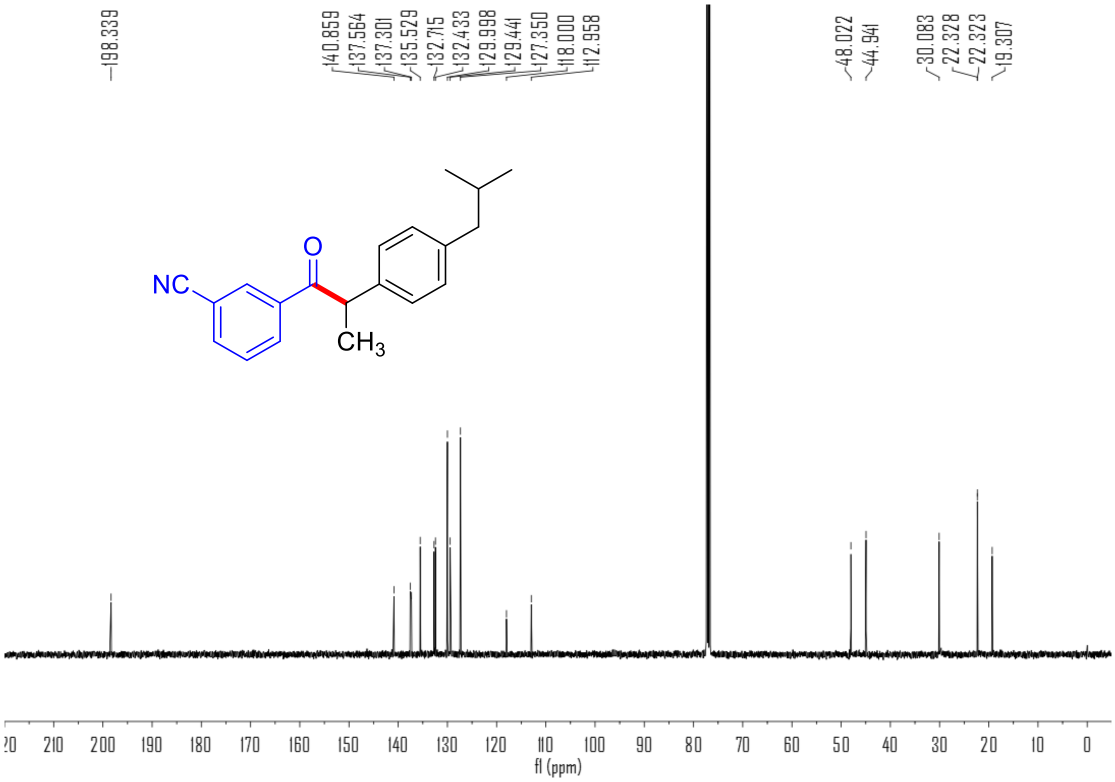

Supplementary Figure 33. ^{13}**C NMR** spectrum of **3l**

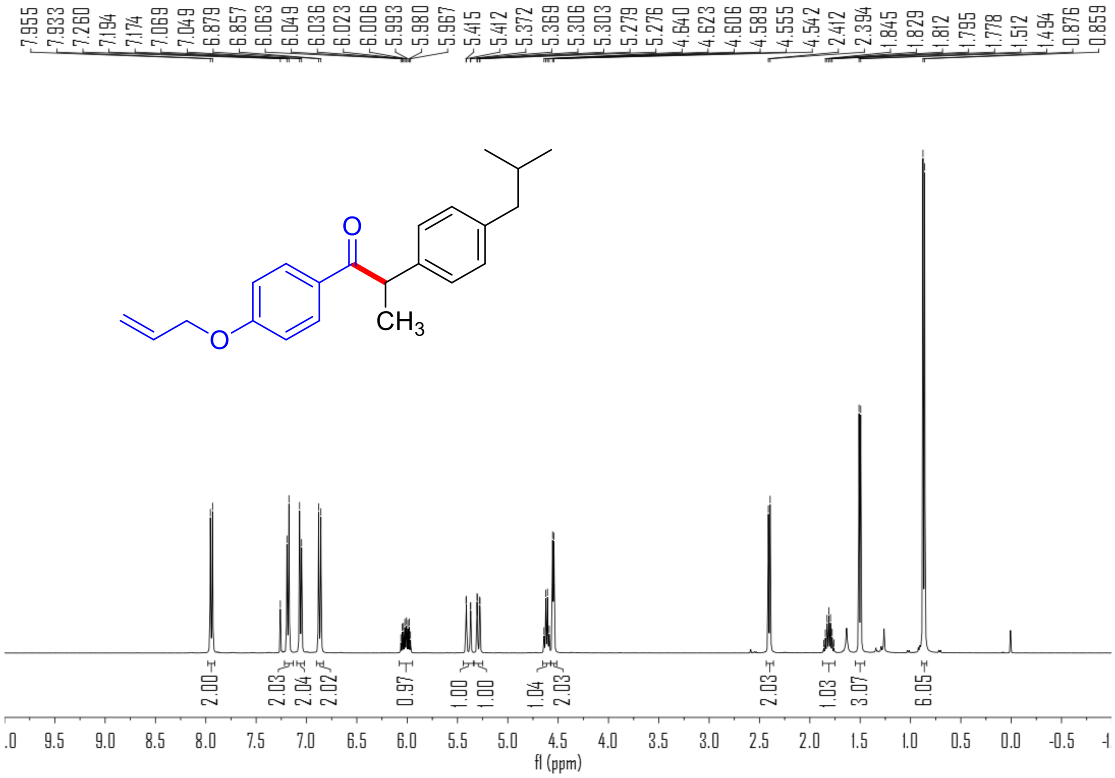

Supplementary Figure 34. **^{1}H NMR** spectrum of **3m**

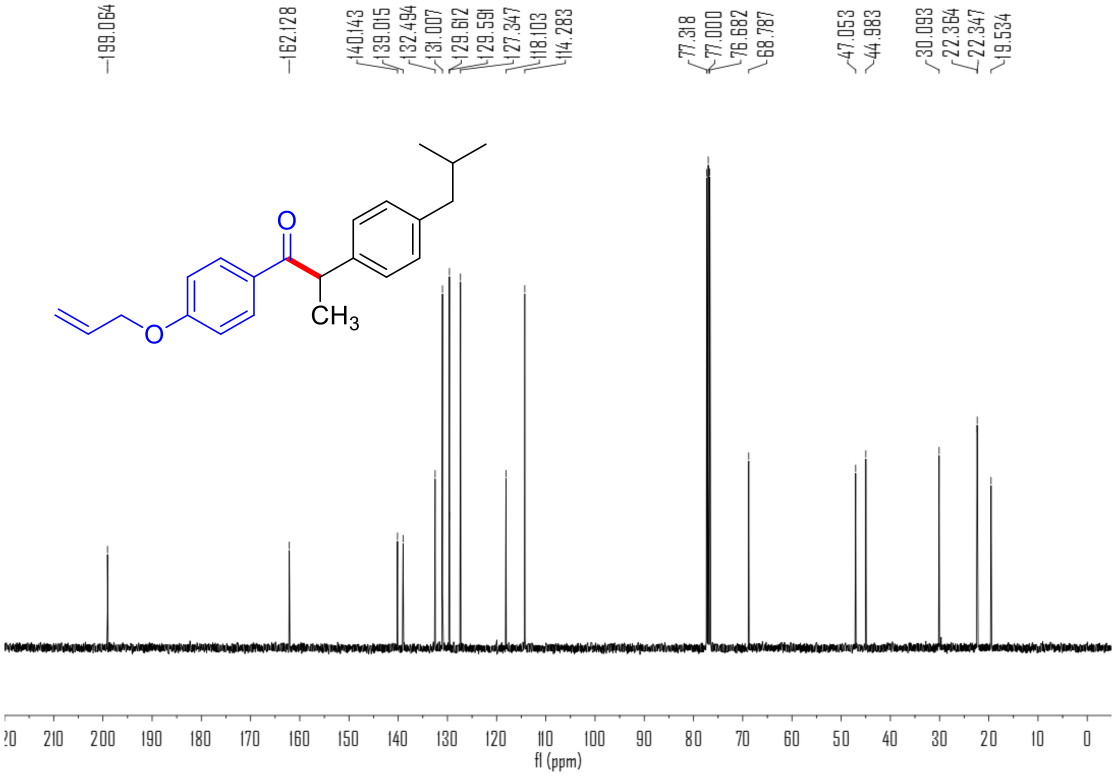

Supplementary Figure 35. **^{13}C NMR** spectrum of **3m**

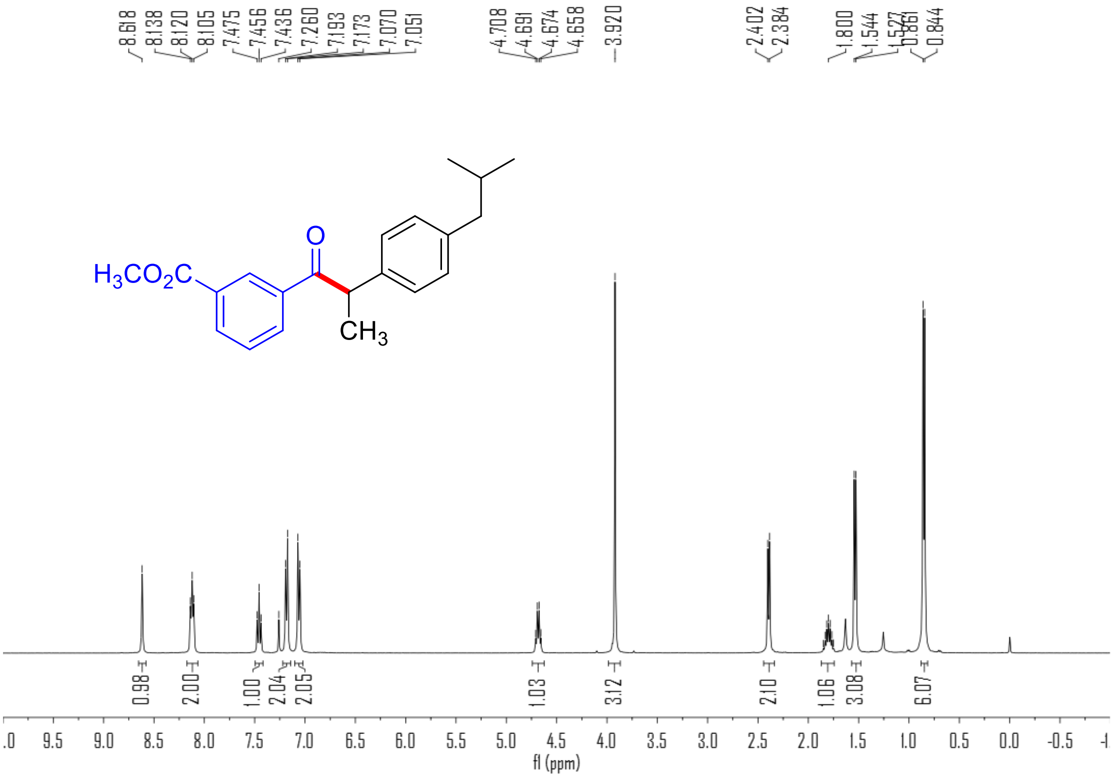

Supplementary Figure 36. ^{1}**H NMR** spectrum of **3n**

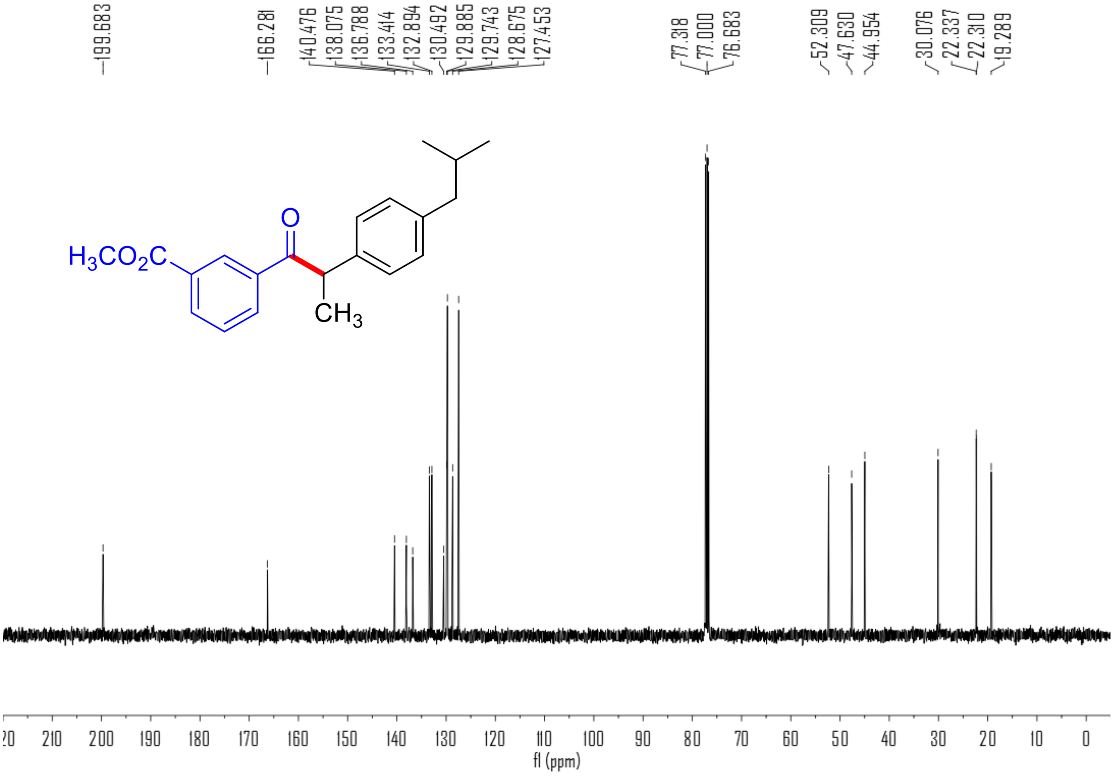

Supplementary Figure 37. ^{13}**C NMR** spectrum of **3n**

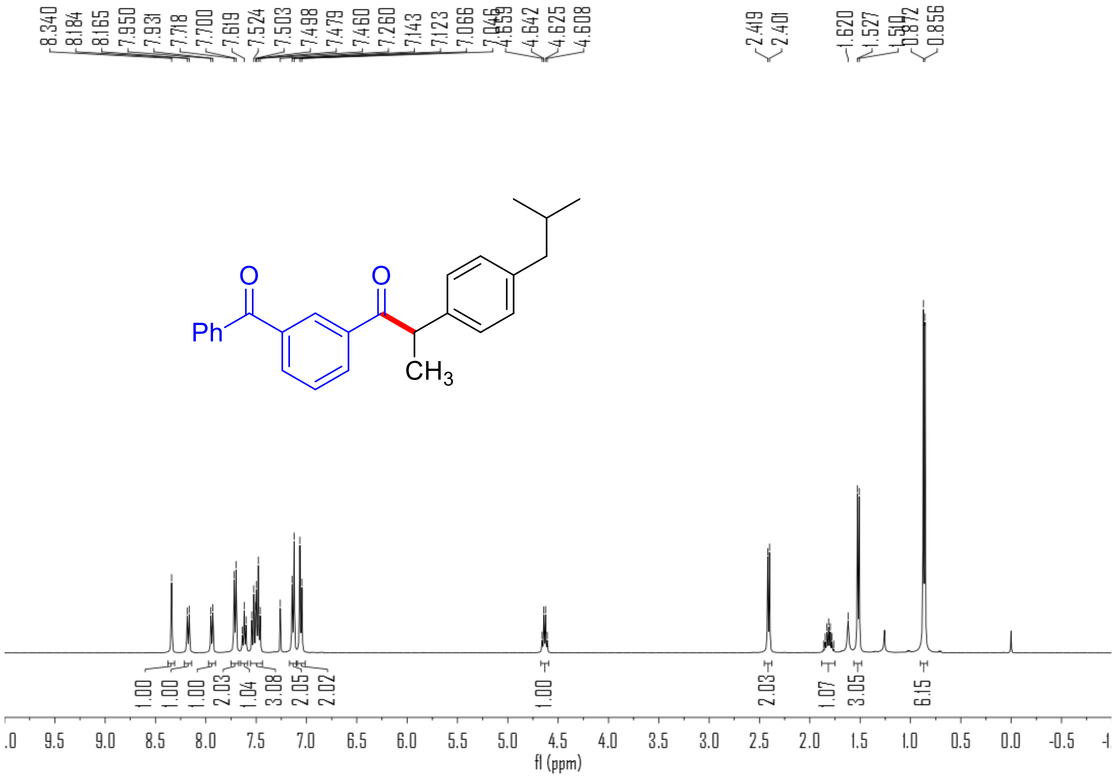

Supplementary Figure 38. ^{1}H NMR spectrum of **3o**

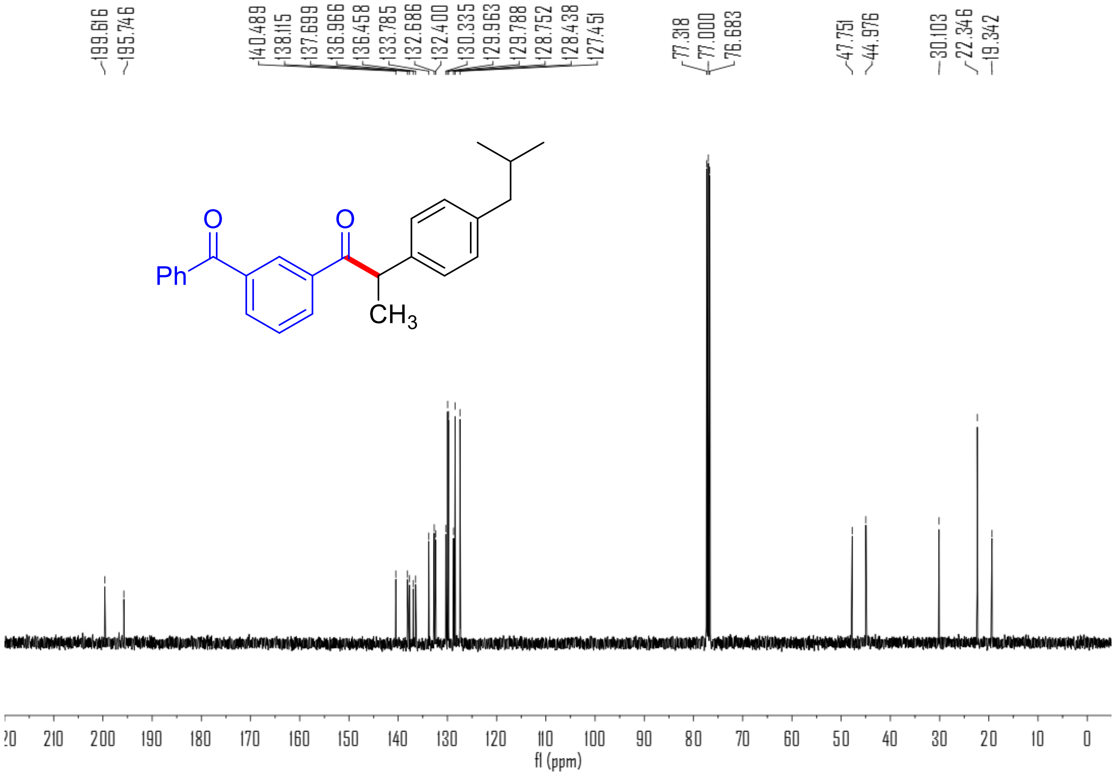

Supplementary Figure 39. ^{13}C NMR spectrum of **3o**

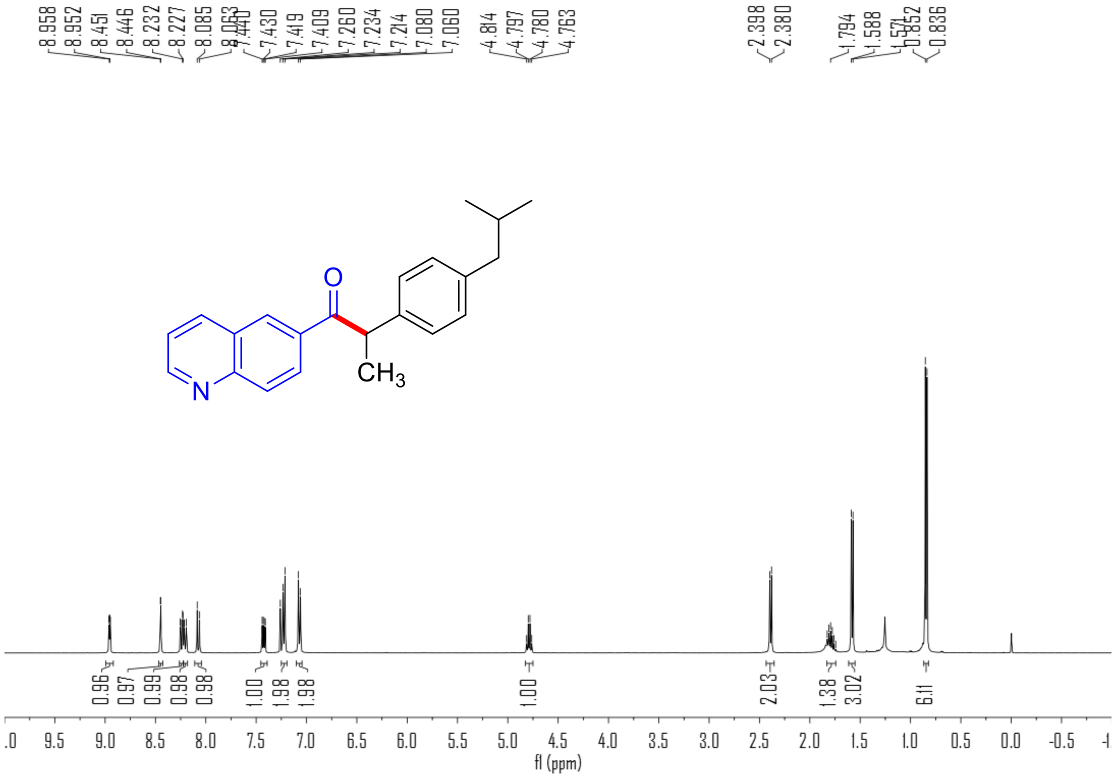

Supplementary Figure 40. ^{1}**H NMR** spectrum of **3p**

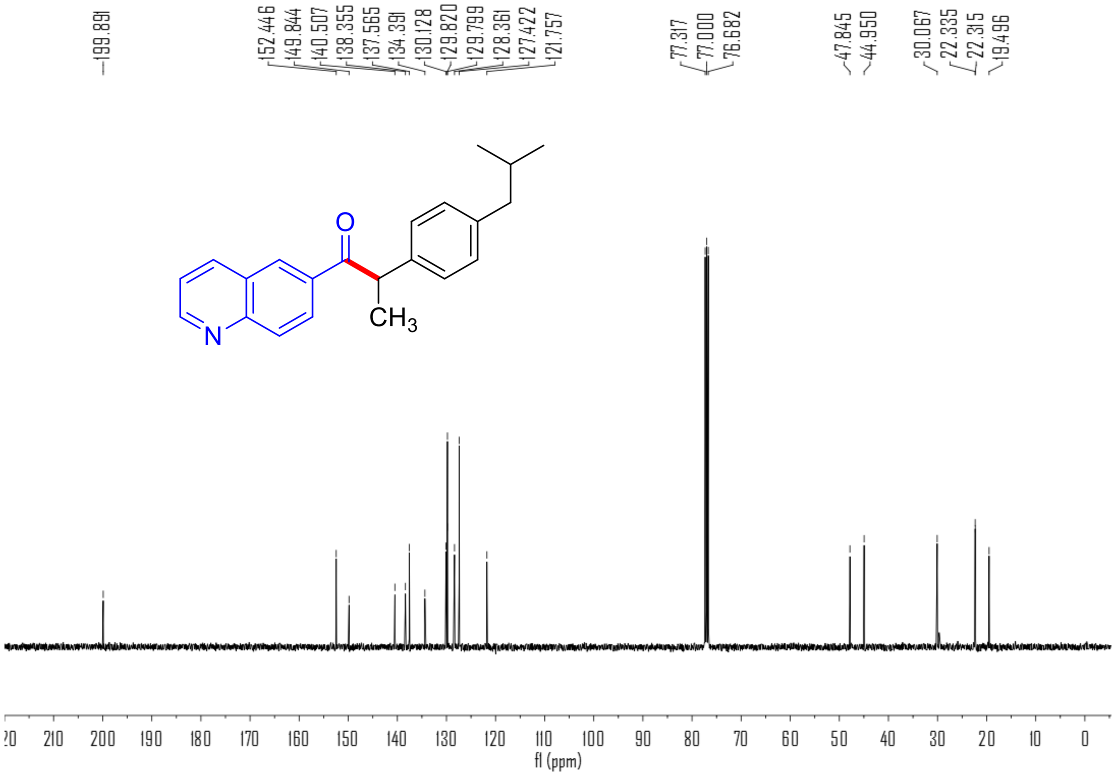

Supplementary Figure 41. ^{13}**C NMR** spectrum of **3p**

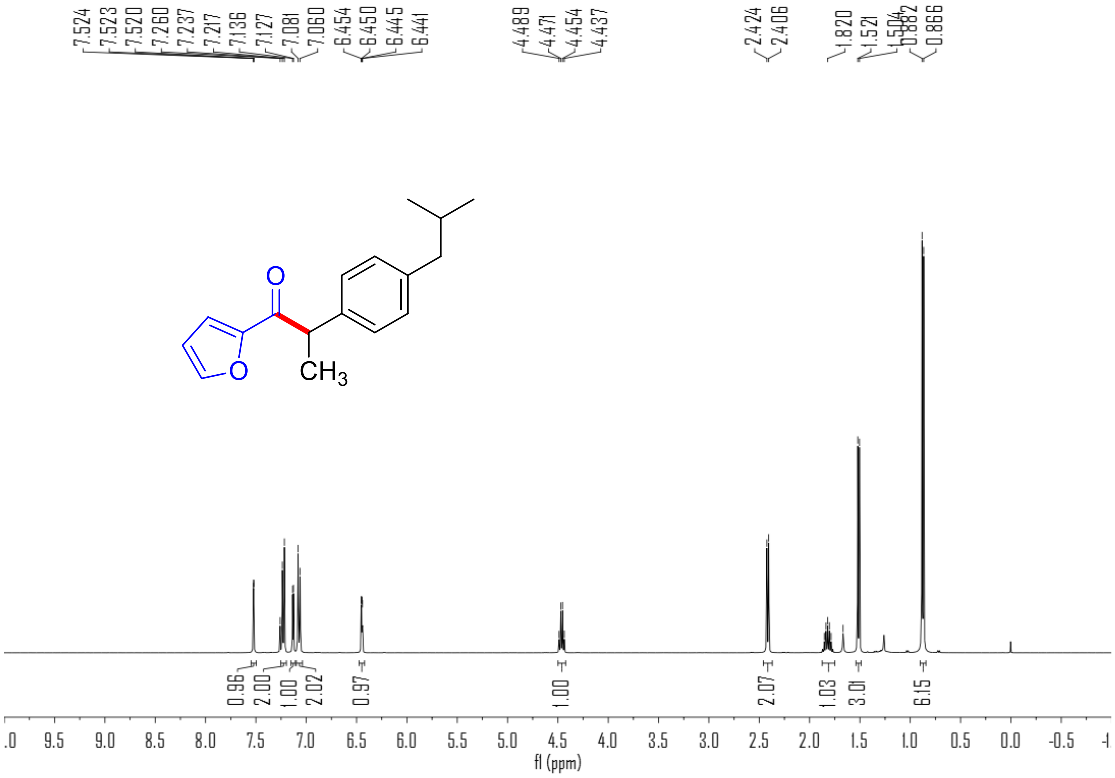

Supplementary Figure 42. **^{1}H NMR** spectrum of **3q**

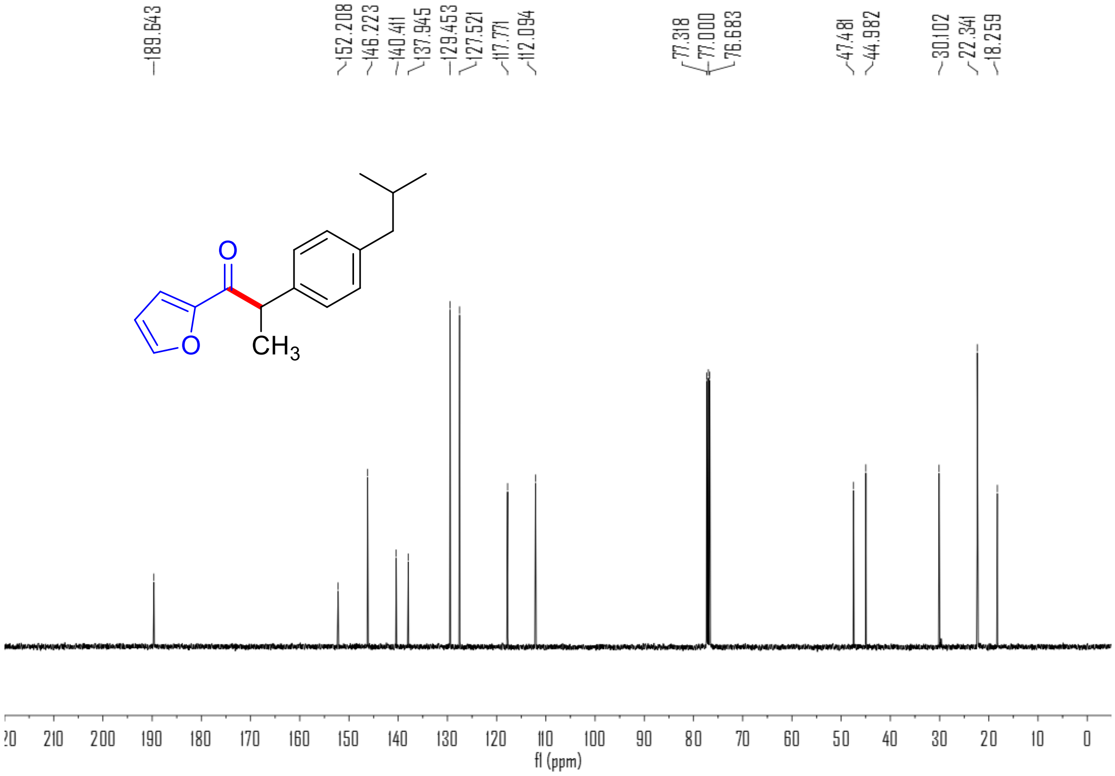

Supplementary Figure 43. **^{13}C NMR** spectrum of **3q**

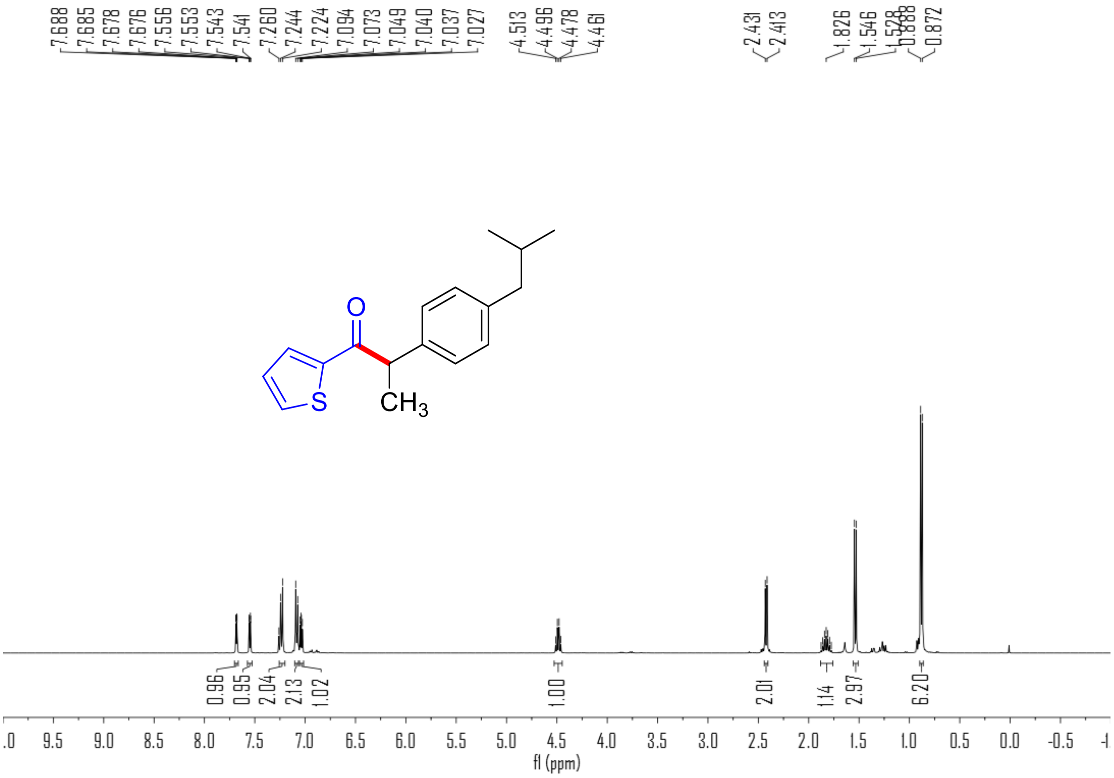

Supplementary Figure 44. ^{1}**H NMR** spectrum of **3r**

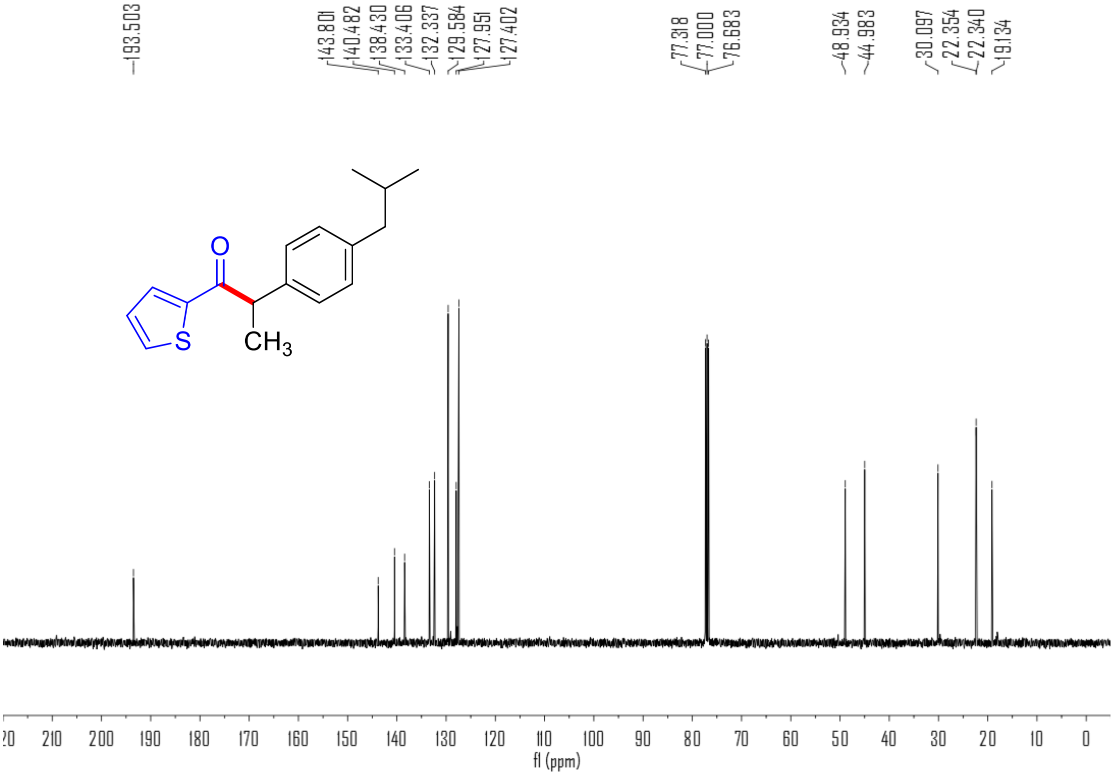

Supplementary Figure 45. ^{13}**C NMR** spectrum of **3r**

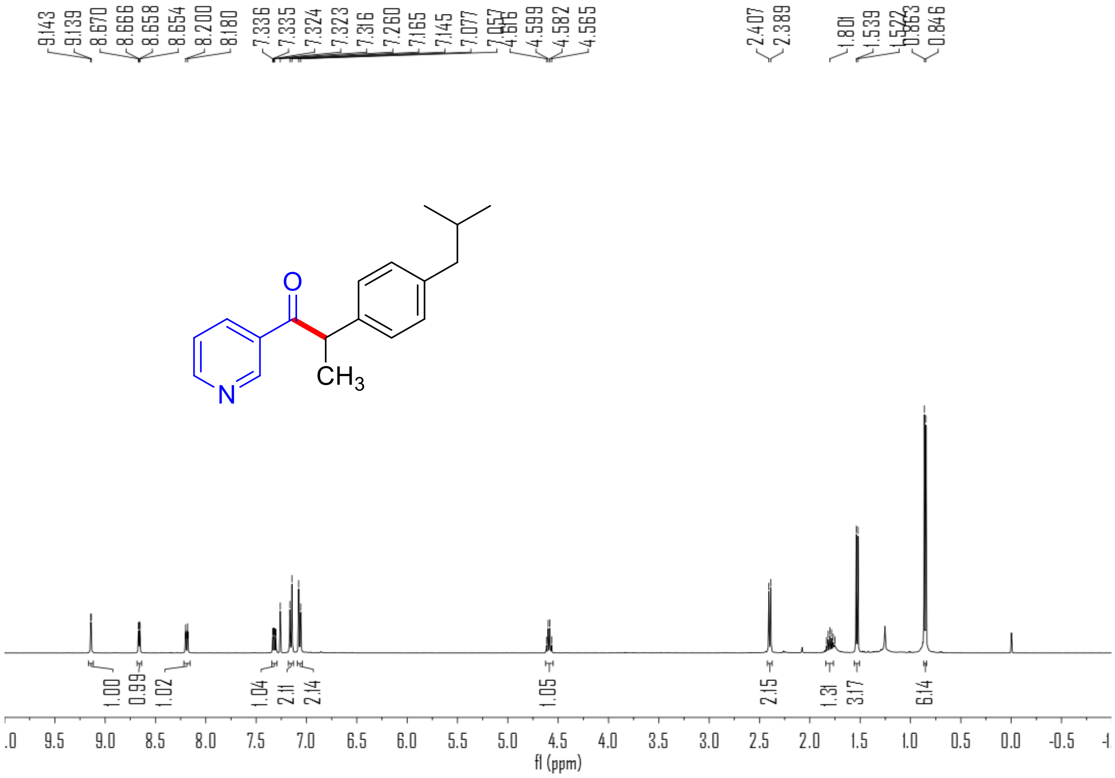

Supplementary Figure 46. ^{1}**H NMR** spectrum of **3s**

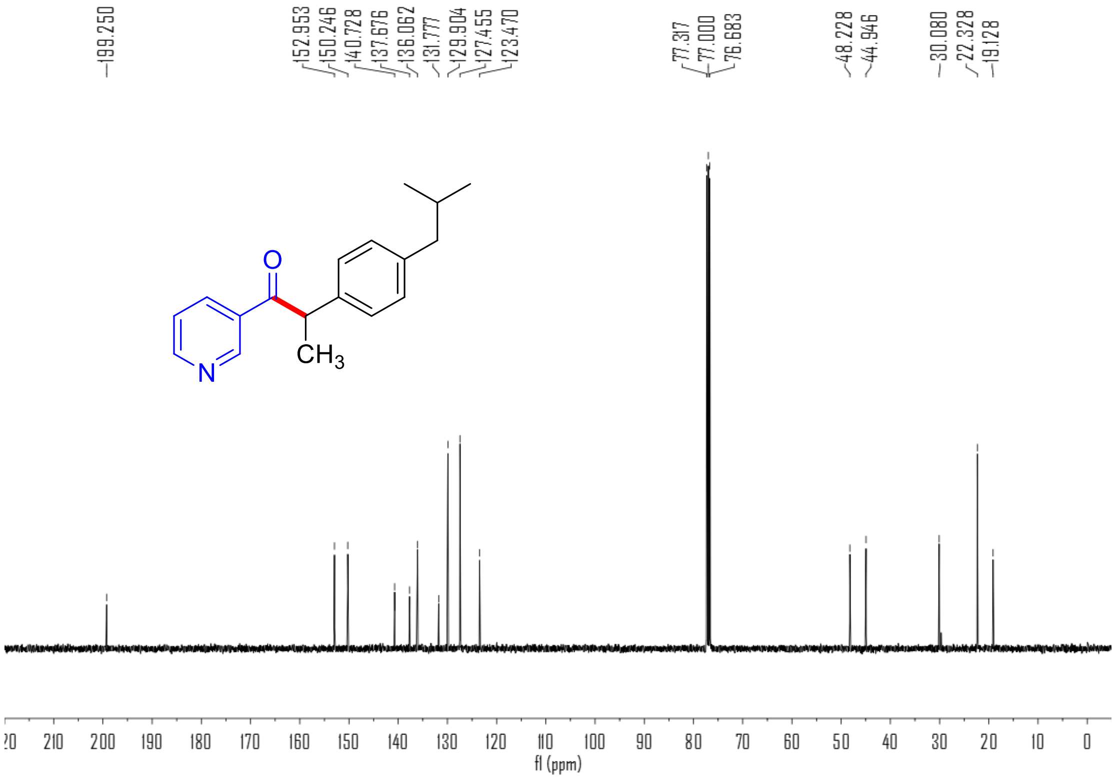

Supplementary Figure 47. ^{13}**C NMR** spectrum of **3s**

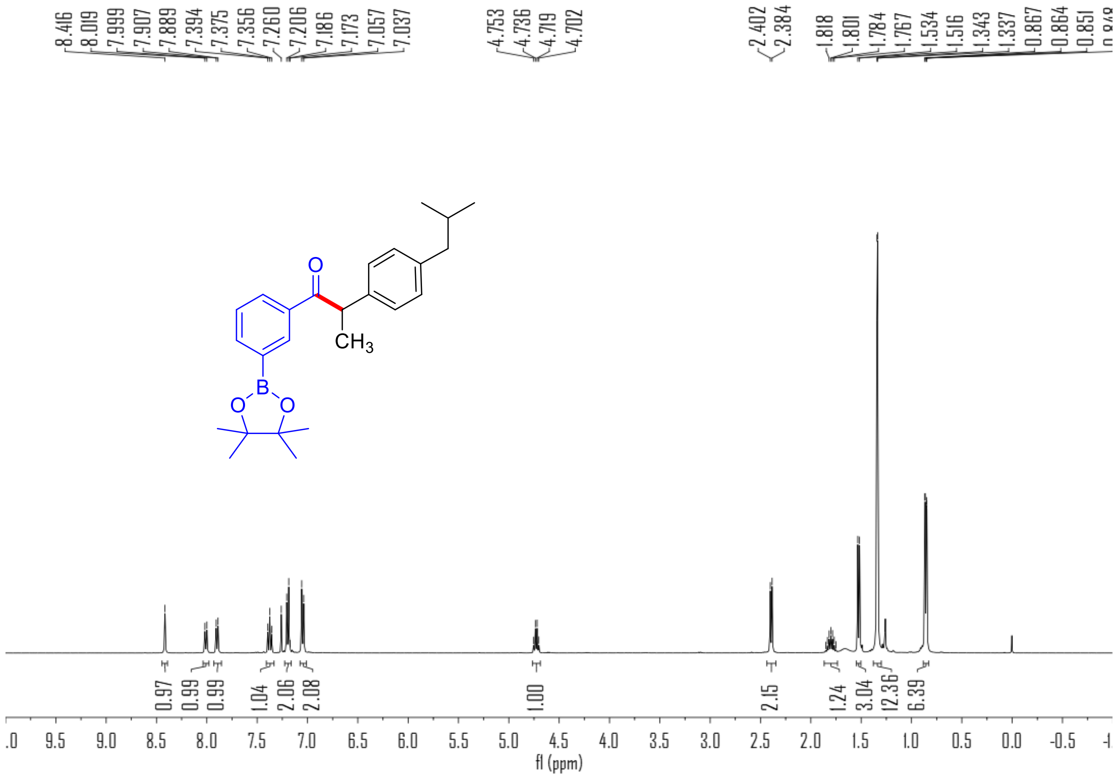

Supplementary Figure 48. ^{1}**H NMR** spectrum of **3t**

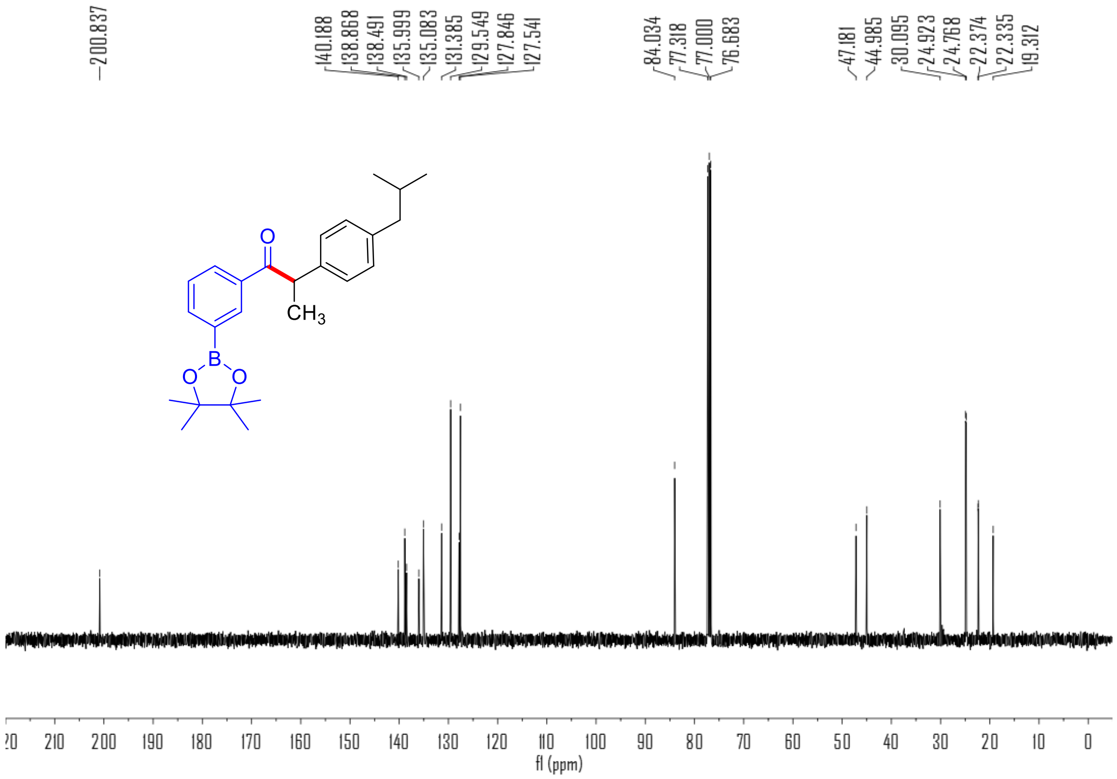

Supplementary Figure 49. ^{13}**C NMR** spectrum of **3t**

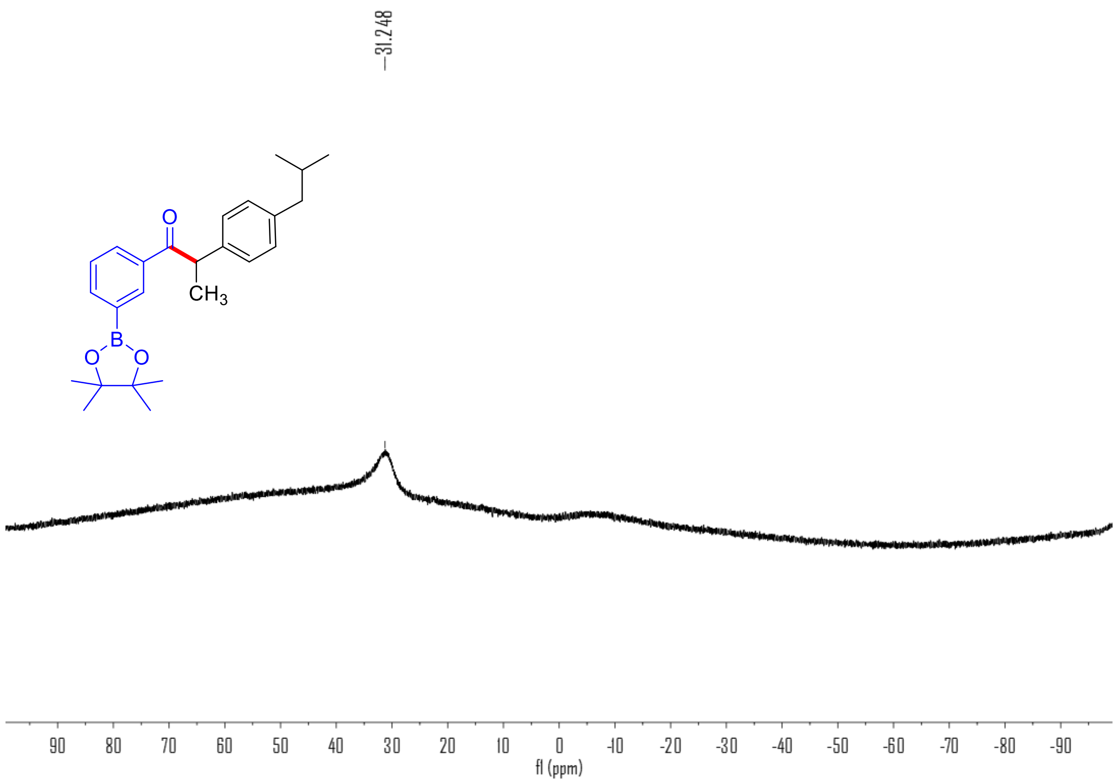

Supplementary Figure 50. ^{11}**B NMR** spectrum of **3t**

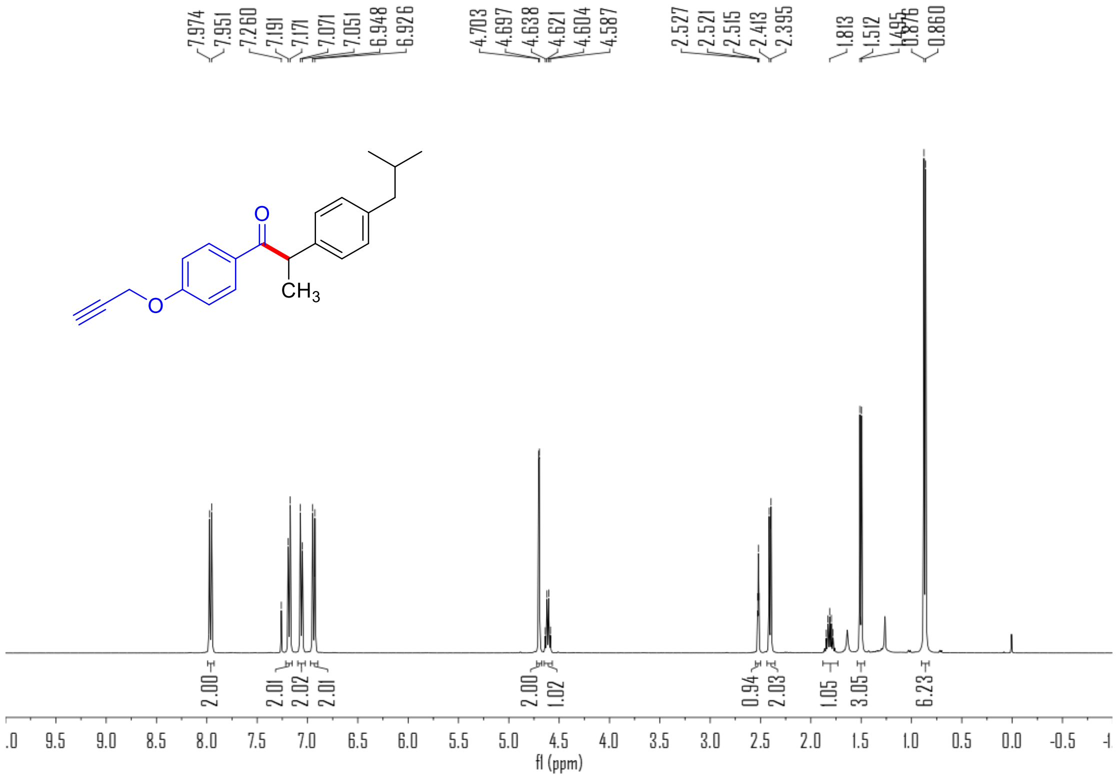

Supplementary Figure 51. ^{1}H NMR spectrum of **3u**

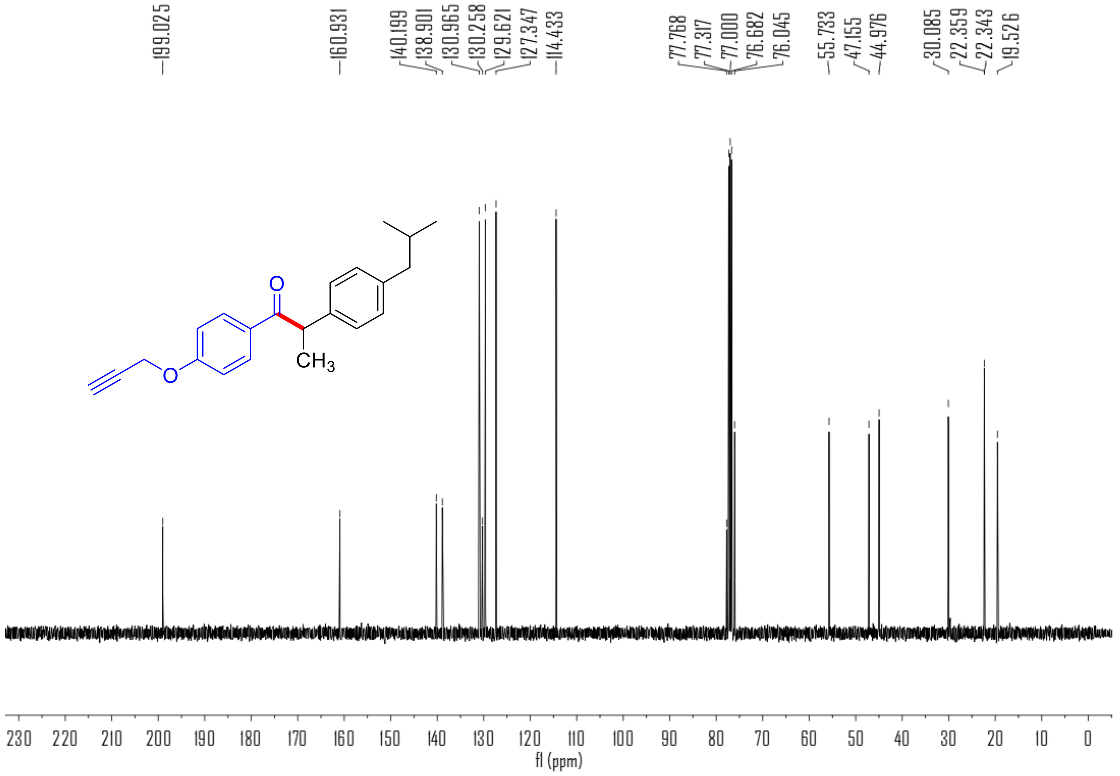

Supplementary Figure 52. ^{13}C NMR spectrum of **3u**

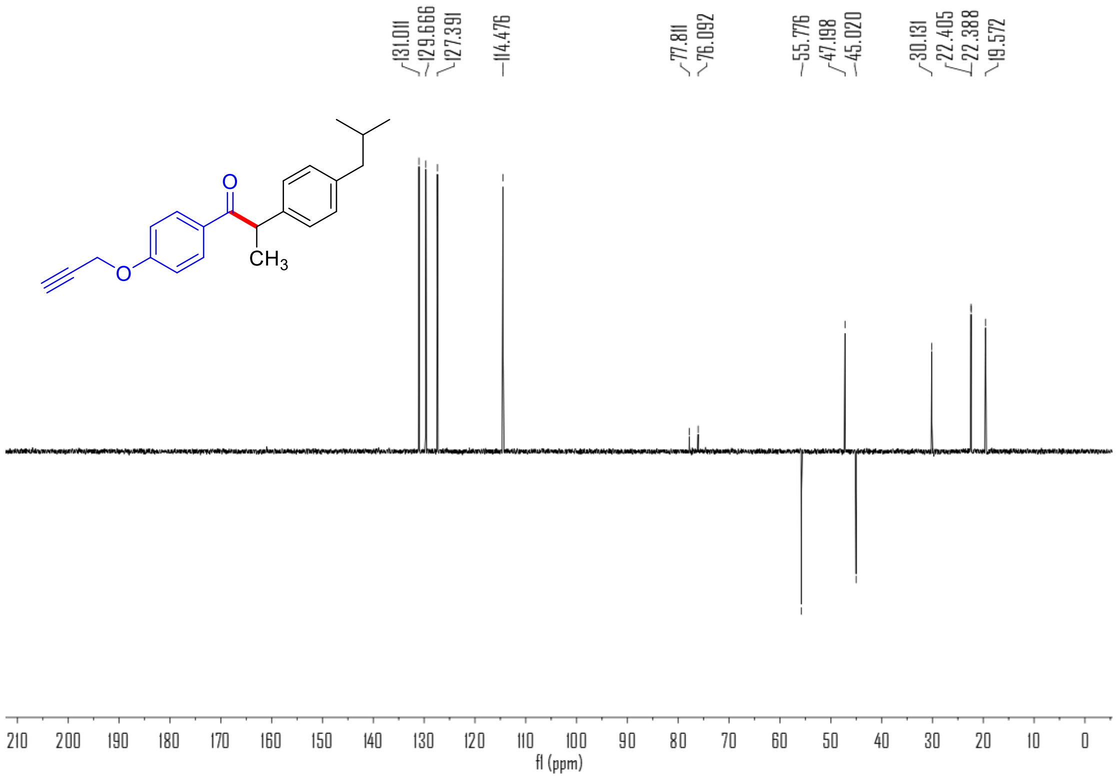

Supplementary Figure 53. DEPT 135 spectrum of **3u**

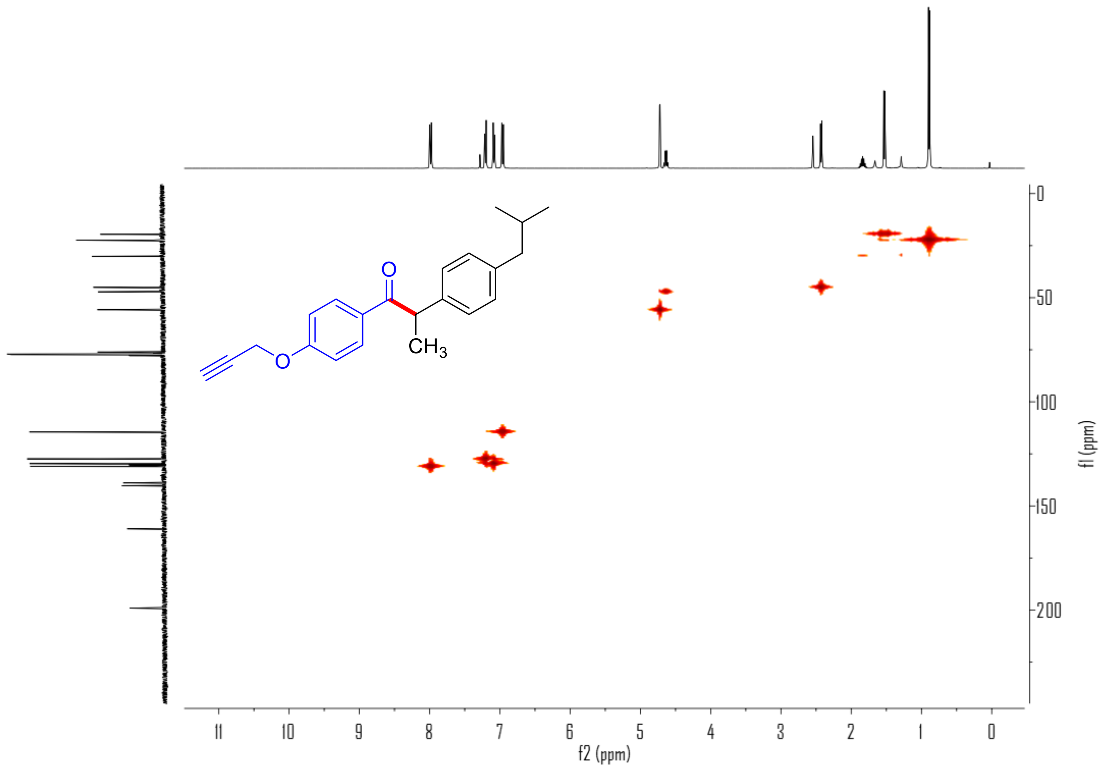

Supplementary Figure 54. HMQC spectrum of **3u**

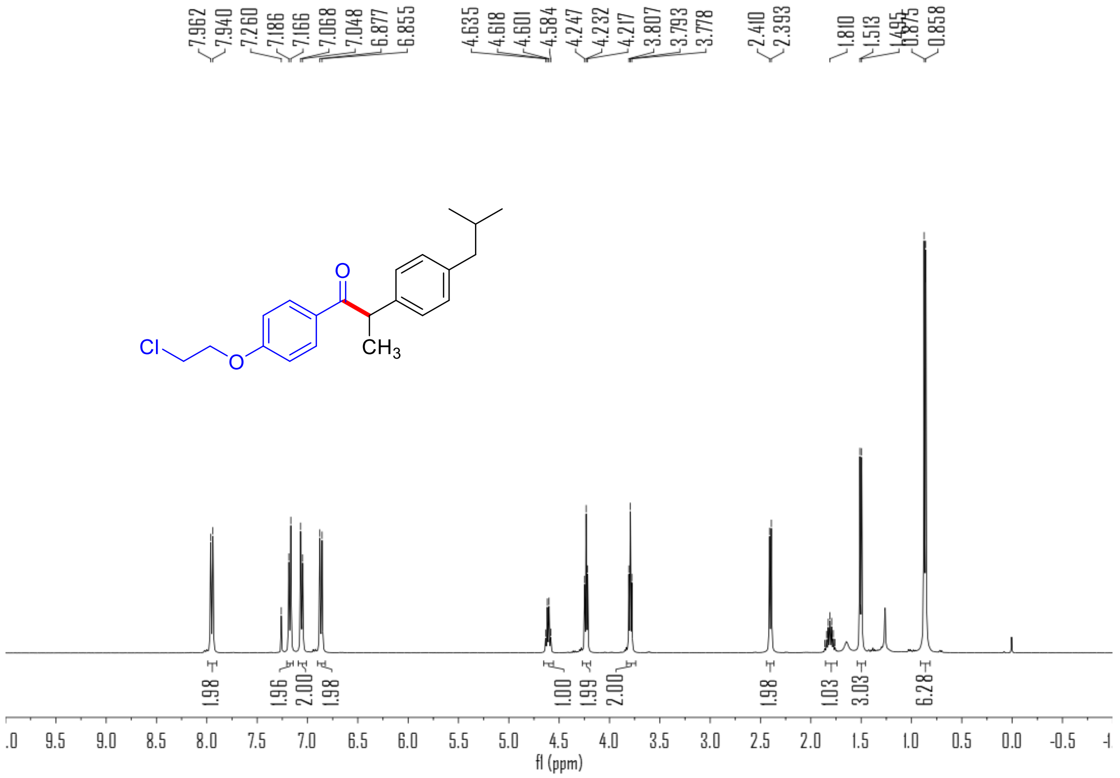

Supplementary Figure 55. ^{1}**H** spectrum of **3v**

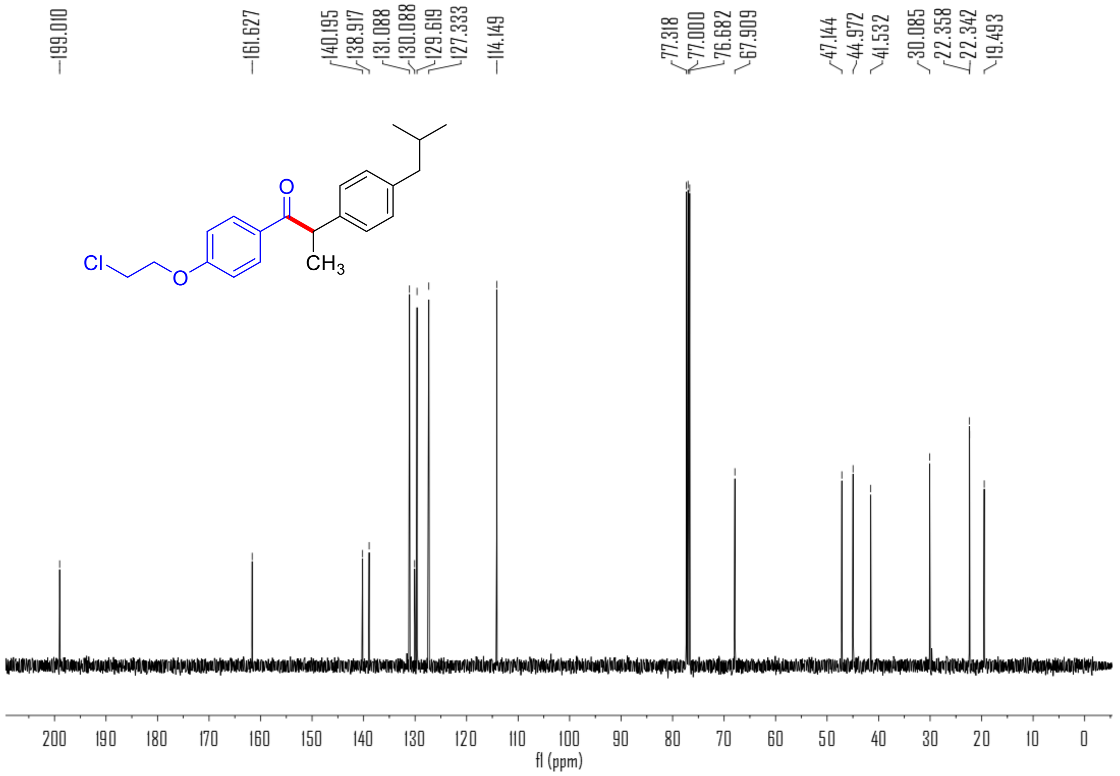

Supplementary Figure 56. ^{13}**C** spectrum of **3v**

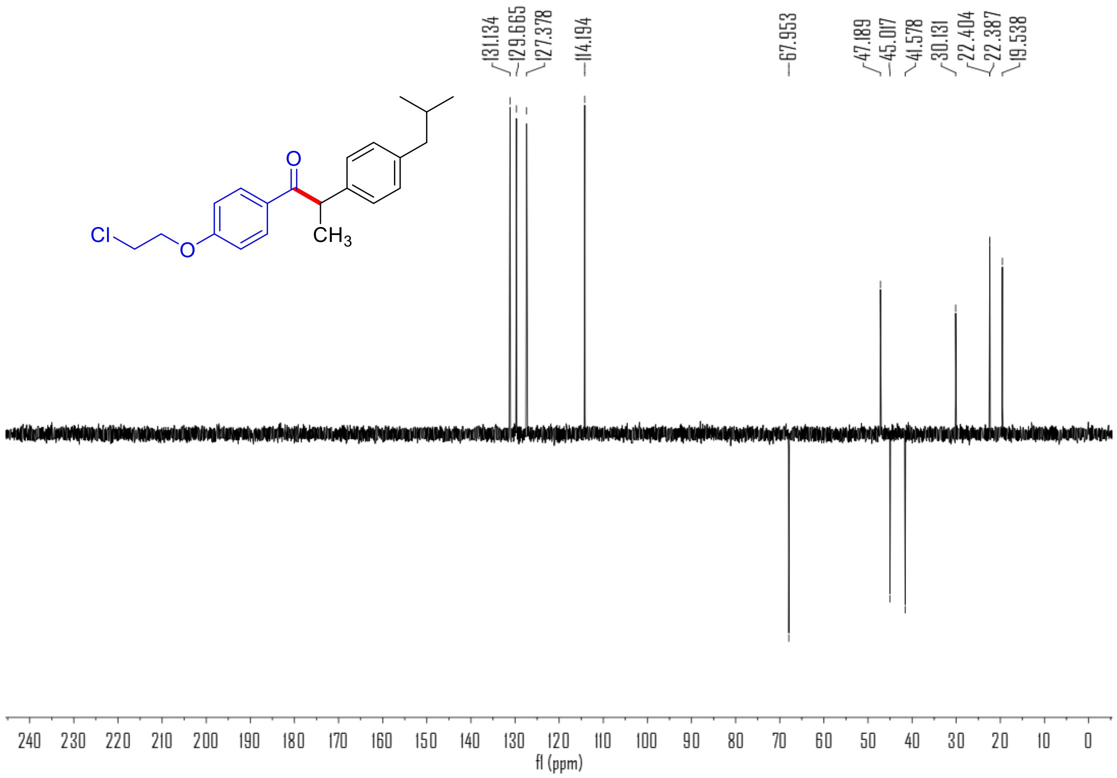

Supplementary Figure 57. DEPT 135 spectrum of **3v**

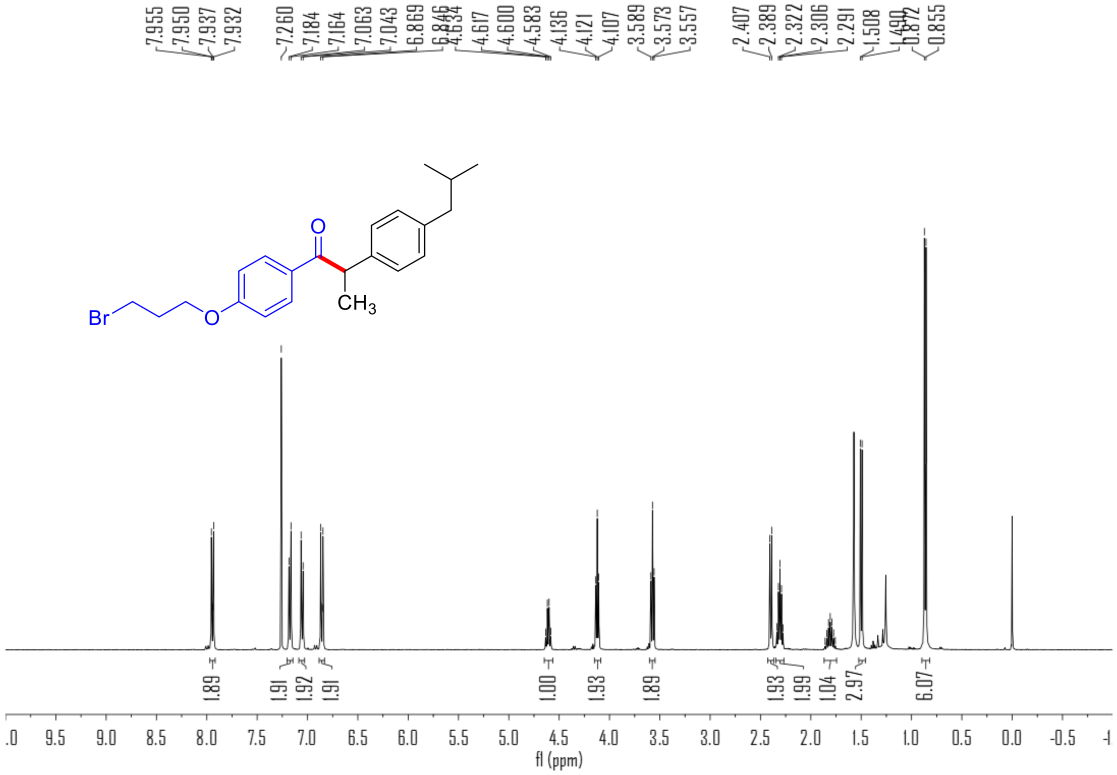

Supplementary Figure 58. ^{1}**H** spectrum of **3w**

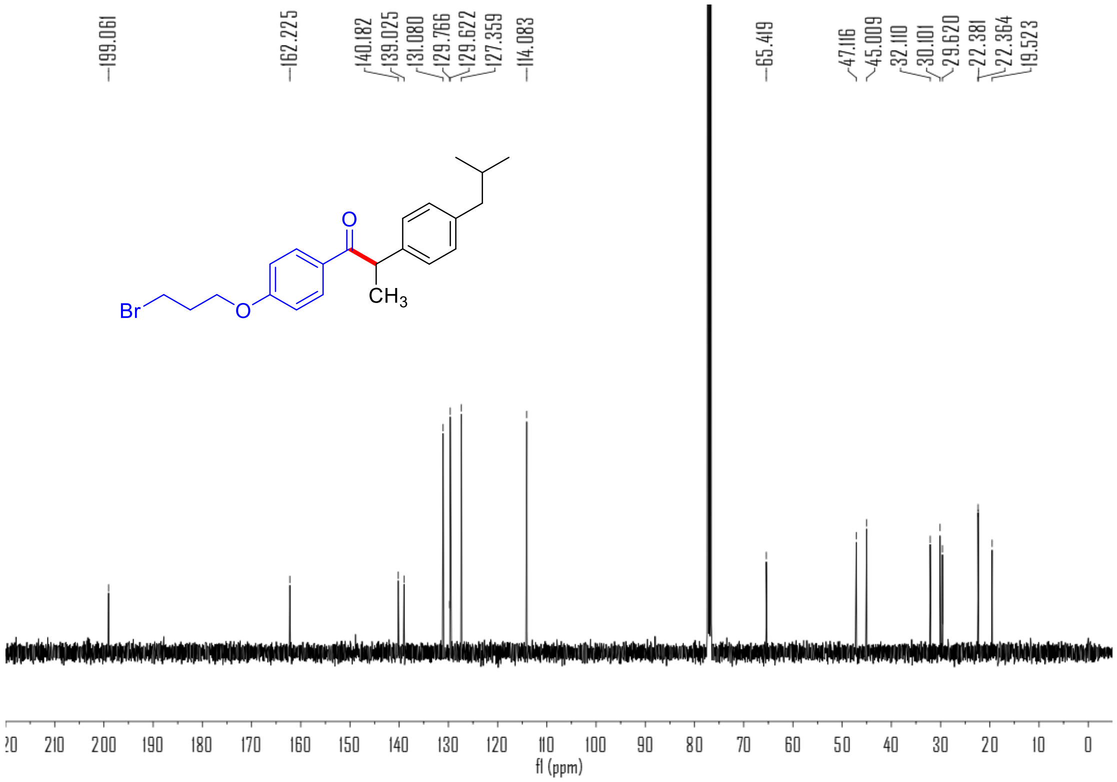

Supplementary Figure 59. ^{13}**C** spectrum of **3w**

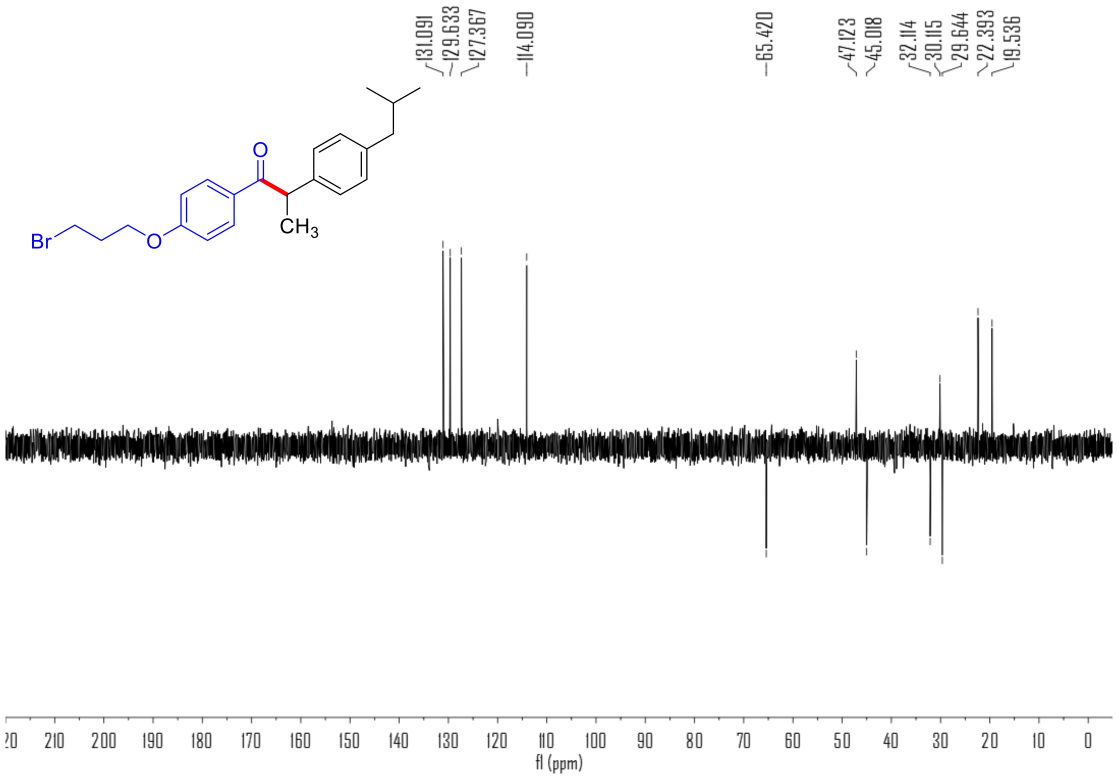

Supplementary Figure 60. DEPT 135 spectrum of **3w**

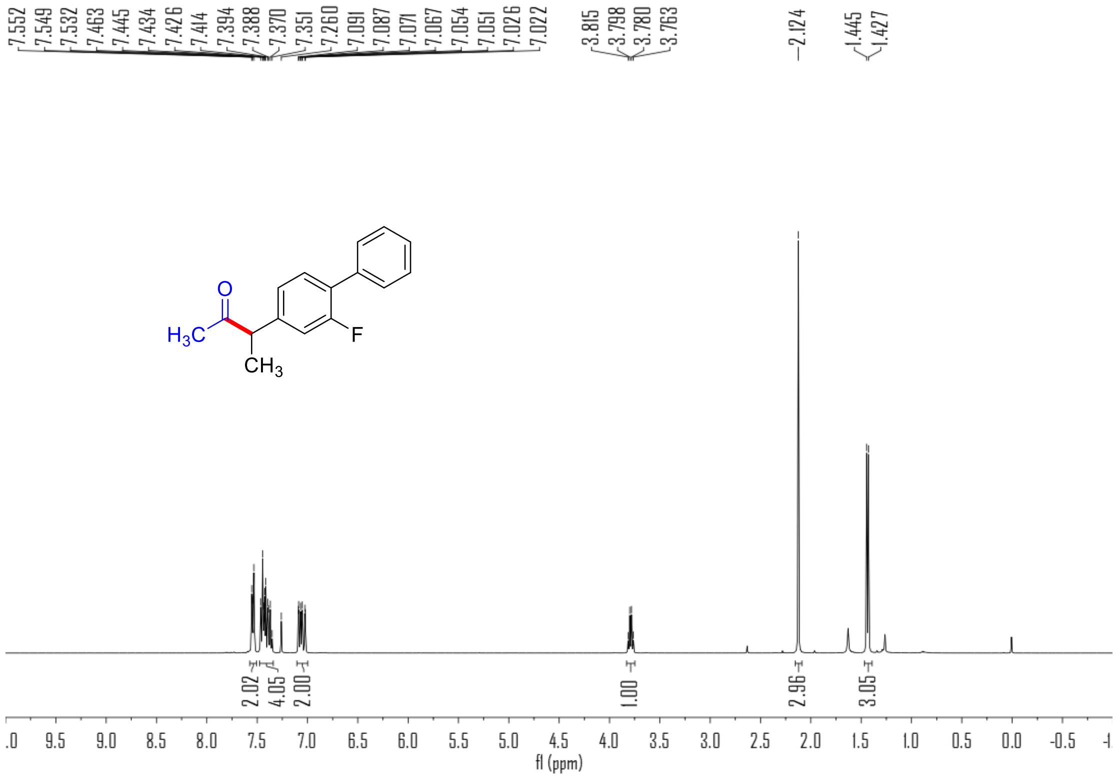

Supplementary Figure 61. ^{1}**H** spectrum of **3x**

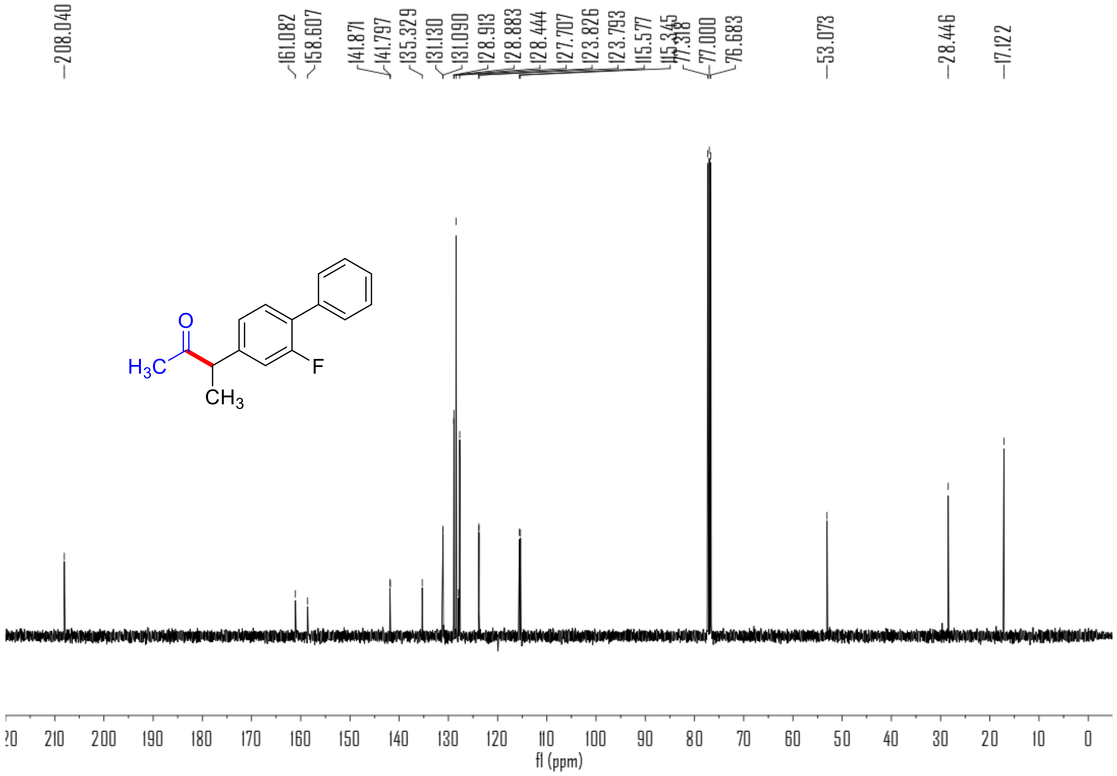

Supplementary Figure 62. ^{13}**C** spectrum of **3x**

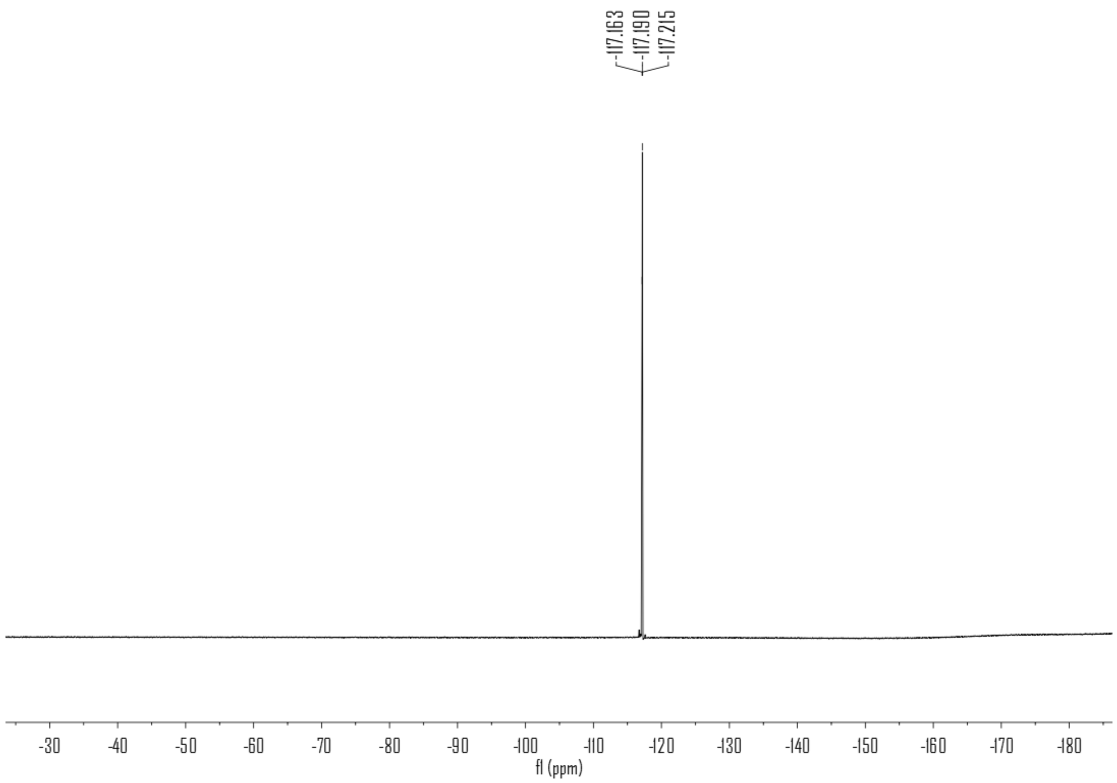

Supplementary Figure 63. ^{19}F spectrum of **3x**

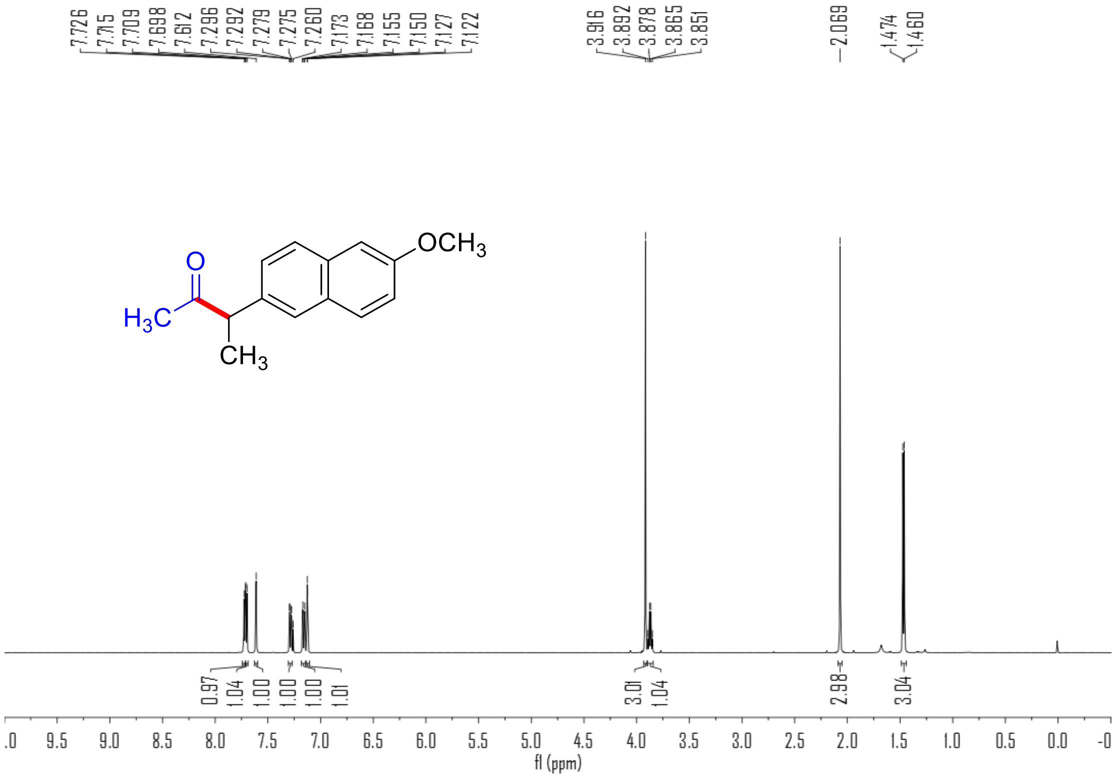

Supplementary Figure 64. **^{1}H NMR** spectrum of **3y**

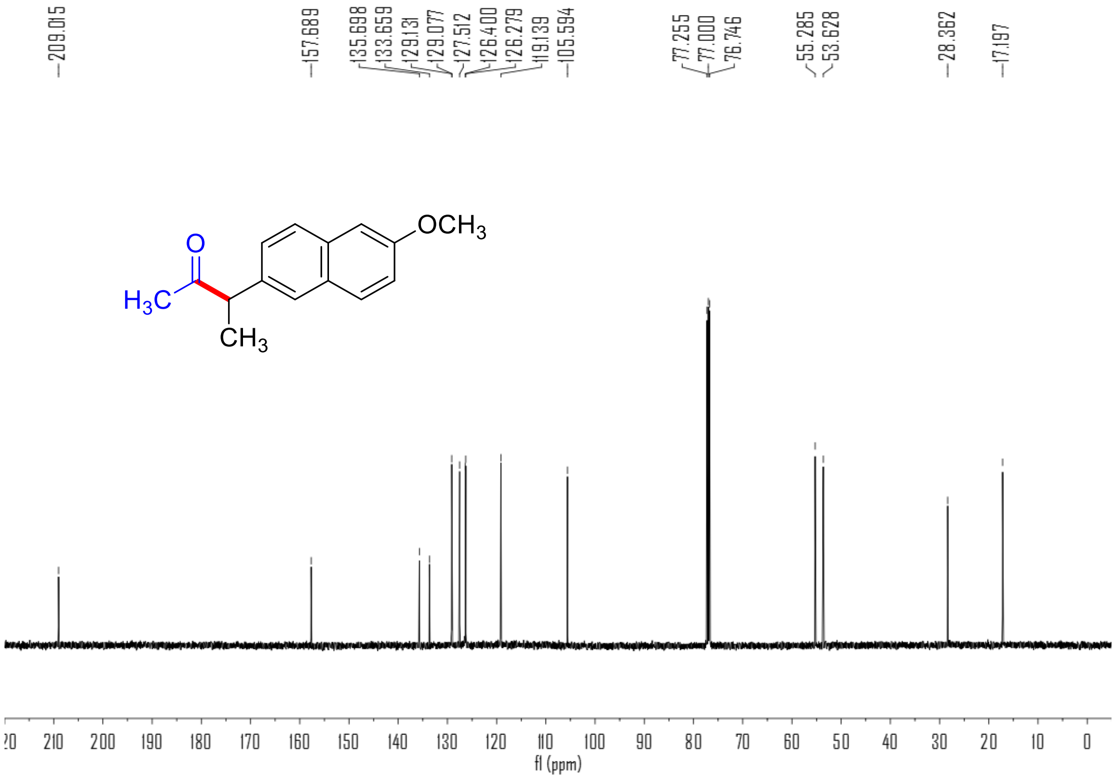

Supplementary Figure 65. **^{13}C NMR** spectrum of **3y**

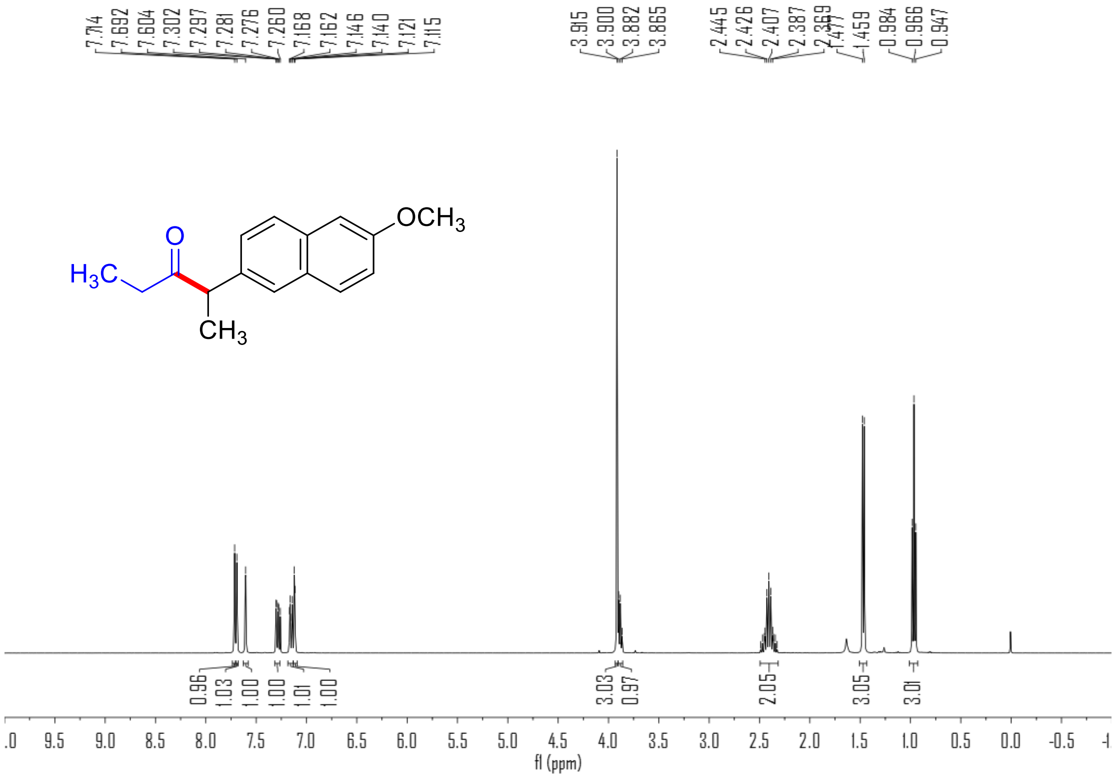

Supplementary Figure 66. ^{1}**H NMR** spectrum of **3z**

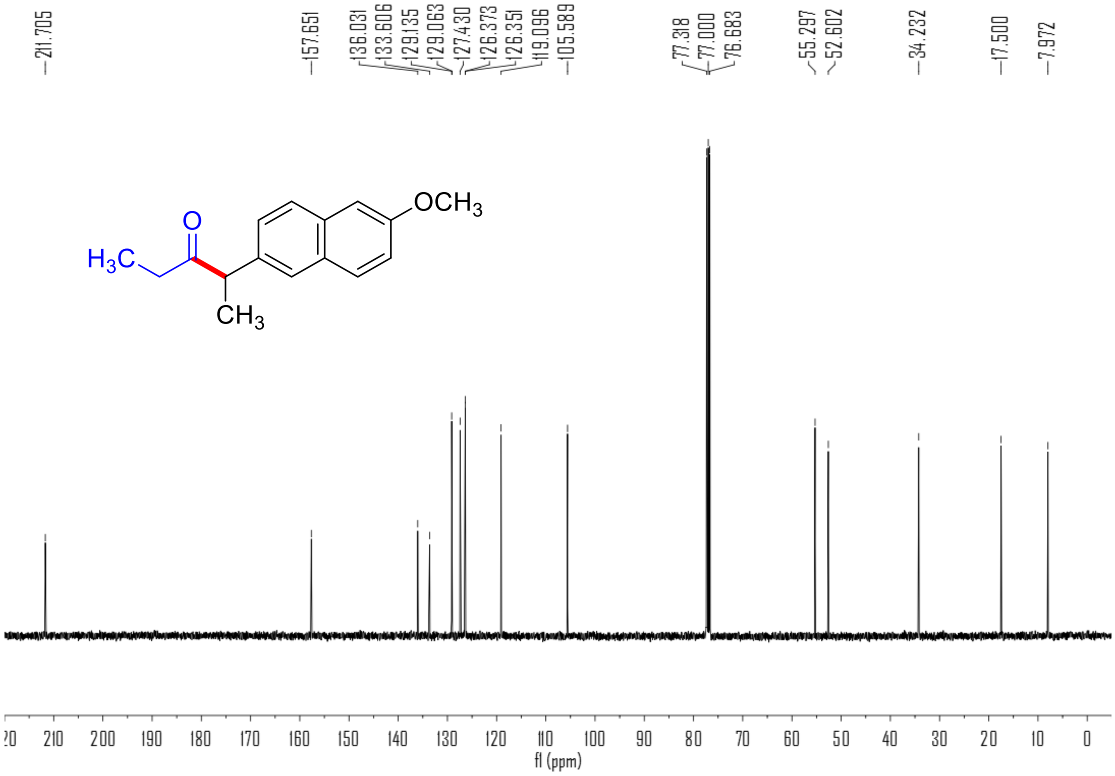

Supplementary Figure 67. ^{13}**C NMR** spectrum of **3z**

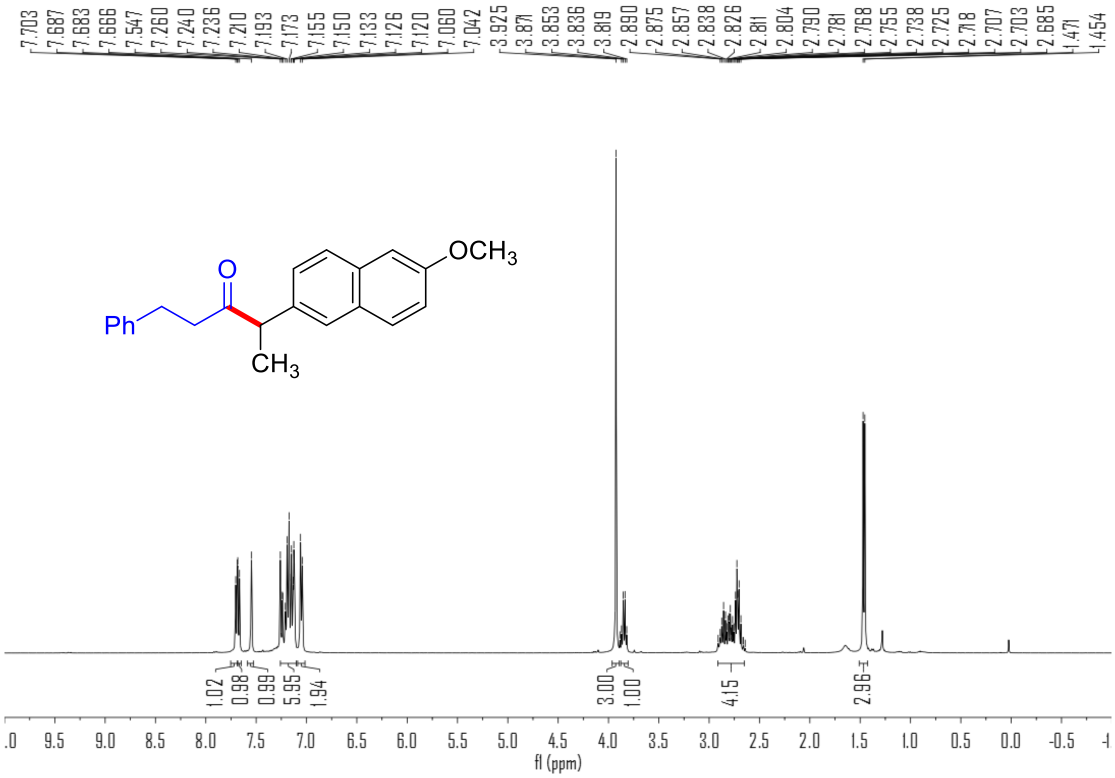

Supplementary Figure 68. ^{1}**H NMR** spectrum of **3aa**

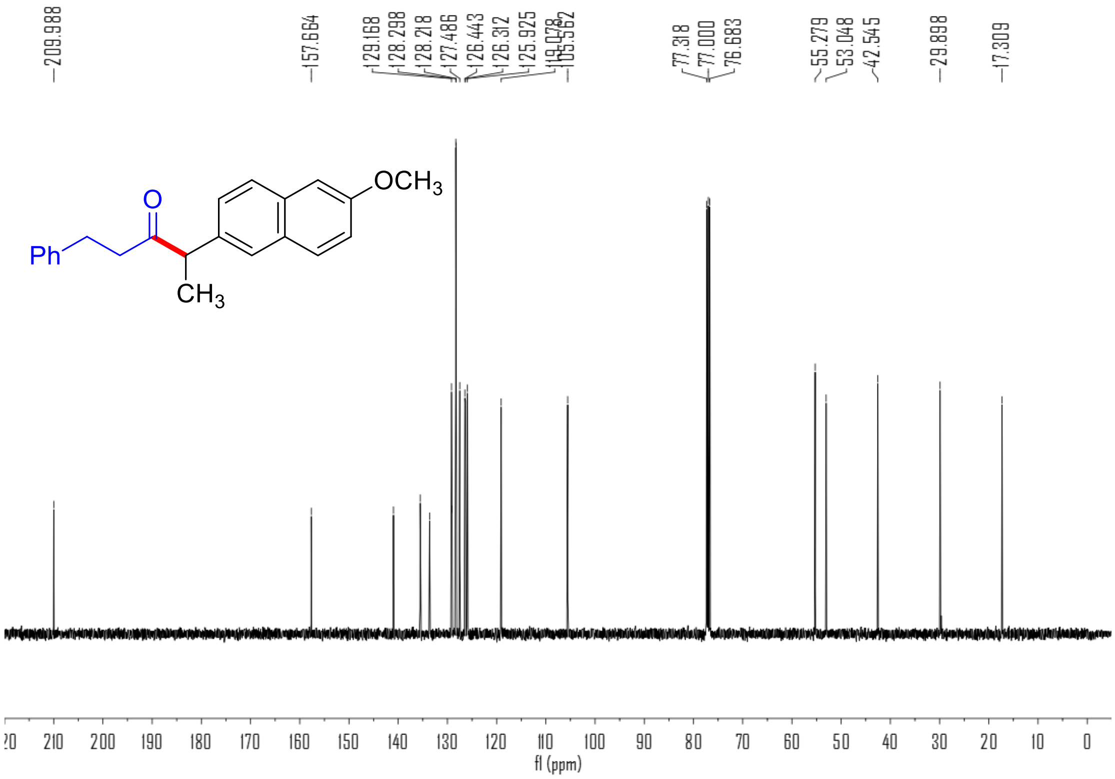

Supplementary Figure 69. ^{13}**C NMR** spectrum of **3aa**

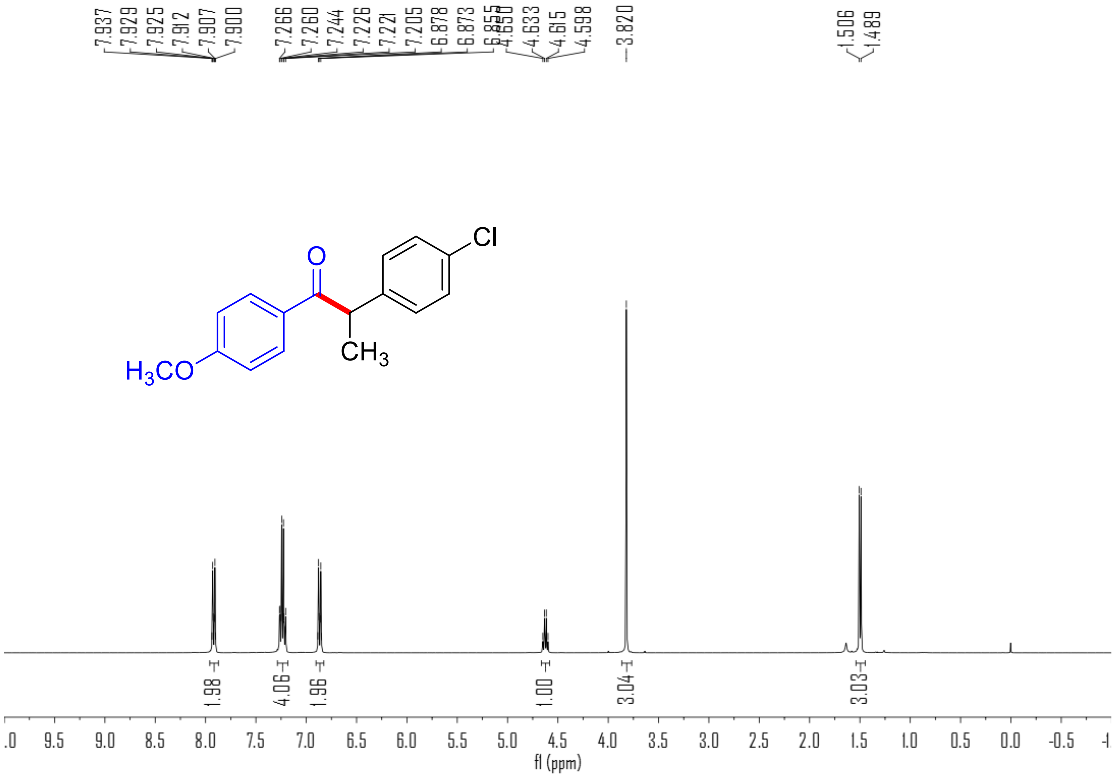

Supplementary Figure 70. ^{1}**H NMR** spectrum of **3ab**

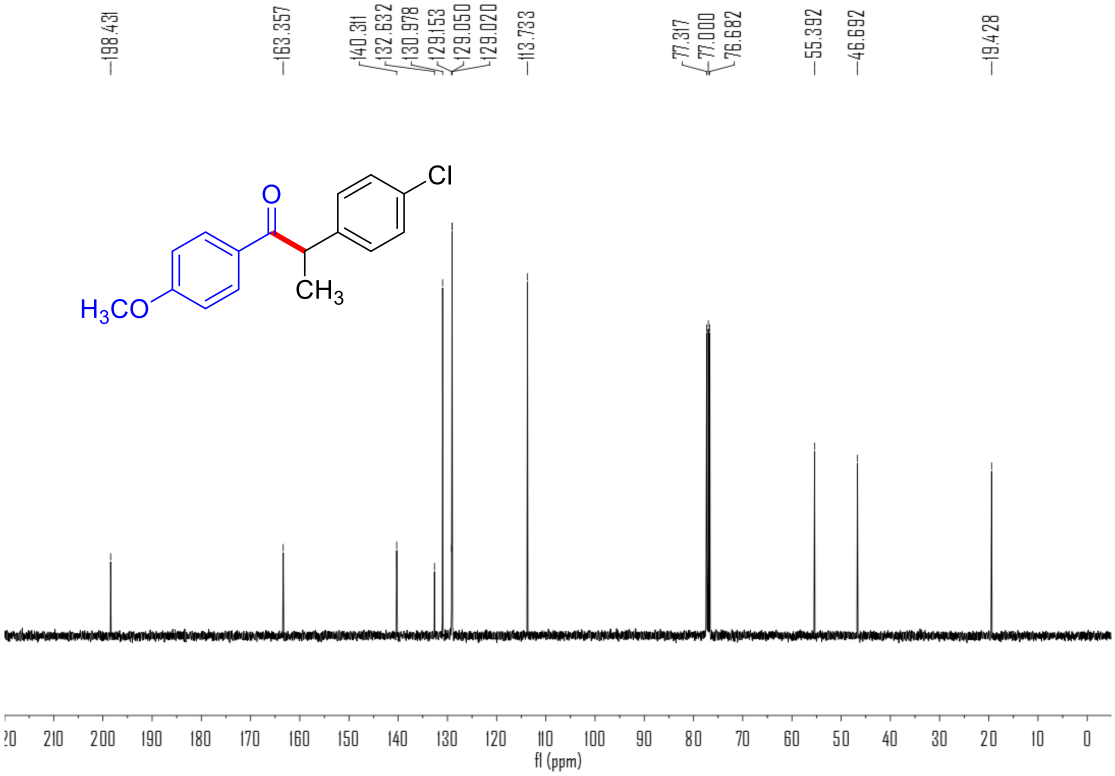

Supplementary Figure 71. ^{13}**C NMR** spectrum of **3ab**

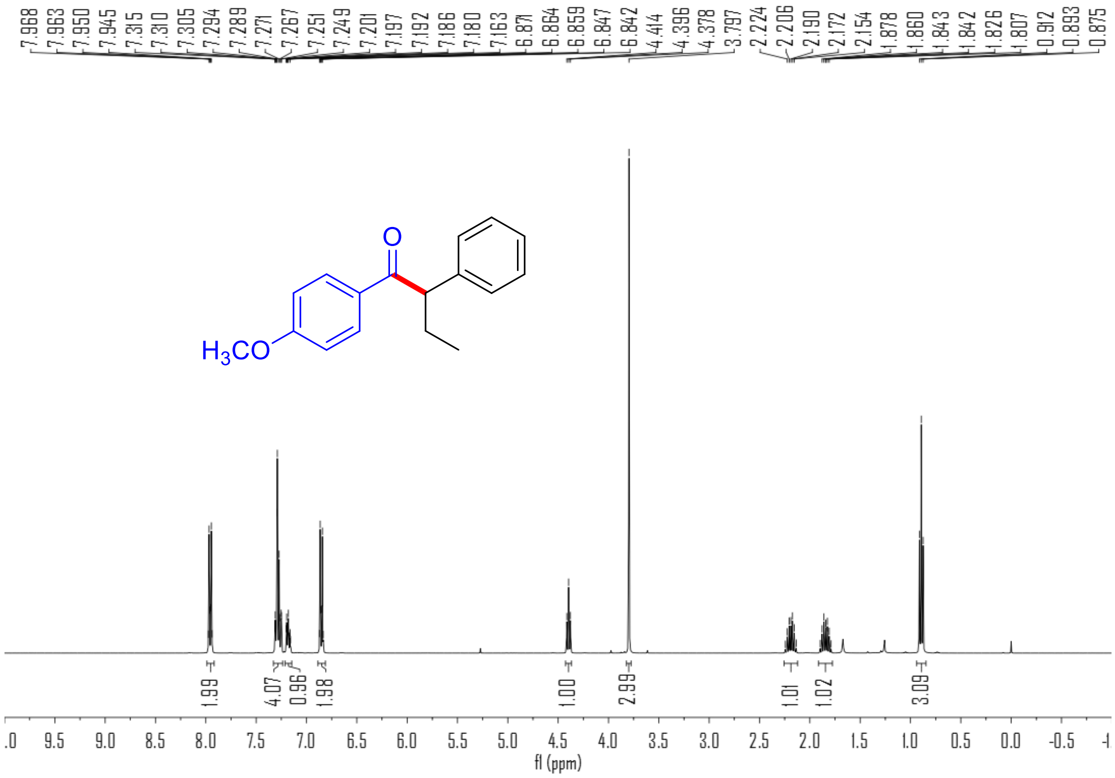

Supplementary Figure 72. ^{1}**H NMR** spectrum of **3ac**

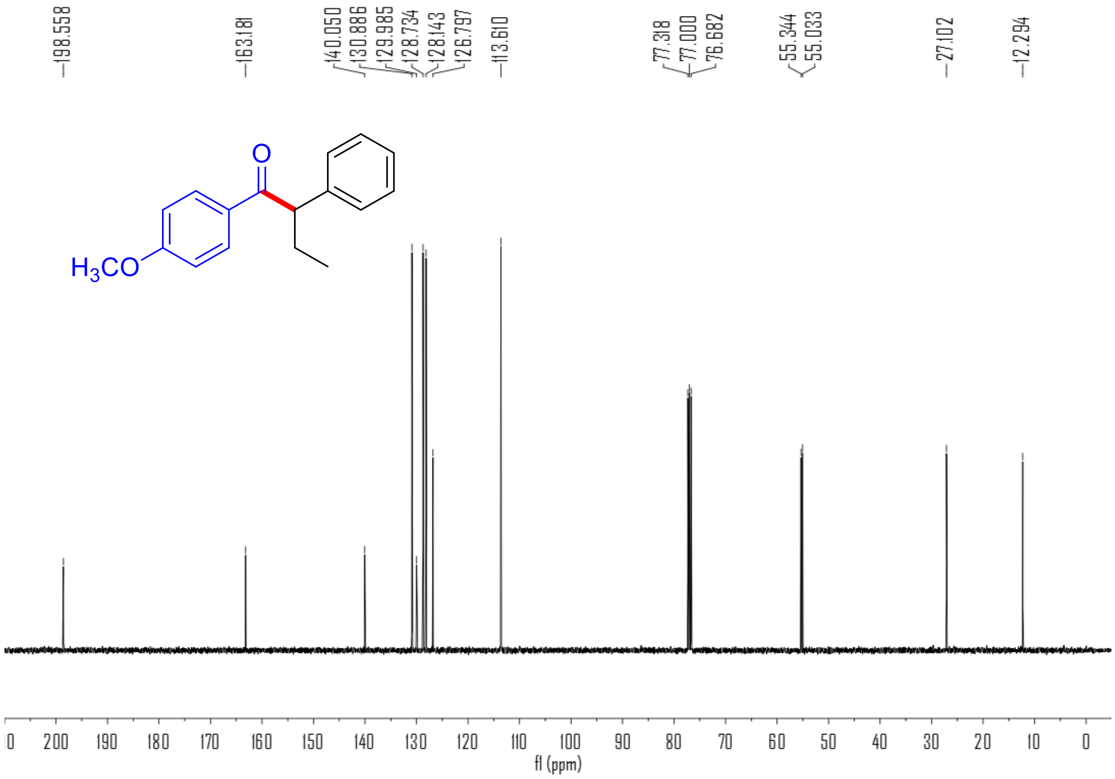

Supplementary Figure 73. ^{13}**C NMR** spectrum of **3ac**

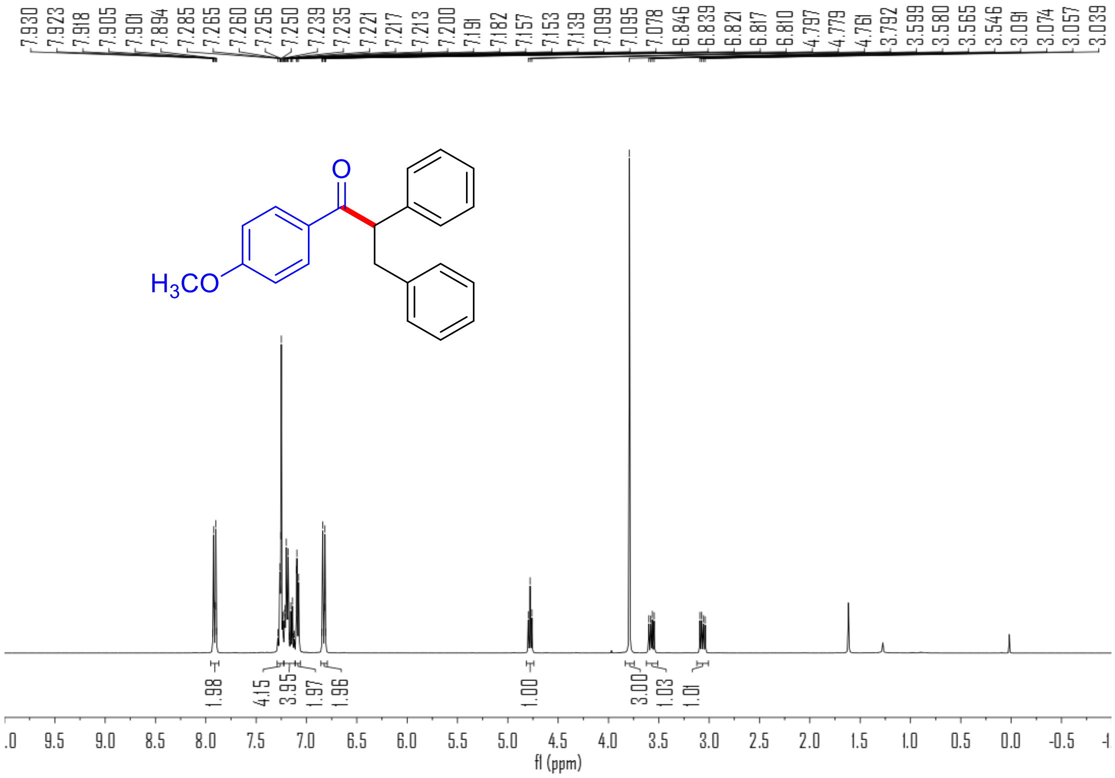

Supplementary Figure 74. ^{1}**H NMR** spectrum of **3ad**

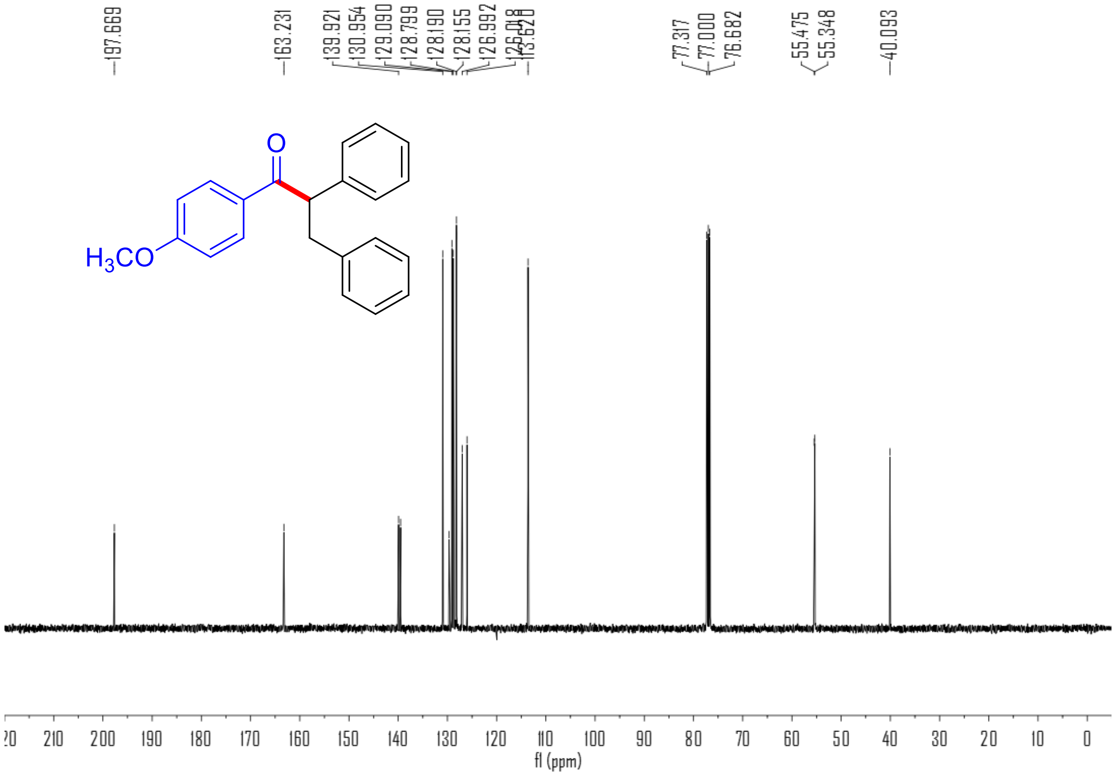

Supplementary Figure 75. ^{13}**C NMR** spectrum of **3ad**

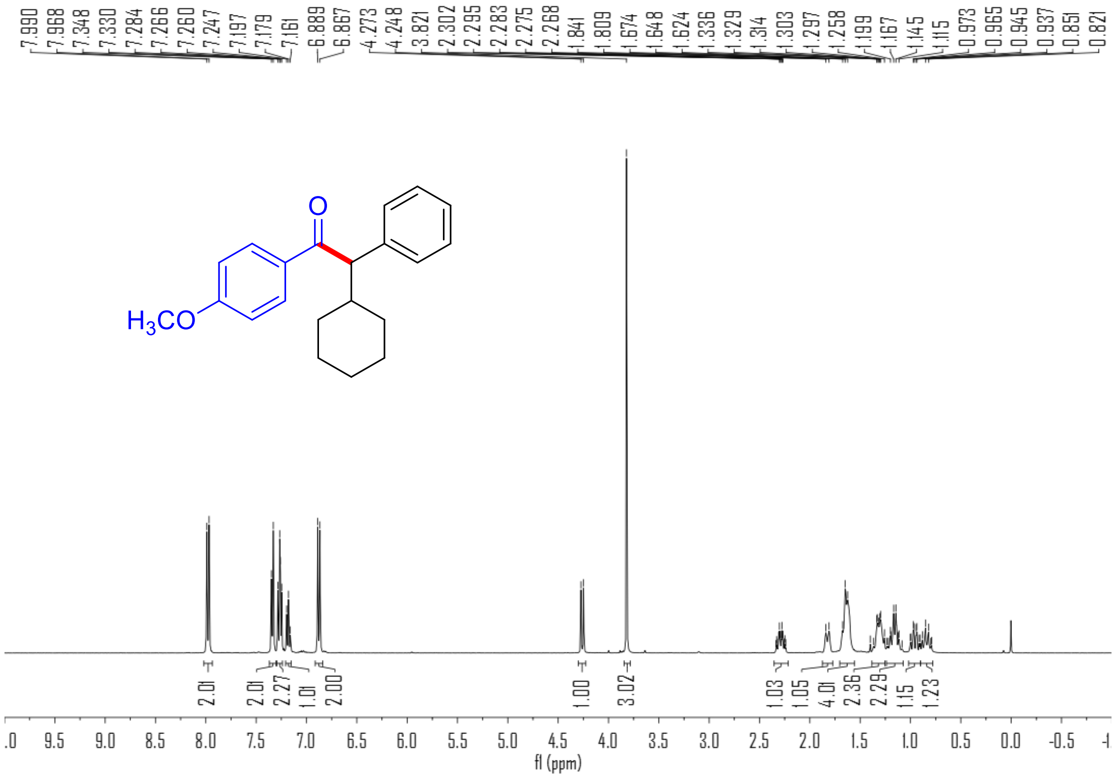

Supplementary Figure 76. ^{1}**H NMR** spectrum of **3ae**

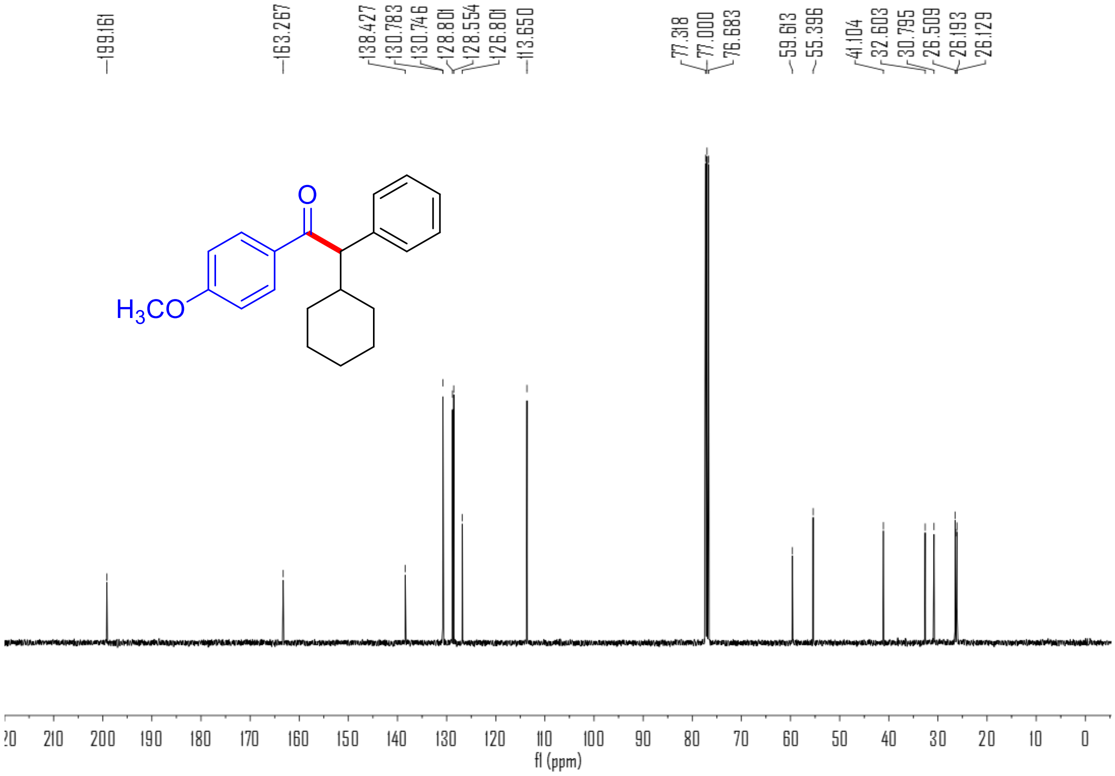

Supplementary Figure 77. ^{13}**C NMR** spectrum of **3ae**

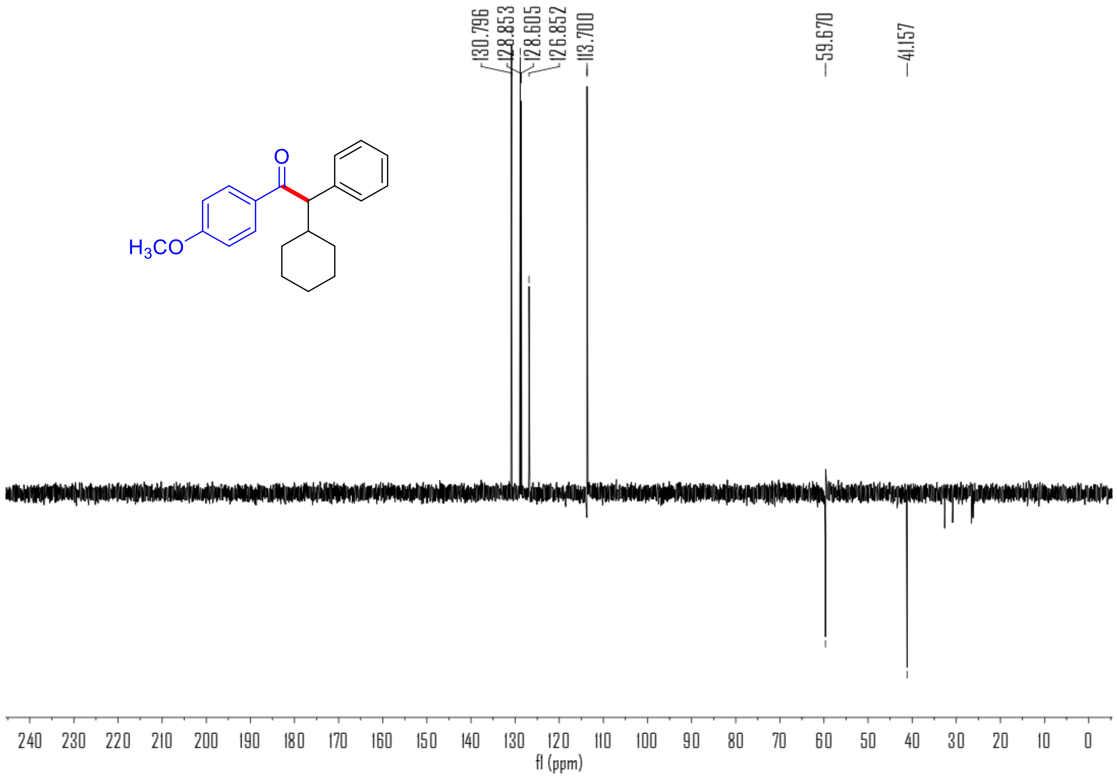

Supplementary Figure 78. DEPT90 spectrum of **3ae**

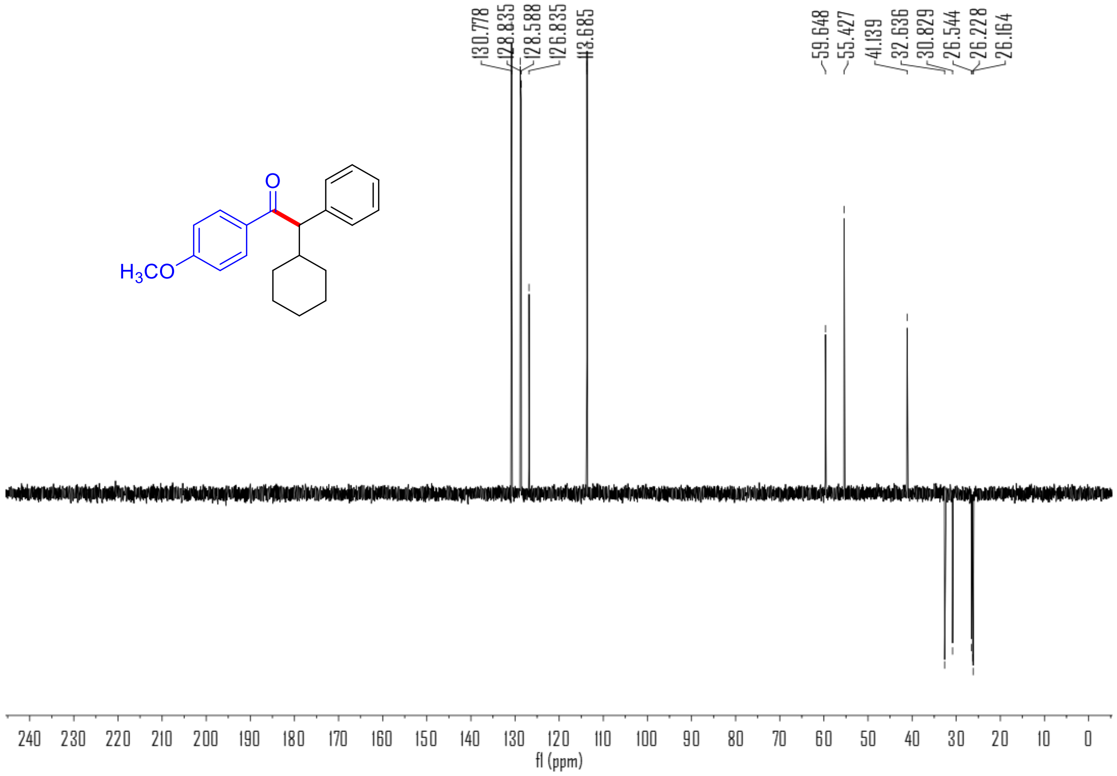

Supplementary Figure 79. DEPT135 spectrum of **3ae**

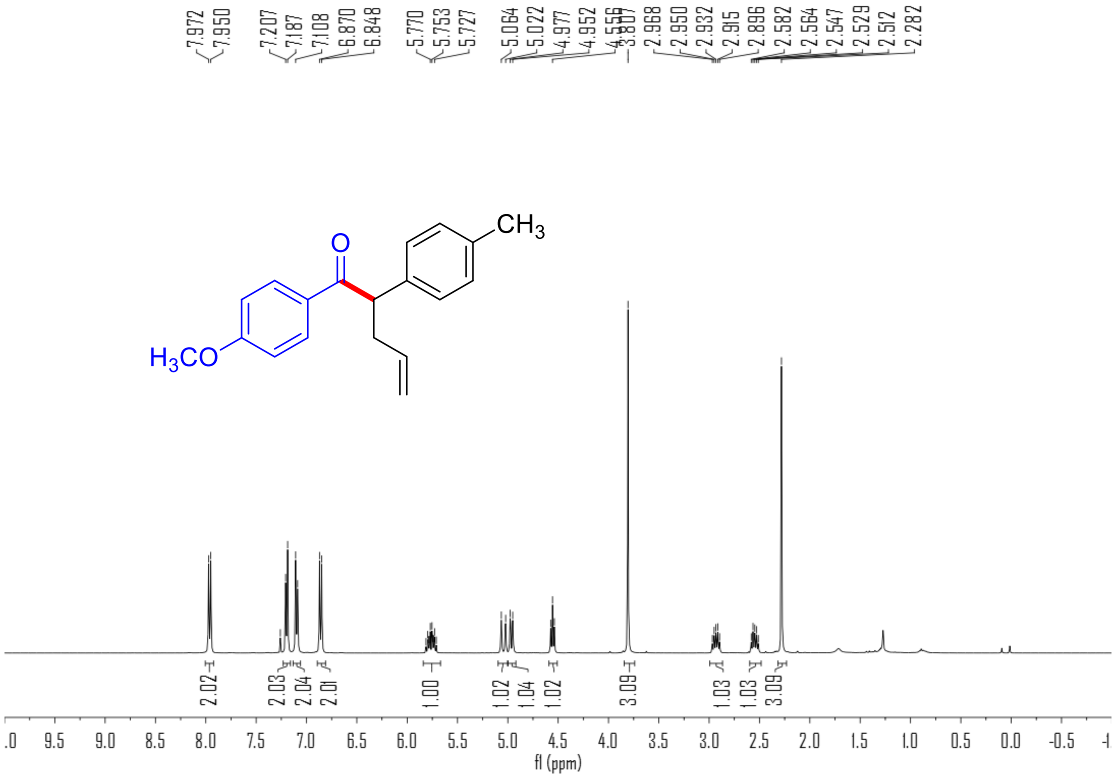

Supplementary Figure 80. ^{1}**H NMR** spectrum of **3af**

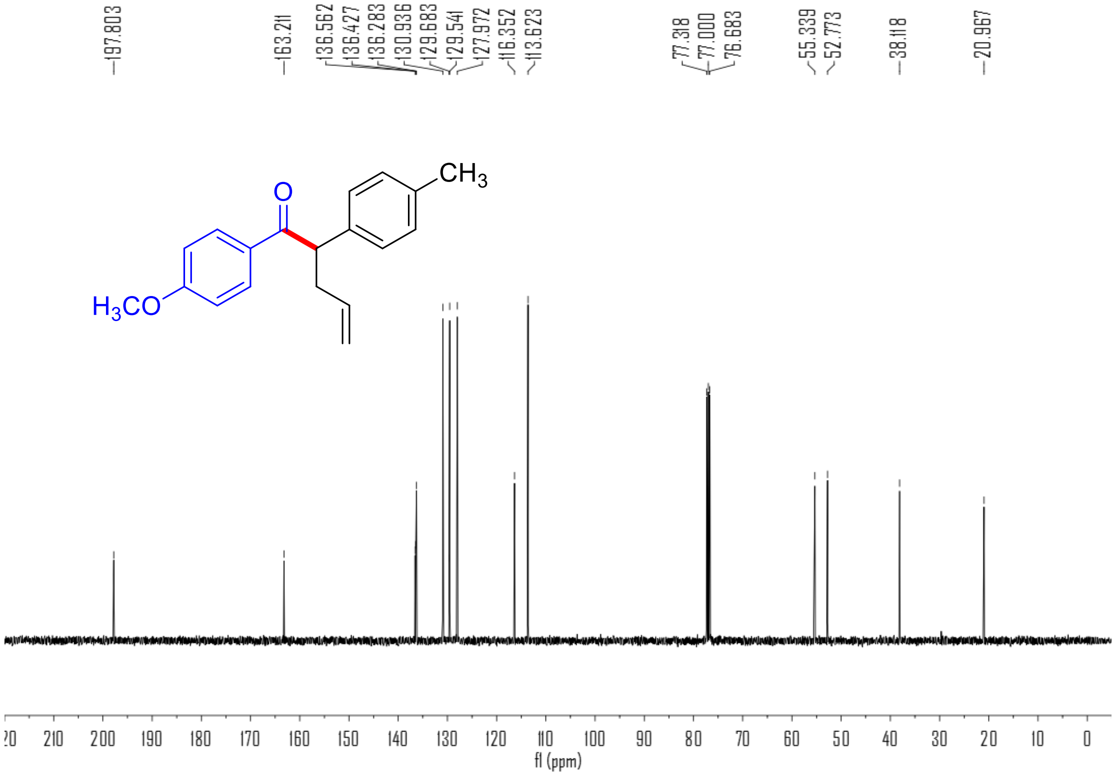

Supplementary Figure 81. ^{13}**C NMR** spectrum of **3af**

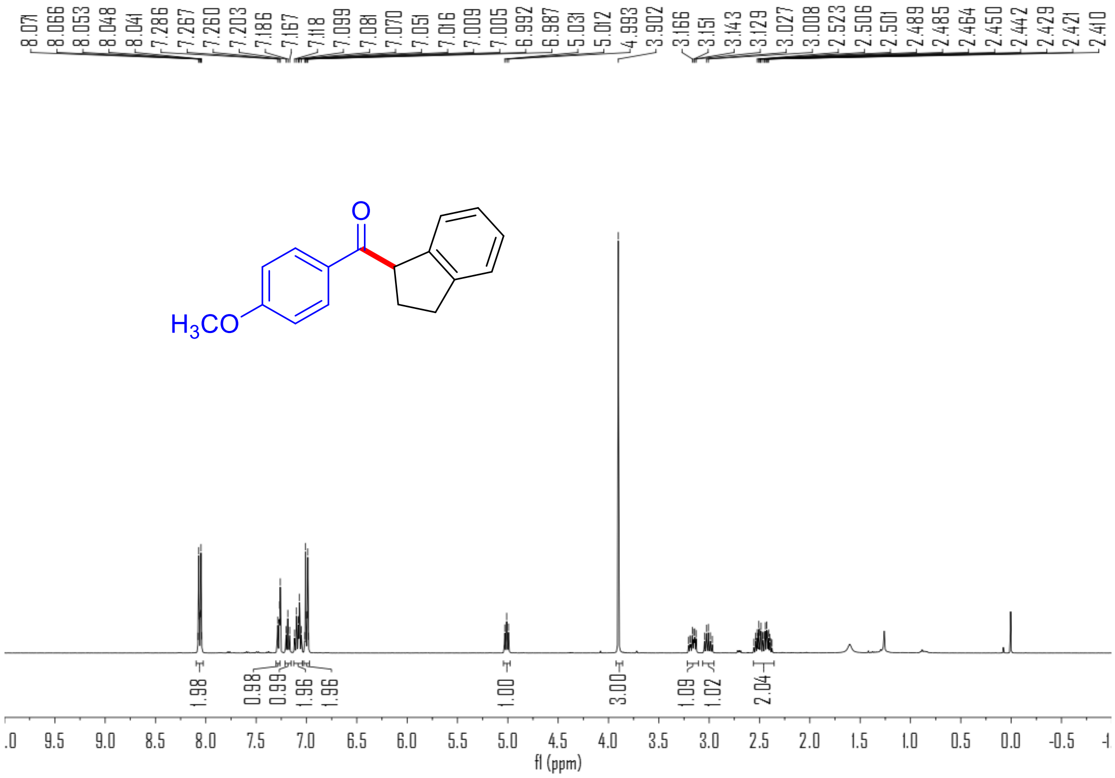

Supplementary Figure 82. ^{1}**H NMR** spectrum of **3ag**

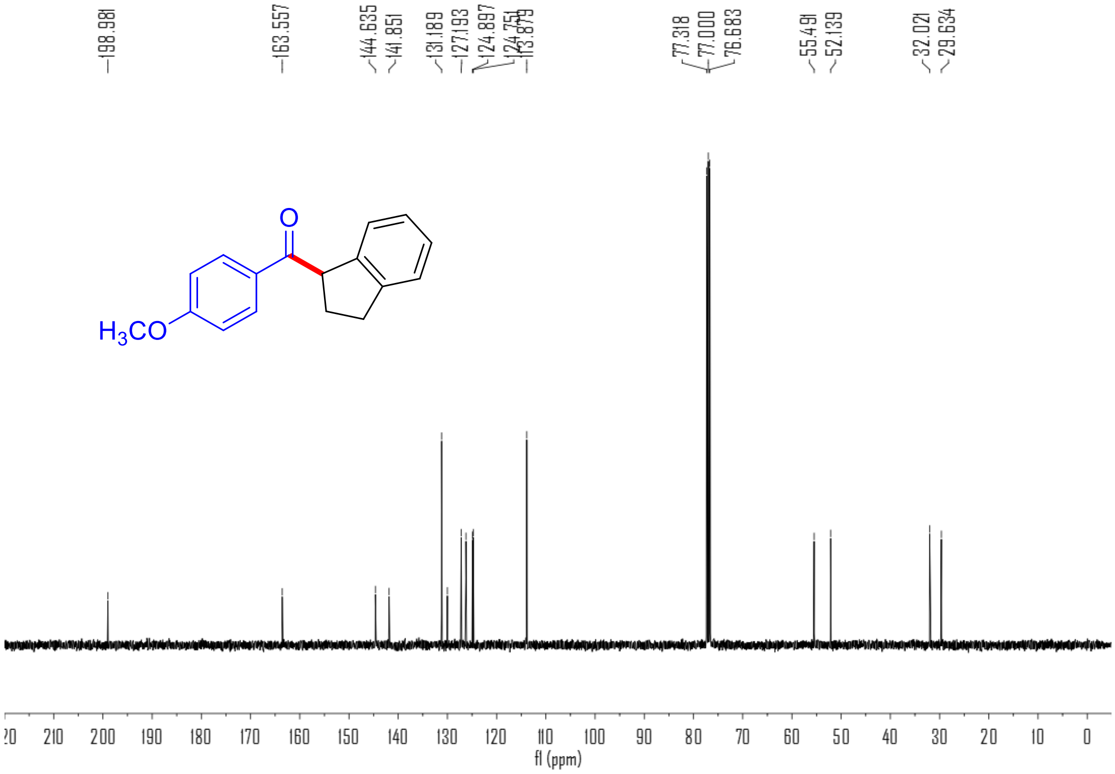

Supplementary Figure 83. ^{13}**C NMR** spectrum of **3ag**

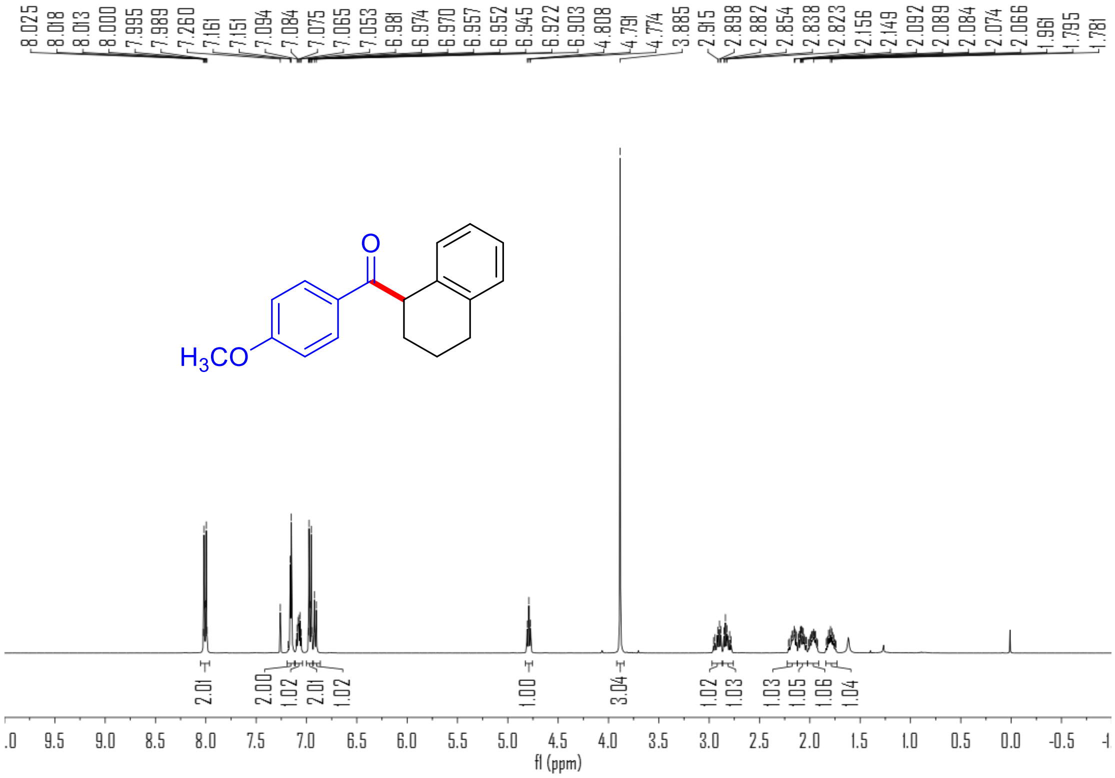

Supplementary Figure 84. ^{1}**H NMR** spectrum of **3ah**

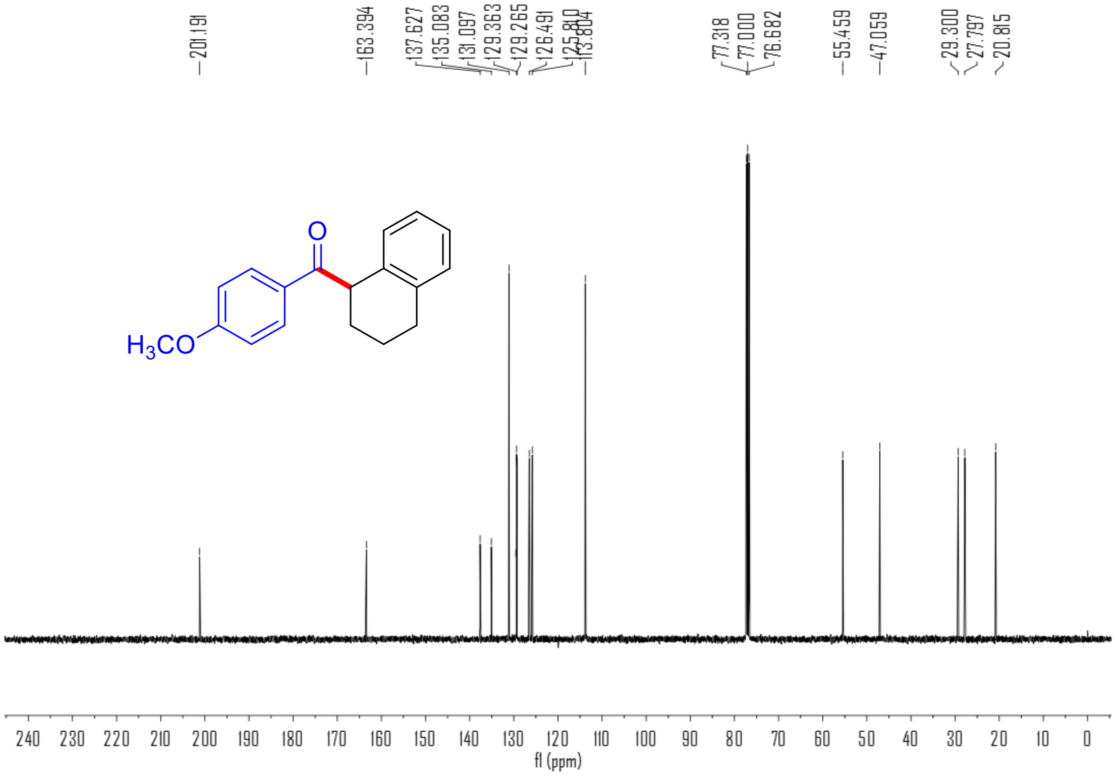

Supplementary Figure 85. ^{13}**C NMR** spectrum of **3ah**

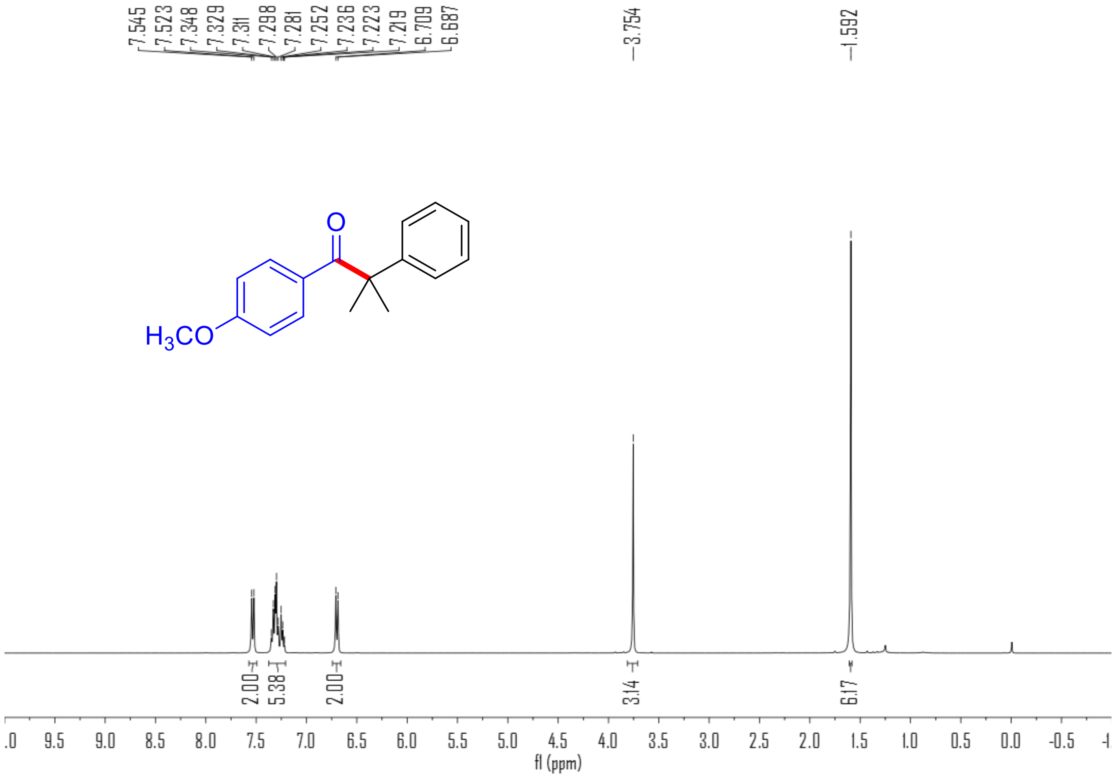

Supplementary Figure 86. **^{1}H NMR** spectrum of **3ai**

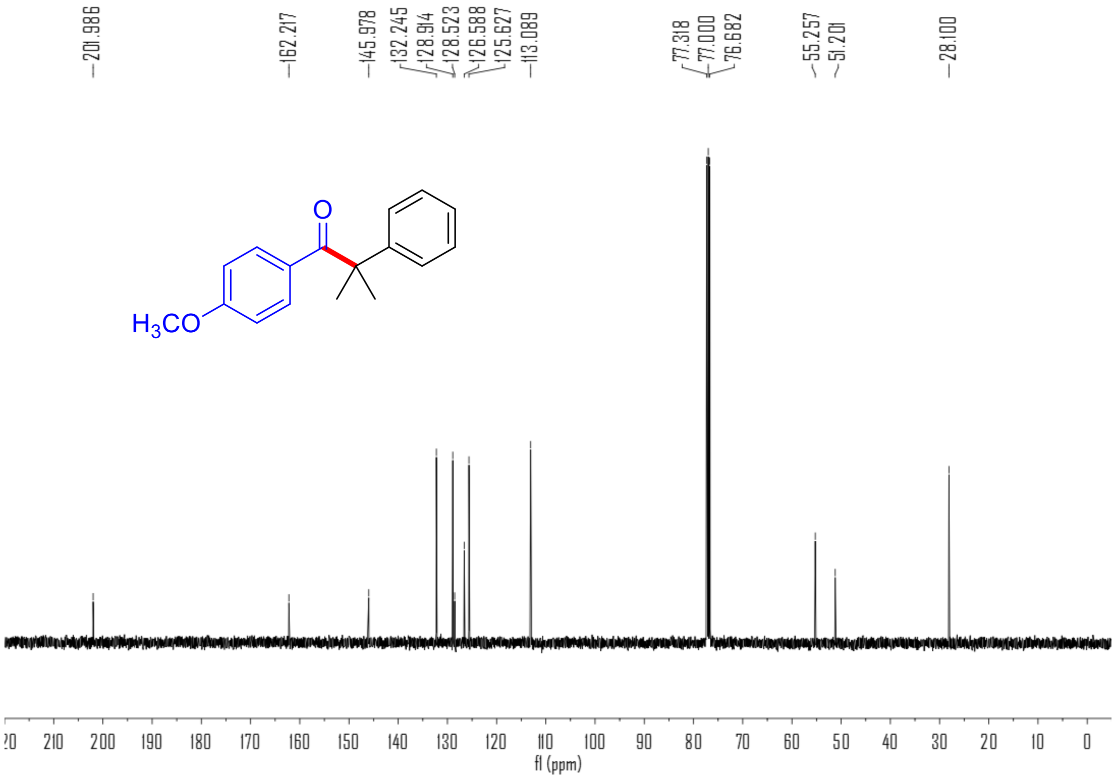

Supplementary Figure 87. **^{13}C NMR** spectrum of **3ai**

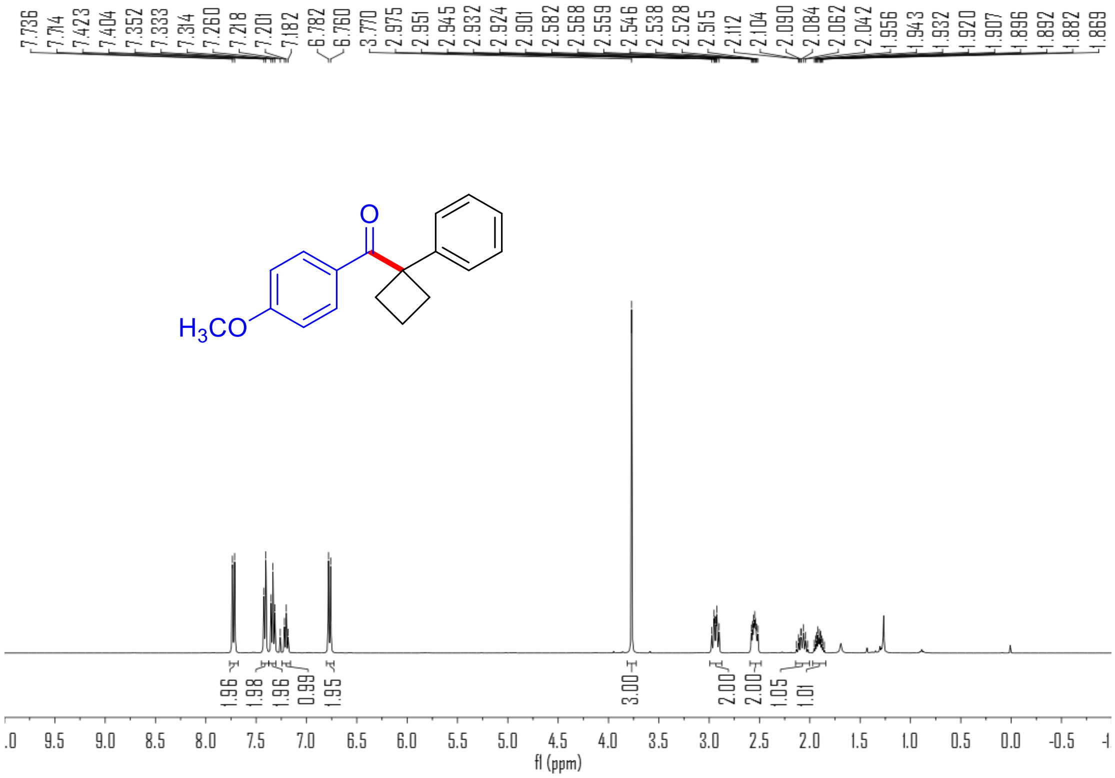

Supplementary Figure 88. **^{1}H NMR** spectrum of **3ag**

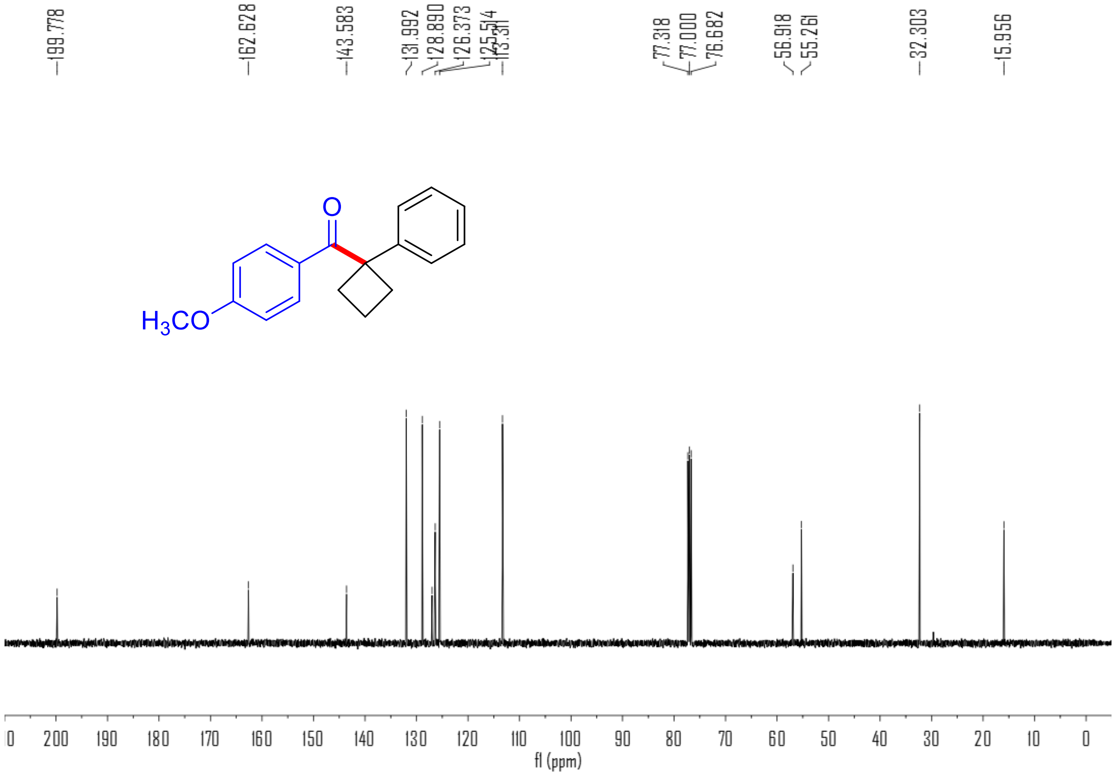

Supplementary Figure 89. **^{13}C NMR** spectrum of **3ag**

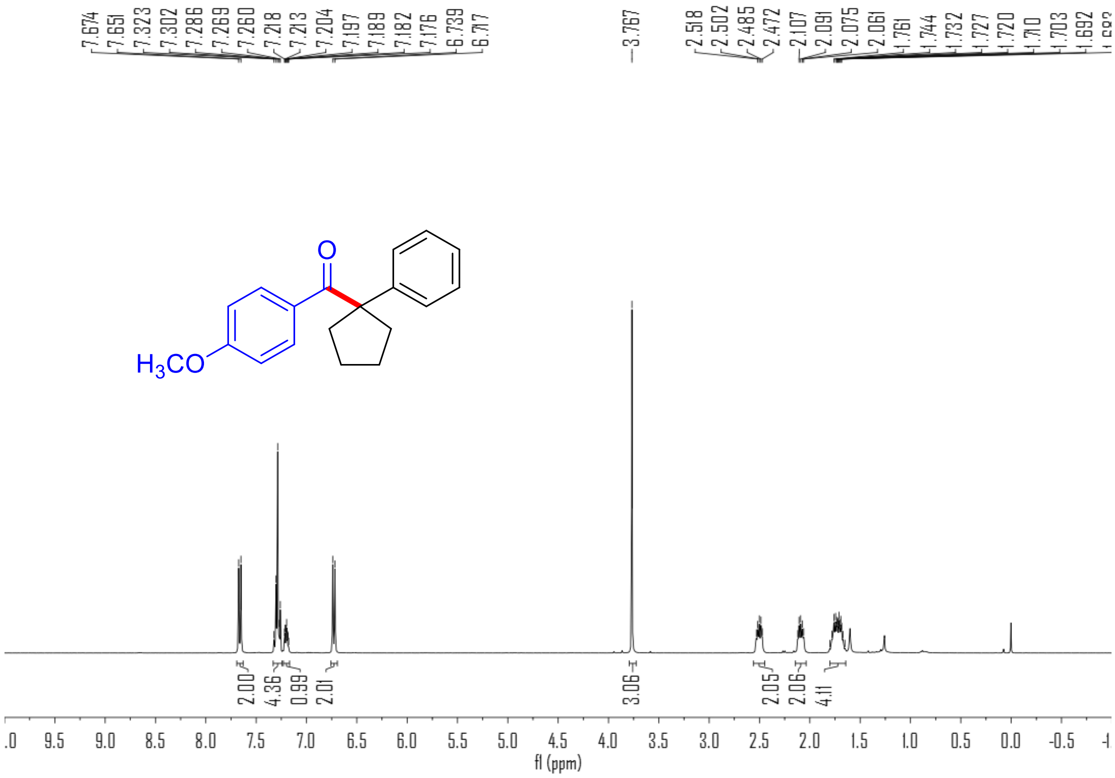

Supplementary Figure 90. **^{1}H NMR** spectrum of **3ak**

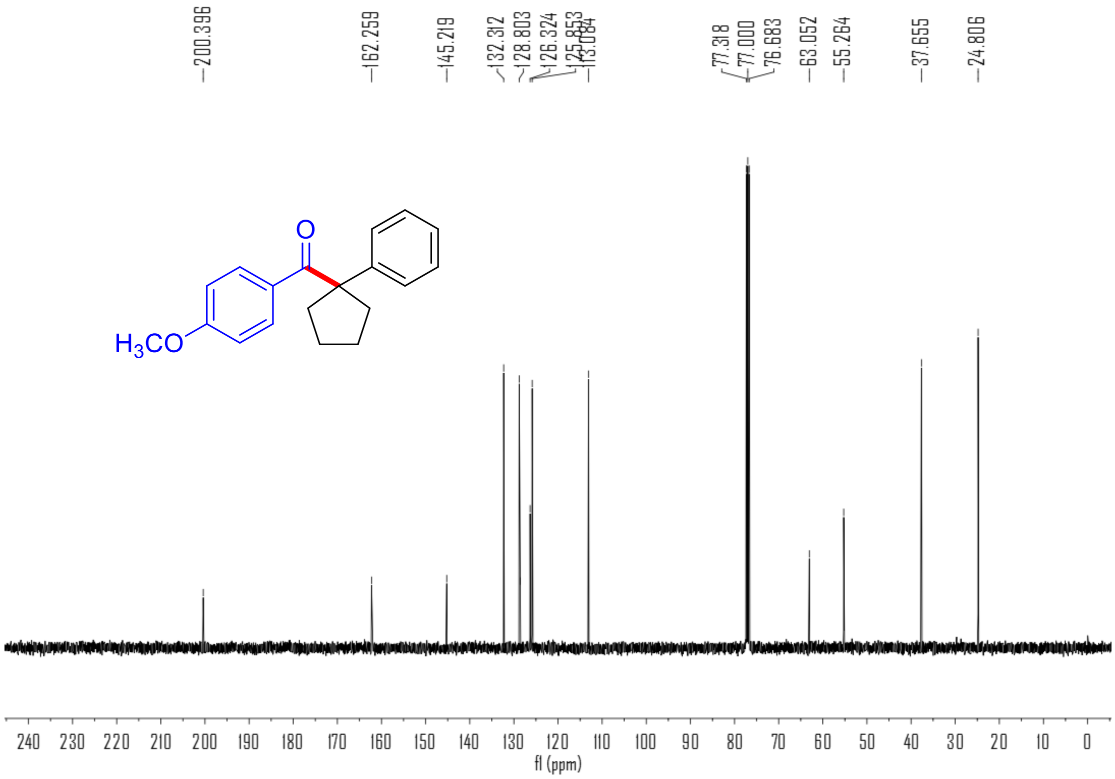

Supplementary Figure 91. **^{13}C NMR** spectrum of **3ak**

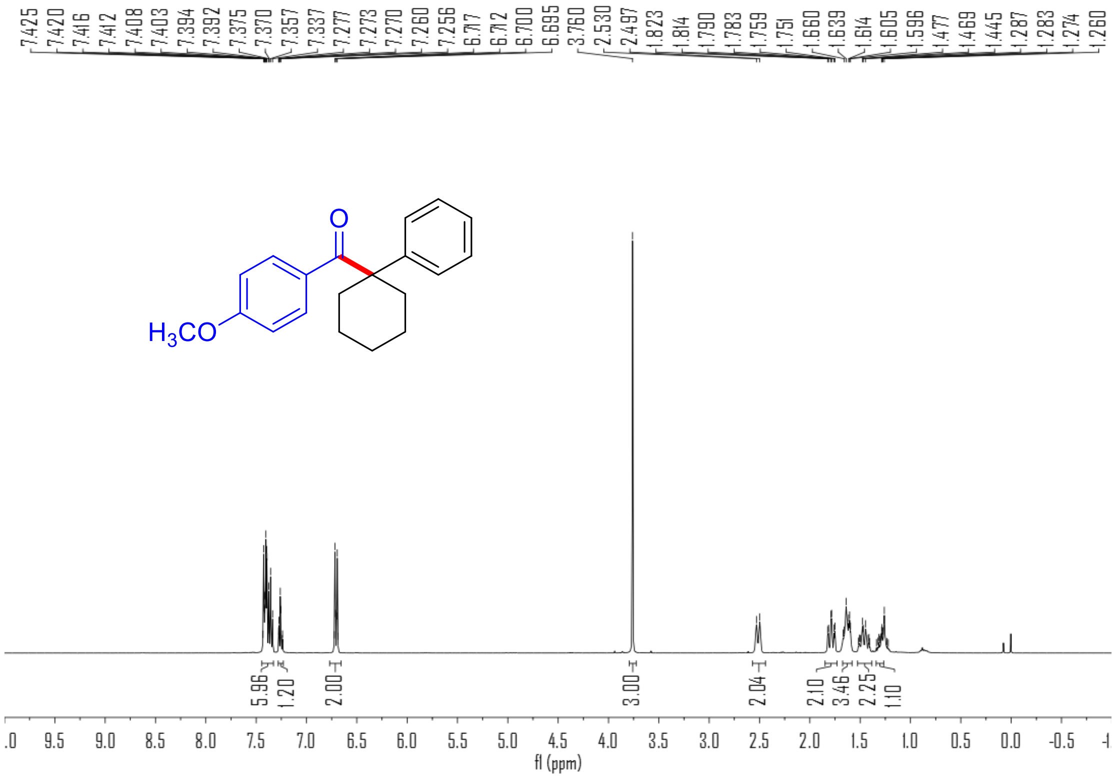

Supplementary Figure 92. ^{1}**H NMR** spectrum of **3al**

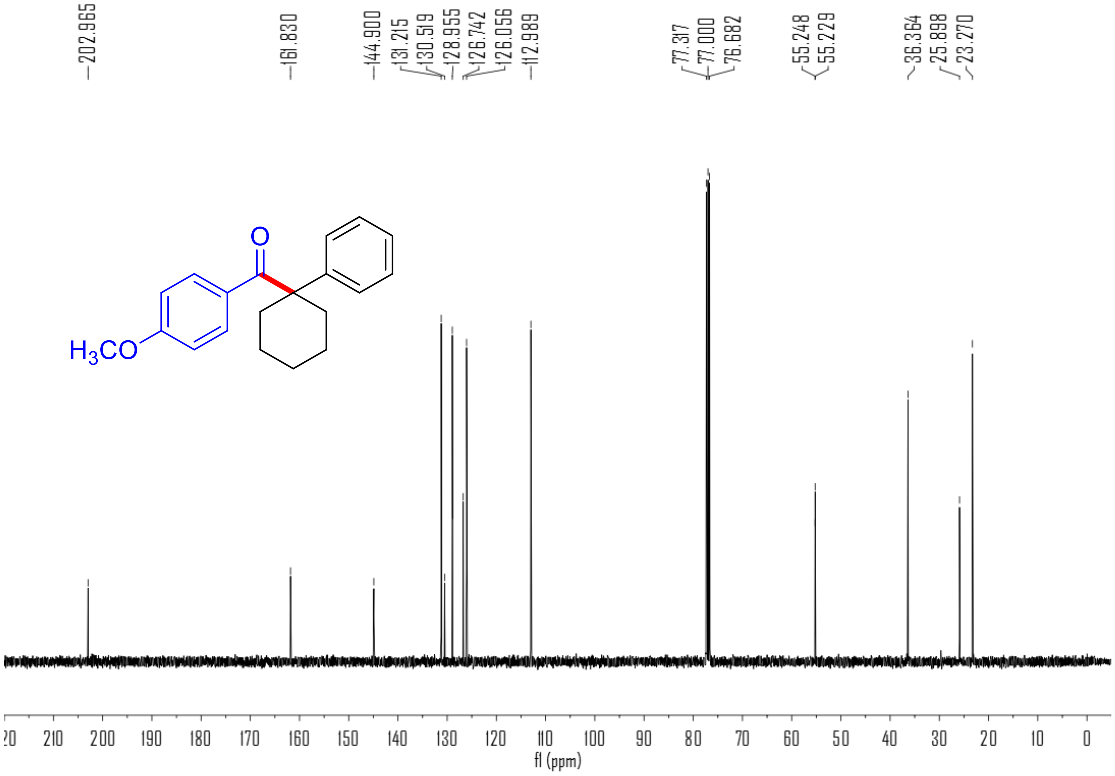

Supplementary Figure 93. ^{13}**C NMR** spectrum of **3al**

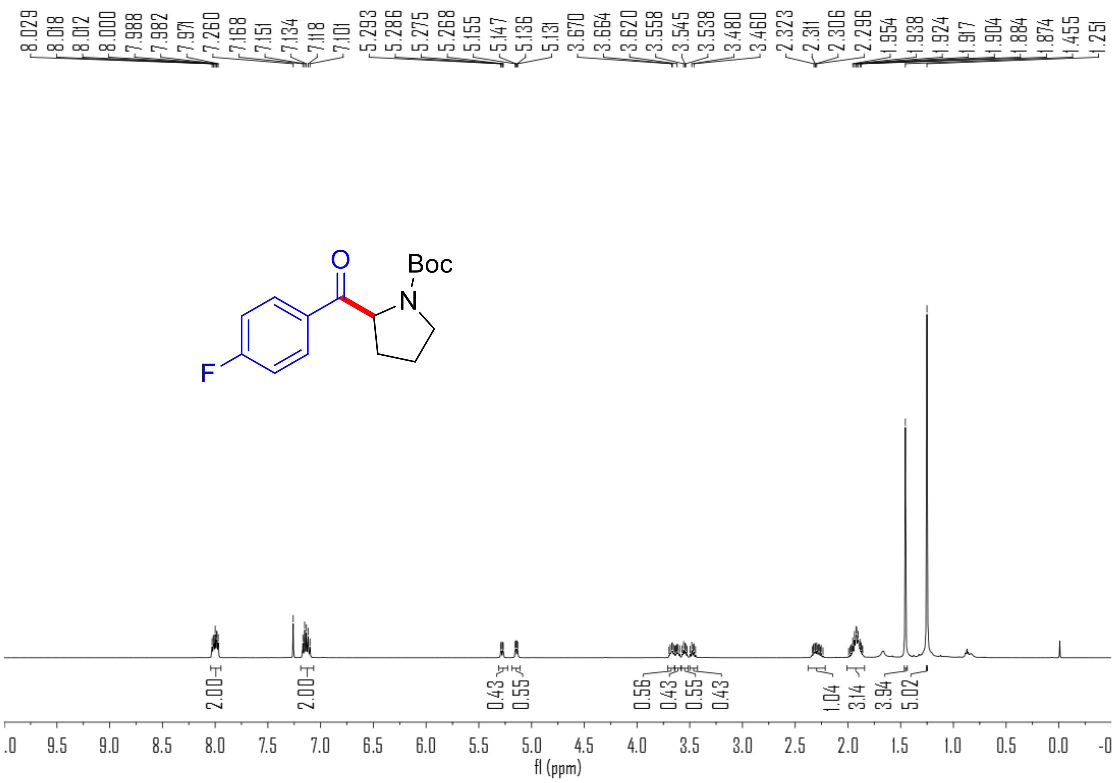

Supplementary Figure 94. ^{1}**H NMR** spectrum of **3an**

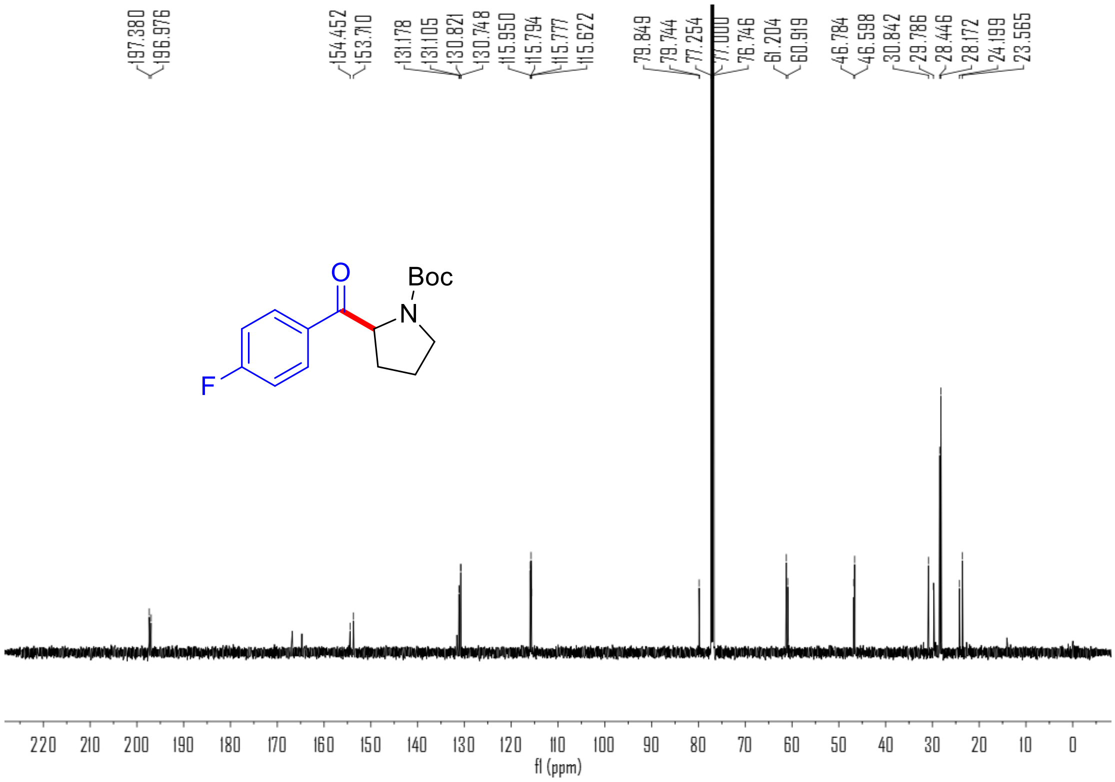

Supplementary Figure 95. ^{13}**C NMR** spectrum of **3an**

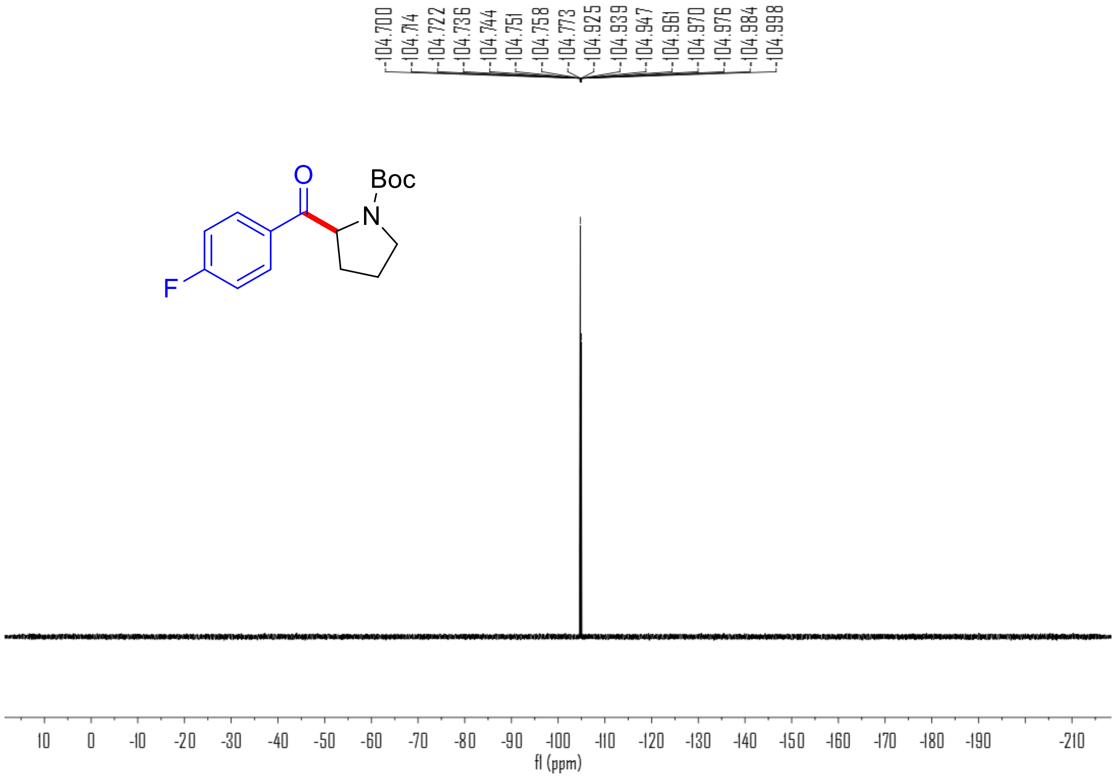

Supplementary Figure 96. ^{19}F NMR spectrum of **3an**

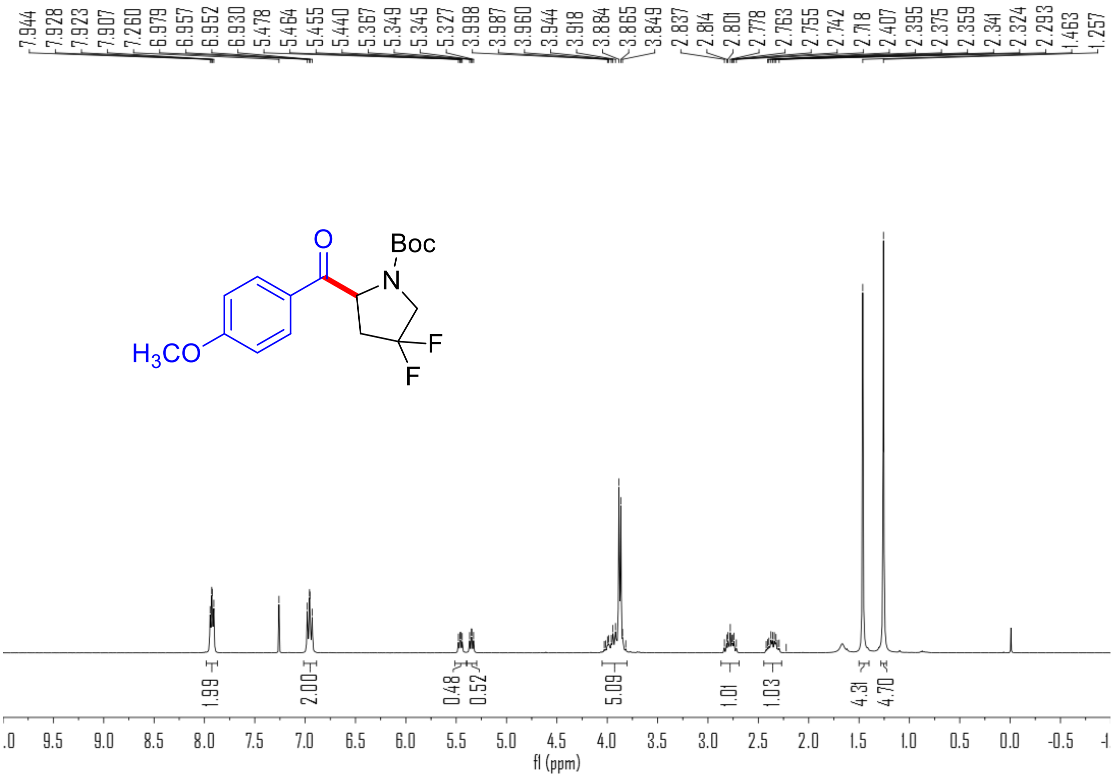

Supplementary Figure 97. ^{1}H NMR spectrum of **3ao**

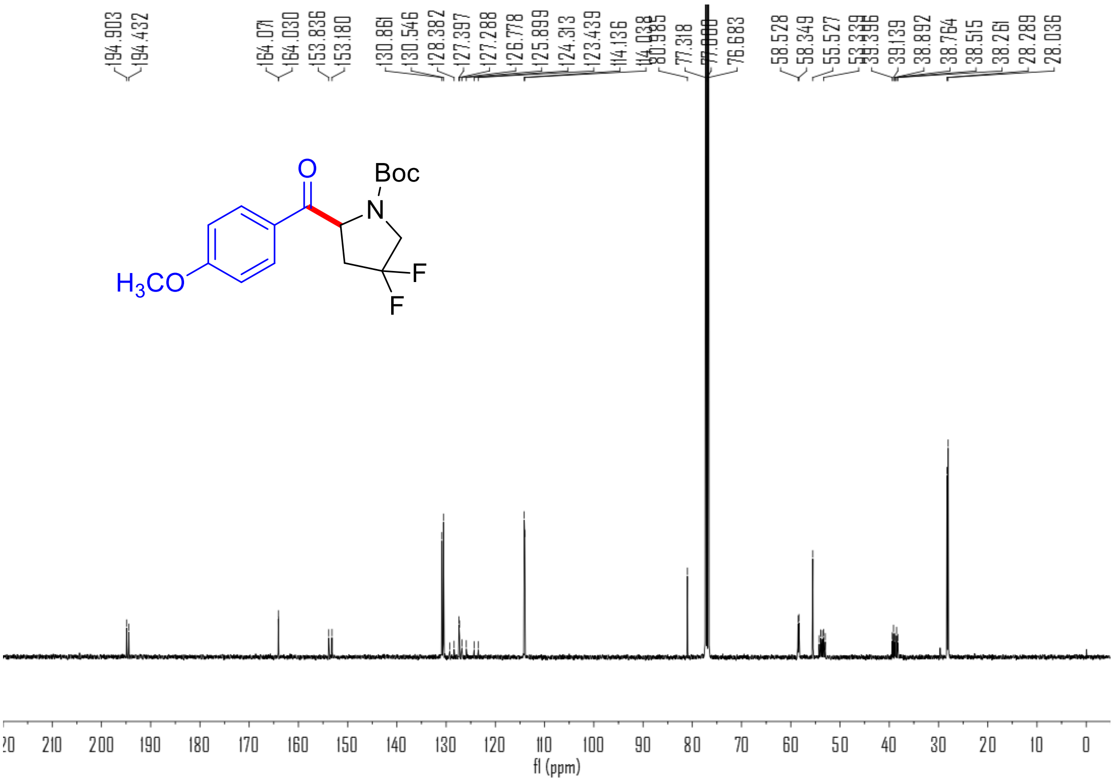

Supplementary Figure 98. ^{13}C NMR spectrum of **3ao**

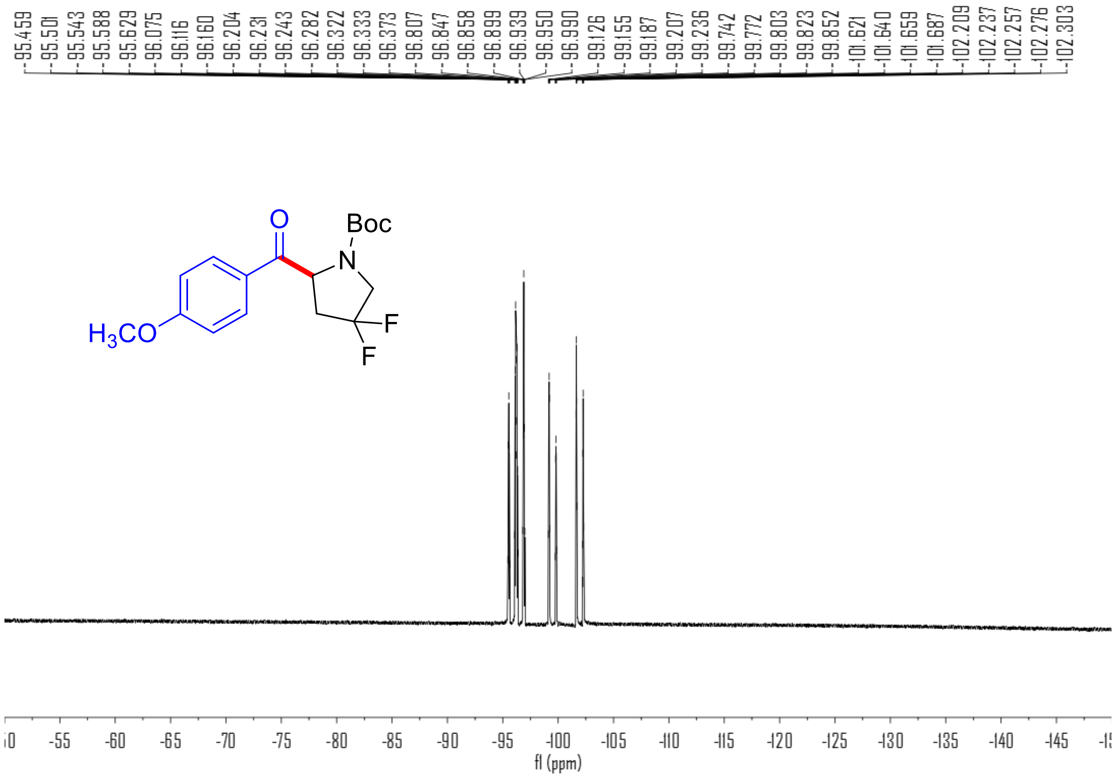

Supplementary Figure 99. ^{19}F NMR spectrum of **3ao**

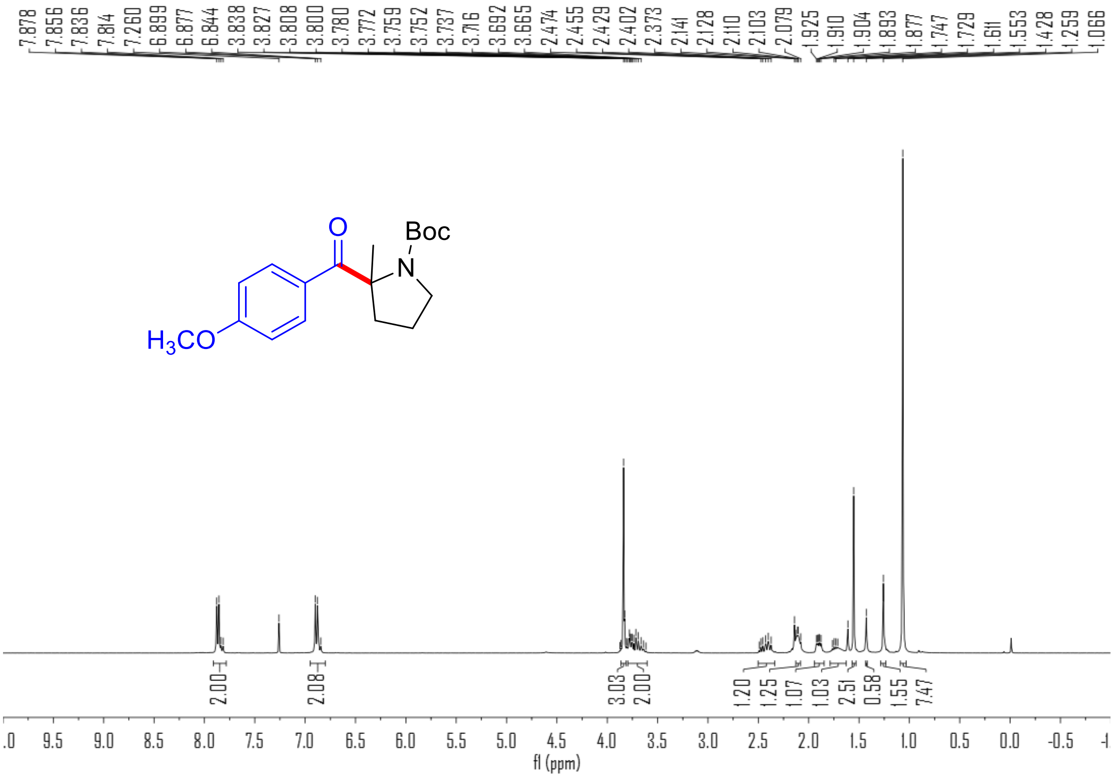

Supplementary Figure 100. ^{1}**H NMR** spectrum of **3ap**

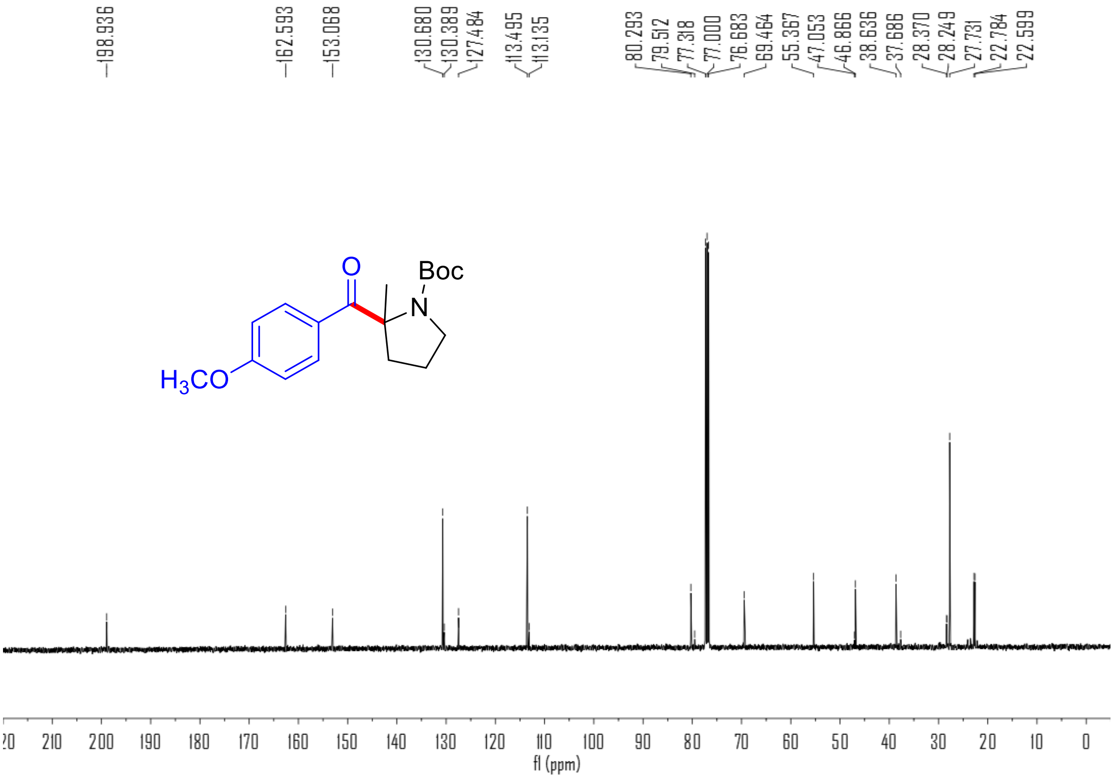

Supplementary Figure 101. ^{13}**C NMR** spectrum of **3ap**

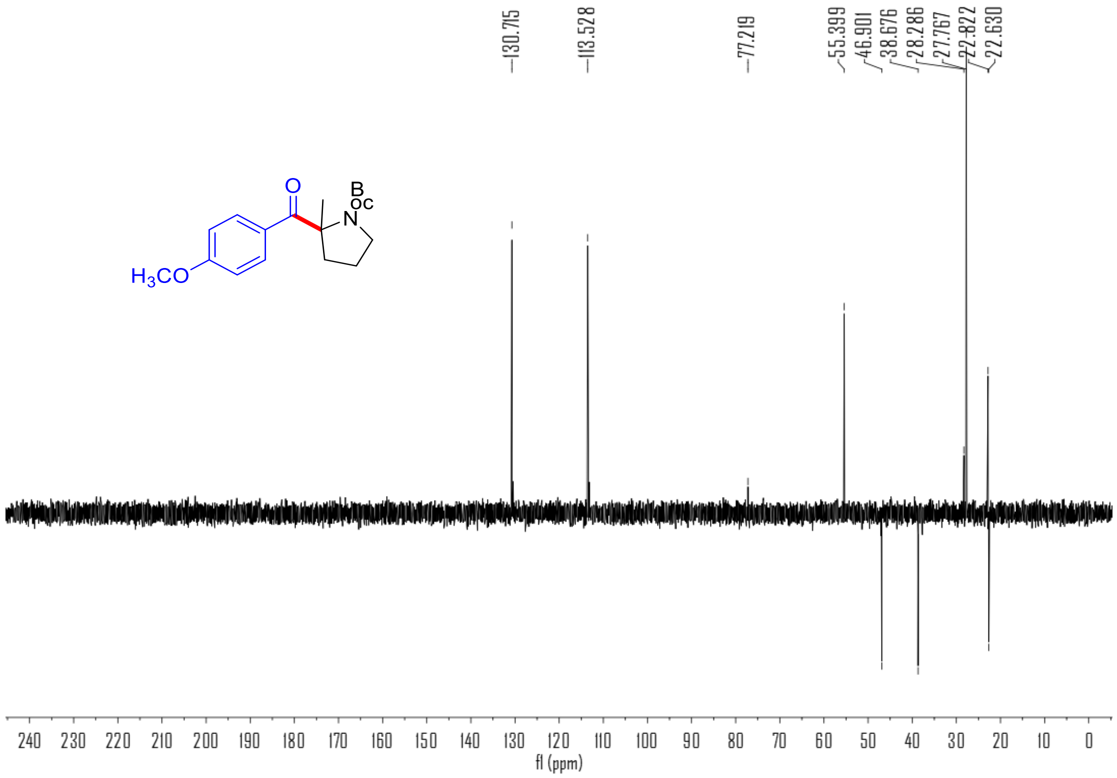

Supplementary Figure 102. DEPT135 spectrum of **3ap**

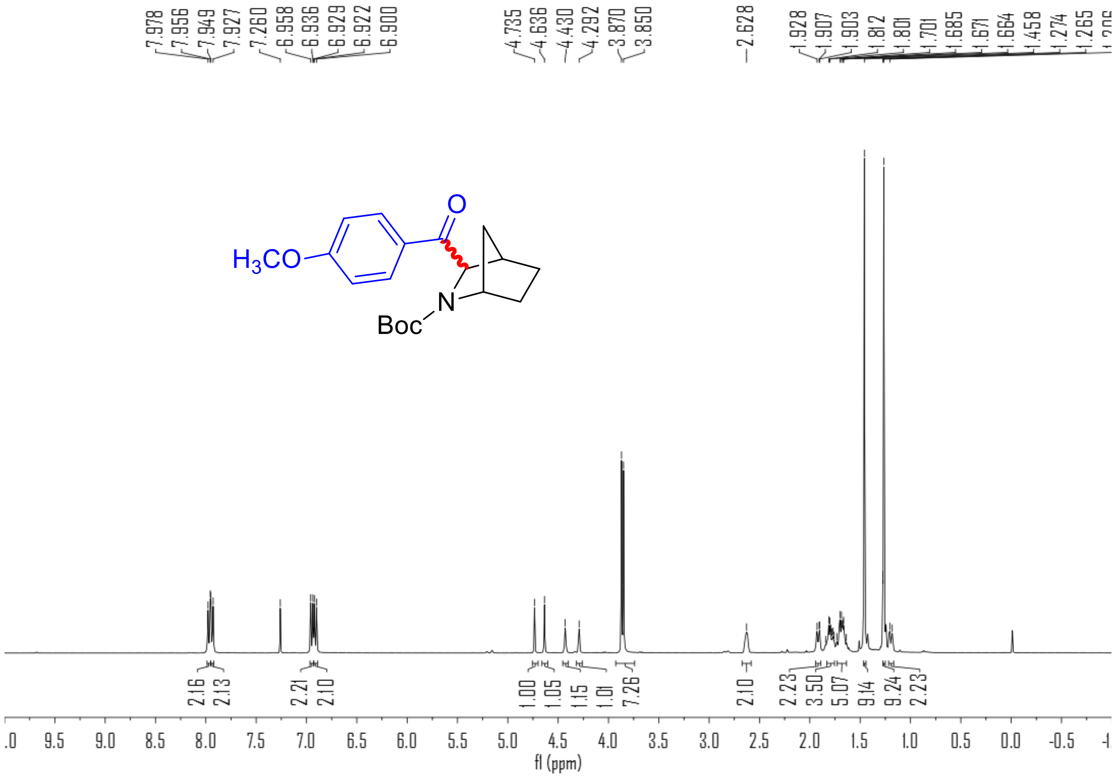

Supplementary Figure 103. ^{1}**H NMR** spectrum of **3aq**

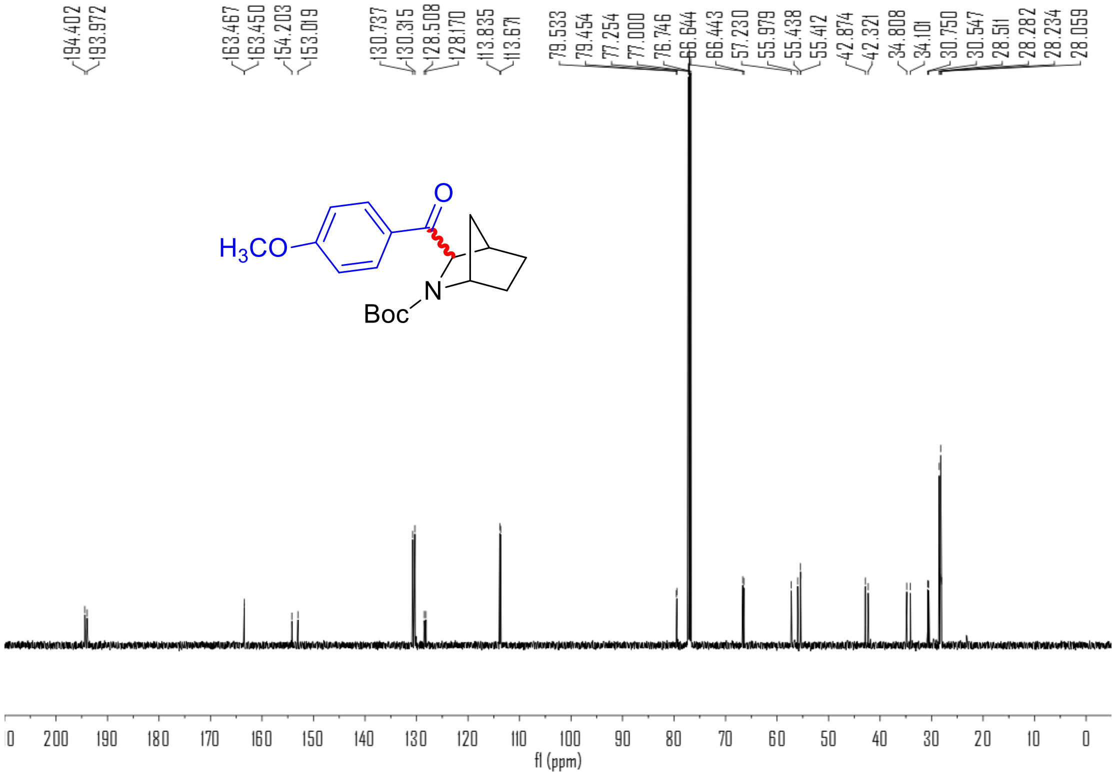

Supplementary Figure 104. ^{13}**C NMR** spectrum of **3aq**

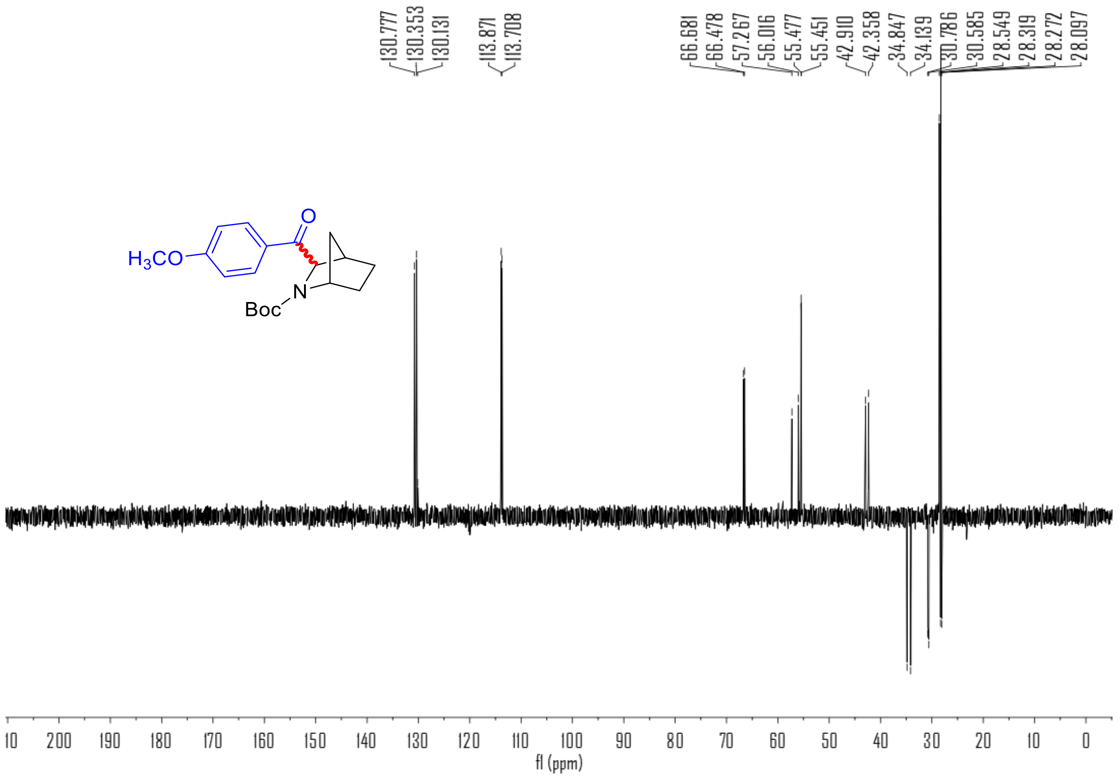

Supplementary Figure 105. DEPT135 spectrum of **3aq**

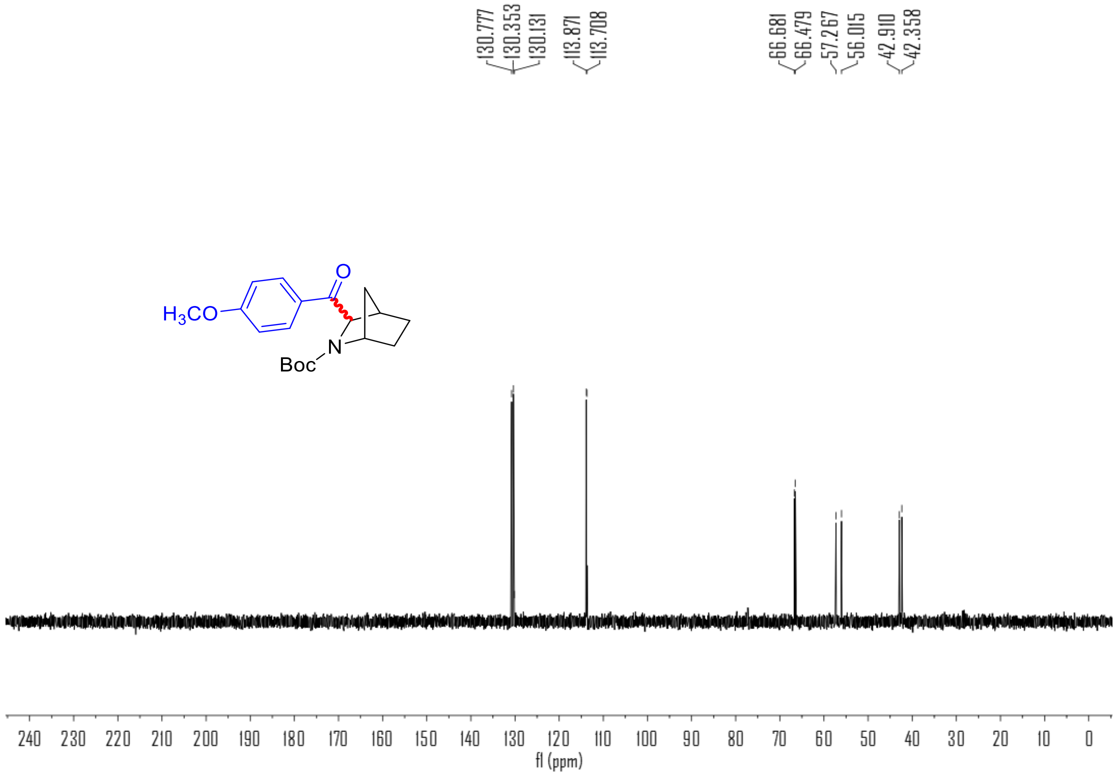

Supplementary Figure 106. DEPT90 spectrum of **3aq**

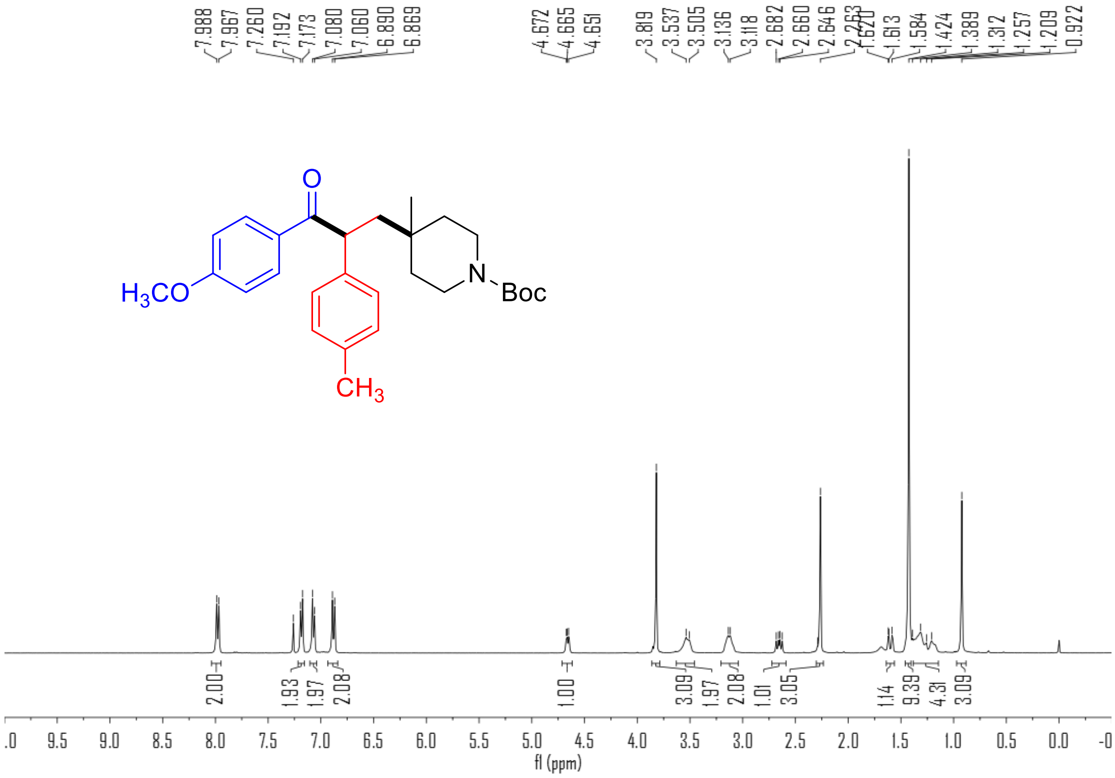

Supplementary Figure 107. ^{1}**H NMR** spectrum of **5a**

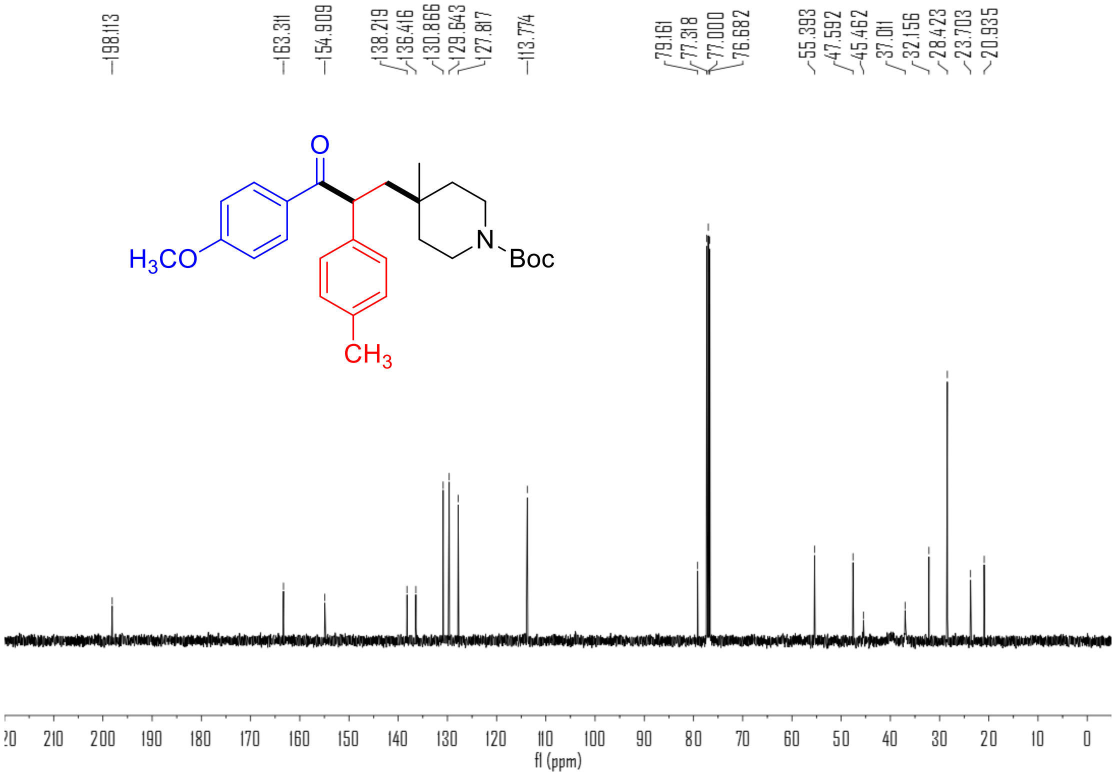

Supplementary Figure 108. ^{13}**C NMR** spectrum of **5a**

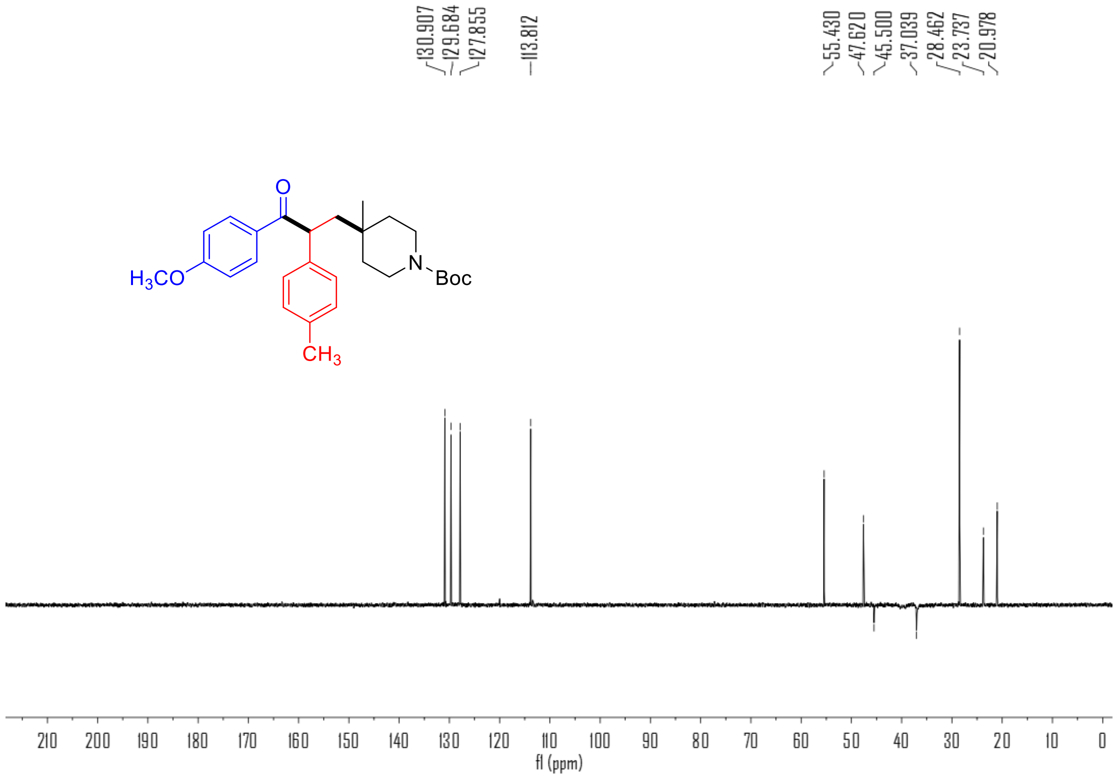

Supplementary Figure 109. DEPT135 spectrum of **5a**

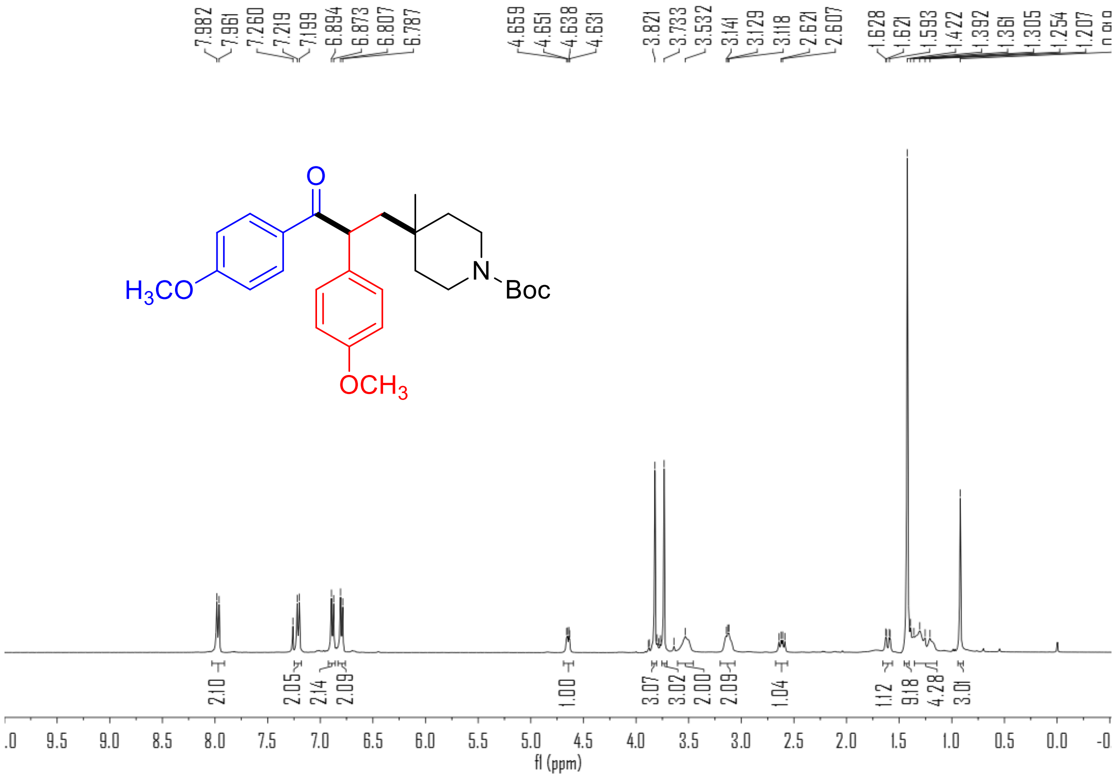

Supplementary Figure 110. ^{1}**H NMR** spectrum of **5b**

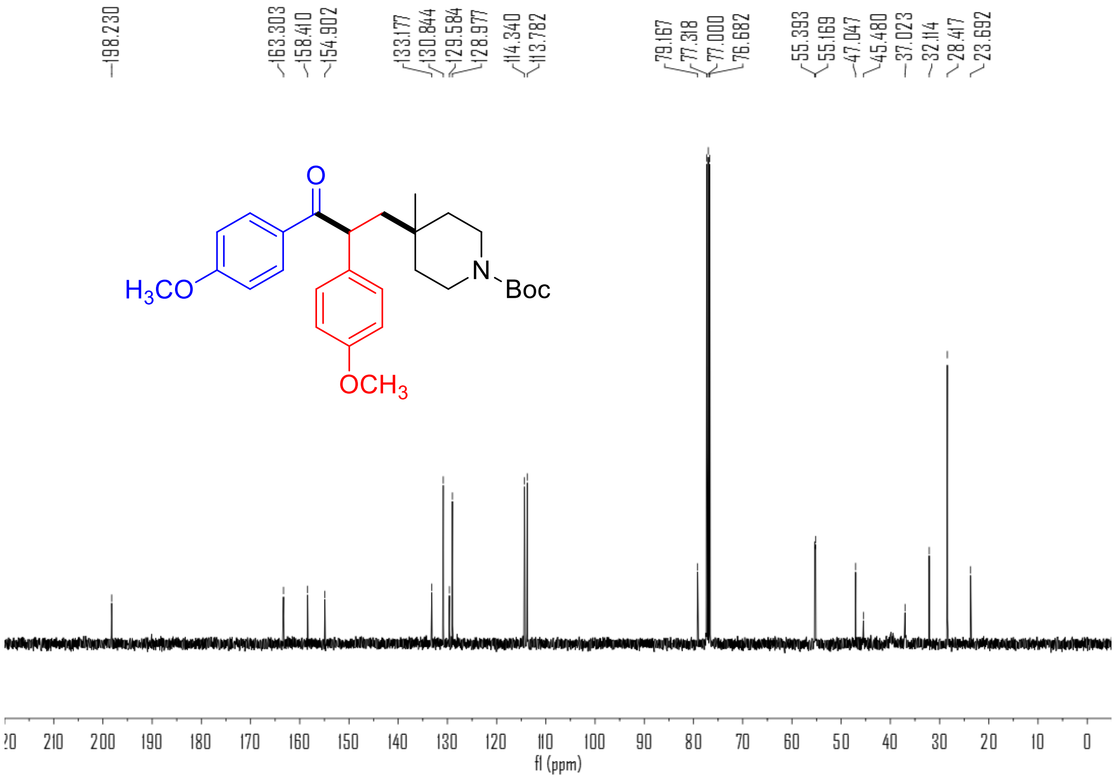

Supplementary Figure 111. ^{13}**C NMR** spectrum of **5b**

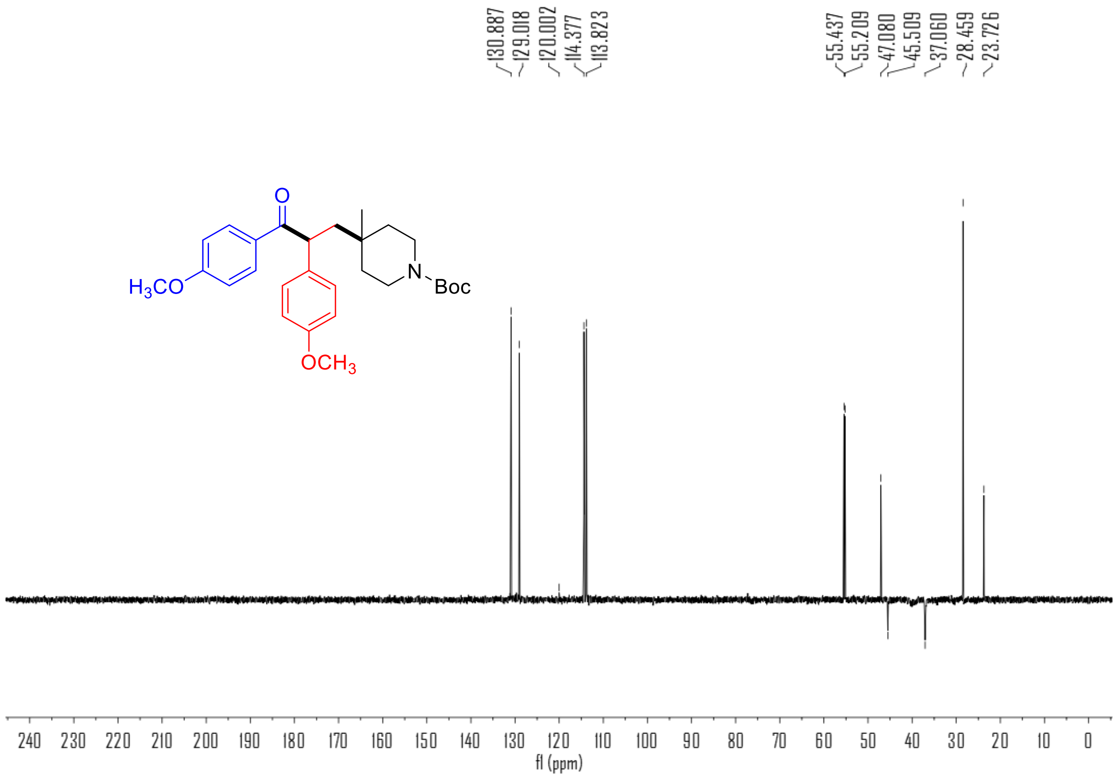

Supplementary Figure 112. DEPT135 spectrum of **5b**

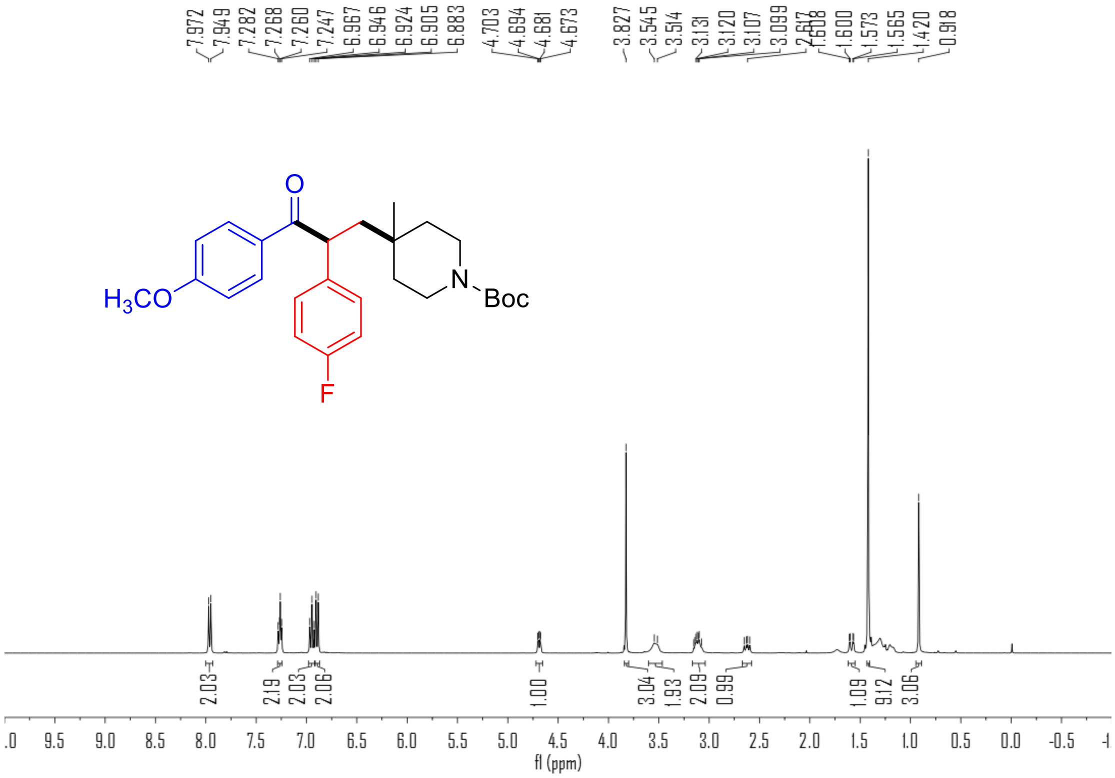

Supplementary Figure 113. ^{1}**H NMR** spectrum of **5c**

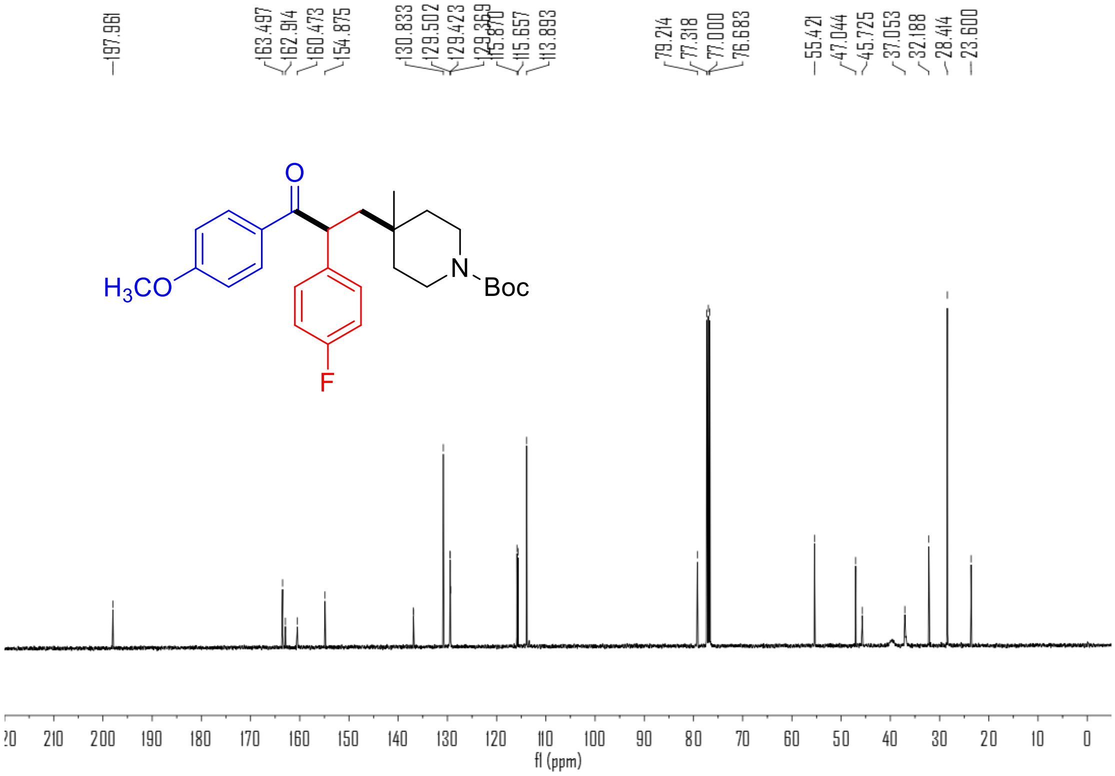

Supplementary Figure 114. ^{13}**C NMR** spectrum of **5c**

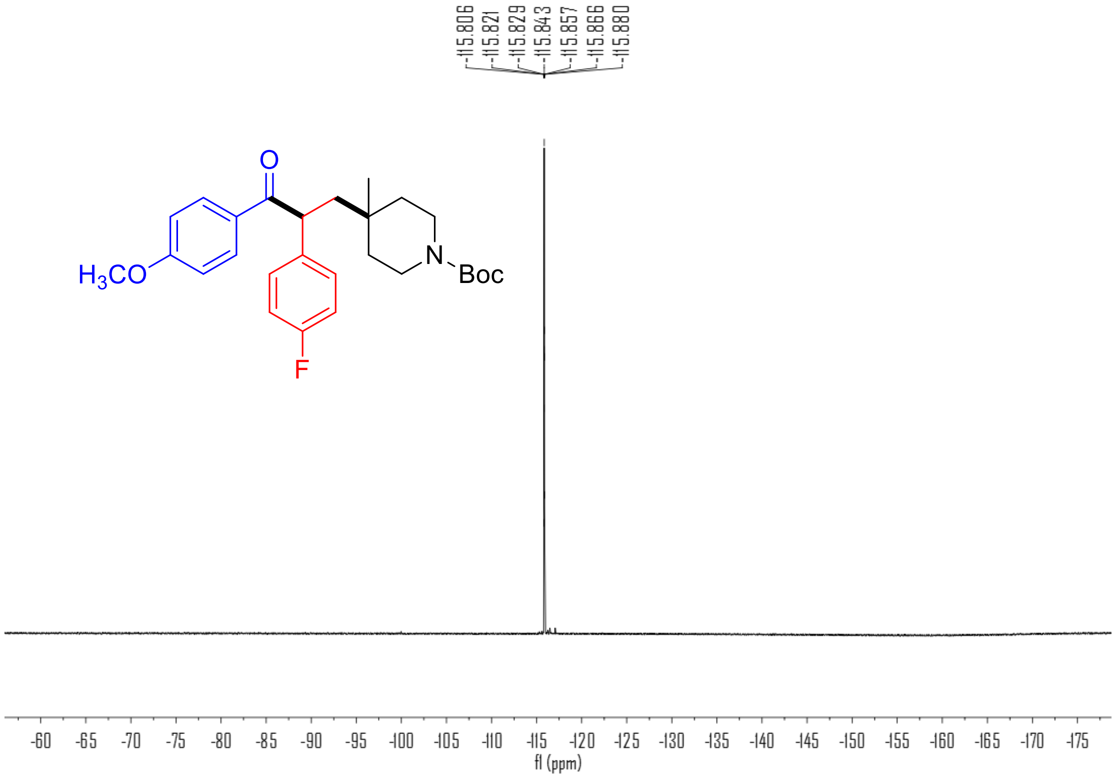

Supplementary Figure 115. **^{19}F NMR** spectrum of **5c**

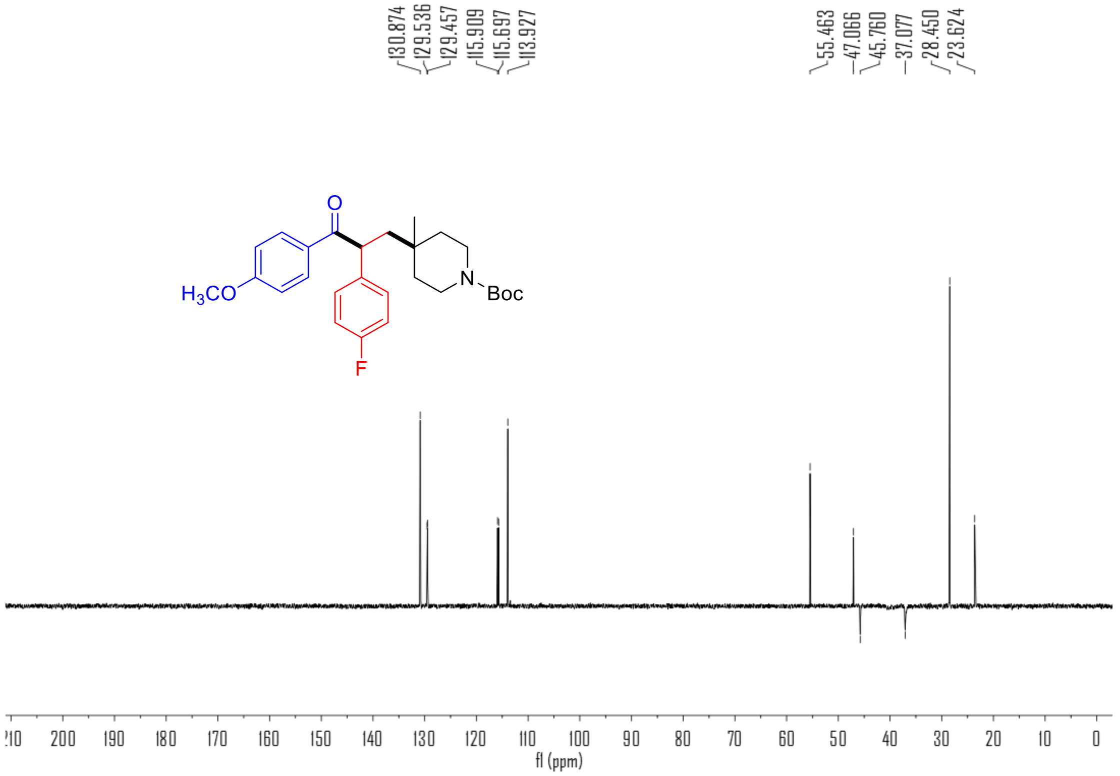

Supplementary Figure 116. DEPT135 spectrum of **5c**

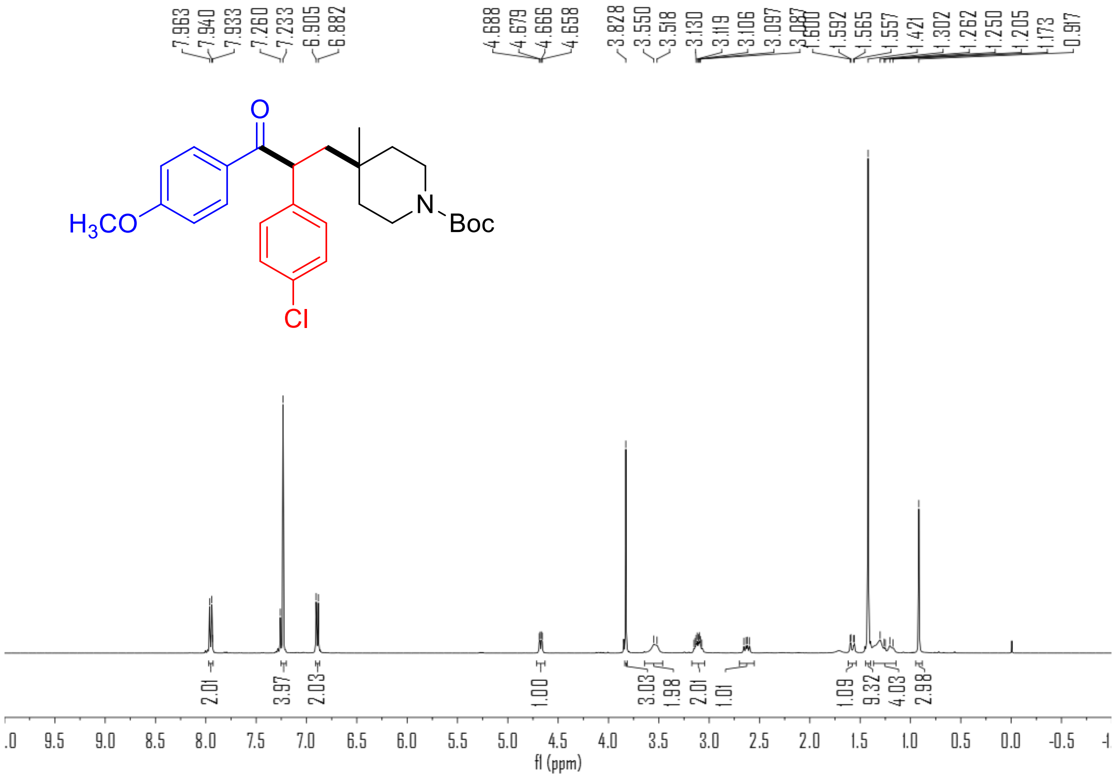

Supplementary Figure 117. ^{1}**H NMR** spectrum of **5d**

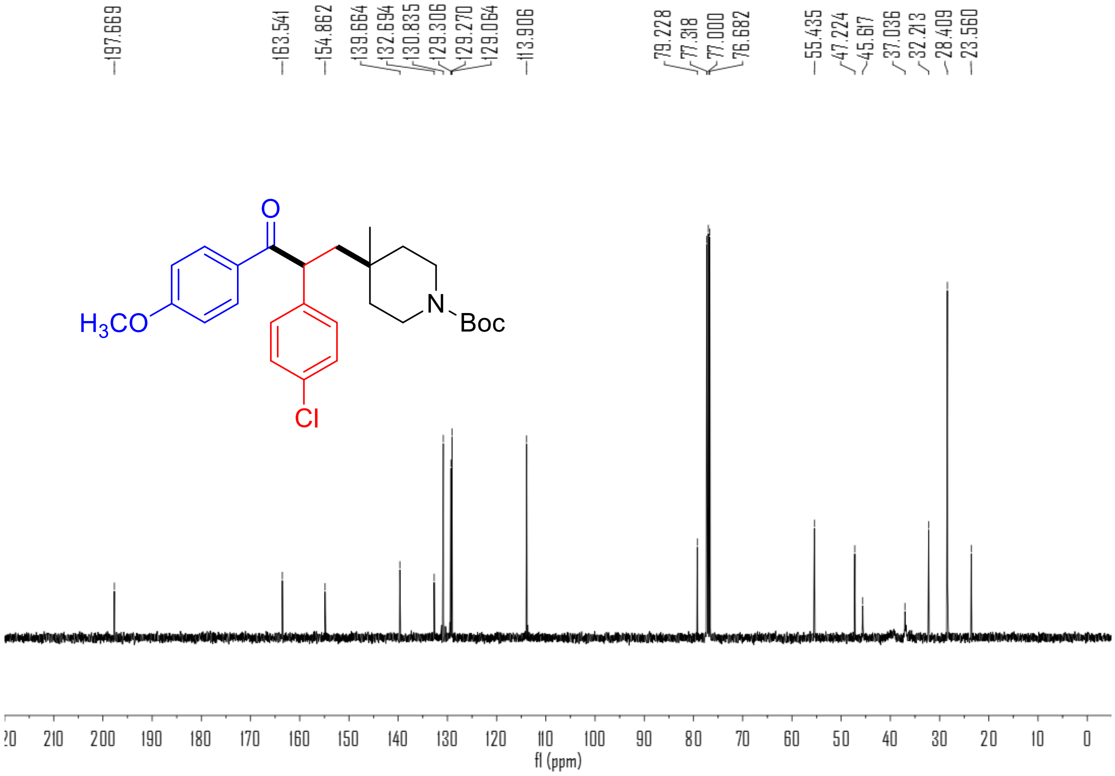

Supplementary Figure 118. ^{13}**C NMR** spectrum of **5d**

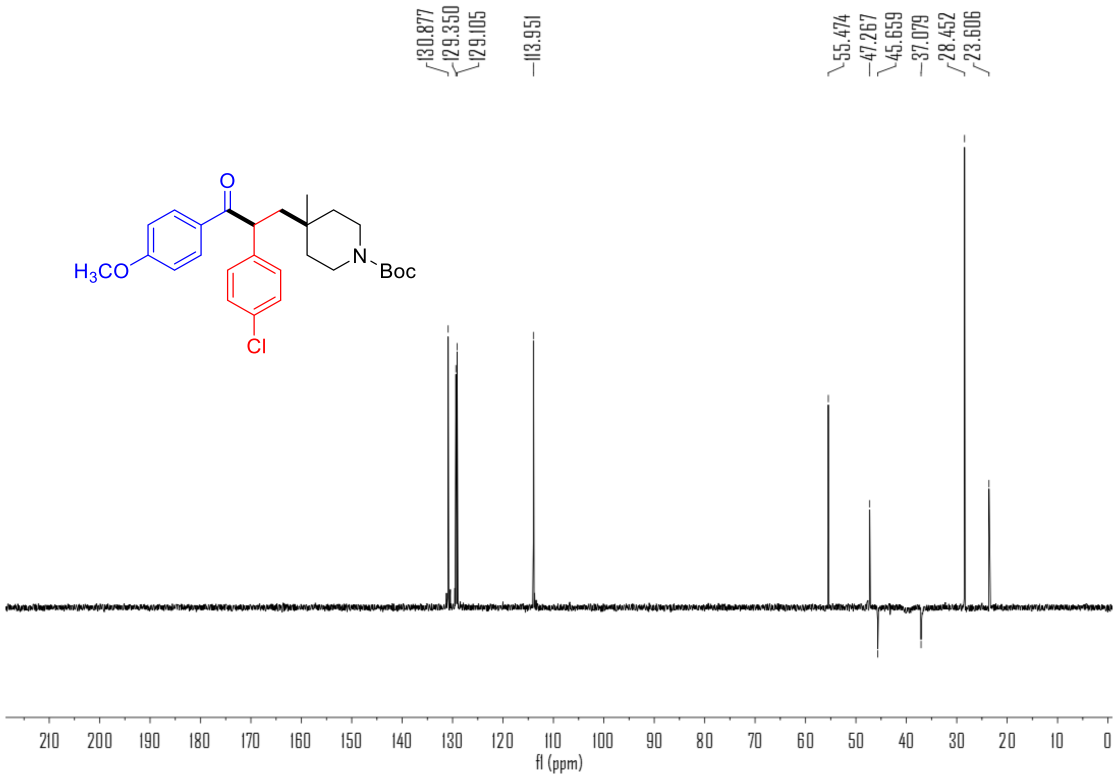

Supplementary Figure 119. DEPT135 spectrum of **5d**

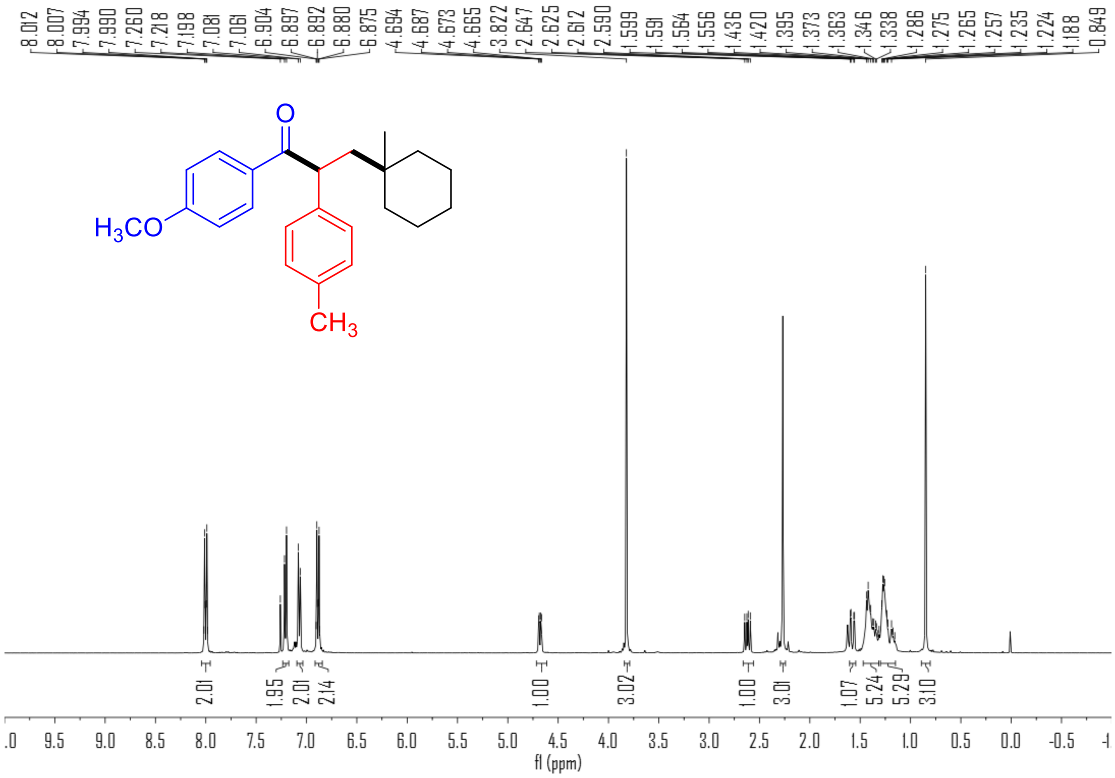

Supplementary Figure 120. ^{1}**H NMR** spectrum of **5e**

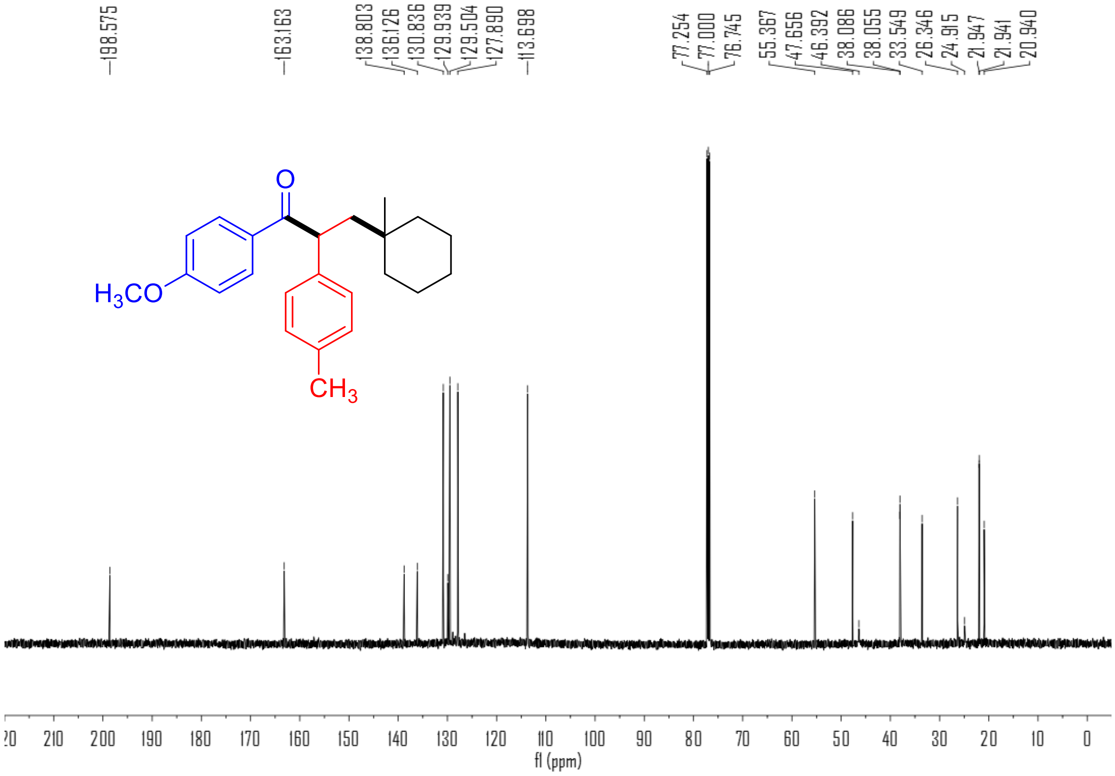

Supplementary Figure 121. ^{13}**C NMR** spectrum of **5e**

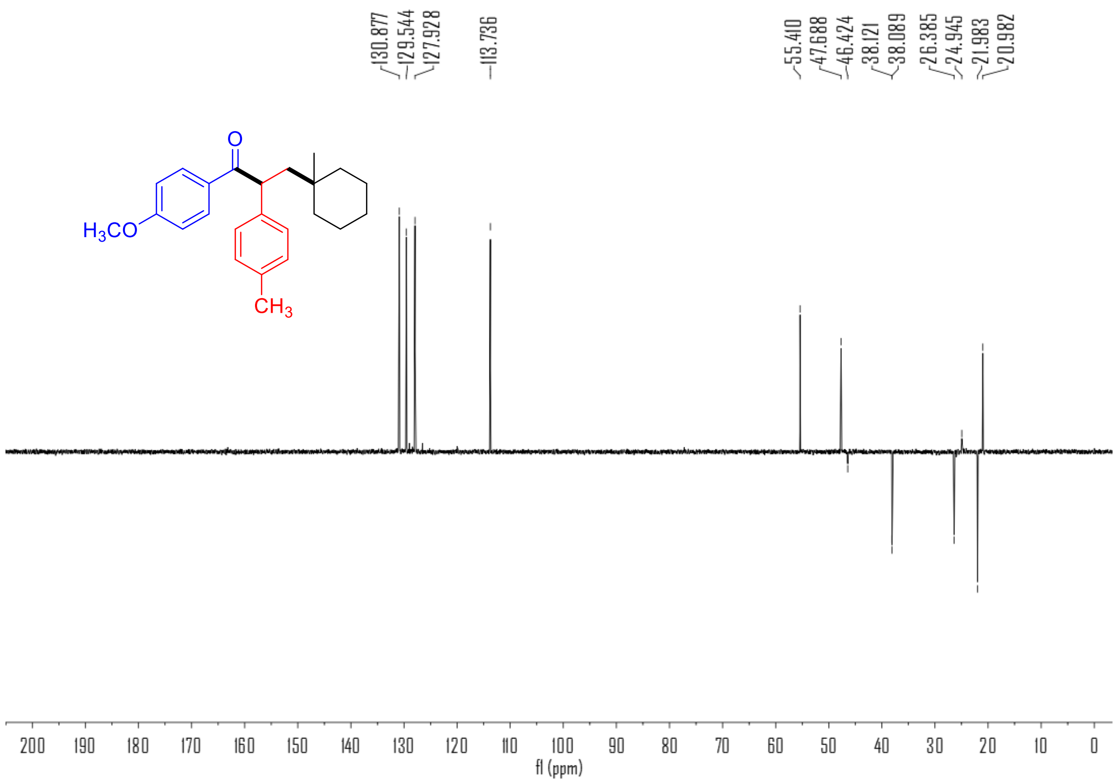

Supplementary Figure 122. DEPT135 spectrum of **5e**

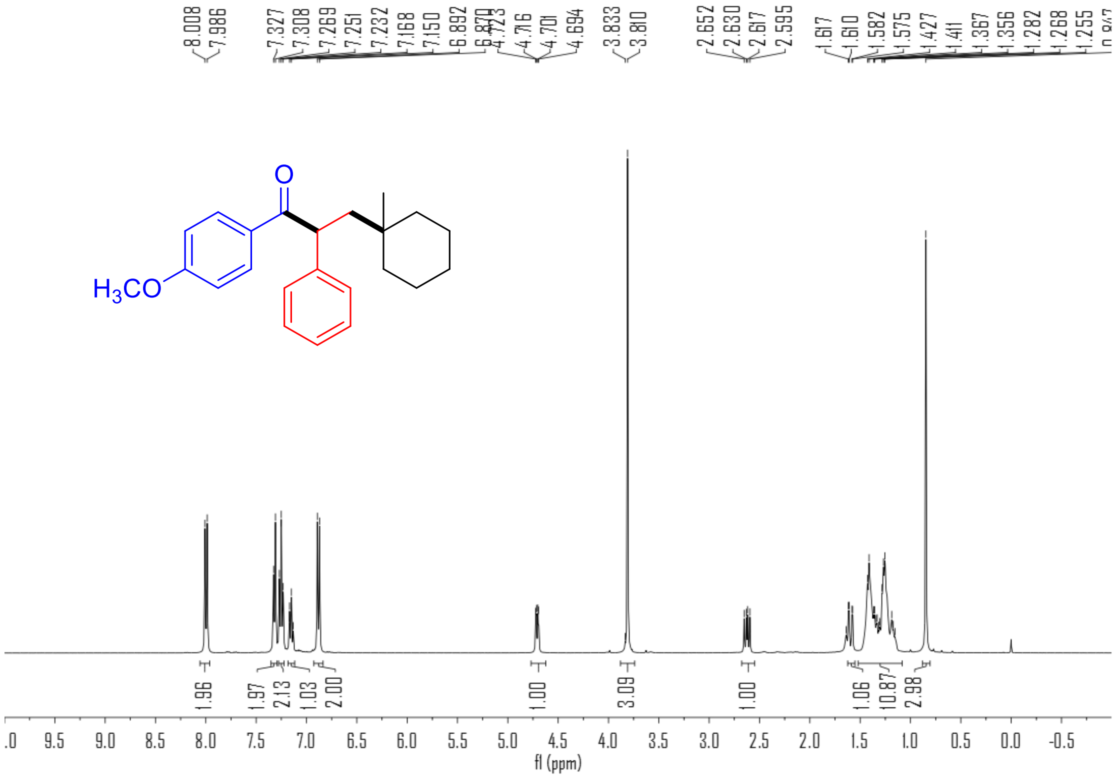

Supplementary Figure 123. **^{1}H NMR** spectrum of **5f**

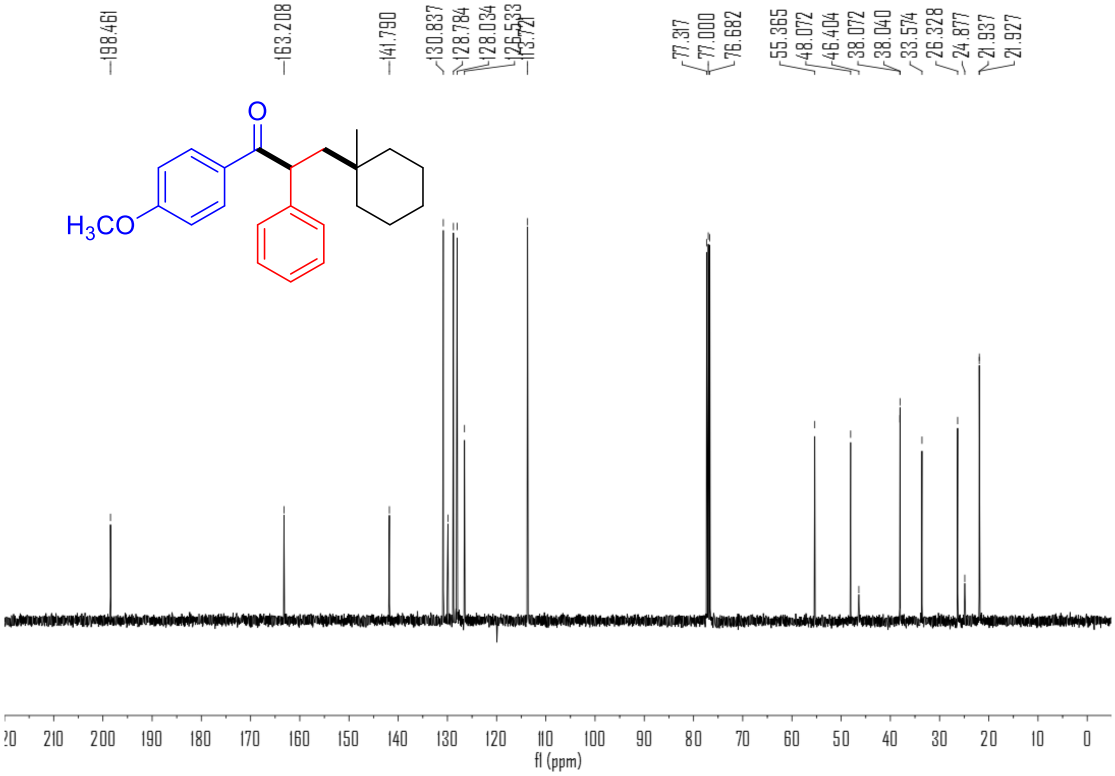

Supplementary Figure 124. **^{13}C NMR** spectrum of **5f**

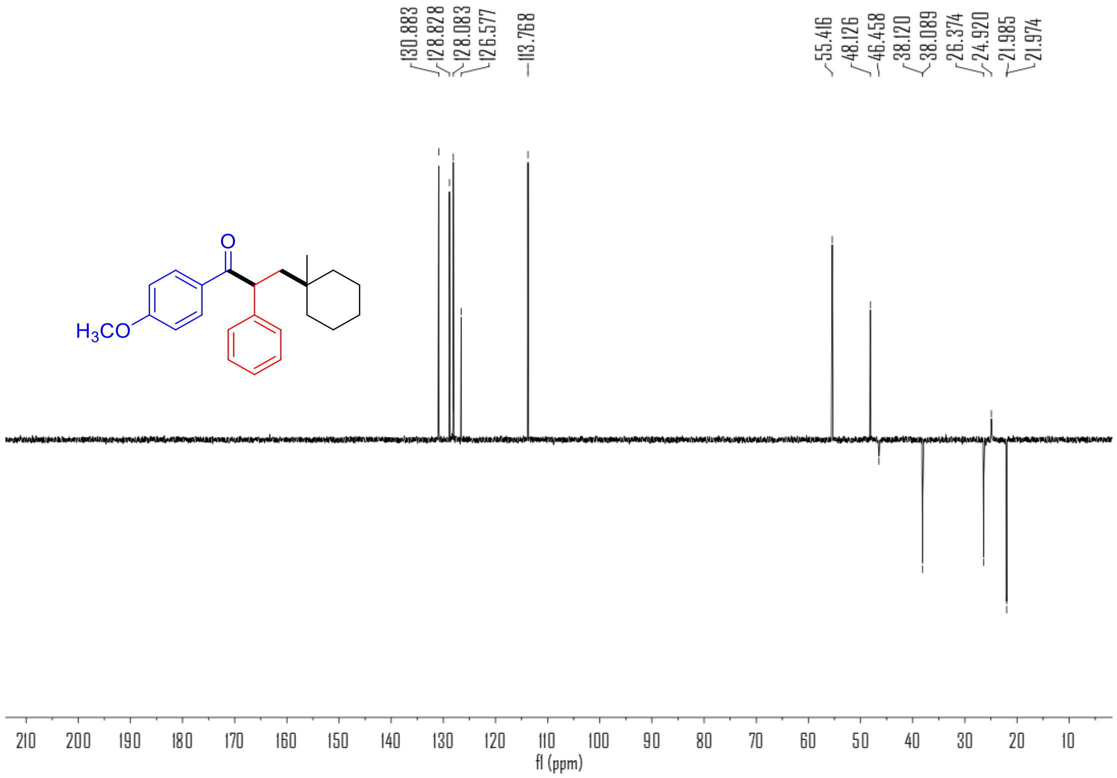

Supplementary Figure 125. DEPT135 spectrum of **5f**

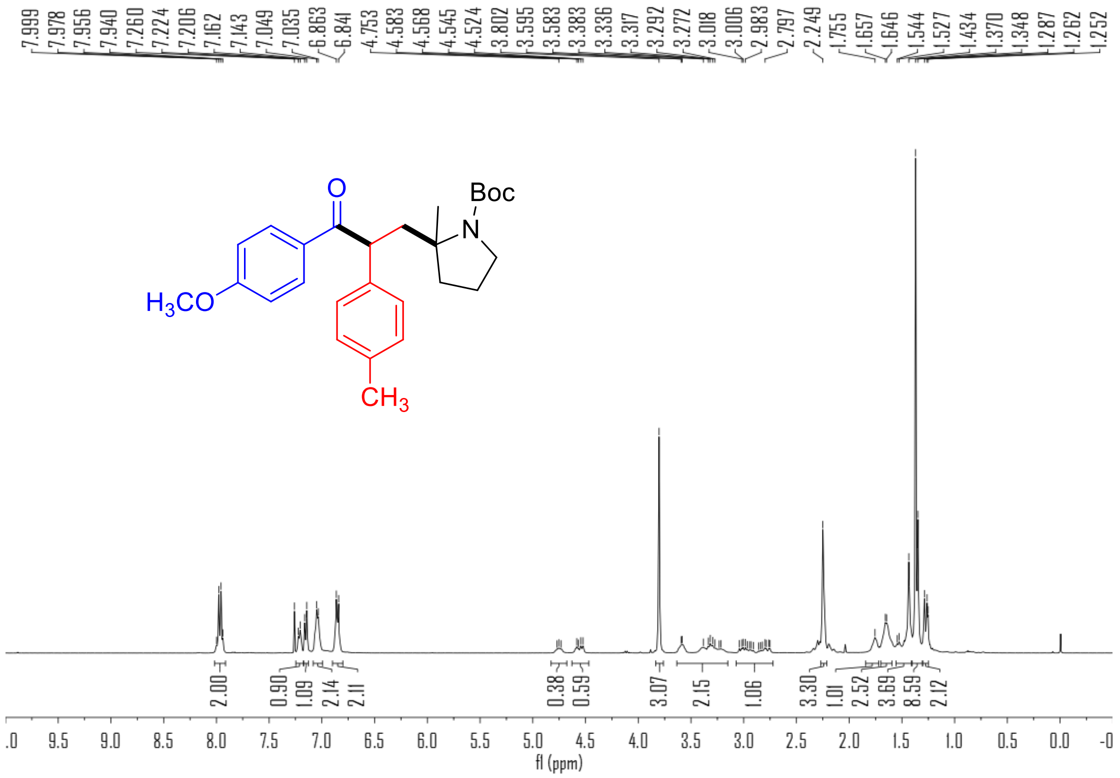

Supplementary Figure 126. ^{1}**H NMR** spectrum of **5g**

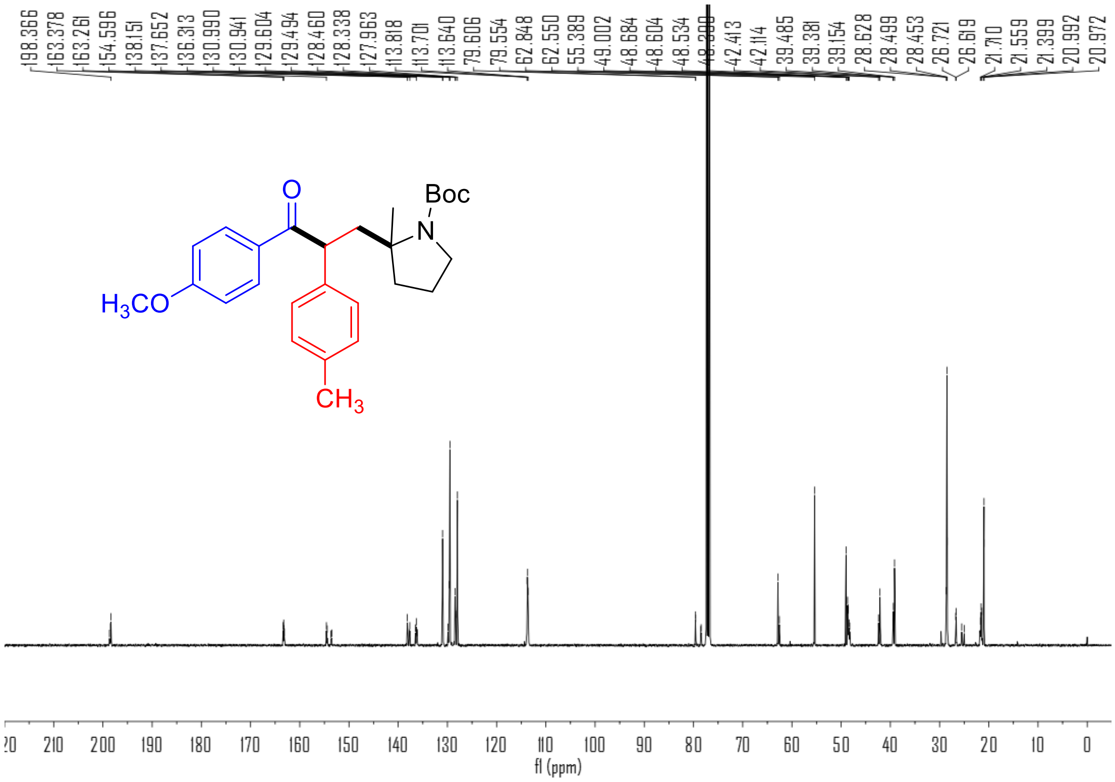

Supplementary Figure 127. ^{13}**C NMR** spectrum of **5g**

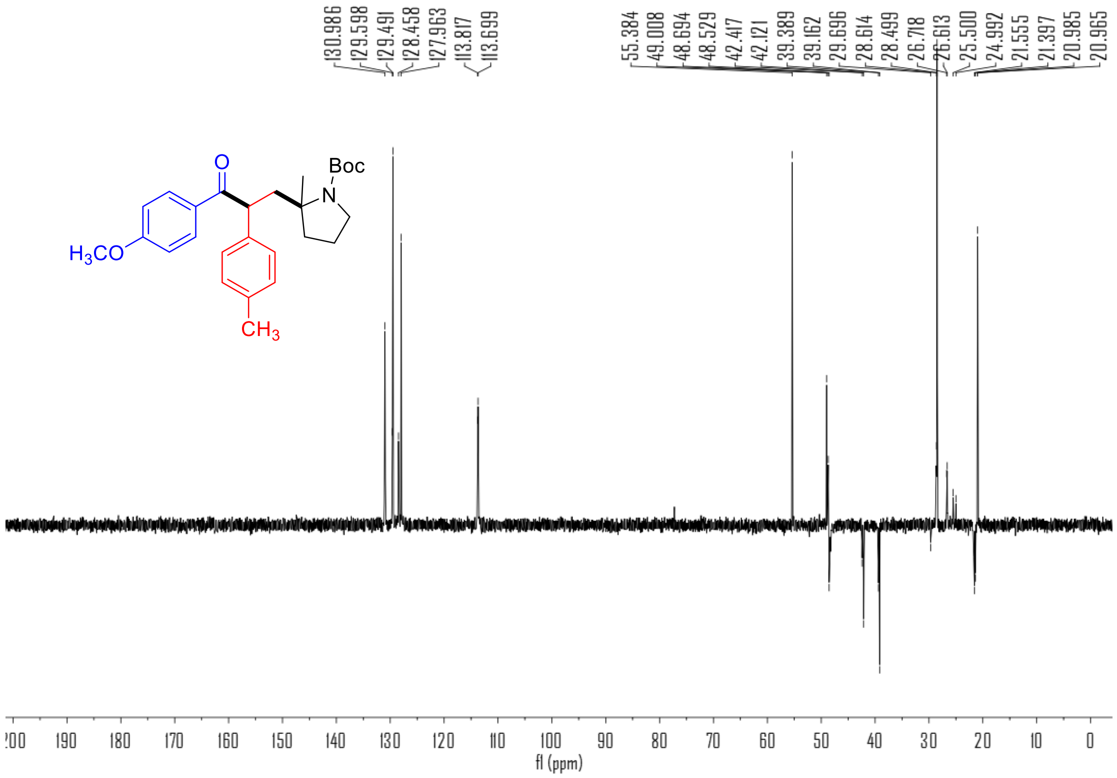

Supplementary Figure 128. DEPT135 spectrum of **5g**

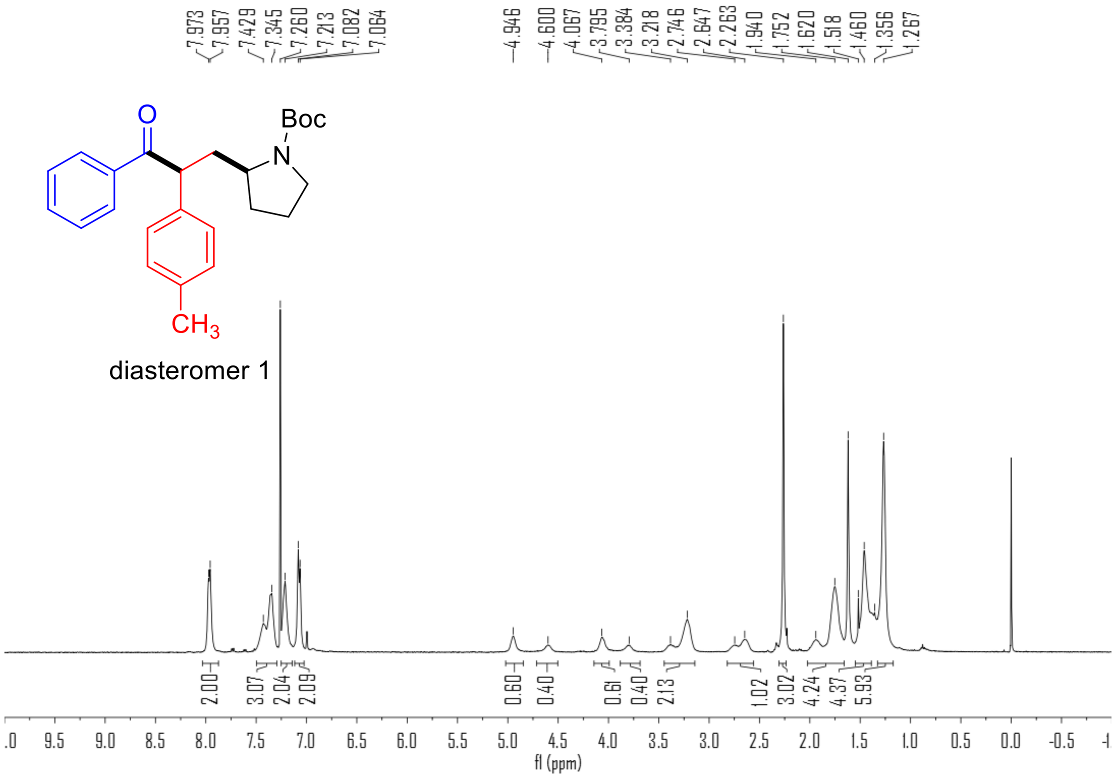

Supplementary Figure 129. ^{1}**H NMR** spectrum of **5h**

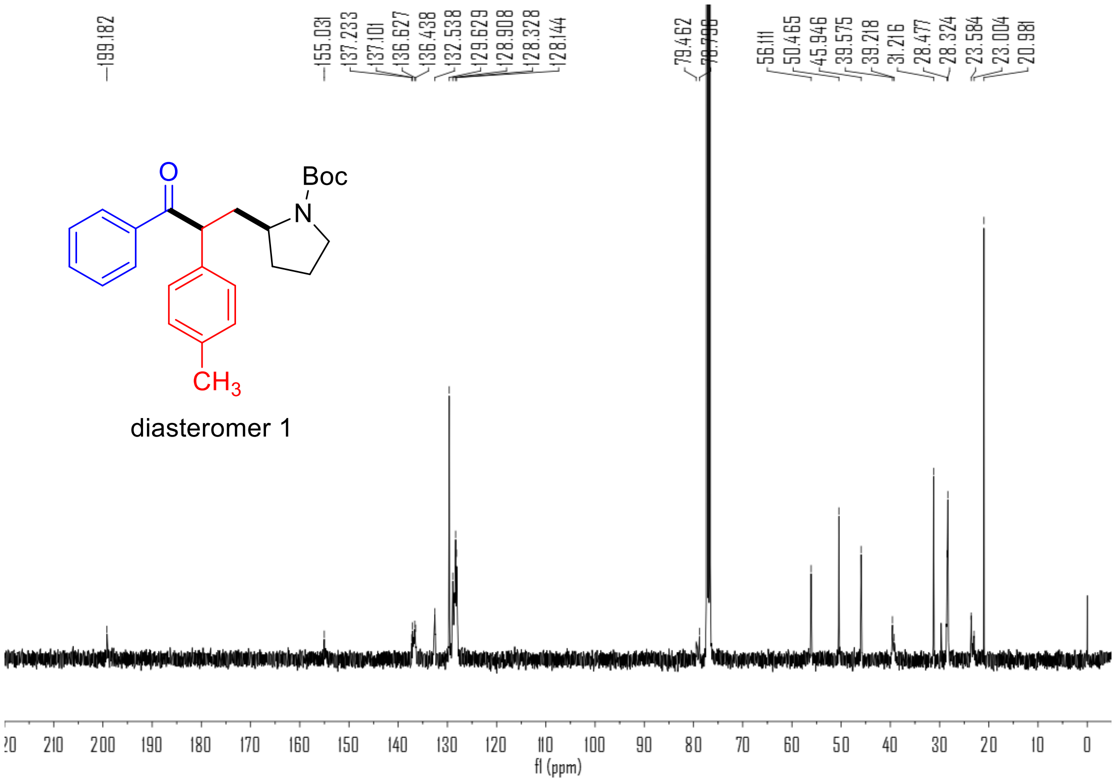

Supplementary Figure 130. ^{13}**C NMR** spectrum of **5h**

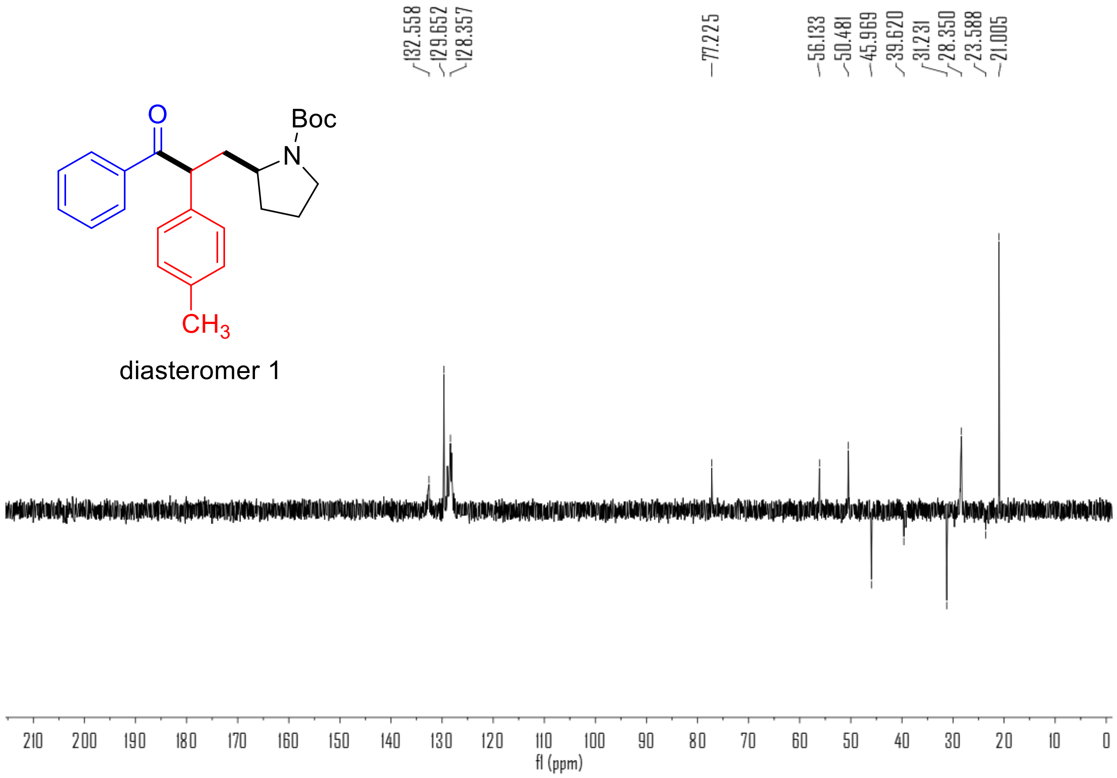

Supplementary Figure 131. DEPT135 spectrum of **5h (diasteromer 1)**

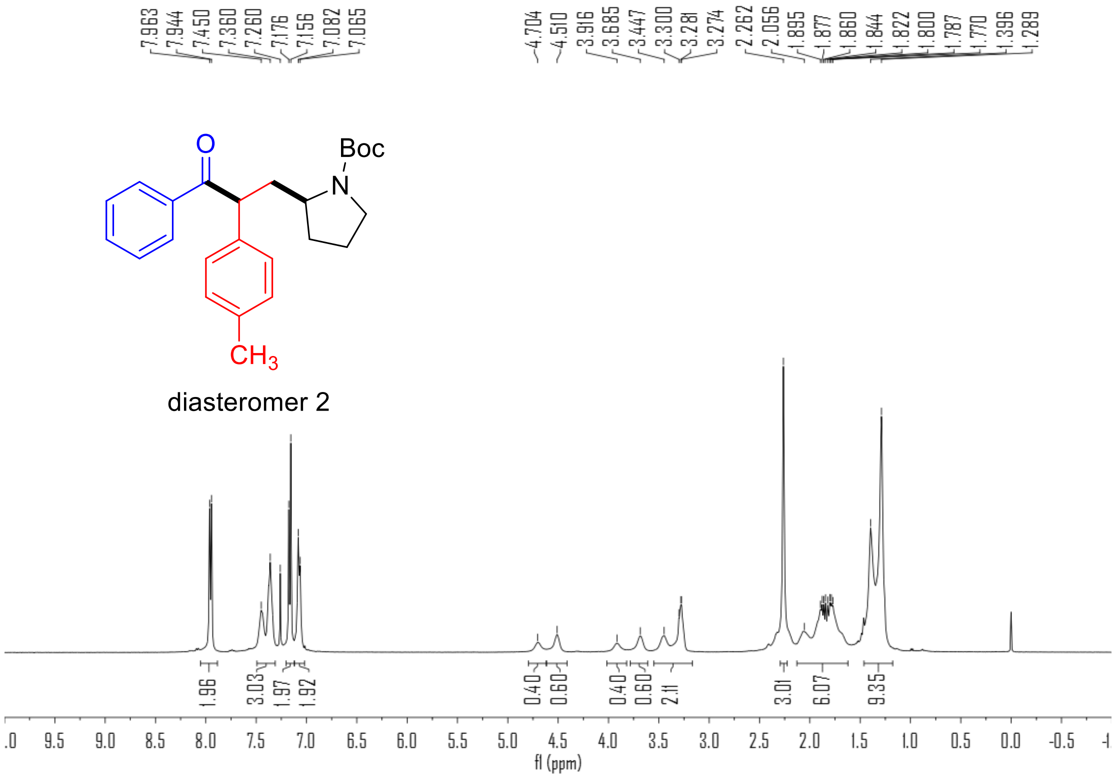

Supplementary Figure 132. ^{1}H NMR spectrum of **5h'**

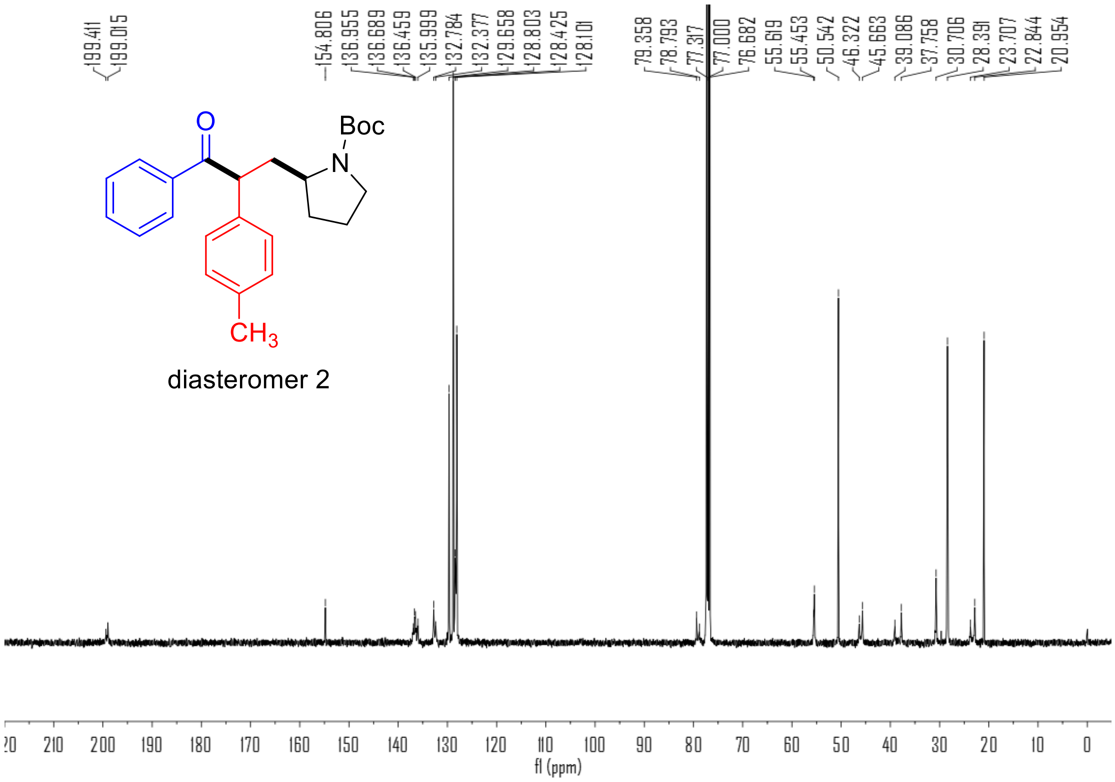

Supplementary Figure 133. ^{13}C NMR spectrum of **5h'**

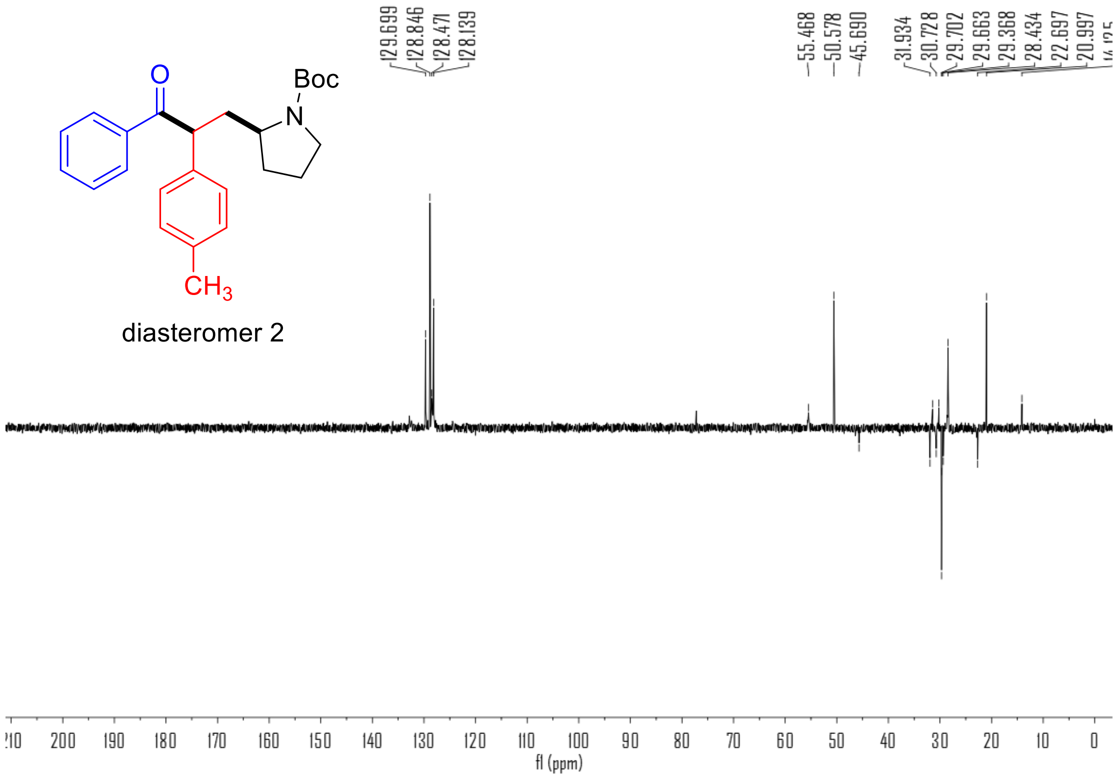

Supplementary Figure 134. DEPT135 spectrum of **5h**

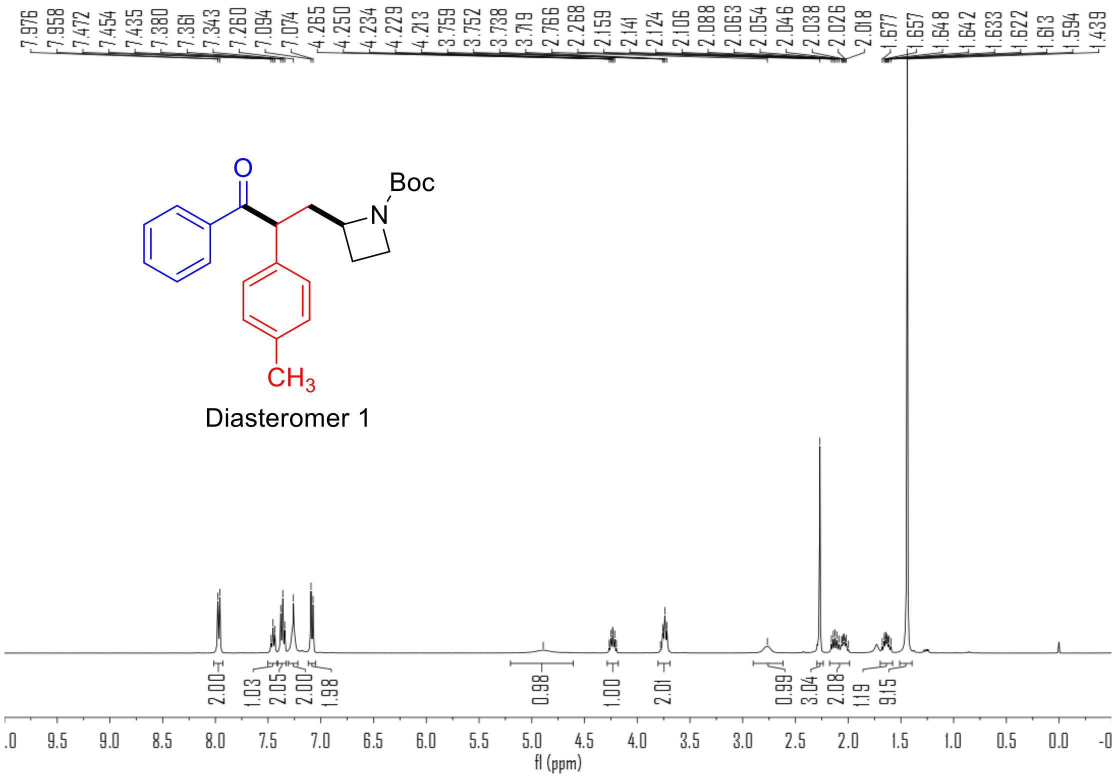

Supplementary Figure 135. ^{1}**H NMR** spectrum of **5i**

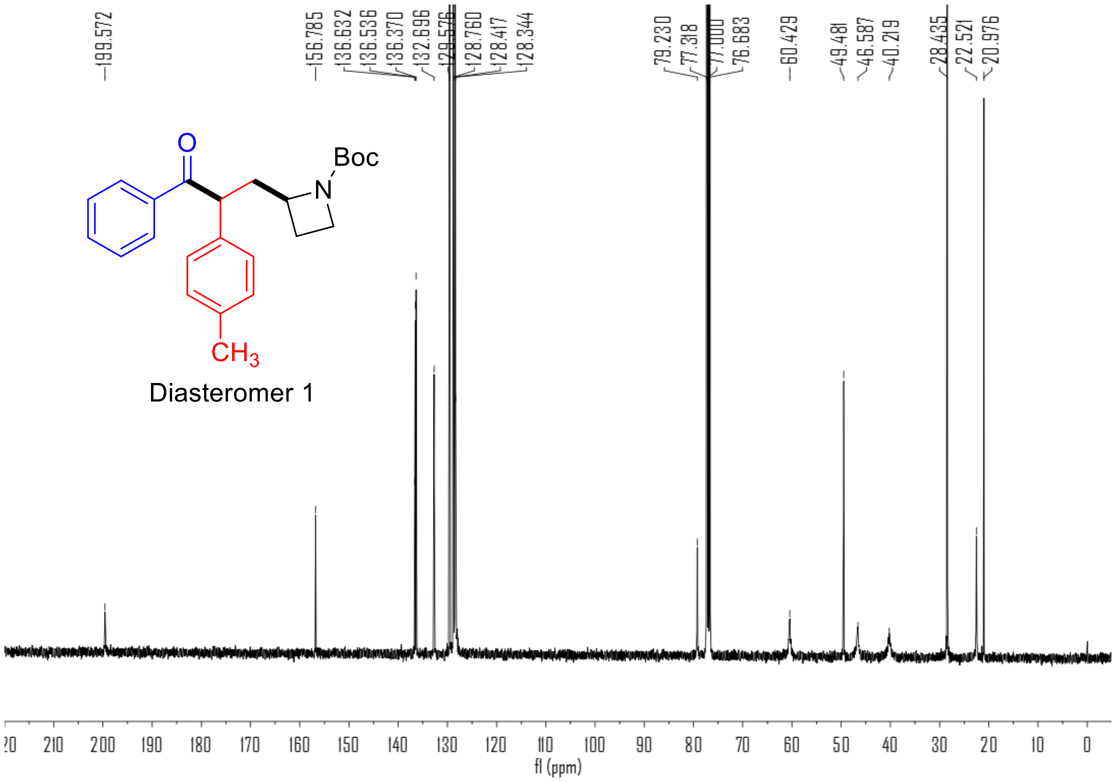

Supplementary Figure 136. ^{13}**C NMR** spectrum of **5i**

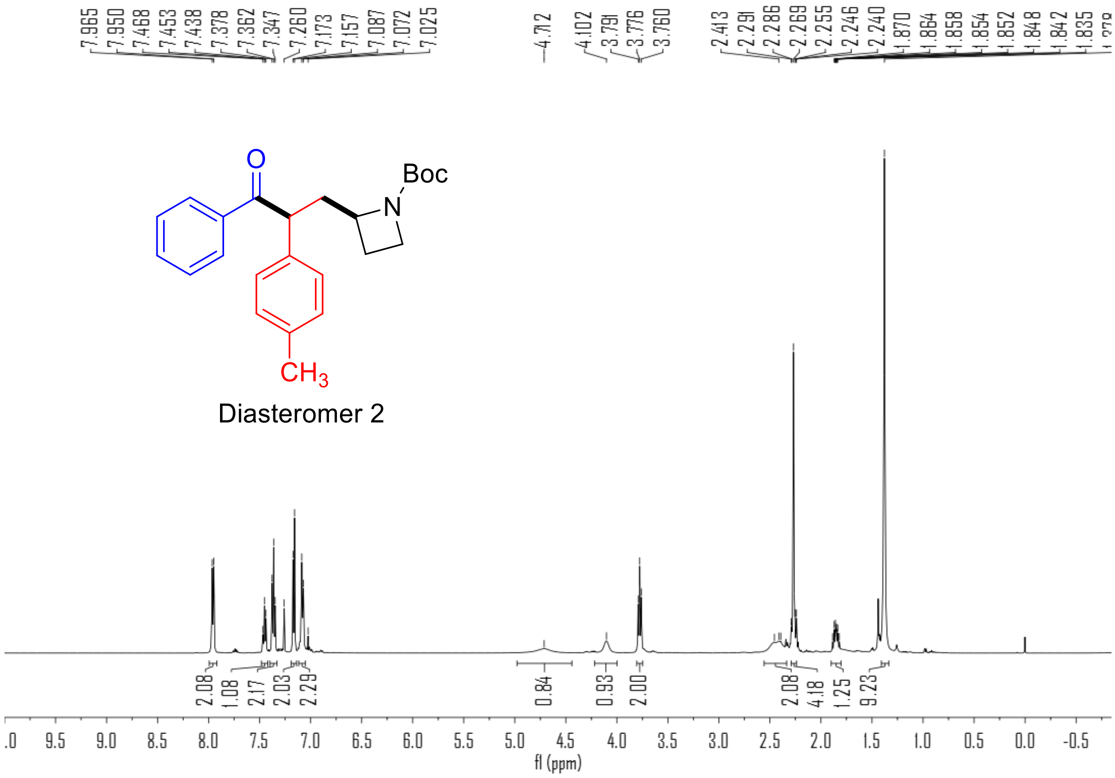

Supplementary Figure 137. ^{1}**H NMR** spectrum of **5i'**

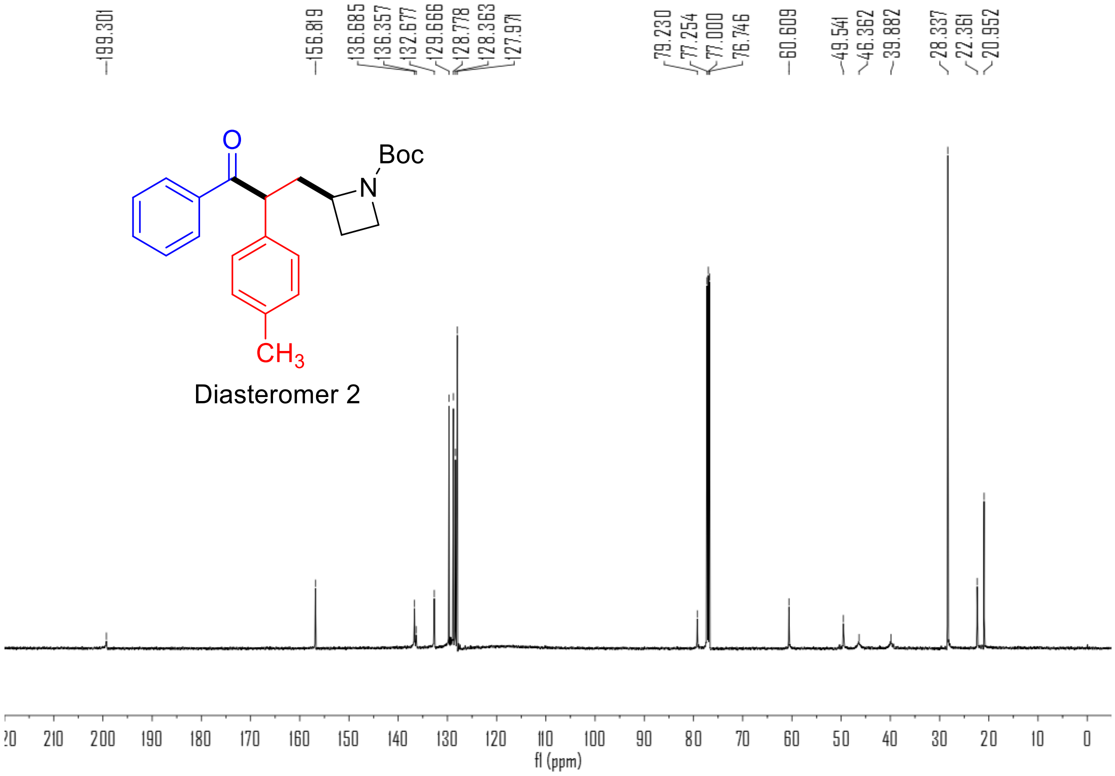

Supplementary Figure 138. ^{13}**C NMR** spectrum of **5i'**

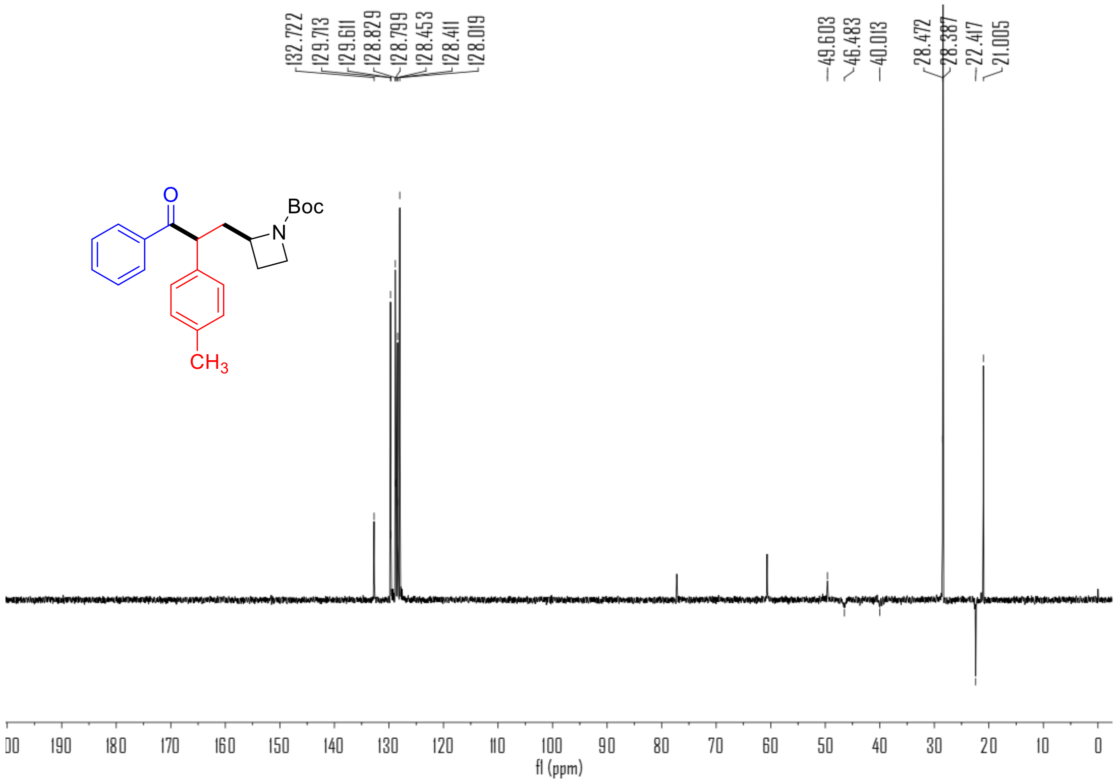

Supplementary Figure 139. DEPT135 spectrum of **5i**

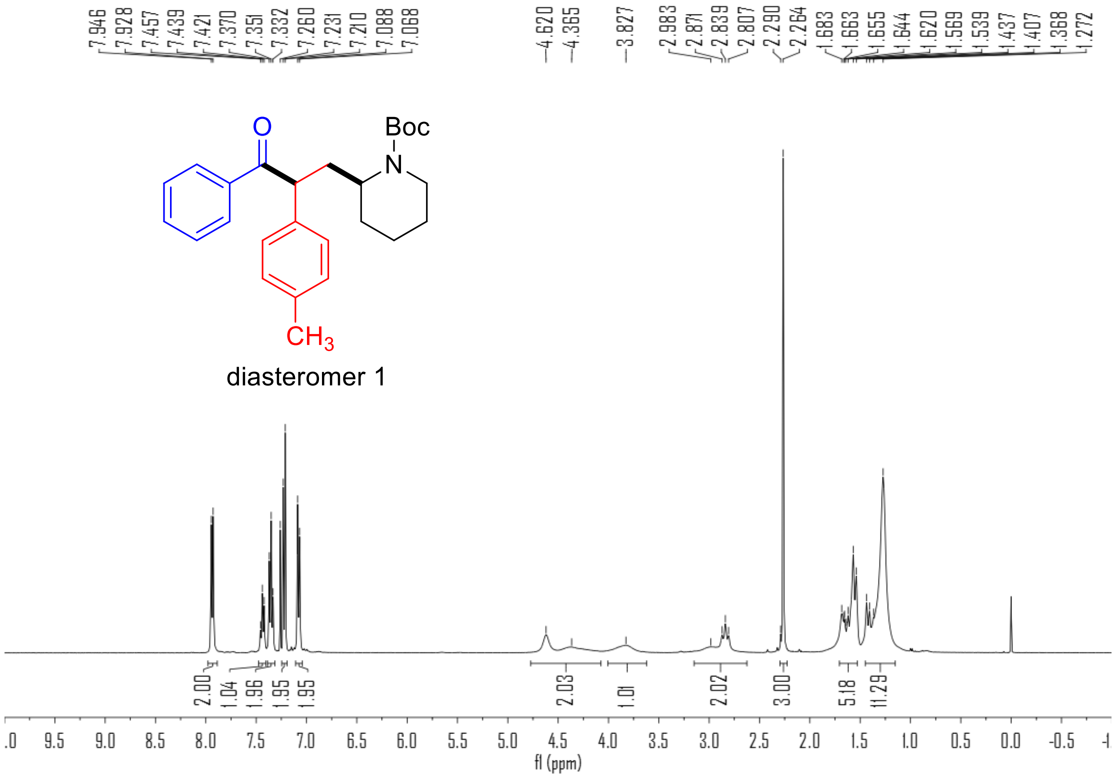

Supplementary Figure 140. ^{1}**H NMR** spectrum of **5j**

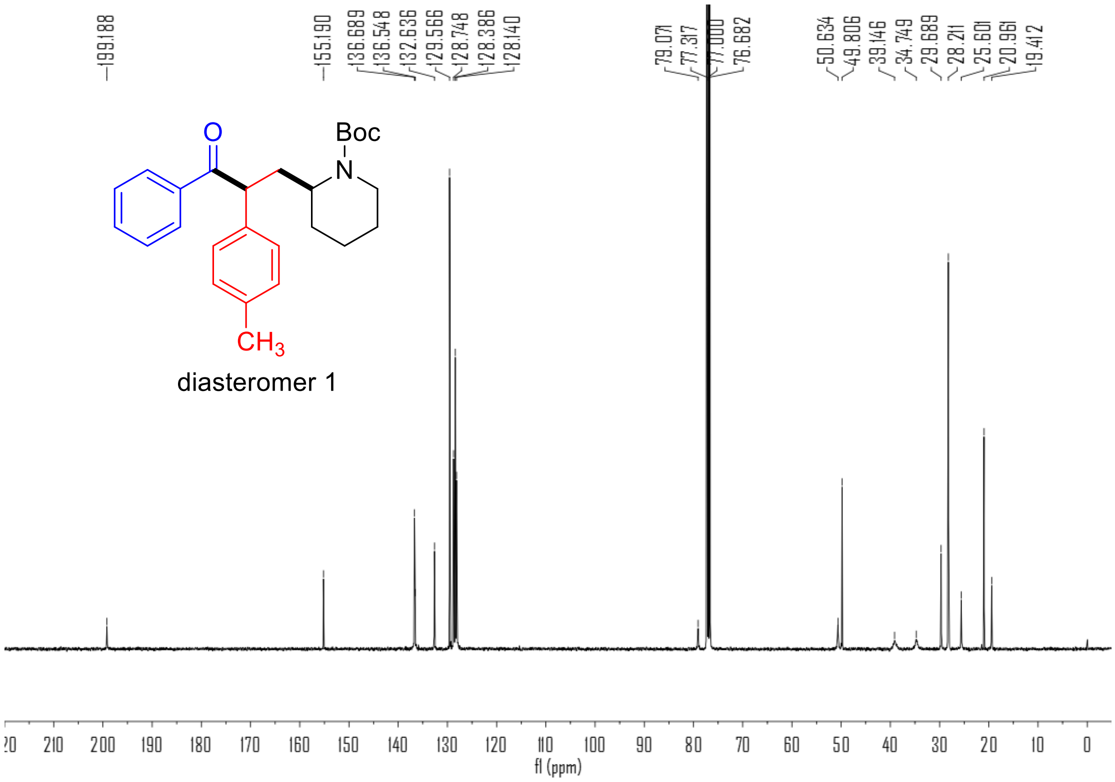

Supplementary Figure 141. ^{13}**C NMR** spectrum of **5j**

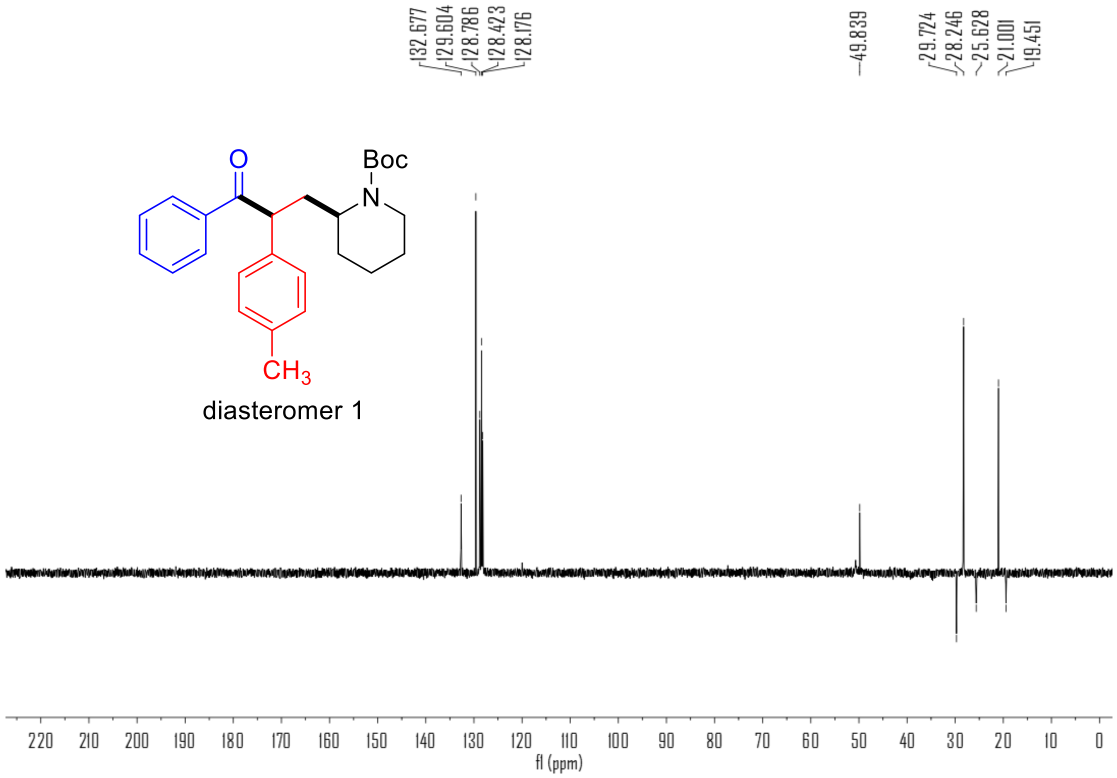

Supplementary Figure 142. DEPT135 spectrum of **5j**

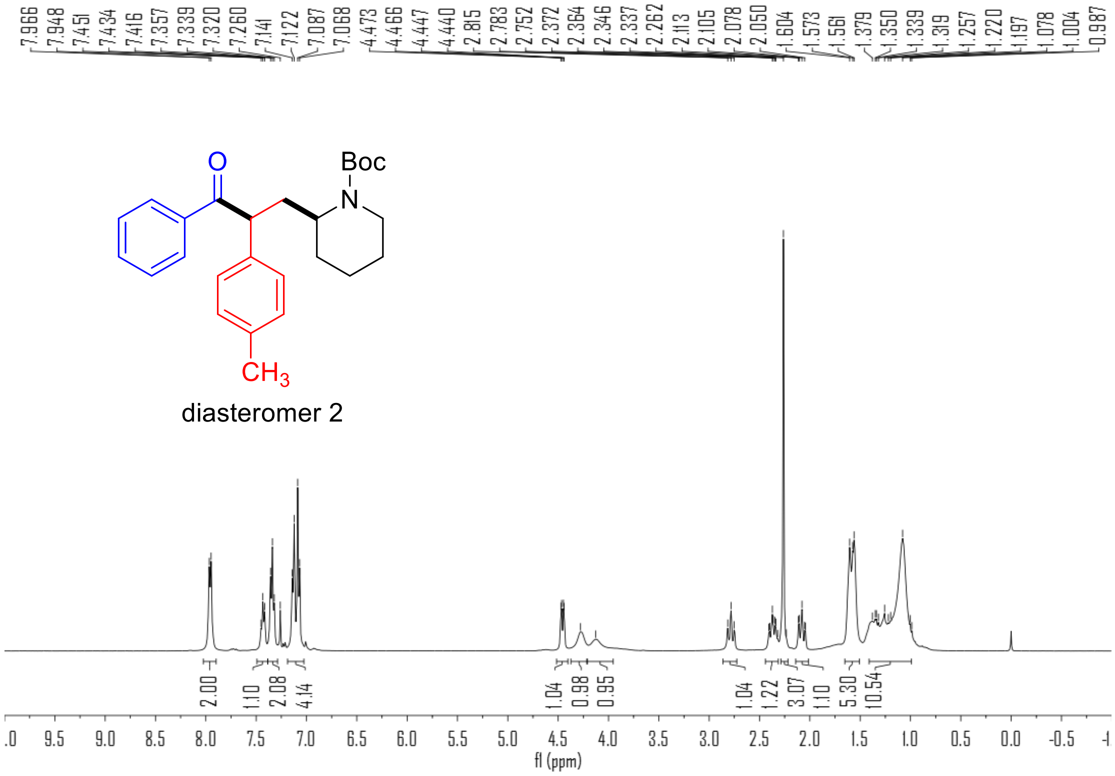

Supplementary Figure 143. **^{1}H NMR** spectrum of **5j'**

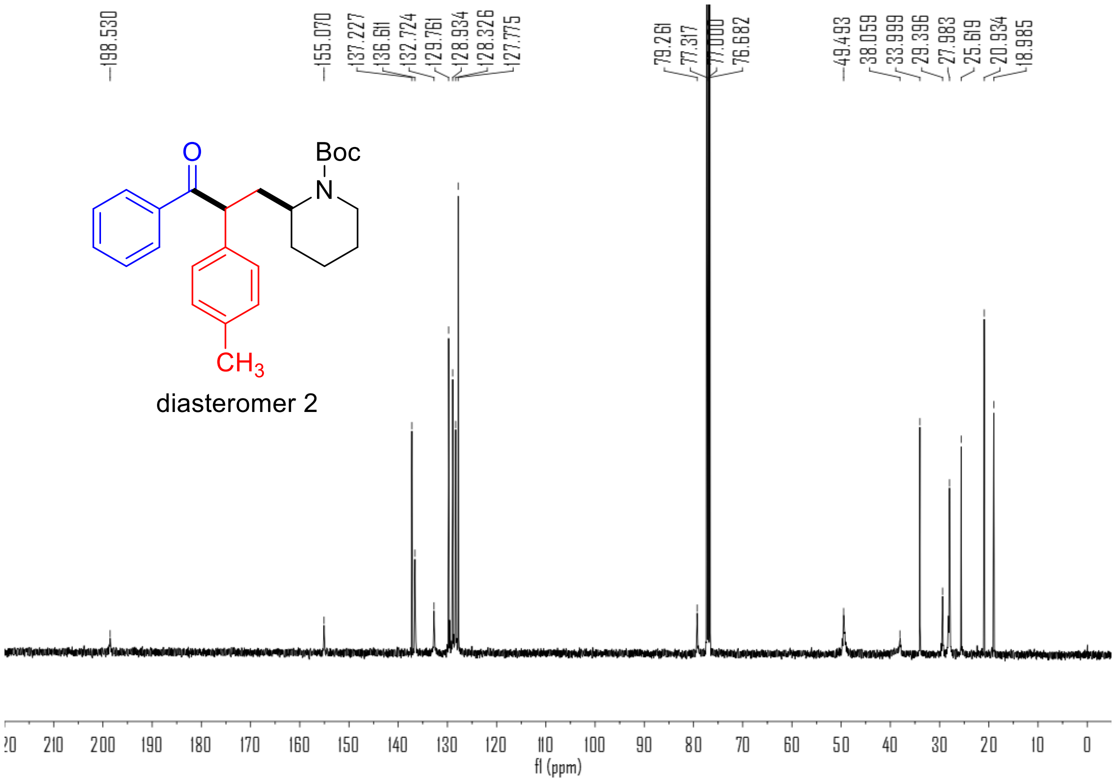

Supplementary Figure 144. **^{13}C NMR** spectrum of **5j'**

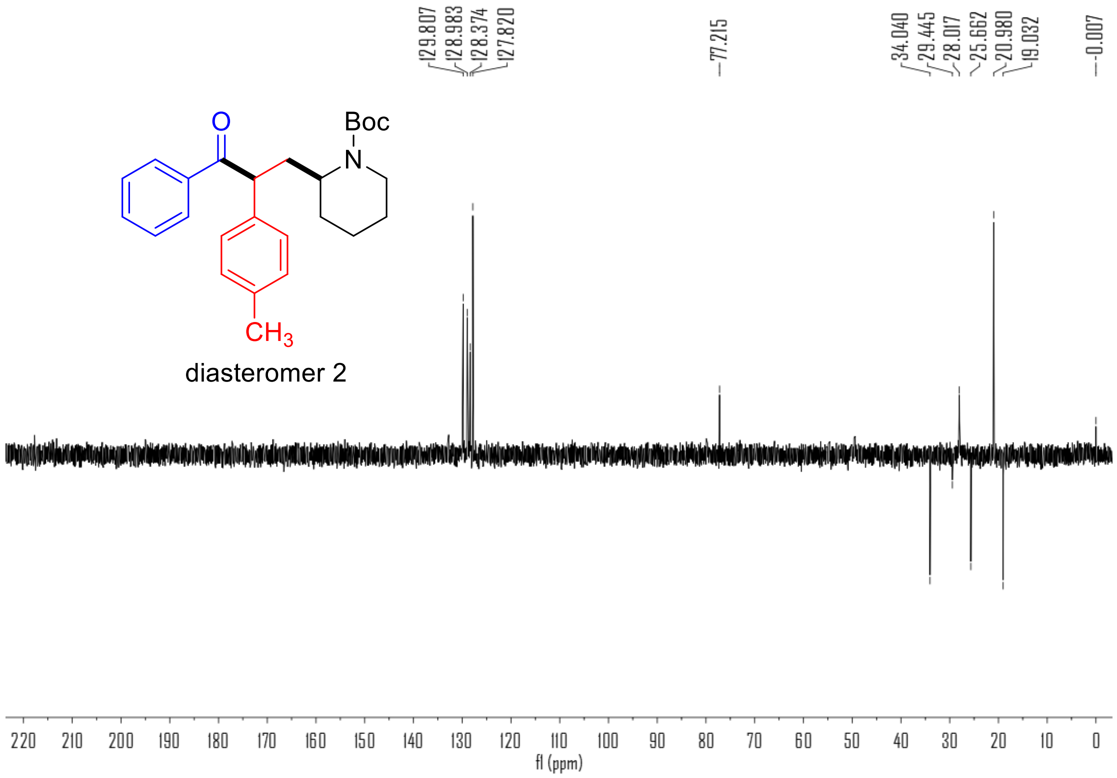

Supplementary Figure 145. DEPT135 spectrum of **5j'**

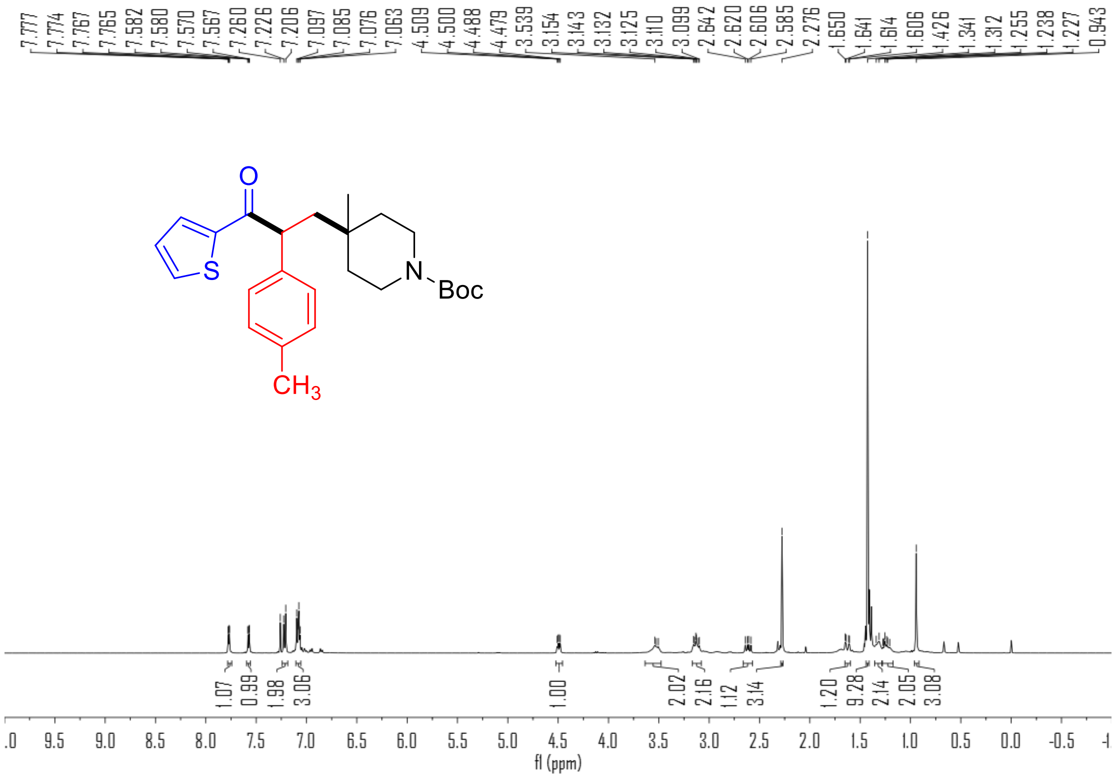

Supplementary Figure 146. ^{1}**H NMR** spectrum of **5k**

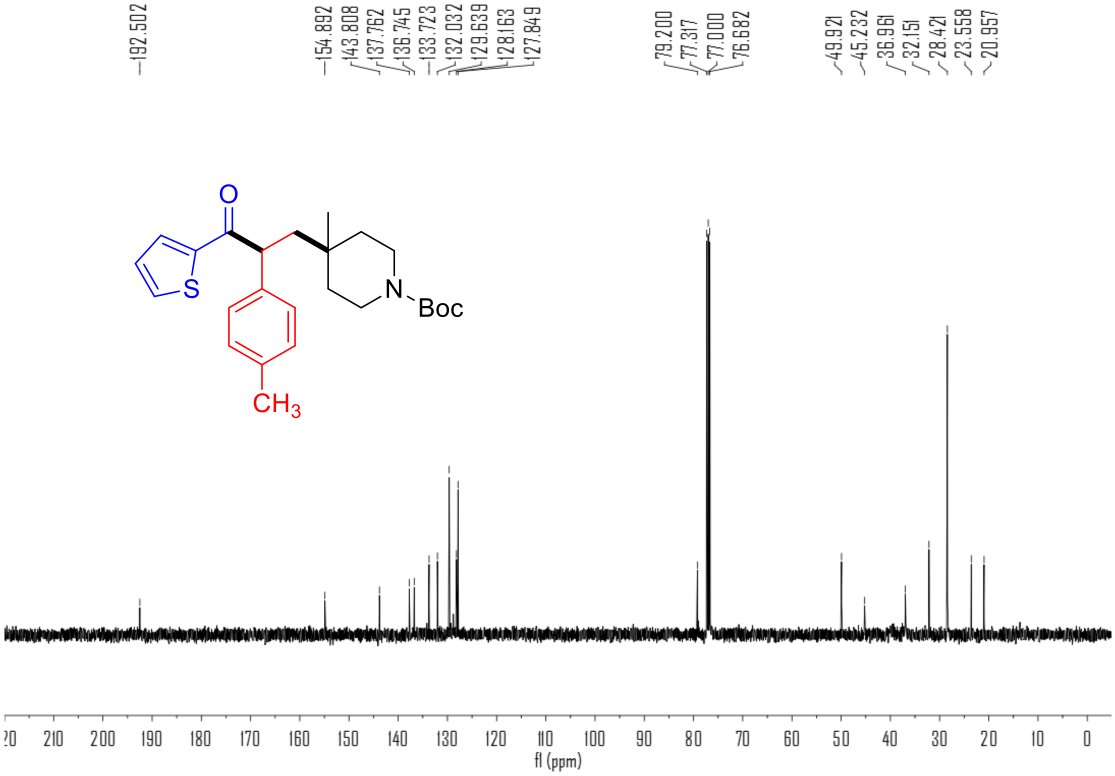

Supplementary Figure 147. ^{13}**C NMR** spectrum of **5k**

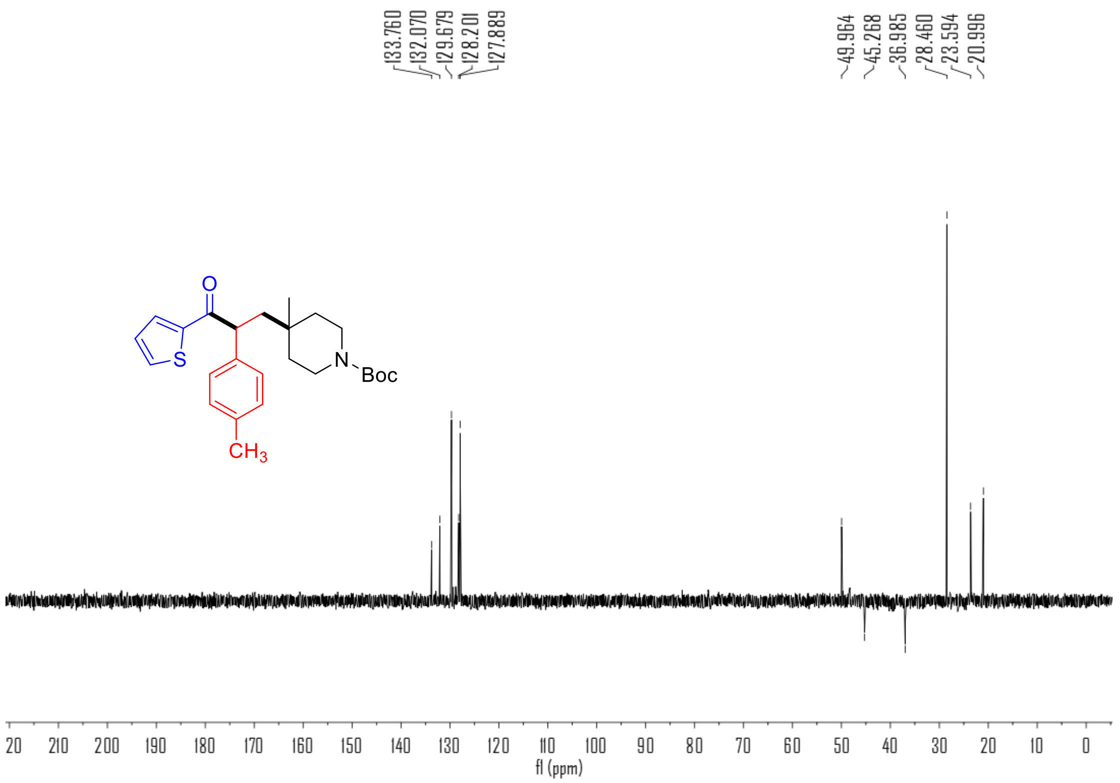

Supplementary Figure 148. DEPT135 spectrum of **5k**

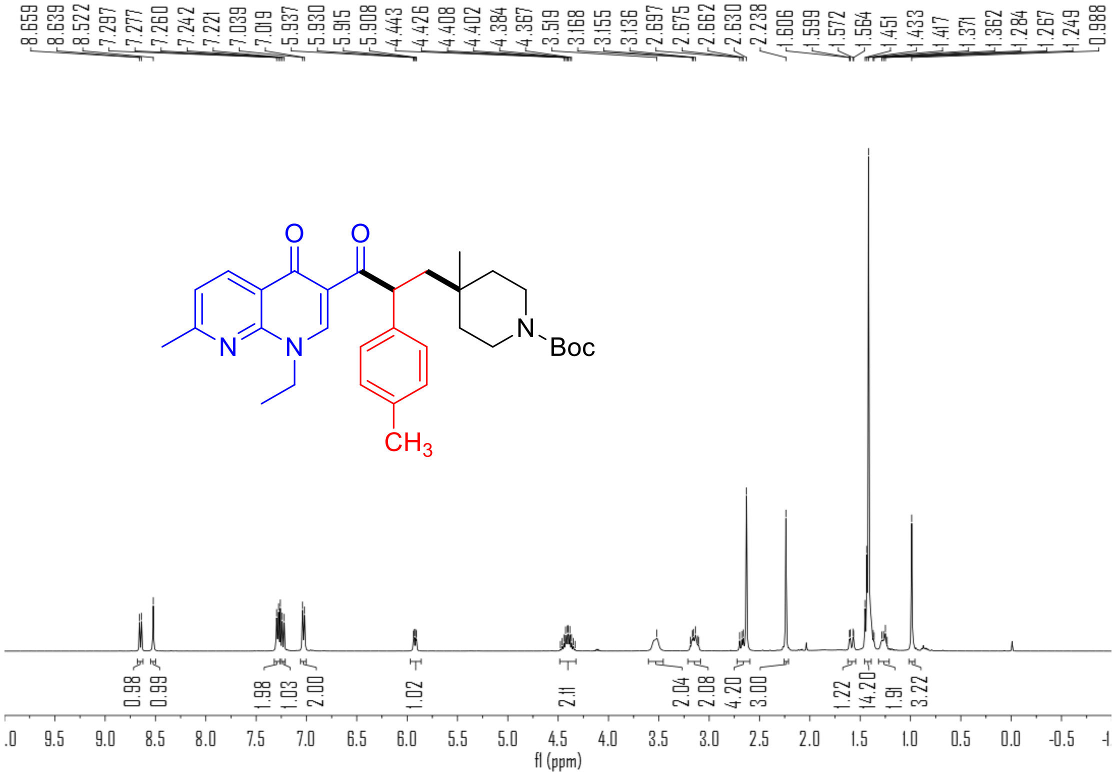

Supplementary Figure 149. ^{1}H NMR spectrum of **5l**

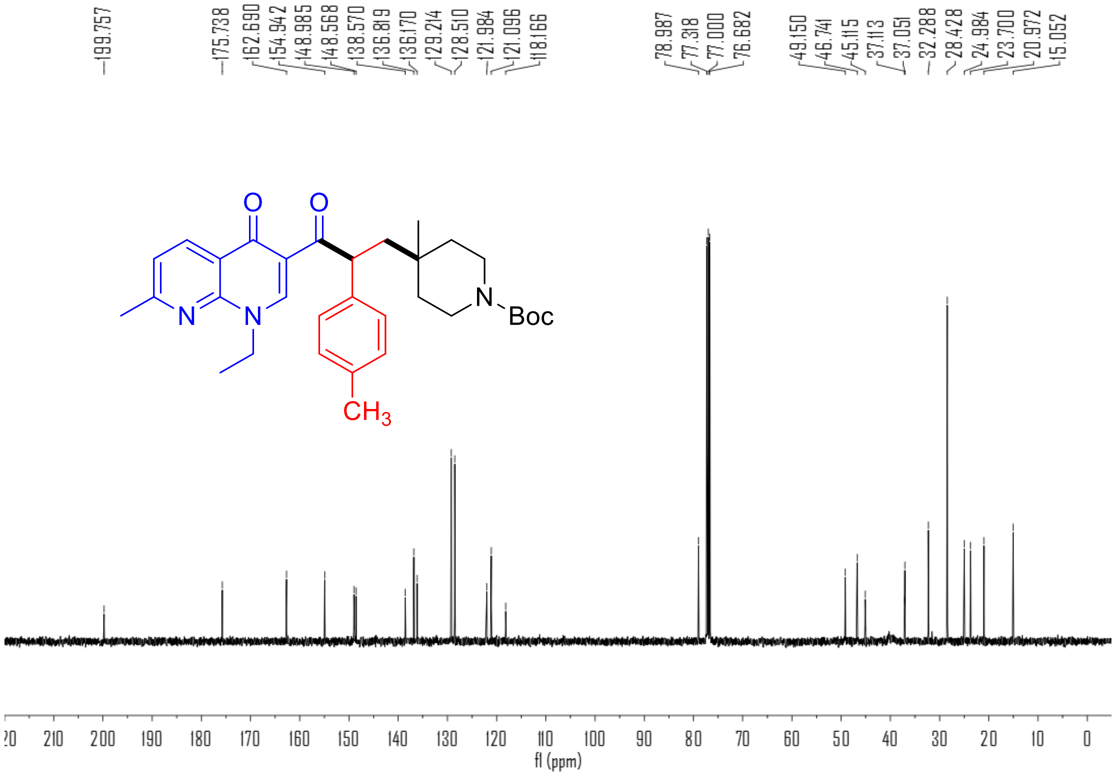

Supplementary Figure 150. ^{13}C NMR spectrum of **5l**

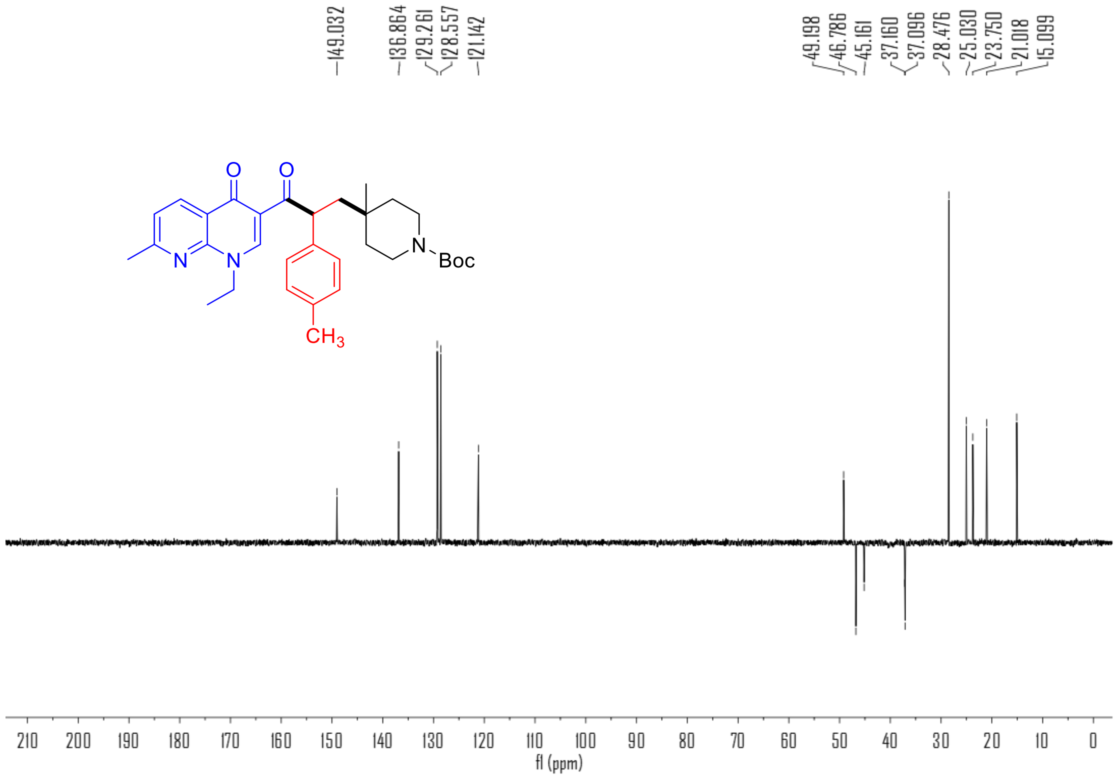

Supplementary Figure 151. DEPT135 spectrum of **5l**

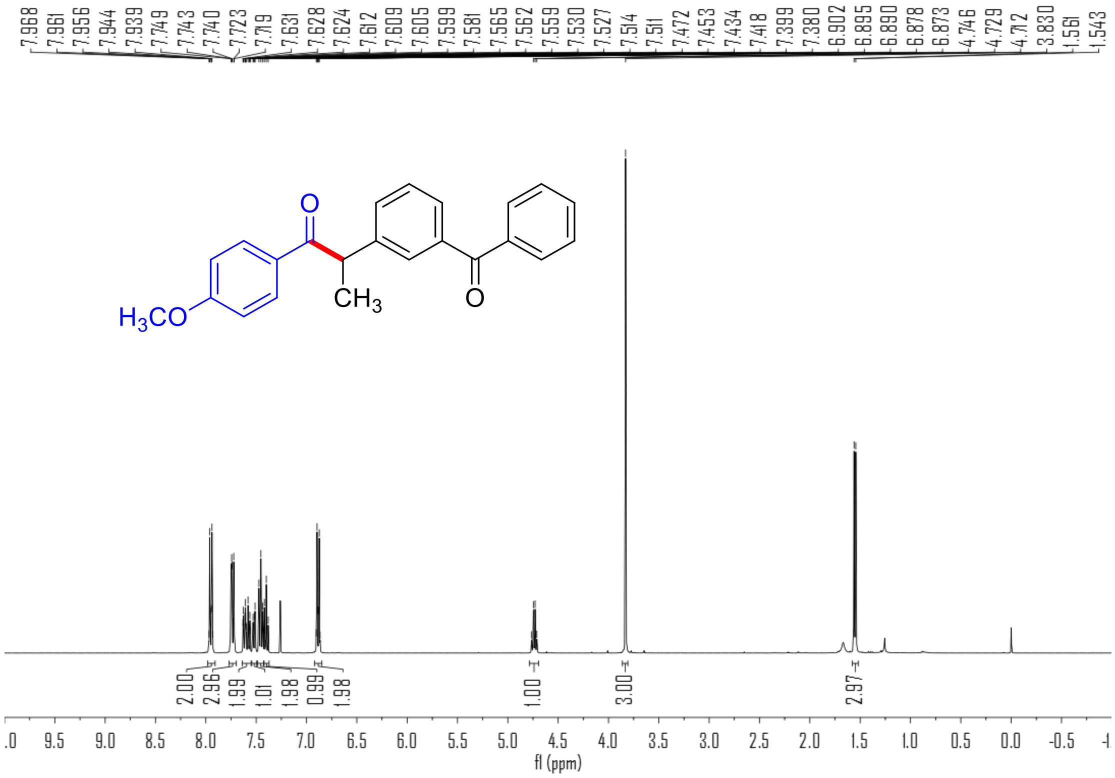

Supplementary Figure 152. ^{1}**H NMR** spectrum of **6a**

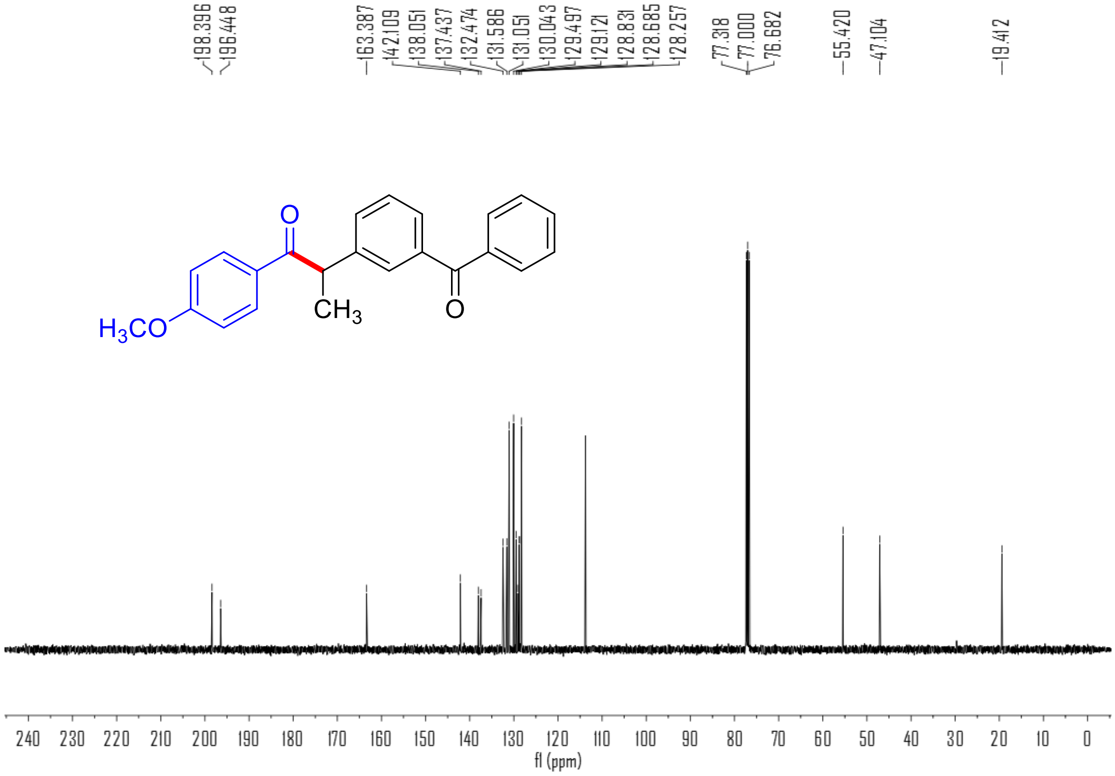

Supplementary Figure 153. ^{13}**C NMR** spectrum of **6a**

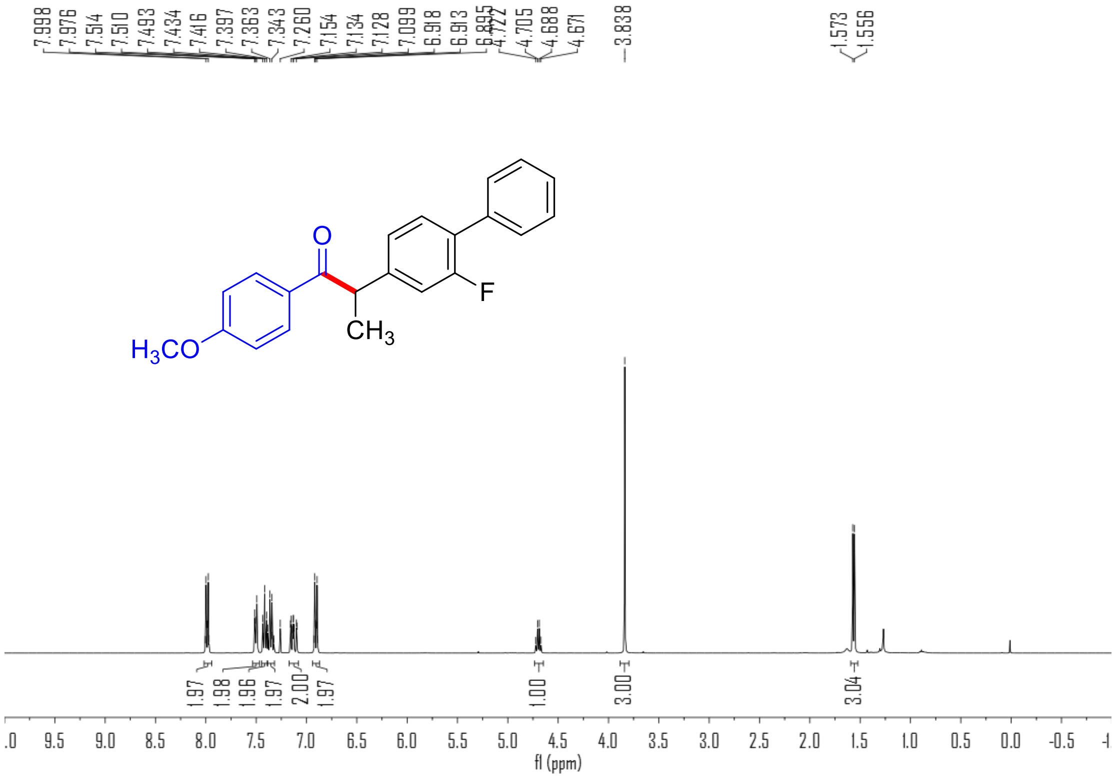

Supplementary Figure 154. ^{1}**H NMR** spectrum of **6b**

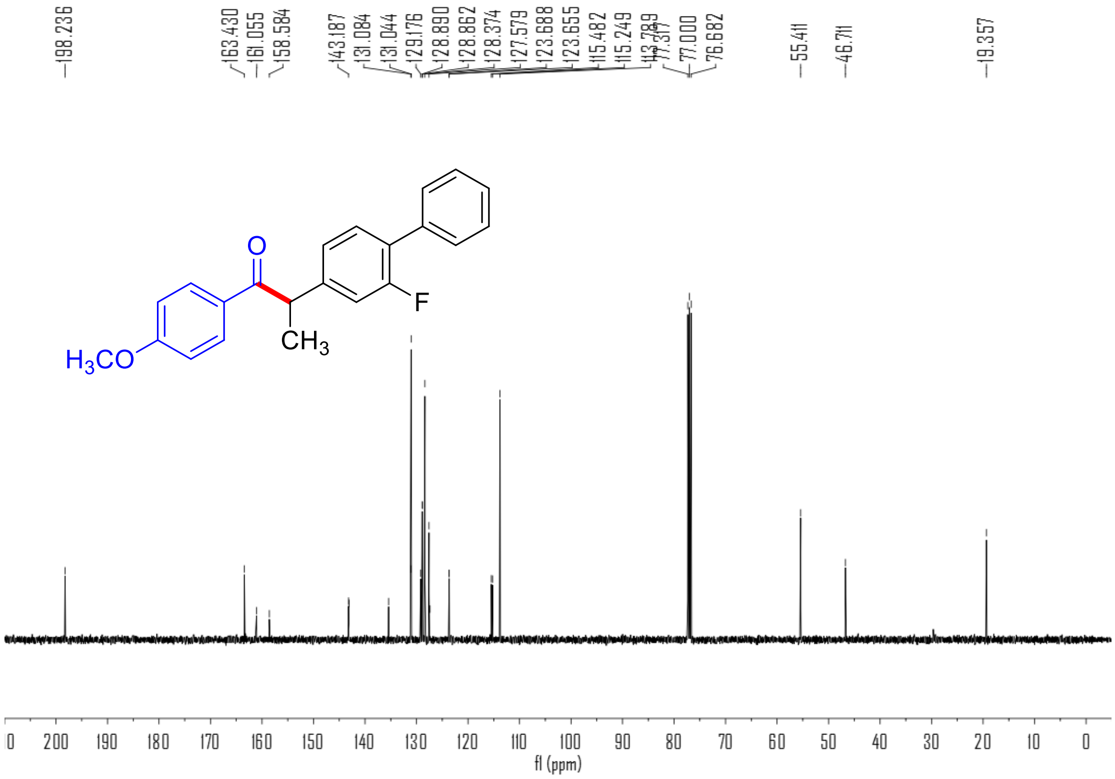

Supplementary Figure 155. ^{13}**C NMR** spectrum of **6b**

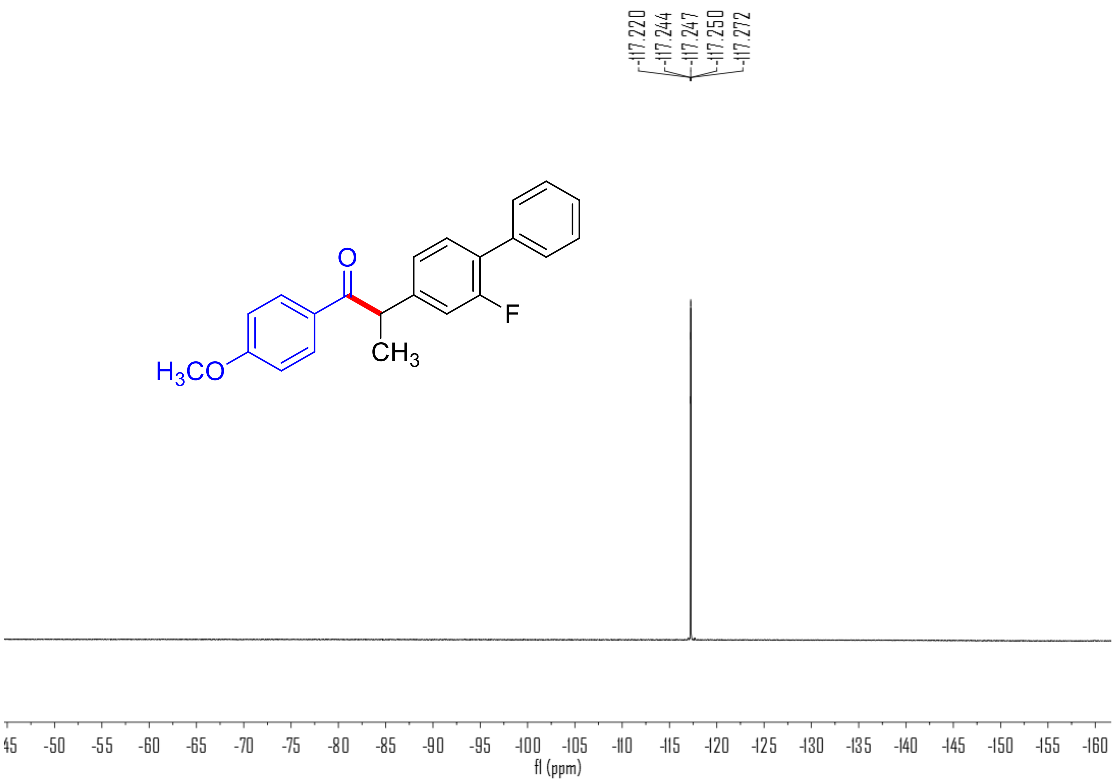

Supplementary Figure 156. ^{19}**F NMR** spectrum of **6b**

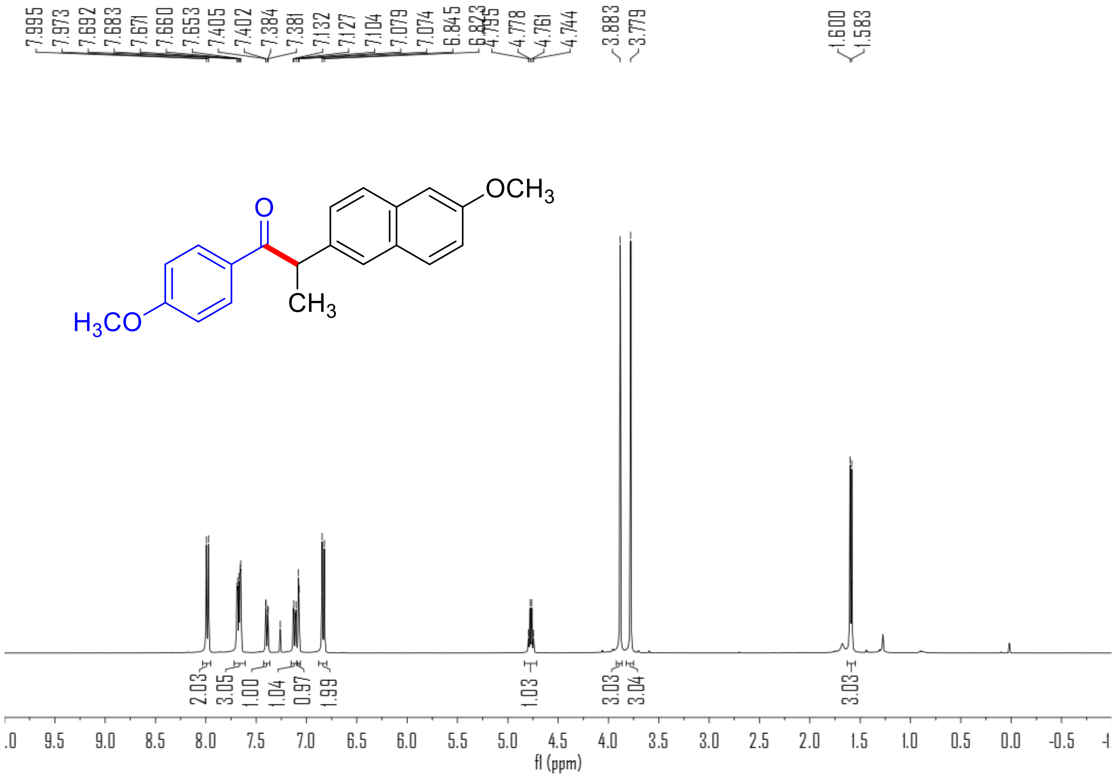

Supplementary Figure 157. ^{1}**H NMR** spectrum of **6c**

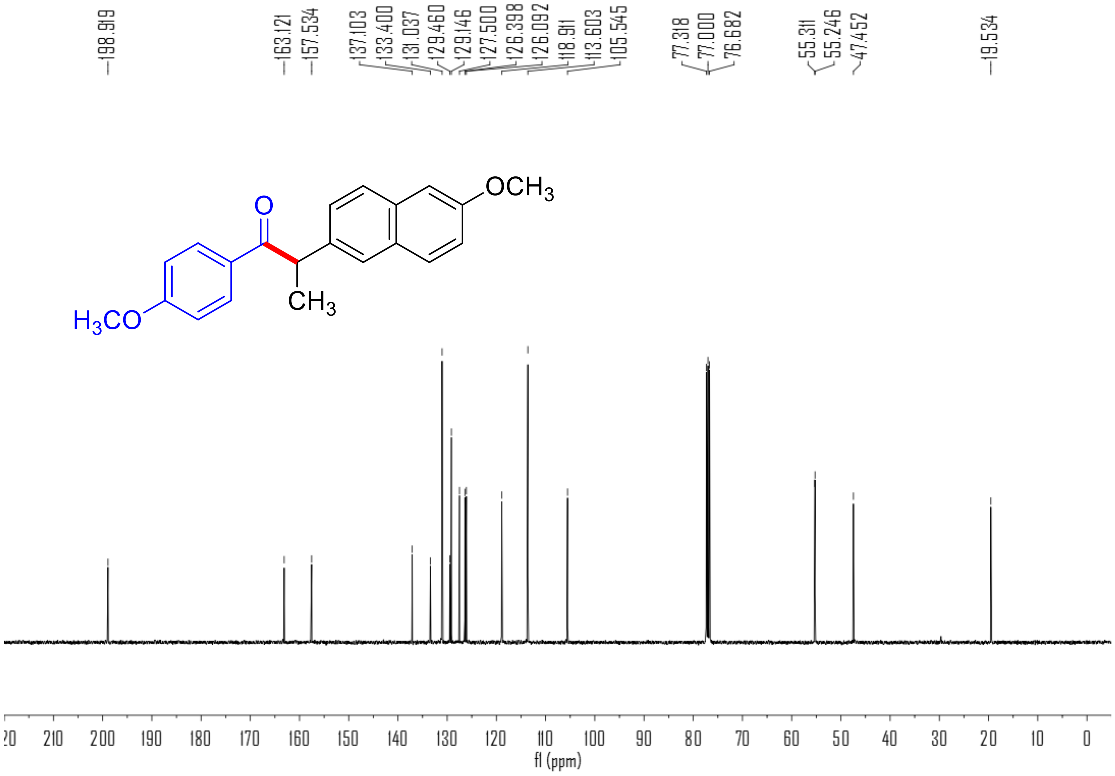

Supplementary Figure 158. ^{13}**C NMR** spectrum of **6c**

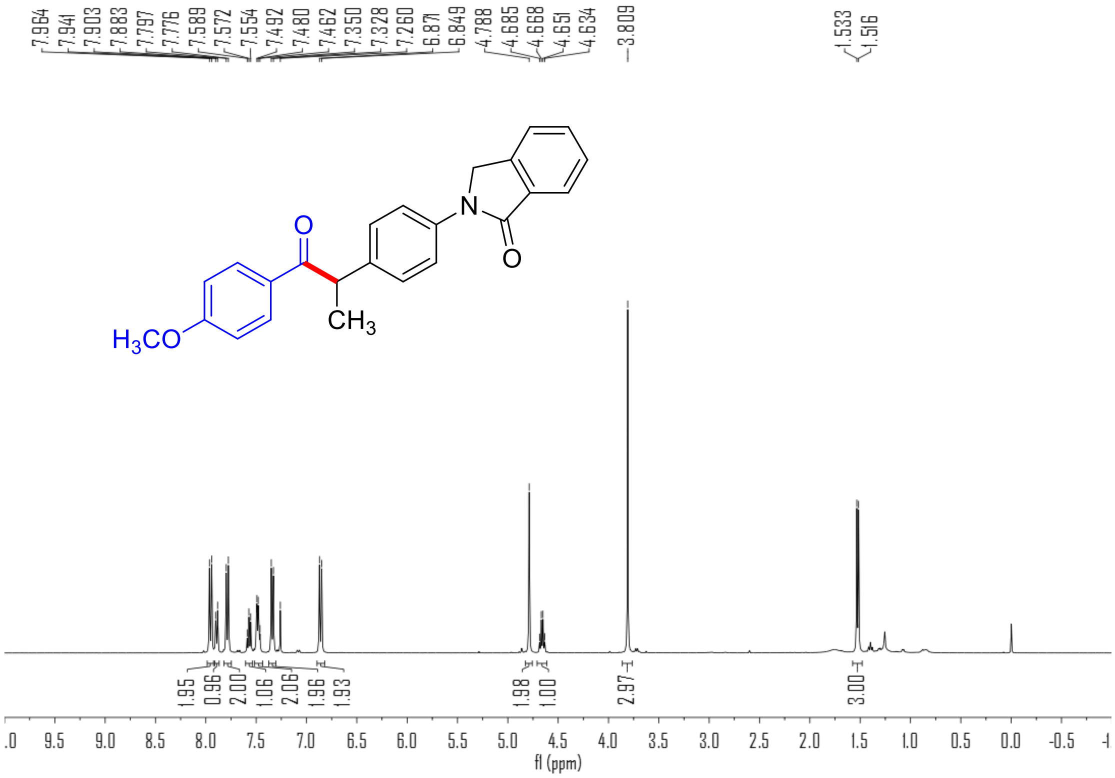

Supplementary Figure 159. ^{1}**H NMR** spectrum of **6d**

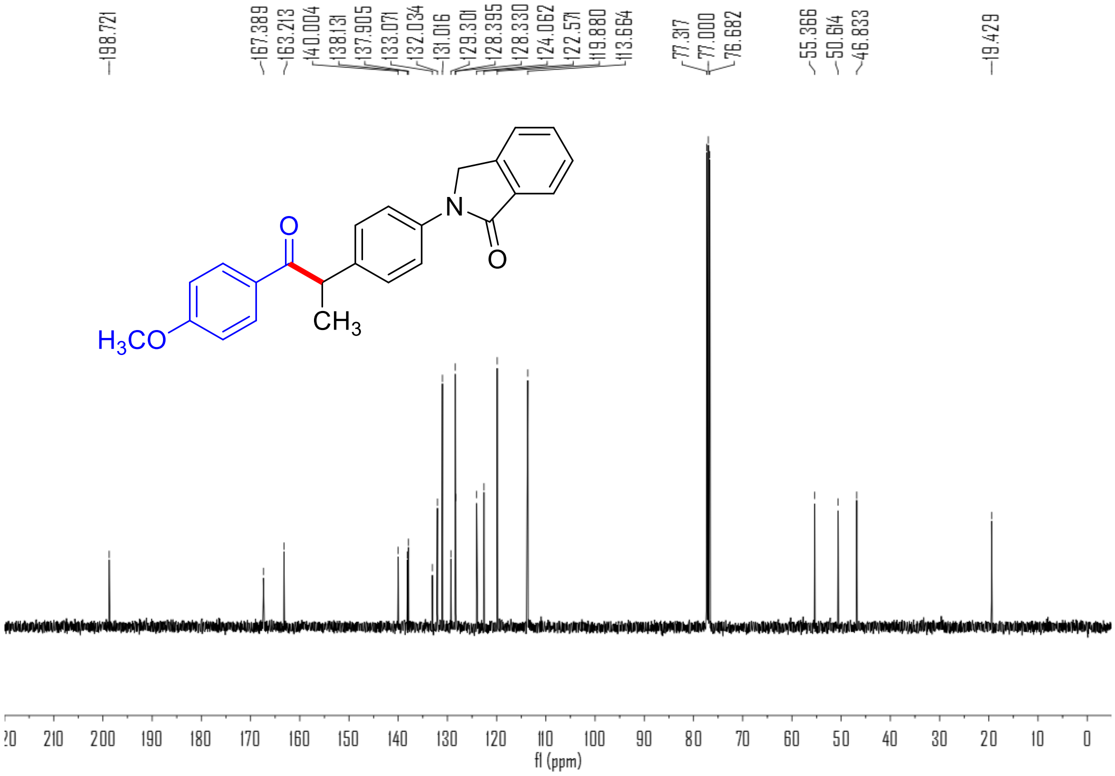

Supplementary Figure 160. ^{13}**C NMR** spectrum of **6d**

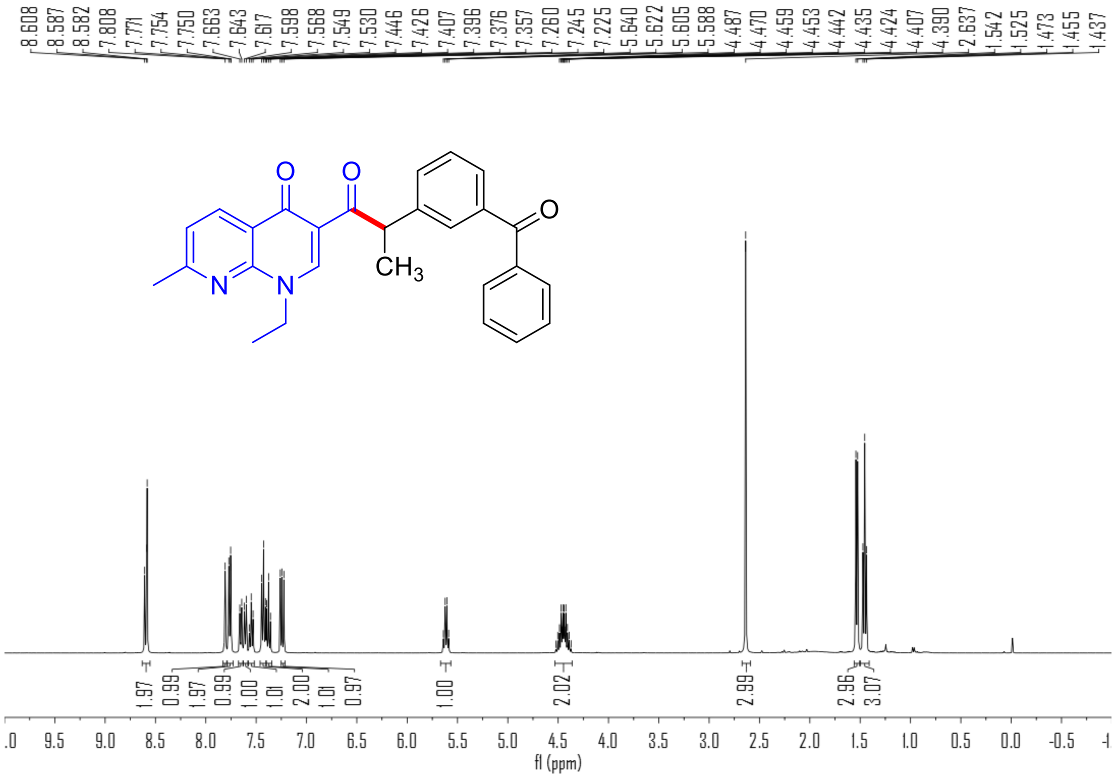

Supplementary Figure 161. ^{1}**H NMR** spectrum of **6e**

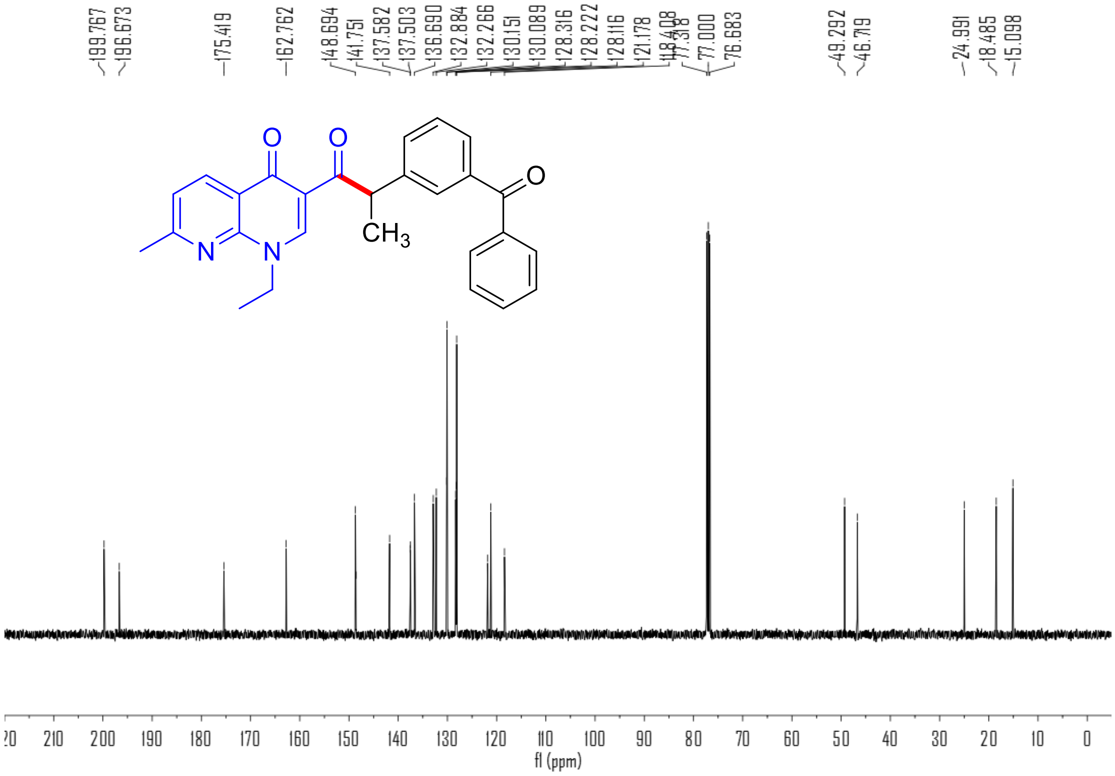

Supplementary Figure 162. ^{13}**C NMR** spectrum of **6e**

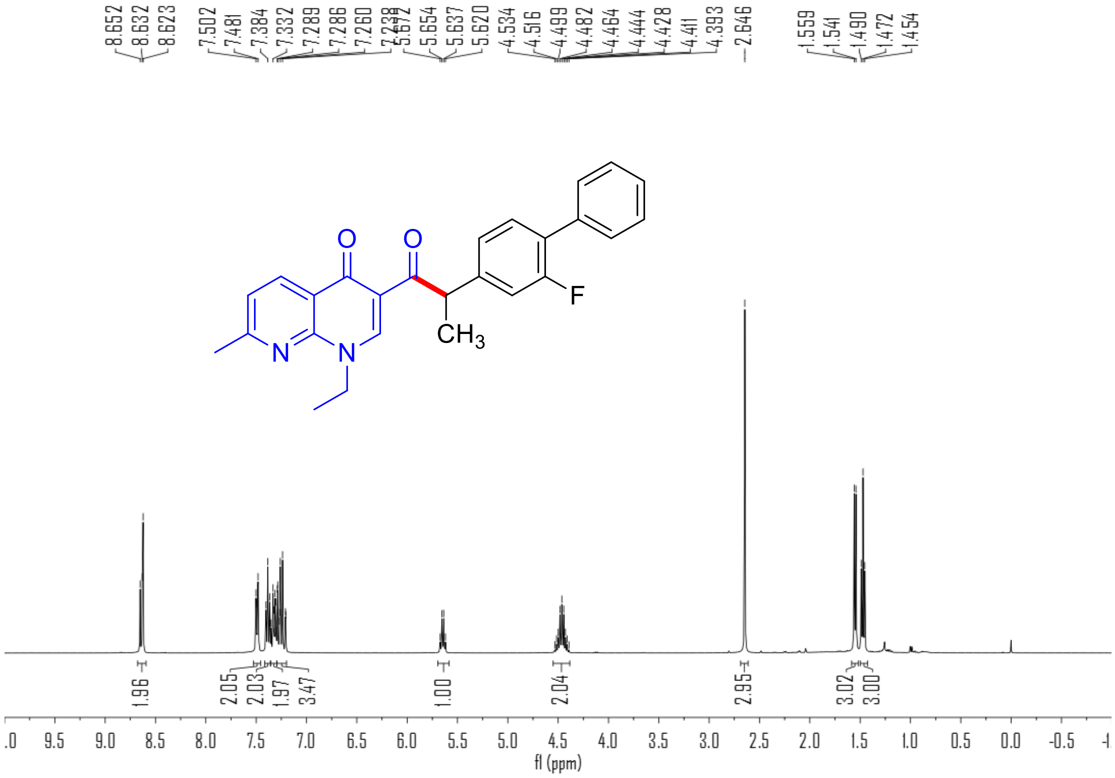

Supplementary Figure 163. ^{1}**H NMR** spectrum of **6f**

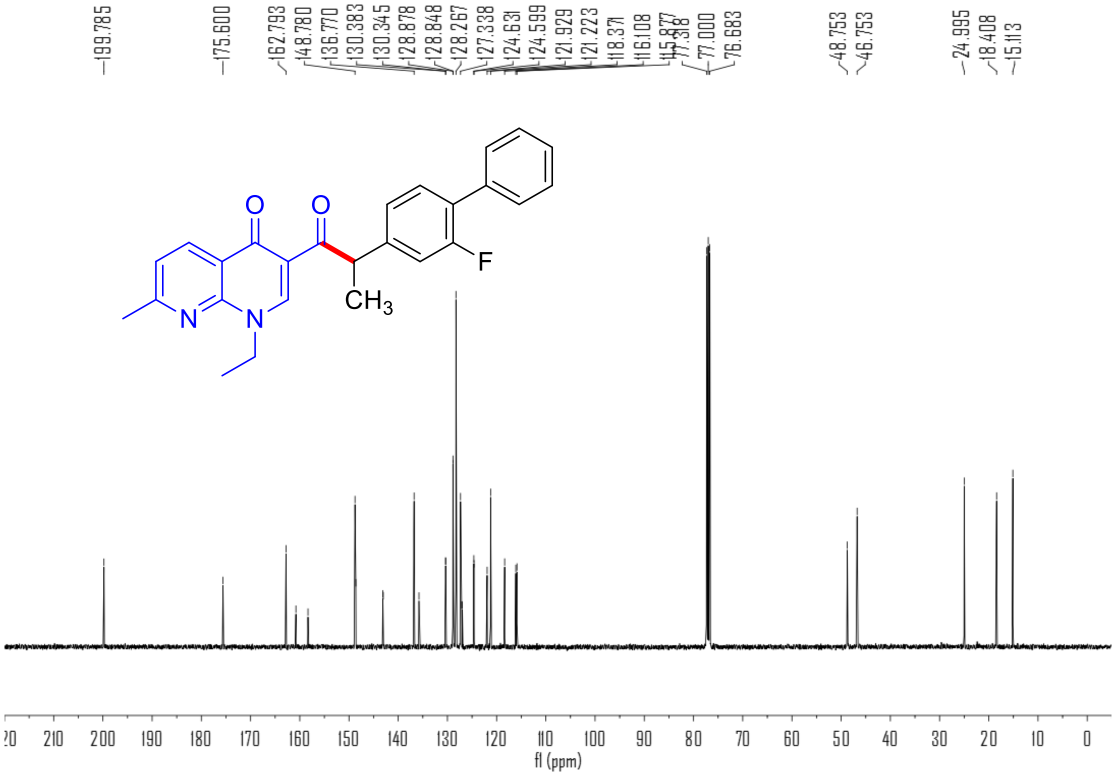

Supplementary Figure 164. ^{13}**C NMR** spectrum of **6f**

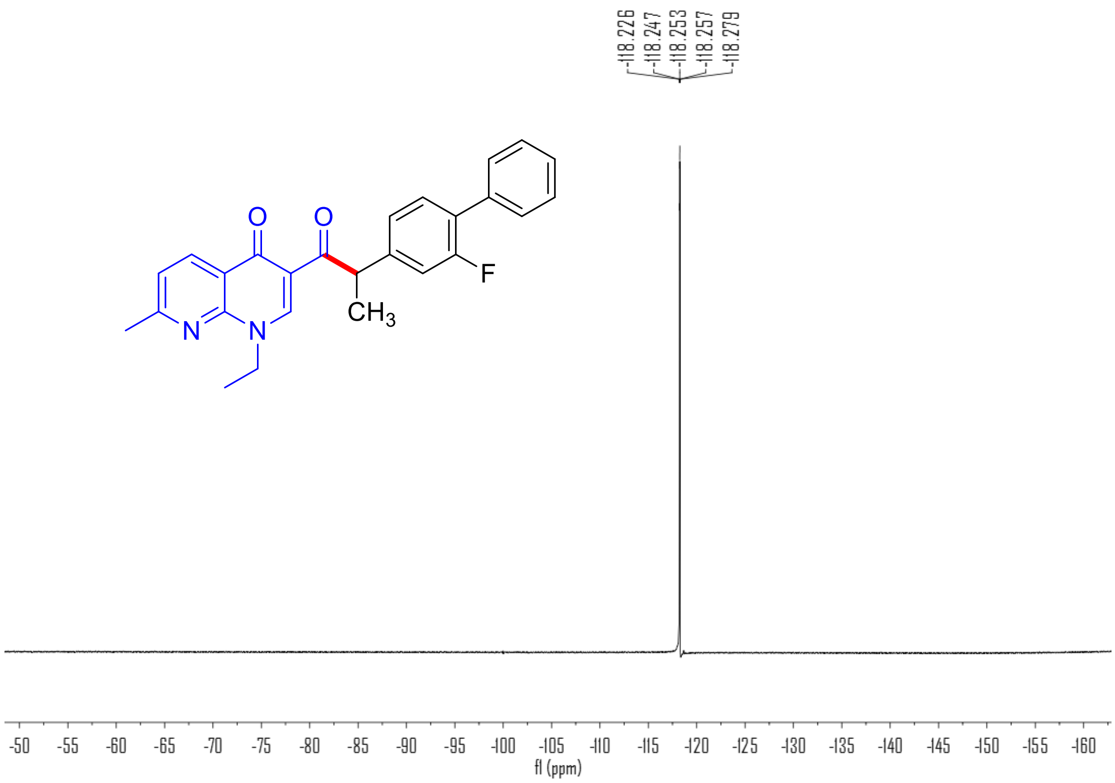

Supplementary Figure 165. ^{19}**F NMR** spectrum of **6f**

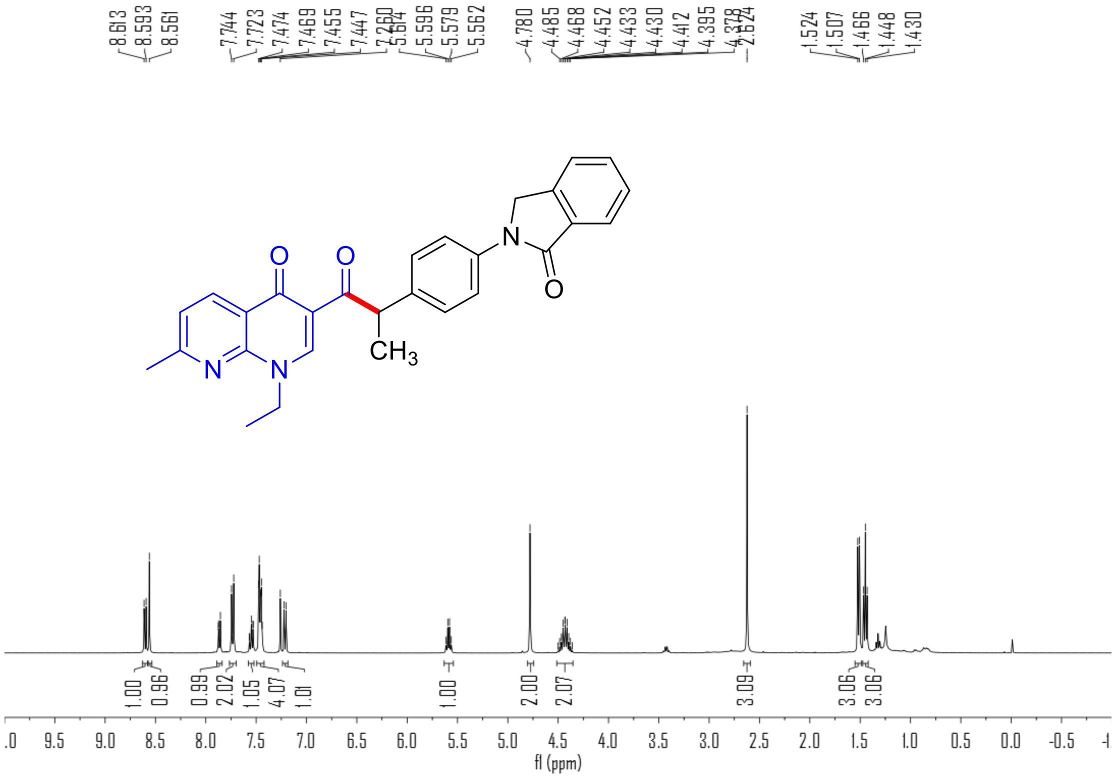

Supplementary Figure 166. ^{1}H NMR spectrum of **6g**

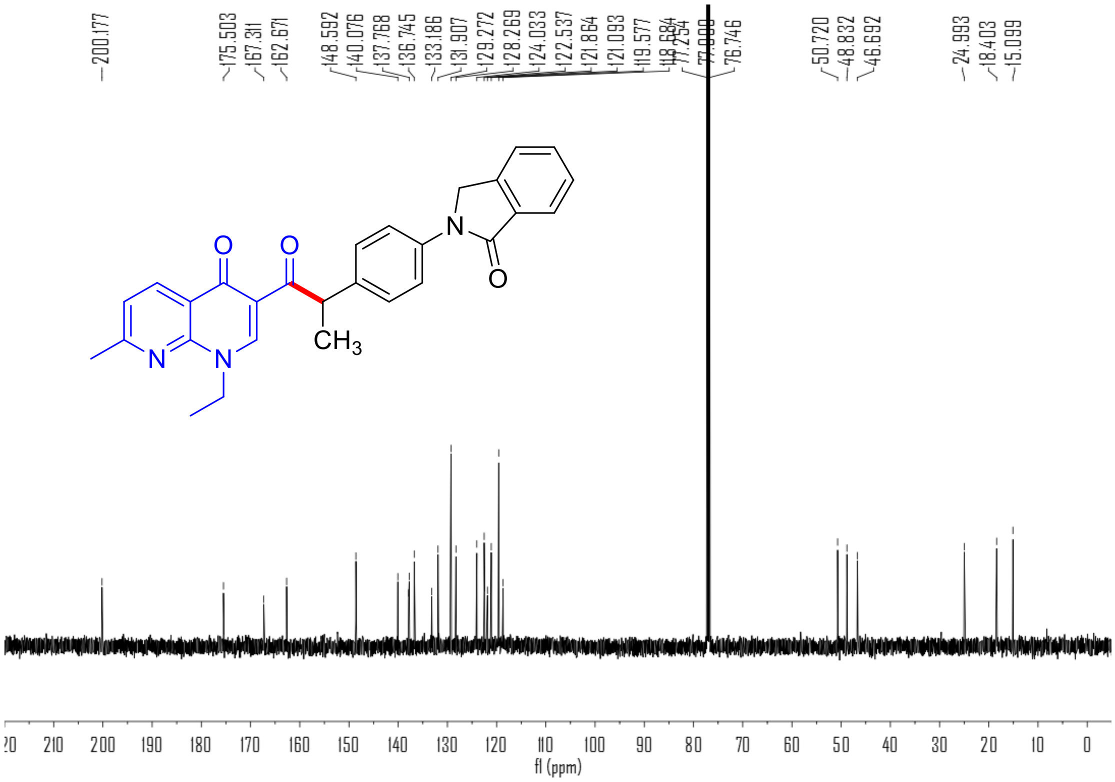

Supplementary Figure 167. ^{13}C NMR spectrum of **6g**

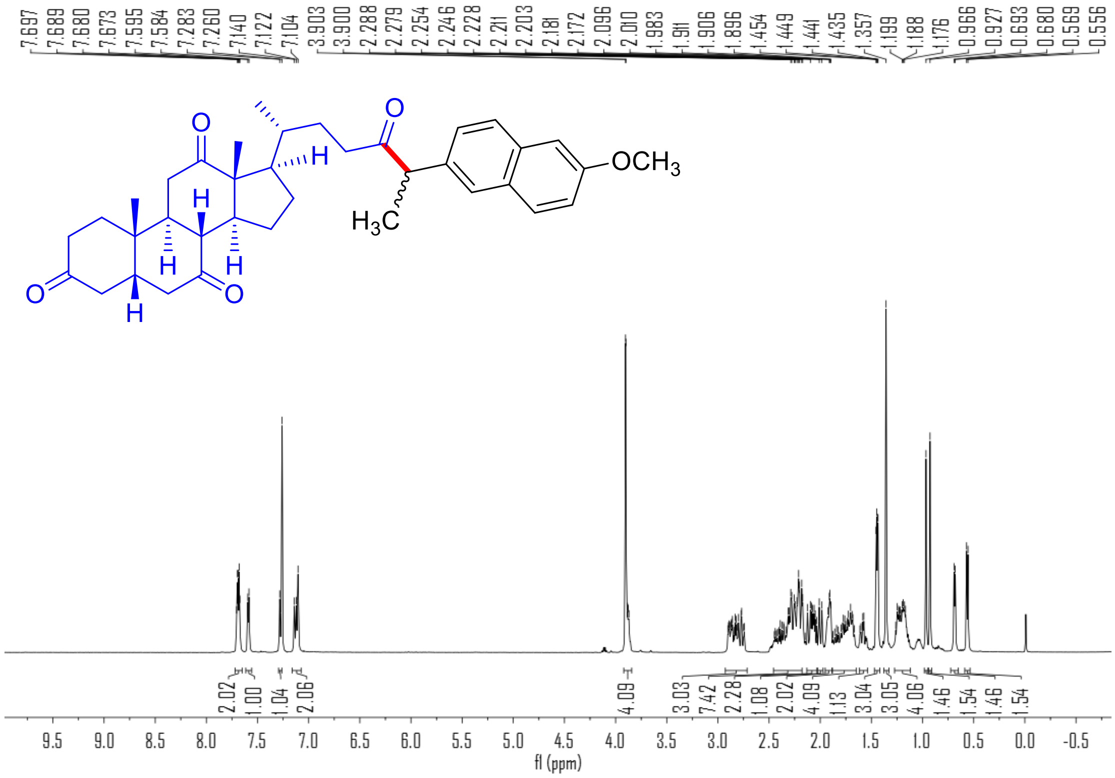

Supplementary Figure 168. ^{1}**H NMR** spectrum of **6h**

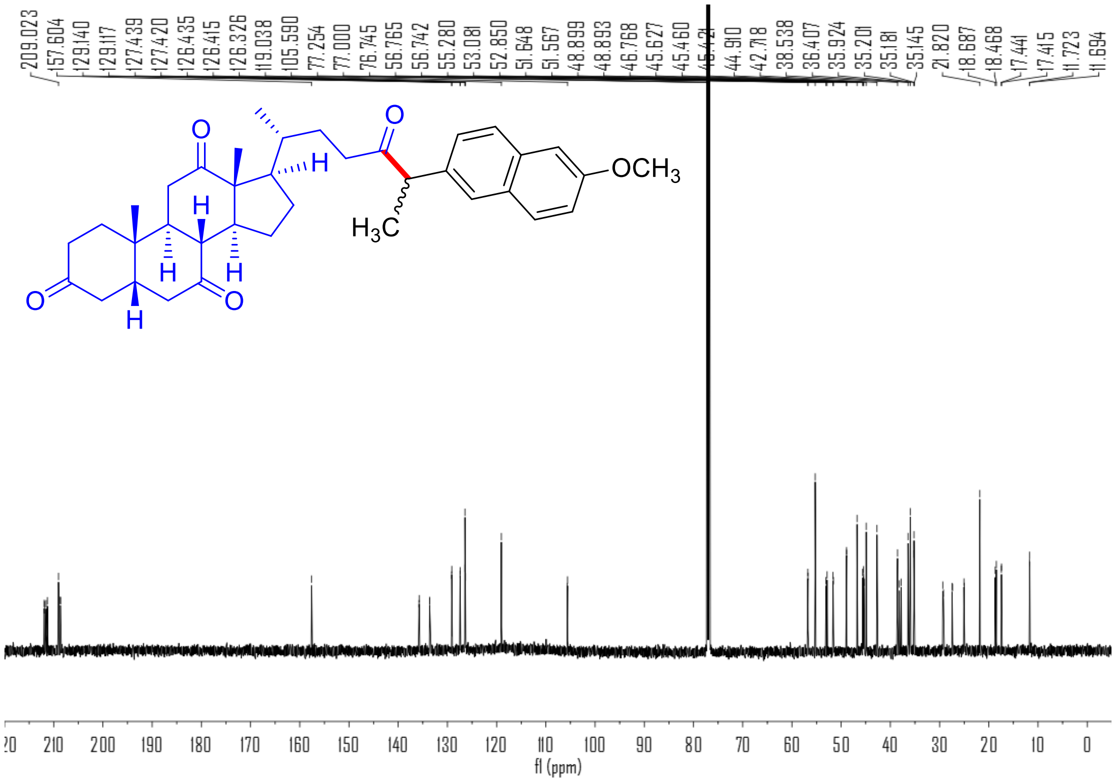

Supplementary Figure 169. ^{13}**C NMR** spectrum of **6h**

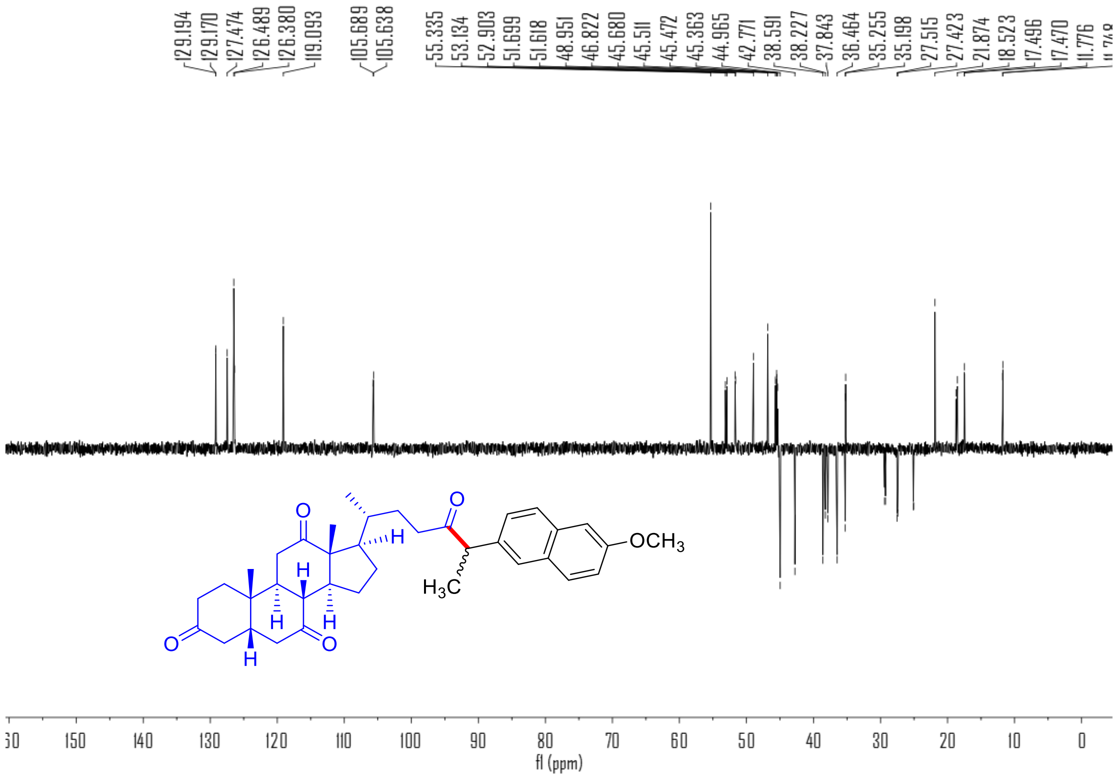

Supplementary Figure 170. DEPT135 spectrum of **6h**

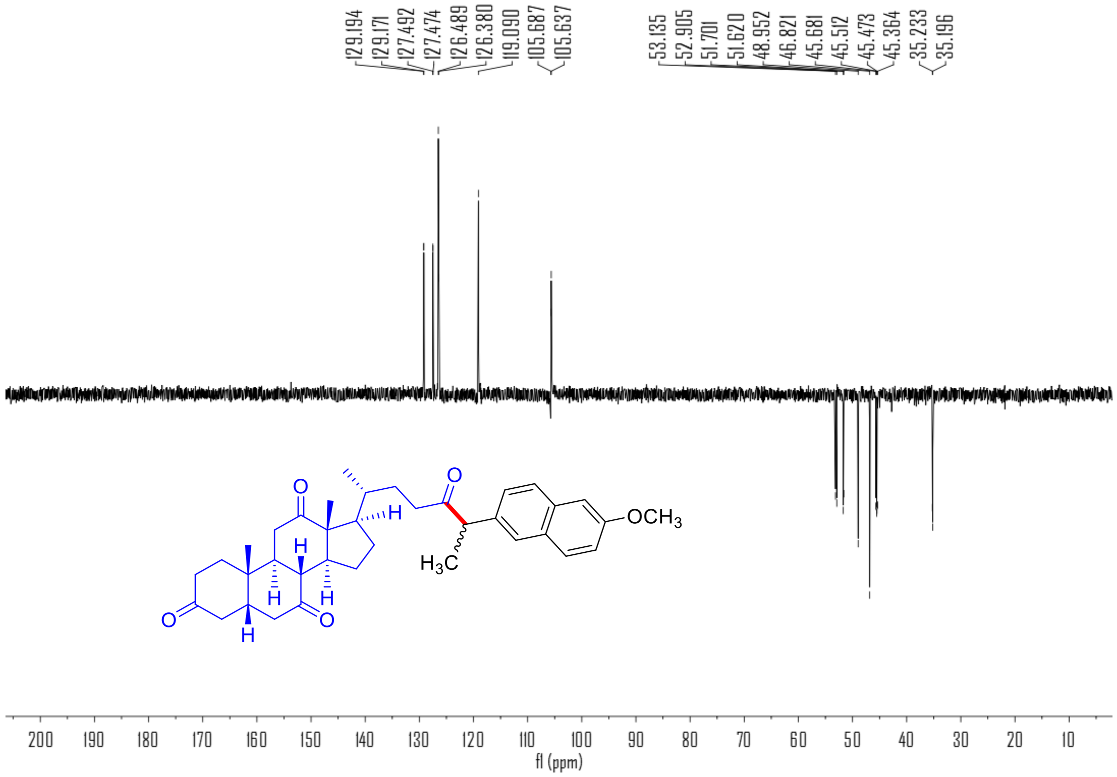

Supplementary Figure 171. DEPT90 spectrum of **6h**

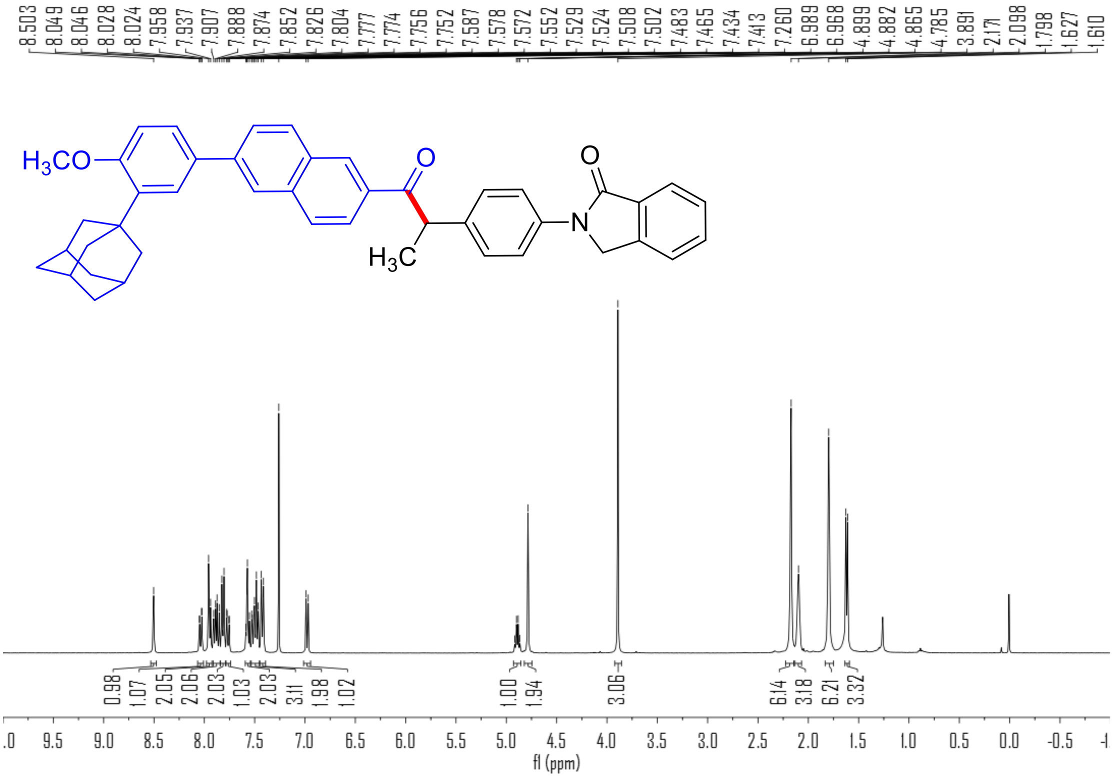

Supplementary Figure 172. ^{1}**H NMR** spectrum of **6i**

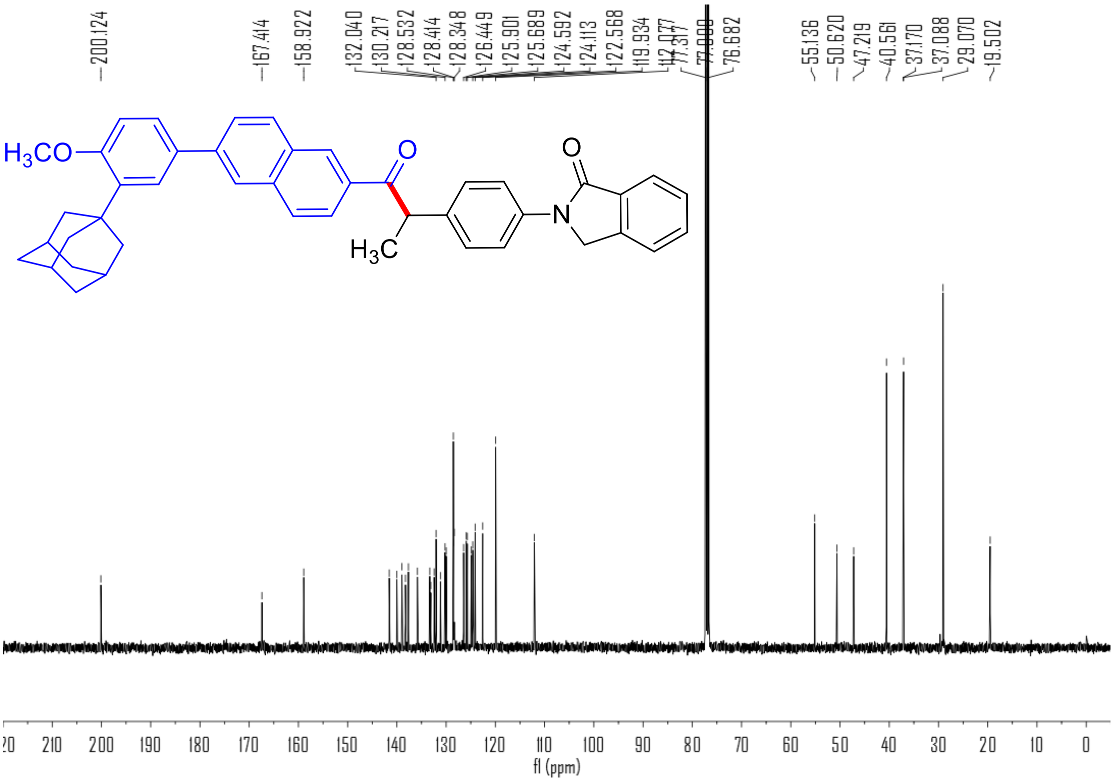

Supplementary Figure 173. ^{13}**C NMR** spectrum of **6i**

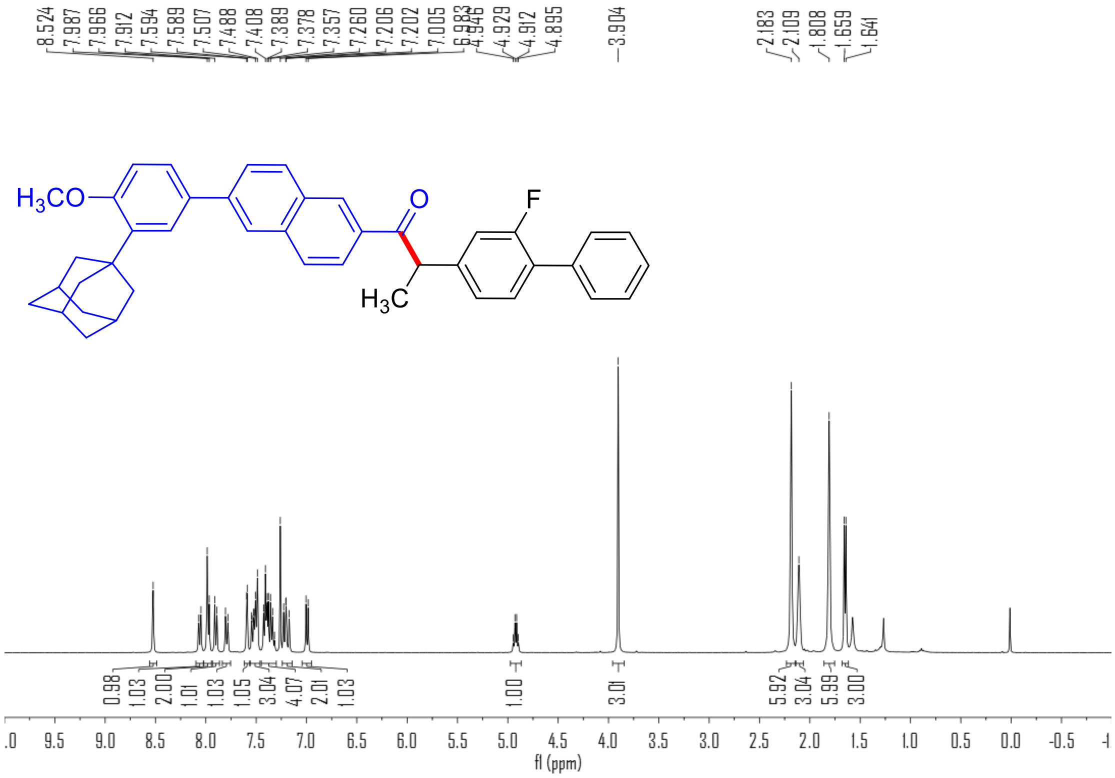

Supplementary Figure 174. ^{1}**H NMR** spectrum of **6j**

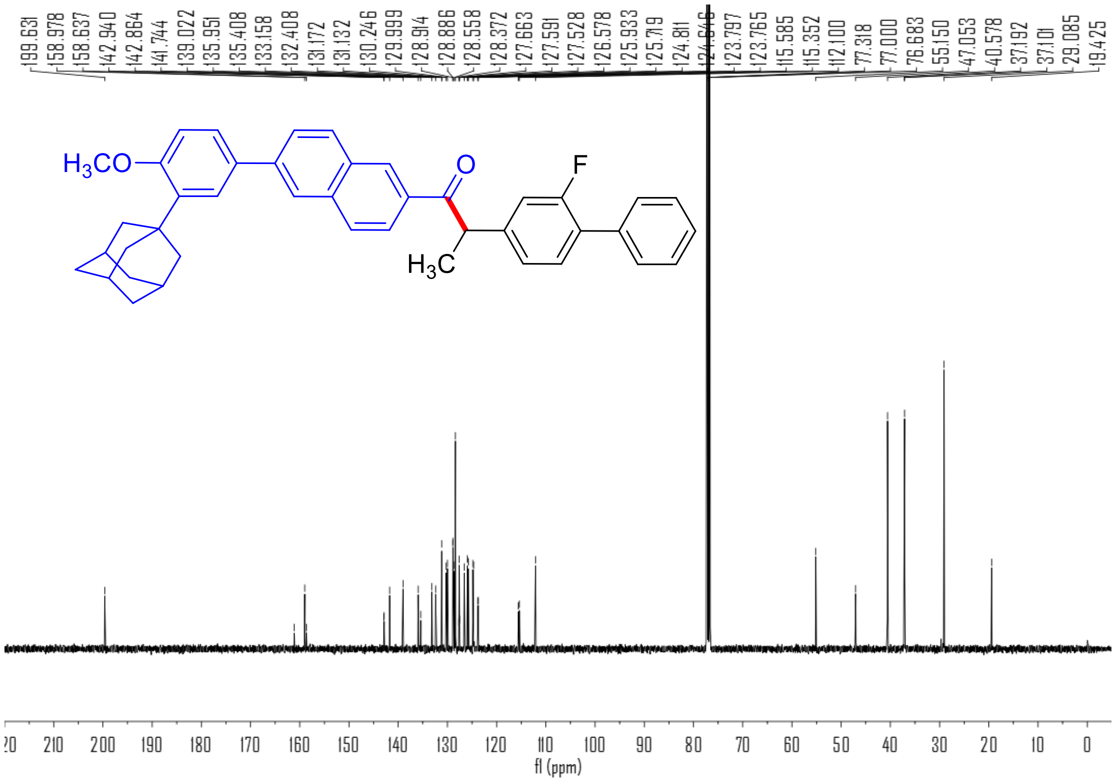

Supplementary Figure 175. ^{13}**C NMR** spectrum of **6j**

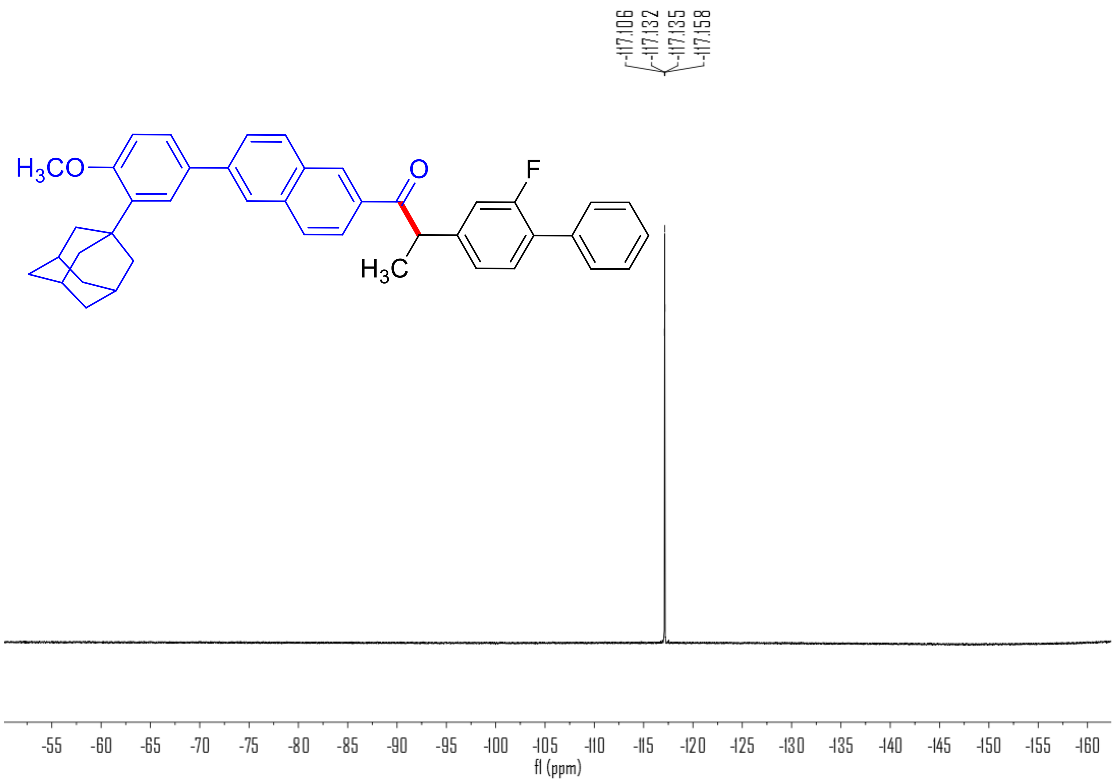

Supplementary Figure 176. ^{19}**F NMR** spectrum of **6j**

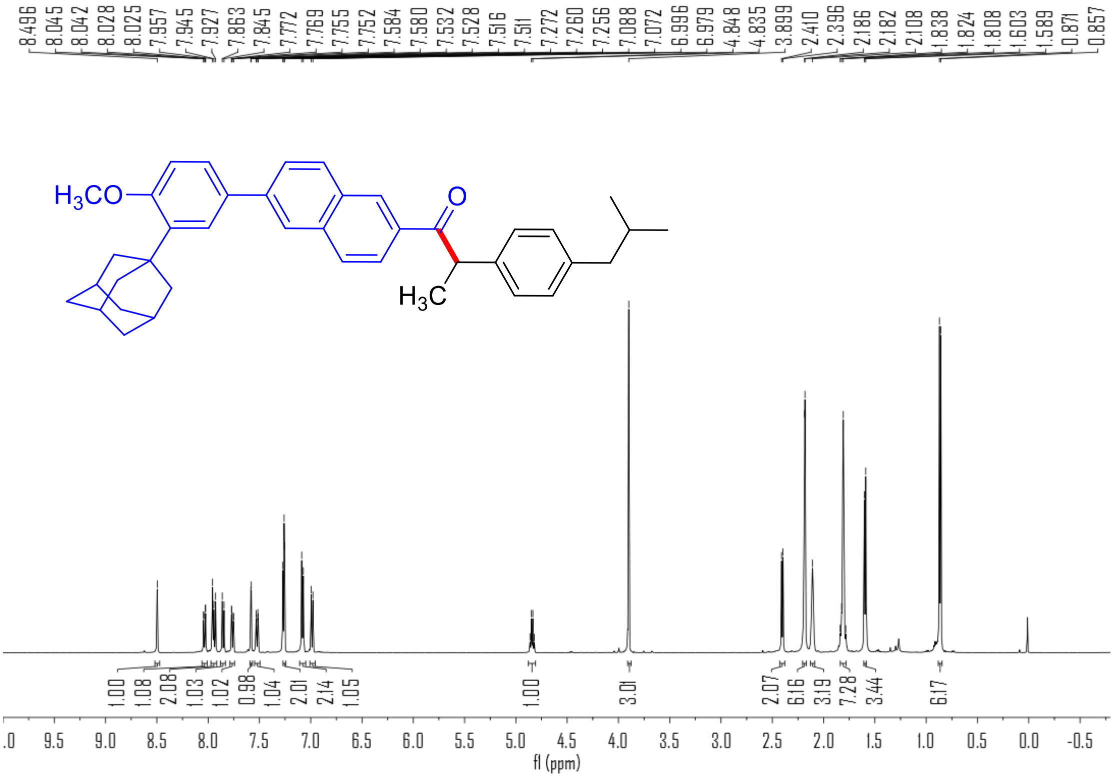

Supplementary Figure 177. ^{1}**H NMR** spectrum of **6k**

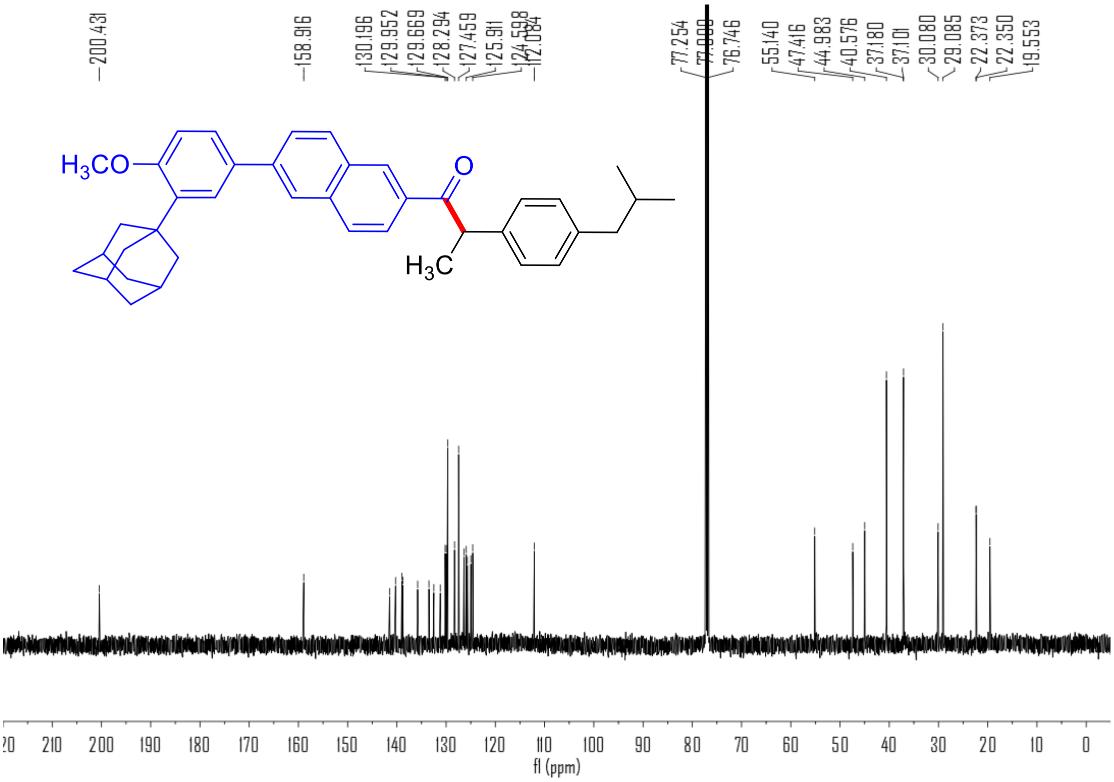

Supplementary Figure 178. ^{13}**C NMR** spectrum of **6k**

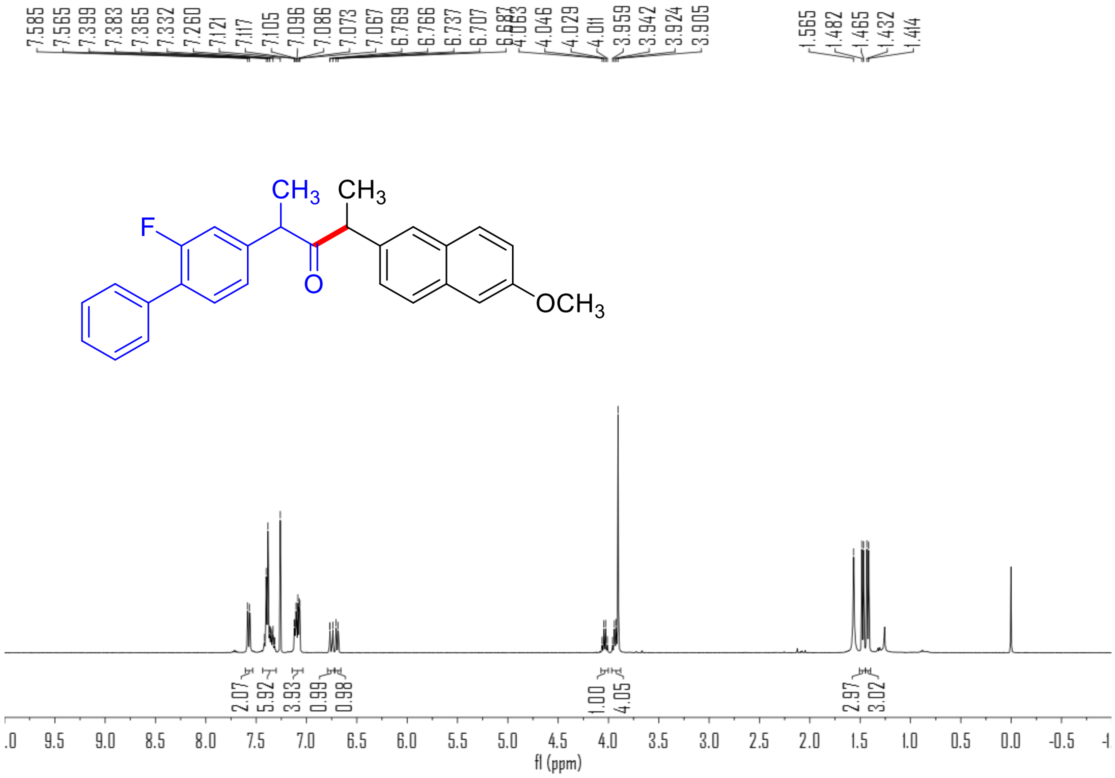

Supplementary Figure 179. ^{1}H NMR spectrum of **6l**

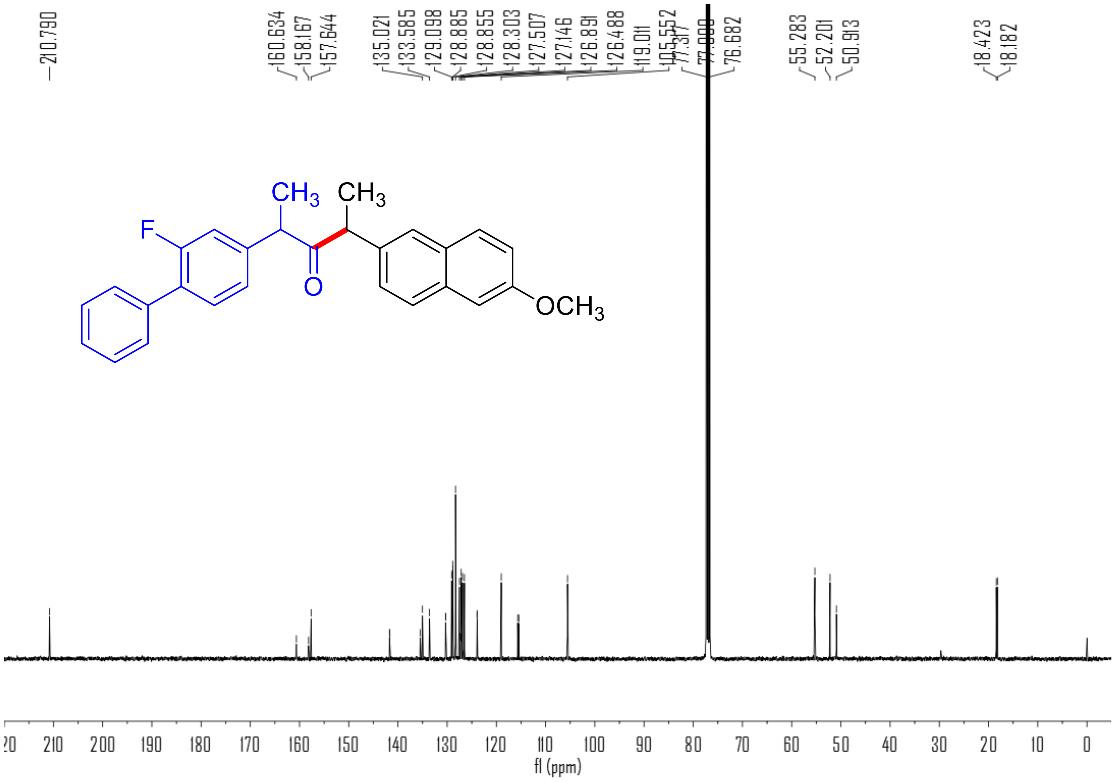

Supplementary Figure 180. ^{13}C NMR spectrum of **6l**

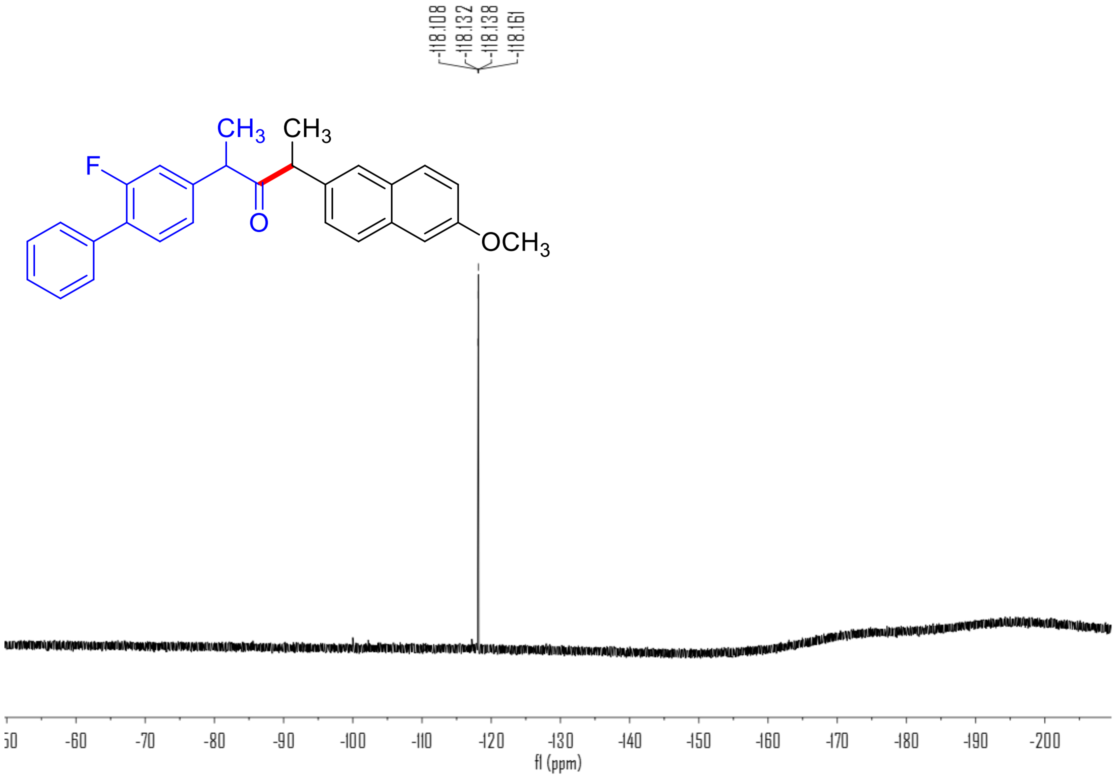

Supplementary Figure 181. ^{19}**F NMR** spectrum of **6l**

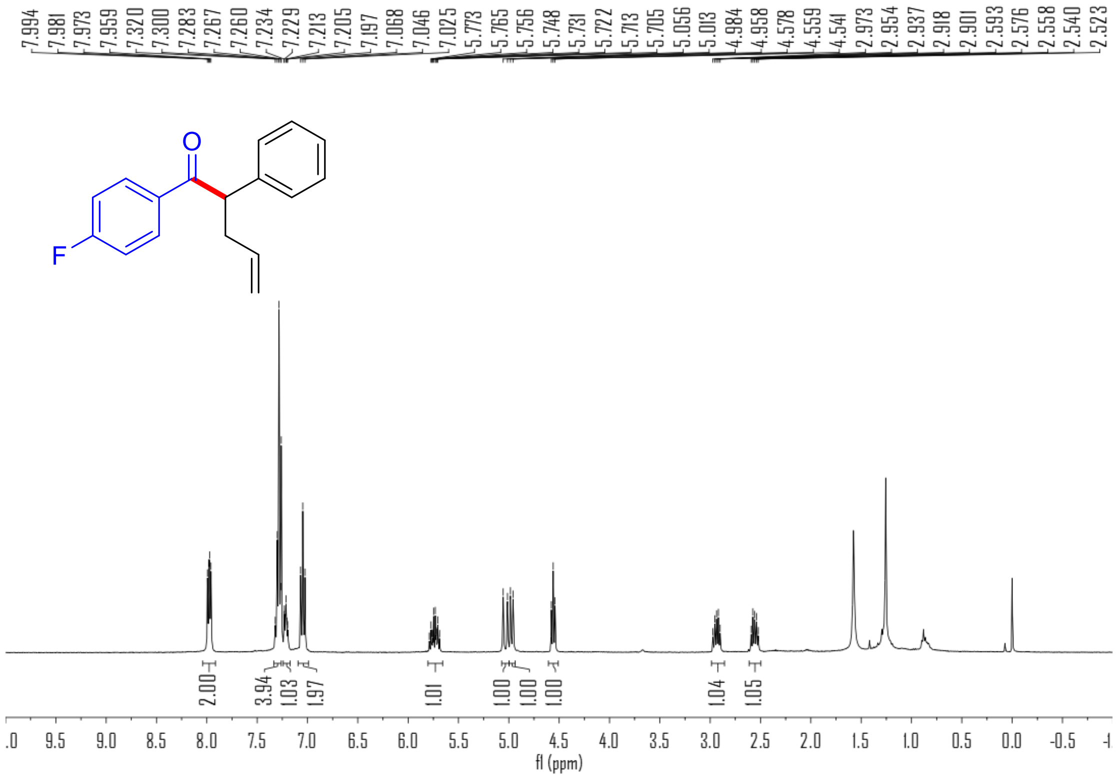

Supplementary Figure 182. ^{1}**H NMR** spectrum of **7**

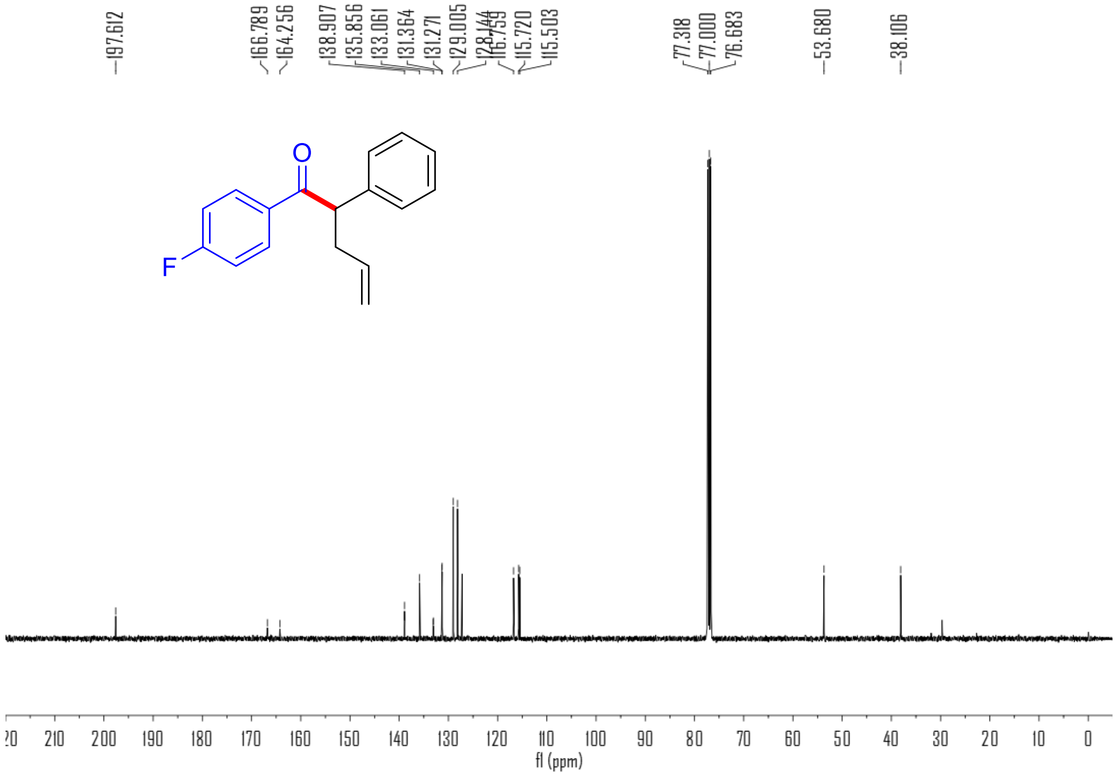

Supplementary Figure 183. ^{13}**C NMR** spectrum of **7**

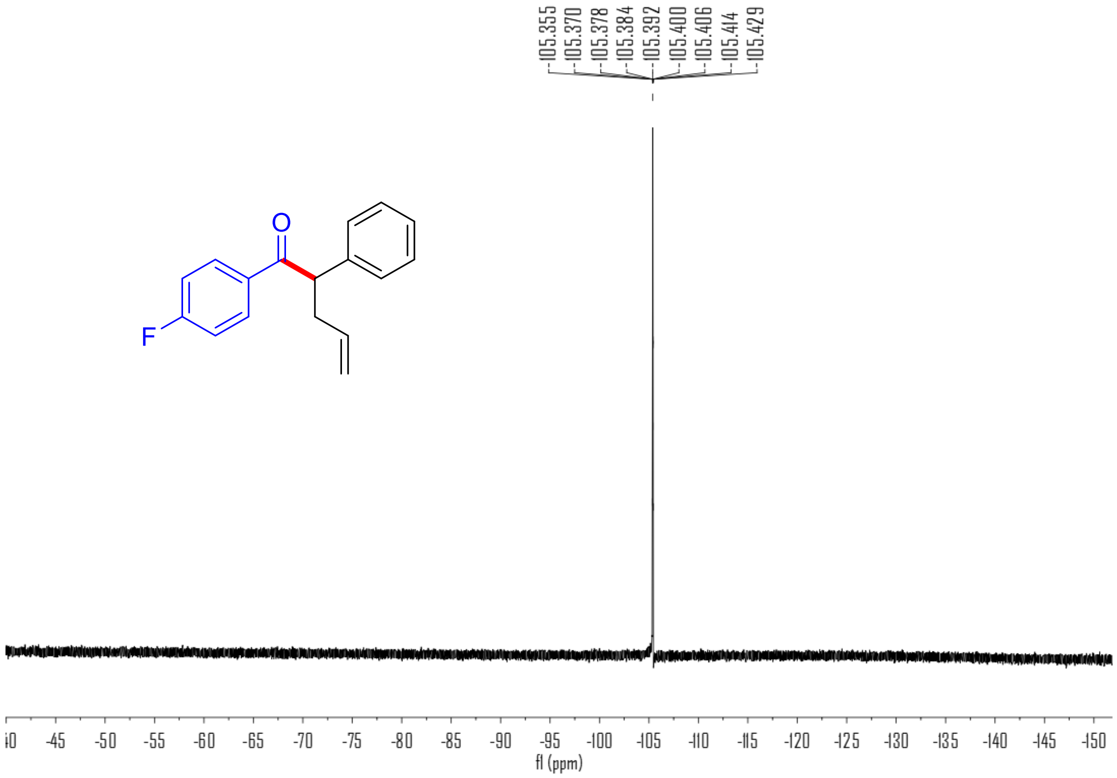

Supplementary Figure 184. ^{19}**F NMR** spectrum of **7**

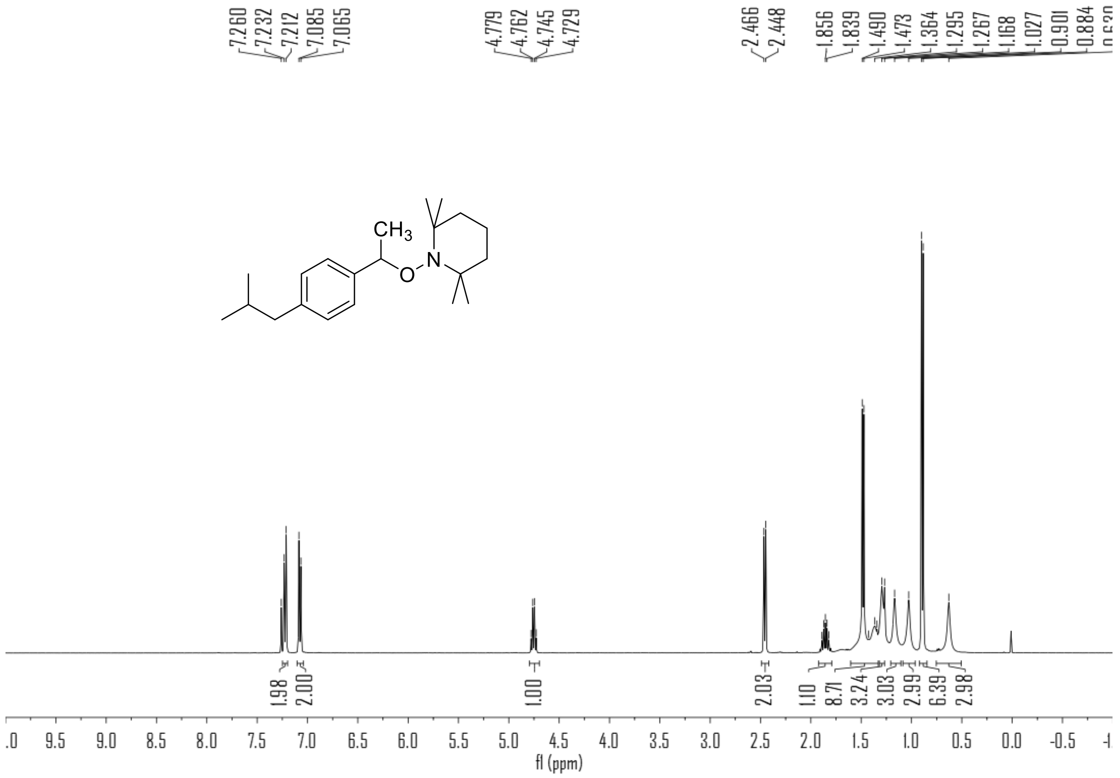

Supplementary Figure 185. ^{1}H NMR spectrum of **TEMPO-adduct**

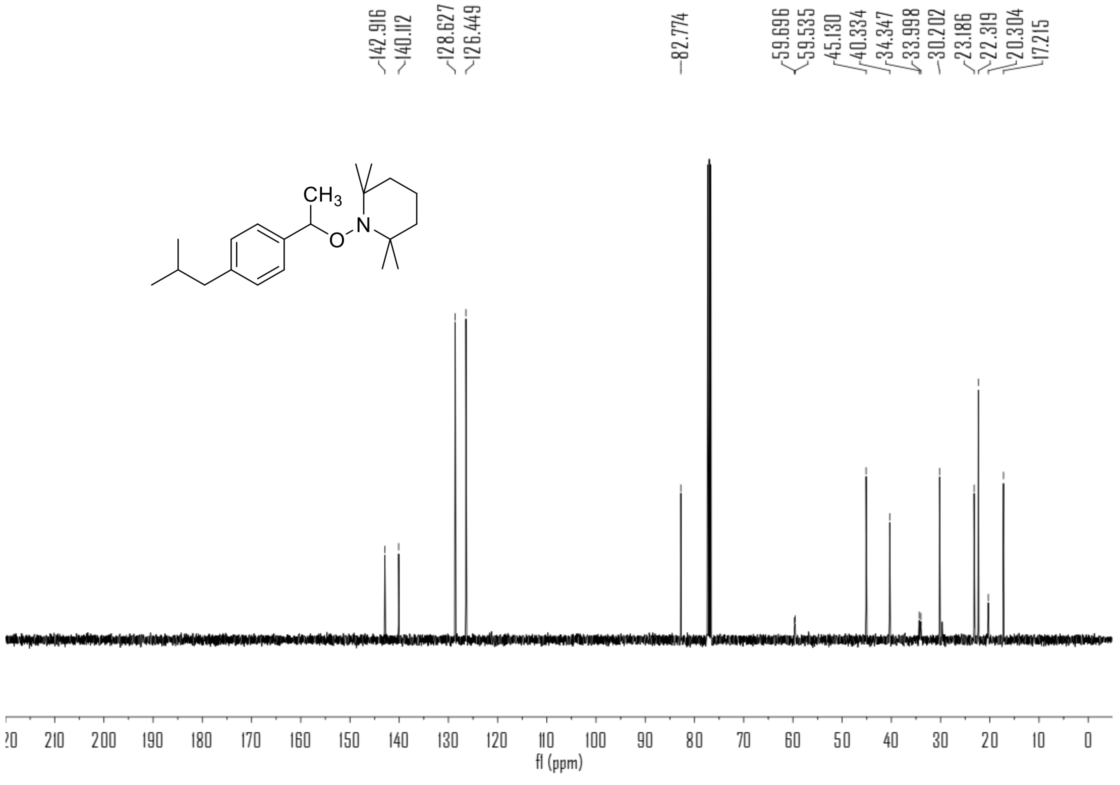

Supplementary Figure 186. ^{13}C NMR spectrum of **TEMPO-adduct**

4. Supplementary references

1. Bryden, M. A., Zysman-Colman, E. Organic thermally activated delayed fluorescence (TADF) compounds used in photocatalysis. *Chem. Soc. Rev.* **50**, 7587–7680 (2021).

2. Lee, A., Scheidt, K. A. *N*-Heterocyclic carbene-catalyzed enantioselective annulations: a dual activation strategy for a formal [4+2] addition for dihydrocoumarins. *Chem. Commun.* **51**, 3407-3410 (2015).

3. Thai, D. L., Sapko, M. T., Reiter, C. T., Bierer, D. E., Perel, J. M. Asymmetric Synthesis and Pharmacology of Methylphenidate and Its Para-Substituted Derivatives. *J. Med. Chem.* **41**, 591-601 (1998).
